# Two-Dimensional Ultrathin Silica Films

**DOI:** 10.1021/acs.chemrev.1c00995

**Published:** 2022-06-22

**Authors:** Jian-Qiang Zhong, Hans-Joachim Freund

**Affiliations:** † School of Physics, Hangzhou Normal University, No. 2318, Yuhangtang Road, Hangzhou, 311121 Zhejiang, China; ‡ Fritz-Haber-Institut der Max-Planck-Gesellschaft, Faradayweg 4-6, 14195 Berlin, Germany

## Abstract

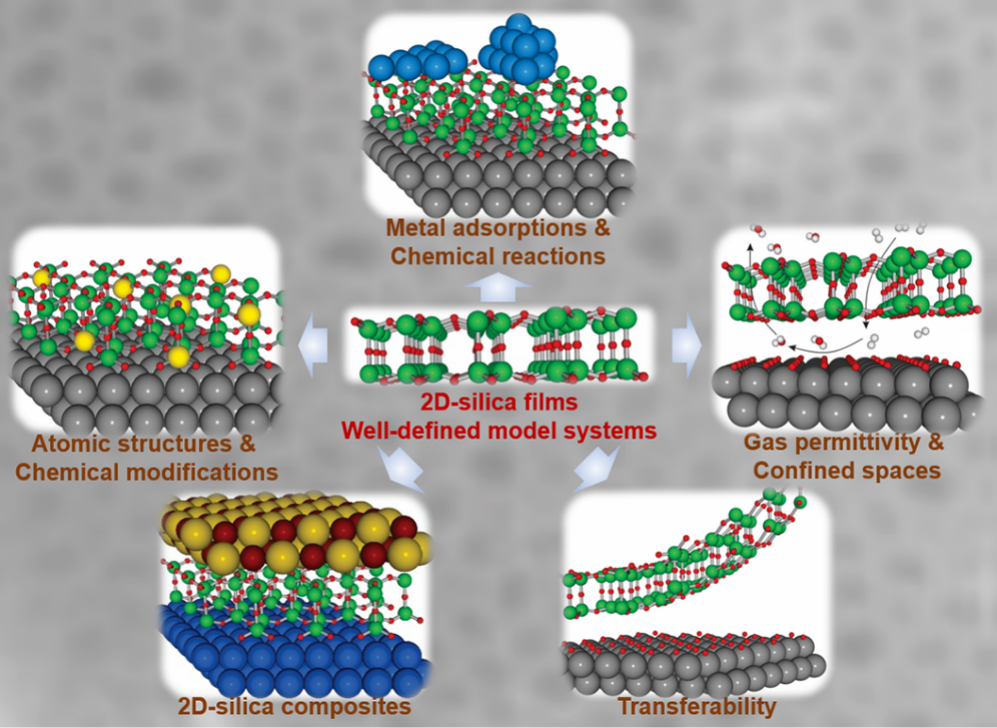

Two-dimensional (2D) ultrathin silica films have the potential to reach technological importance in electronics and catalysis. Several well-defined 2D-silica structures have been synthesized so far. The silica bilayer represents a 2D material with SiO_2_ stoichiometry. It consists of precisely two layers of tetrahedral [SiO_4_] building blocks, corner connected via oxygen bridges, thus forming a self-saturated silicon dioxide sheet with a thickness of ∼0.5 nm. Inspired by recent successful preparations and characterizations of these 2D-silica model systems, scientists now can forge novel concepts for realistic systems, particularly by atomic-scale studies with the most powerful and advanced surface science techniques and density functional theory calculations. This Review provides a solid introduction to these recent developments, breakthroughs, and implications on ultrathin 2D-silica films, including their atomic/electronic structures, chemical modifications, atom/molecule adsorptions, and catalytic reactivity properties, which can help to stimulate further investigations and understandings of these fundamentally important 2D materials.

## 1 Introduction

**Figure 1:**
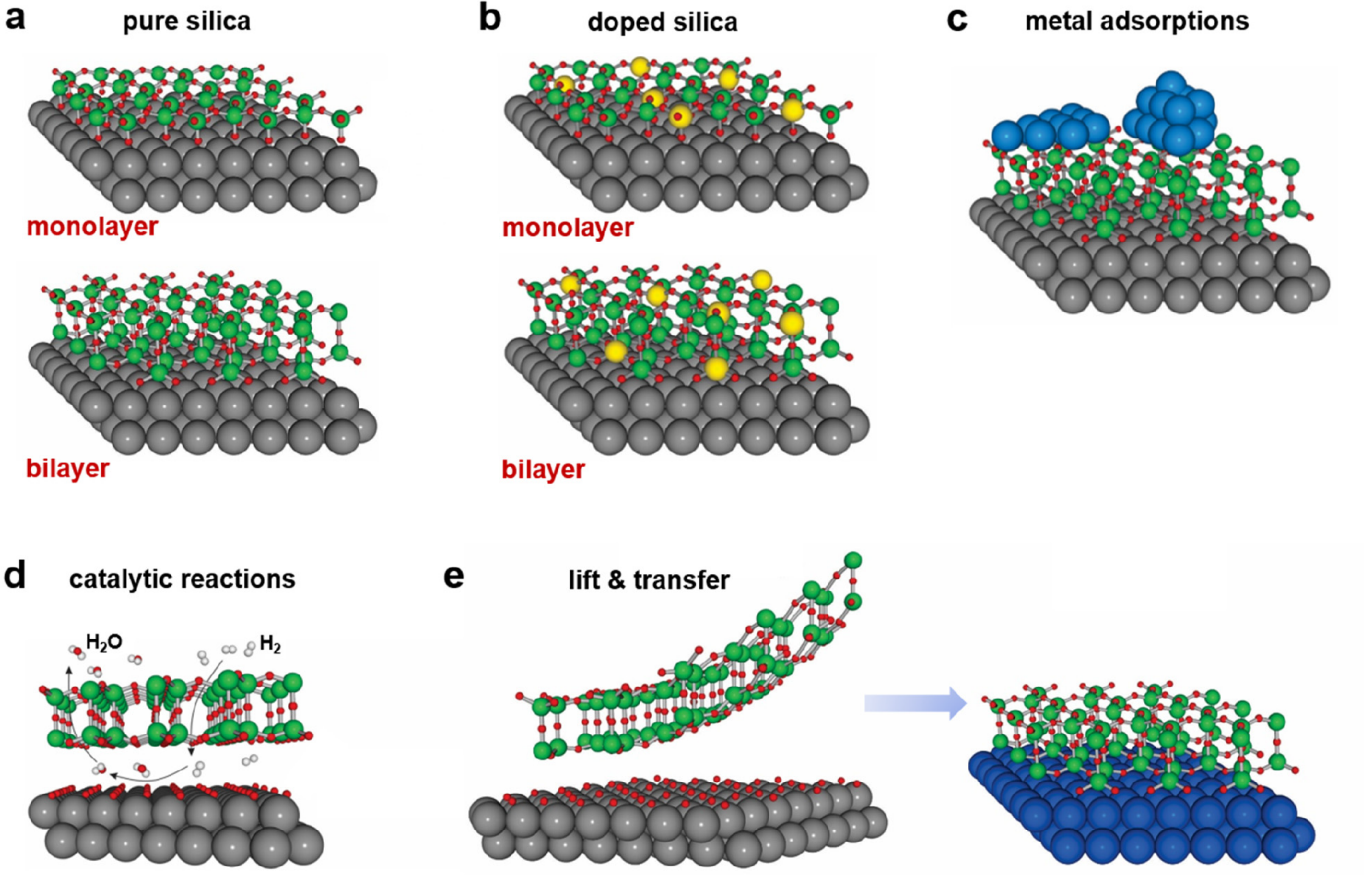
(a) Schematic representation of a silica monolayer and a silica bilayer on metal substrates. The monolayer is chemically bound to the substrate through oxygen atoms of the corner-shared tetrahedra. In contrast, the bilayer is only attached to the substrate via dispersive forces (O, red ball; Si, green ball; substrate, gray ball). (b) Schematic indication of a modification of the silica monolayer and bilayer through replacement of Si atoms by dopants (e.g., Al or Ti) (dopants, yellow ball). (c) Schematic representation of a further modification of the bilayer by anchoring small metal clusters on the surface (clusters, blue ball). (d) Schematic representation of a reaction in confined space. H_2_ may diffuse through the bilayer and react with adsorbed atomic oxygen to form water, which may escape through the openings in the layer into the gas phase. (e) The bilayer may be lifted off the substrate that it was grown on and deposited on a different substrate.

Silicon dioxide (SiO_2_), also known as silica, is one of the most abundant substances exhibiting a variety of complex structures. As an essential raw material for modern technology, silica has been widely used in microelectronics and catalysis.^1,2^ Its function depends on many parameters, including structure.^3^ In order to shed light on the structure–function relationship, it is necessary to develop systems with variable structure and perfection, so that it is possible to study them with a variety of experimental techniques and tools at the atomic scale.^4^ Before we address specific systems and aspects in six sections and address the historical development in section 1, let us consider the general approach in more detail (Figure 1), as it allows us to identify the crucial ingredients and the perspectives of the approach, as well as the outline of this Review.

Some schematic structures of 2D-silica films are captured in Figure 1a. There is a base-support, typically a single crystal of a material (often a metal) onto which silicon is evaporated and oxidized to form a well-ordered silica overlayer. In the case of the monolayer (ML) (Figure 1a, top), the structure may be described as a network of corner-shared [SiO_4_] tetrahedra forming interconnected 6-membered Si–O rings in the plane and the remaining six oxygen atoms bound to silicon interacting with the substrate.^5^ The film stoichiometry is SiO_2.5_ according to the size of the surface unit cell. The prepared monolayers may be grown thicker, approaching overall SiO_2_ stoichiometry. However, experience shows that this degrades the film’s structural quality, as we will discuss in section 1.1. The exception is a bilayer (BL) (Figure 1a, bottom). Here, a structure is formed that may be looked at as two monolayers bound to each other through the oxygen atoms forming a bilayer, which is then attached to the substrate.^6^ Its stoichiometry is SiO_2_! Another unique feature of this bilayer is that only the dispersive forces dominate the interactions at the interface to the metal substrate.

The quality of the created films, naturally, depends on structural parameters at the support interface and the formed oxide.^7,8^ Once well-structured systems have been formed, they may be studied at the atomic level with many spectroscopic and structure determining experimental tools, both in ultrahigh vacuum (UHV) and under ambient conditions, including scattering experiments, vibrational and electronic spectroscopy, diffraction, and scanning probe techniques, as will be covered in section 2. Subsequently, the system may be modified in a controlled way (Figure 1b), for example, by substituting the Si atoms with Al or Ti.^9−11^ Al substitution leads to models mimicking zeolite films. The structure–function relations of those modified systems may be studied (see section 3) in the same way as the pure silica films, of course. In taking the spirit of such a methodology even further, a so-called model catalyst approach^12−15^ has been developed within the field of heterogeneous catalysis (sections 4 and 5) in order to capture the intrinsic features of the real heterogeneous powder catalysts, which, however, are too complex to be studied at the atomic level. Within this approach, a support, i.e., silica, is modeled by a thin silicon oxide film, and subsequently, the active component, for example, metal nanoparticles, is added (Figure 1c). Such systems are then studied at the same level as the pure films. This approach allows the identification of the relevant parameters determining the properties of a real powder catalyst at the atomic level systematically. As silica is a relevant support and an active component in heterogeneous catalysis, it is an important material to be studied within such an approach.

An area of potential appeal for those researchers interested in understanding basic phenomena and concepts in heterogeneous catalysis is related to chemistry in zeolites.^16,17^ The reason is connected with the discovery that one may create a bilayer silica film, as discussed above, which, due to the presence of the corner-sharing network, possibly modified by incorporating Al atoms, also constitutes a permeable membrane (crystalline or vitreous), through which molecules may diffuse to the metal surface and there react with species adsorbed on it.^18^ Given the small space left between the metal surface and the 2D-silica layer, this defines a situation often found for reactions in confined space (Figure 1d, using water formation as an example). Such ideas had been proposed before for systems involving weakly bound graphene flakes on metal surfaces,^19−21^ leaving metal surfaces open to adsorption. In this case, the diffusion of molecules may only occur from the flake edge, while for the silica bilayer, diffusion through the silica network is an option, which will be discussed in section 5.

Interestingly, the bilayer films may also come in a vitreous form, and the flat nature of the bilayer film allows us for the first time to structurally completely characterize at the atomic level using scanning probe techniques, which opens up the possibility of studying the crystal–glass transition at the atomic scale.^22,23^ The work is still in progress and holds potential for rather fundamental work. We will touch on this aspect in detail in section 2.2. However, as we will see in the following section, the initial studies on silica films have been performed in connection with microelectronics, as amorphous and polycrystalline silica forms on silicon wafers through exposure to oxygen.^24−27^ The current approach to growing silica films may open up unexpected possibilities in microelectronics. A surprising property of the bilayer film is that those vitreous films may be peeled off from their metallic substrates if the dispersive forces are minimized by keeping an adsorbed layer of oxygen atoms on the metal substrate (Figure 1e).^28^ After lift-off (as will be discussed in section 6), they may be relocated to other substrates, offering exciting perspectives in creating new complex systems potentially valuable for microelectronics, as silica films, even if only two-layer thick, are wide-band-gap insulators.^29^

The various aspects mentioned in this short introductory section open the way for surface science to contribute potentially significantly to our understanding of fundamental phenomena in fields typically not identified as playgrounds for surface science. The current Review provides a detailed summary of what has been achieved so far and offers several perspectives where studies on silica films may lead to new fundamental knowledge.

### 1.1 Historical Overview of the Silica Growth

**Figure 2:**
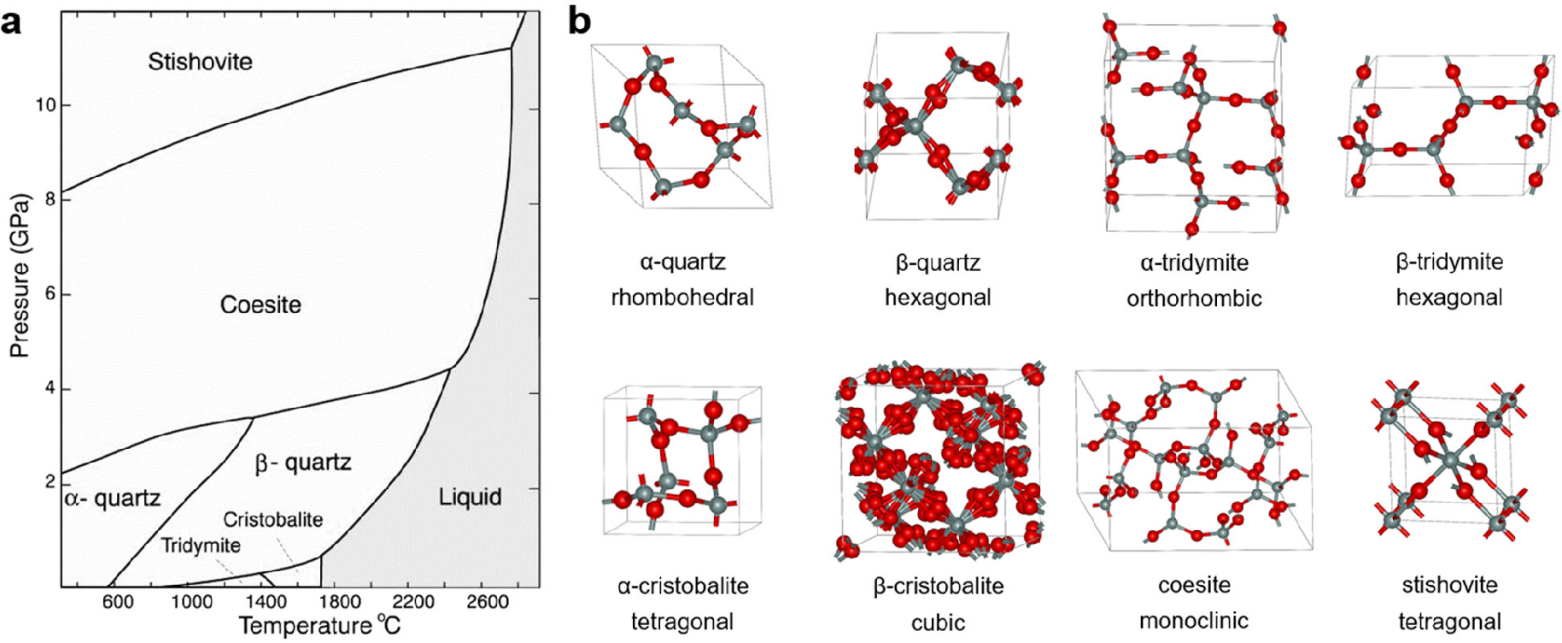
(a) Phase diagram of the most common silica polymorphs. (b) Collection of some crystalline forms of SiO_2_.

As already alluded to above, in the semiconductor industry, understanding the electronic properties of silica films is essential if novel transistors are to be built. Intensive research efforts have been devoted to miniaturizing these electronic devices by synthesizing ultrathin silica layers, where the latter were used as gate dielectrics.^1,2^ In the field of catalytic industry, silica is used as a catalyst or catalyst support.^30^ The catalytic performance of silica or silica-derived materials in heterogeneous catalysis is primarily determined by their mesoporosity and the specific atomic structures.^16,17^ Silica comes in various crystalline forms, such as α-quartz, β-quartz, cristobalite, tridymite, and high-pressure variants (e.g., coesite and stishovite) (Figure 2).^31,32^ Except stishovite, most polymorphs involve the tetrahedral [SiO_4_] units, which are connected through either the corner, edge, or face sharing.

Silica thin films may be assembled via those SiO_2_ crystalline structures, which is true in a similar sense for almost all oxides.^33^ However, silica can also occur as stable vitreous structures. The best known is, of course, silica glass, which is used in many fields of science and technology and many areas of human life and our environment.^34^ Thin films of vitreous silica form in many cases, for example, when silicon is exposed to oxygen at appropriate conditions. Those films are used in device technologies.^35^ The atomic structure of those films, however, is not well understood, and we will come back to this topic (see below).^36−38^ It is noteworthy that many oxides do not form stable glasses. The most prominent glass formers are silicon dioxide and diboron trioxide.^39^

Understanding the structure of vitreous silica has a long history. Until the beginning of the 1930s, it was, based upon X-ray studies, assumed that the vitreous material consists of microcrystallites. W.H. Zachariasen,^36^ however, discussed obvious inconsistencies in the conclusions from X-ray studies and proposed, instead, three-dimensional (3D) network structures with randomly connected [SiO_4_] tetrahedrons, which can be formed by rapid melt quenching. A notable example of vitreous silica is silicate glass.^40^ Fused silica is a glass made of pure silica without containing other ingredients. Although having been discussed for a long time, detailed knowledge of the atomic structure of vitreous silica is still in an “embryonic” state,^36−38^ based on a combination of measurements of pair distribution functions (PDF) and the modeling of those.^41,42^ We will see in section 2.2.1.2 how the structure of vitreous silica, as proposed by Zachariasen, is connected to 2D vitreous silica thin films.

**Figure 3:**
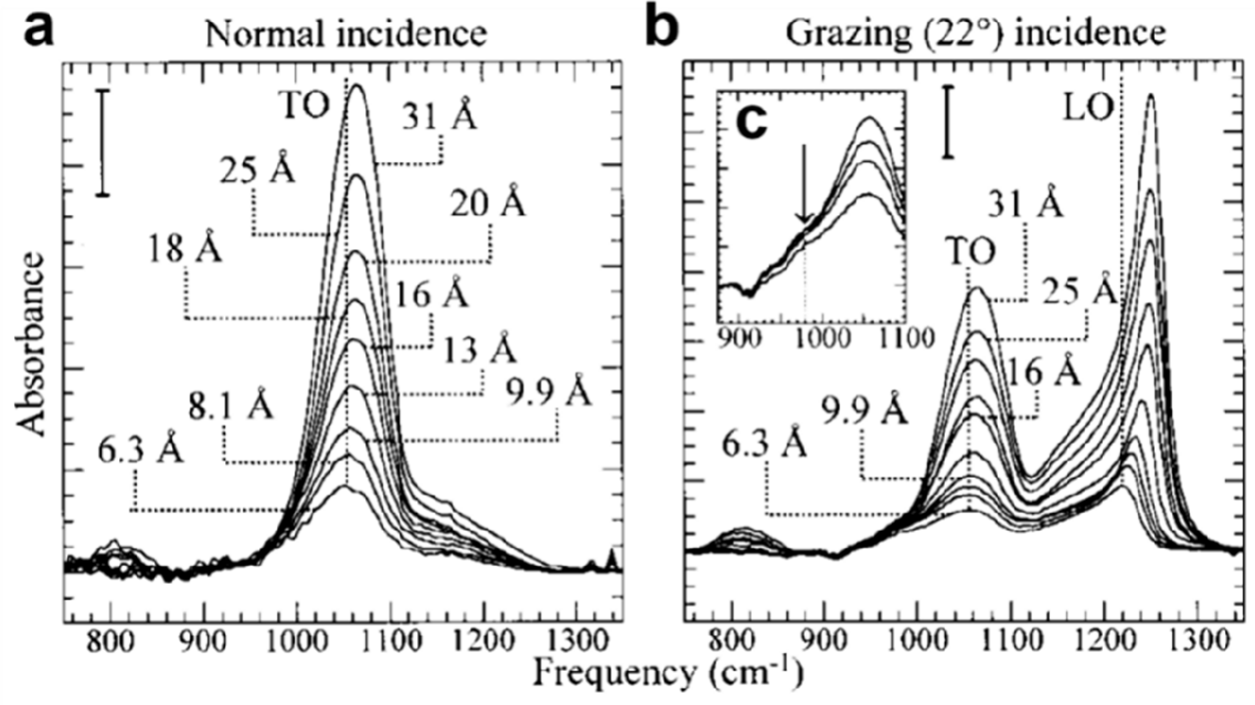
IRAS analysis of the thermally grown SiO_2_ films with the infrared beam incident at (a) normal and (b) grazing to the sample surface. The SiO_2_ film was uniformly thinned from 3.1 to 0.63 nm. (c) The inset shows the spectra with grazing incidence for the four thinnest films. Reproduced with permission from ref (47). Copyright 2000 AIP Publishing.

Silica thin films with different structures, including amorphous and microcrystalline, can be prepared on Si single crystal surfaces via direct oxidation. As motivated by the pivotal role of the SiO_2_/Si interface in metal-oxide-semiconductor field-effect transistor (MOSFET) technology, many studies were carried out to address the details of silica film growth and its structural and electronic properties.^35^ An important aspect of those studies was to investigate the properties of those films as a function of thickness, as the latter strongly influences the properties and function of MOSFETs.^43^ The atomic structure of those films is complex, as revealed through infrared reflection–absorption spectroscopy (IRAS) data as shown in Figure 3. The interpretation is based on calculations of the amorphous silica film structure^44,45^ and allows for identifying surface phonon modes and functionalities. The comparison of normal and grazing incidence data enables the identification of transverse optical (1000–1120 cm^–1^) and longitudinal optical (1180–1300 cm^–1^) modes and their shift as a function of layer thickness. The substoichiometric silicon oxide species that may be identified through the analysis of the spectra account for the shifts and the interfacial layer constitution.^46^

**Figure 4:**
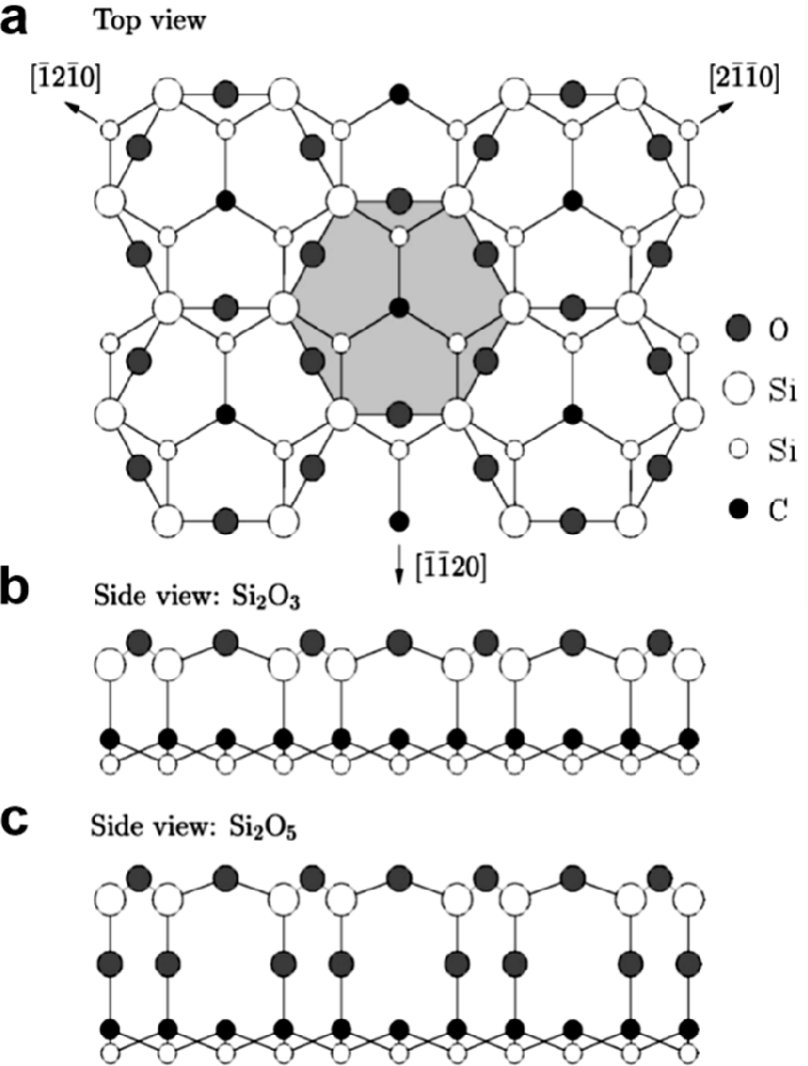
(a) Schematic top view of the silica adlayers on 6H-SiC(0001̅). Side view of (b) Si_2_O_3_ and (c) Si_2_O_5_ silica adlayers. Reproduced with permission from ref (50). Copyright 2000 American Physical Society.

Heinz and co-workers first reported a well-ordered silica layer formed on the SiC(0001̅) surface.^48,49^ Based on a low-energy electron diffraction (LEED) study on an ordered silica (√3 × √3)R30° overlayer grown on SiC(0001̅), the authors came to the conclusion via an I/V LEED analysis that the overlayer has a Si_2_O_3_ structure as shown in Figure 4b, where an ordered layer is bound directly via the Si atoms to the carbon atoms in the silicon-carbide surface. Based on *ab initio* pseudopotential calculations, Pollmann and co-workers concluded a different structure that, giving rise to the same (√3 × √3)R30° pattern, is more stable.^50^ In this structure shown in Figure 4c, the silica layer is of Si_2_O_5_ stoichiometry and is bound via oxygen atoms to the silicon-carbide substrate. We will see in section 2.1.1 that a similar structure has also been verified for a silica layer bound to a Mo(112) surface, where a Si_2_O_5_ silica film is bound via the oxygen atoms to the metal substrate.^5^

### 1.2 Epitaxial Growth of the Silica Films on Metal Surfaces

**Figure 5:**
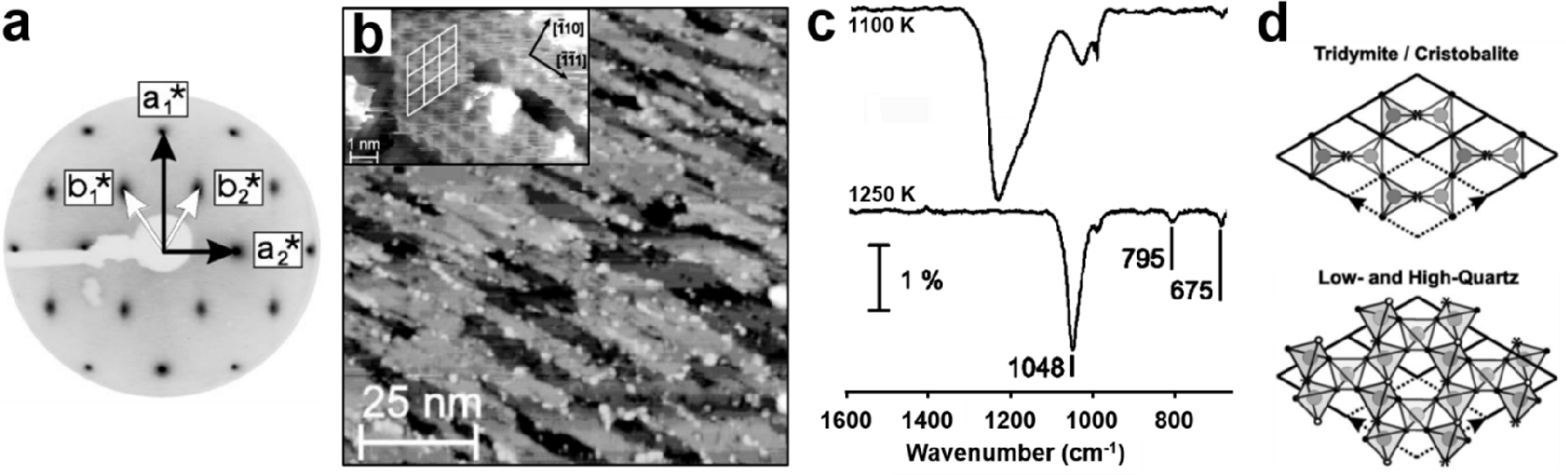
(a) Typical LEED pattern of a Mo(112)-supported well-ordered silica film. Unit cells of silica and Mo(112) are indicated by black and white arrows, respectively. (b) Scanning tunneling microscopy (STM) image of the silica/Mo(112) after UHV annealing at 1250 K (100 × 100 nm^2^, *U*_s_ = −4.9 V). The inset shows the high-resolution STM image of a flat silica terrace (5 × 8 nm^2^, *U*_s_ = −0.5 V). (c) IRAS of the silica/Mo(112) surface after annealing at 1100 and 1250 K, respectively. (d) Structure models of the silica layer. Reproduced with permission from ref (71). Copyright 2002 American Physical Society.

As far as we know, Goodman and co-workers were the first to prepare thin silica films on metal surfaces.^51−62^ Before this, only adventitious silica on metal surfaces had been observed.^63−66^ In the Goodman work, molybdenum single crystals were chosen as the substrates as the metal is easy to clean by thermal treatments. By evaporating silicon onto Mo(110) and Mo(100) surfaces in an oxygen environment (∼10^–5^ Torr) and subsequently annealing in ultrahigh vacuum (UHV), silica overlayers are formed. The layer is not well-ordered but has been characterized by several surface-sensitive techniques. The Fritz-Haber group later investigated the formation of silica overlayers on a Mo(112) surface.^67−71^ Some of the results are presented in Figure 5.

**Figure 6:**
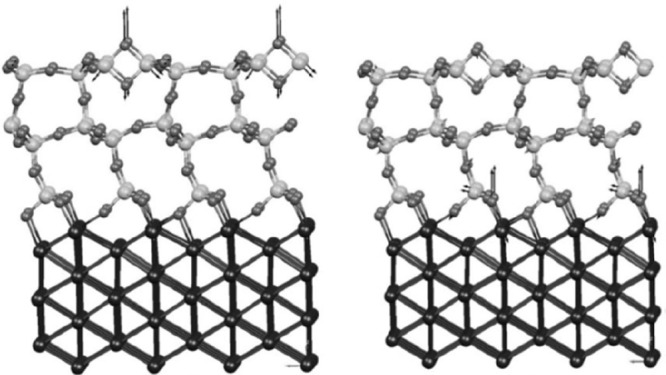
DFT simulated structures of SiO_2_ (cristobalite) on Mo(112). The vibration modes are calculated on the 2-membered silica ring (left, 784 cm^–1^) and at the SiO_2_/Mo(112) interface (right, 677 cm^–1^), respectively. Reproduced with permission from ref (72). Copyright 2004 American Physical Society.

This layer is very well-ordered, and a number of surface science techniques have been applied, including studies by the Goodman group. Based on those investigations, two different structural models have been suggested: a structure where isolated silica tetrahedra are bound to the metal, as opposed to a model where silica tetrahedra are connected to form a 2D network. There was a long debate, but in the end, the silica network has been proven via a combination of detailed STM studies and theoretical calculations. The first density functional theory (DFT) calculations were performed by the Pacchioni group and led to a suggestion as shown in Figure 6.^72^ The final theoretical description was provided by the Sauer group.^5^ The details of those studies led to an understanding of ultrathin silica films on metals, as discussed in section 2 of this Review.

## 2 Atomic Structures of the 2D-Silica

Due to the structural complexity of silica, the determination of its atomic structures was and is challenging.^73^ Various diffraction methods,^74,75^ nuclear magnetic resonance (NMR) methods,^76,77^ and electron microscopy methods^78,79^ have been applied to identify its atomic arrangements in both crystalline and amorphous solids. However, it was impossible to resolve and characterize the real surface morphology at the atomic scale.^80,81^ During the past two decades, modern preparation methods combined with advanced scanning tunneling and atomic force microscopies enabled us to address these complex silica systems.^82,83^ In this section, we attempt to review both experimental and theoretical atomic-scale studies of the ultrathin 2D-silica films available to date (Table 1). We only present pristine 2D-silica films, whereas in section 3, introduction of dopants and chemical functionalization will be discussed.

**Table 1:** Main Breakthroughs in Structural Studies of Ultrathin 2D-Silica Films

year	breakthroughs	characterization methods	refs
2005	ML silica/Mo(112)	STM, IRAS, LEED, XPS, DFT	(5, 84, 85)
2006	ML silica stripes/Mo(112)	STM, IRAS, XPS, DFT	(86, 87)
	ML aluminosilicate/Mo(112)	STM, IRAS, XPS, DFT	(9)
2010	BL silica/Ru(0001)	STM, IRAS, LEED, XPS, DFT	(6)
2012	ML silica/Ru(0001)	STM, IRAS, LEED, XPS, DFT	(88)
	BL silica/Pt(111)	STM, IRAS	(7, 89)
	BL silica/graphene	TEM, EELS, DFT	(80, 81)
	Vitreous silica/Ru(0001)	STM, AFM, LEED, DFT	(90−92)
	BL aluminosilicate/Ru(0001)	STM, IRAS, XPS, DFT	(10)
2013	BL silica/Pd(100)	STM, LEED, AES, DFT	(93, 94)
	Fe-silicate/Ru(0001)	STM, IRAS, LEED, XPS, DFT	(95)
	cellular silica/Ru, Co, and Fe nanoplatelets	TEM, EELS, DFT	(96)
2015	Ti-silicate/Ru(0001)	STM, IRAS, DFT	(11)
	silicatene/silicon-carbide hybrids	STM, IRAS, XPS, DFT	(97, 98)
2016	Ge-silicate	DFT	(99)
	Fe-aluminosilicate/Ru(0001)	STM, IRAS, XPS	(100)
2017	BL silica/Pd(111)	STM, LEED, IRAS, AES, DFT	(101, 102)
	BL silica/Ni_*x*_Pd_1–*x*_(111)	STM, LEED	(103)
	BL aluminosilicate/Pd(111)	STM, LEED, XPS	(101)
2018	zigzag silica/Ru(0001)	STM, IRAS, LEED, XPS, DFT	(104)
2019	Ni-silicate/Ni_*x*_Pd_1–*x*_(111)	STM, LEED, XPS, IRAS, DFT	(8, 105)
2020	ML silica/CuO_*x*_/Cu(111)	STM, STS, DFT	(106)
2022	BL silica/Au foil, and Pd foil	STM, IRAS, LEED, XPS, TEM	(107, 108)

### 2.1 Monolayer Structures

The investigations on metal-supported silica films were, as mentioned above, started in Goodman’s group. Molybdenum single crystals were chosen as the substrates. Silica films can be synthesized by evaporating silicon in ambient oxygen onto Mo(110) or Mo(100) substrates. With increasing Si deposition, a complete monolayer is formed, followed by either a layer-by-layer or a three-dimensional film growth.^51−53^ The obtained silica films with a thickness of a few nanometers were proposed to be amorphous and consist of short-ranged networks of [SiO_4_]. The crystallinity of the silica films can be significantly improved when they are prepared on Mo(112) substrates at a monolayer thickness.^67−71^

#### 2.1.1 On Mo(112)

The Mo(112) surface is composed of atomic rows, which are closely packed and orientated in the [1̅1̅1] direction with separated furrows in the [1̅10] direction.^109^ Generally, the preparation of silica films includes deposition of approximately one ML Si onto an oxygen-precovered Mo(112) surface and subsequent high-temperature annealing in a vacuum. The crystallinity of the resulting films strongly depends on the annealing temperatures.^68^

##### 2.1.1.1 Crystalline 2D-Structure

**Figure 7:**
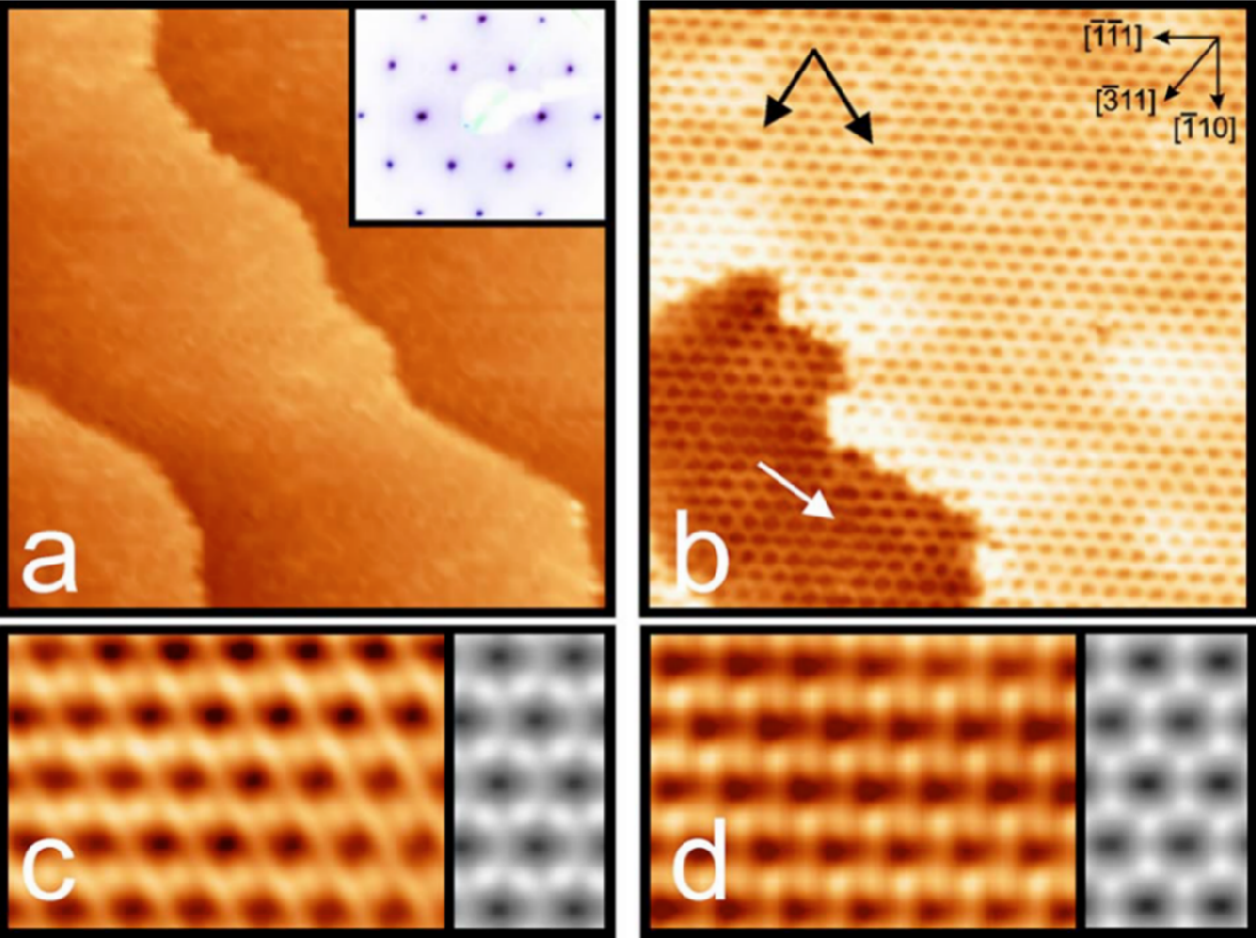
STM images of ML silica film on Mo(112). (a) Large-scale STM image (75 × 75 nm^2^, *U*_s_ = 2.0 V, *I* = 0.2 nA). The inset in panel a shows the corresponding LEED pattern with a *c*(2 × 2) structure. (b) Close-up STM image showing the line defects propagating in the [1̅10] direction of Mo(112) as indicated by the arrows (14 × 14 nm^2^, *U*_s_ = 1.3 V, *I* = 0.45 nA). Bias-dependent atomically resolved STM images: (c) *U*_s_ = 0.65 V, *I* = 0.8 nA; (d) *U*_s_ = 1.2 V, *I* = 0.35 nA. The right panels in parts c and d show the simulated STM images, which are based on the DFT-optimized structural model. The tunneling gap is set at 4 Å at 0.65 V in part c and 6 Å at 1.2 V in part d. Reproduced with permission from ref (84). Copyright 2006 American Physical Society.

After annealing at a high temperature of ∼1250 K, well-ordered silica films can be obtained as shown in Figure 7.^5,84,110^ Large-scale STM images reveal an atomically flat film with the absence of silica particles and patches (Figure 7a). The silica film has wide terraces with a step height of 1.2 Å, corresponding to a single atomic step of the Mo support. Close-up STM images show a honeycomb-like structure with a periodicity of ∼5.5 Å in the [111] direction and ∼5.2 Å in the [3̅11] direction, consistent with the *c*(2 × 2) pattern obtained in LEED. In addition, there are antiphase-domain-boundaries (APDB) as indicated by the black arrows, which are propagating in the [1̅10] direction. It should be noted that the honeycomb-like structures seen in atomically resolved STM images depend on the tunneling conditions (Figure 7c,d), indicating that several electronic states are involved in the tunneling process.

**Figure 8:**
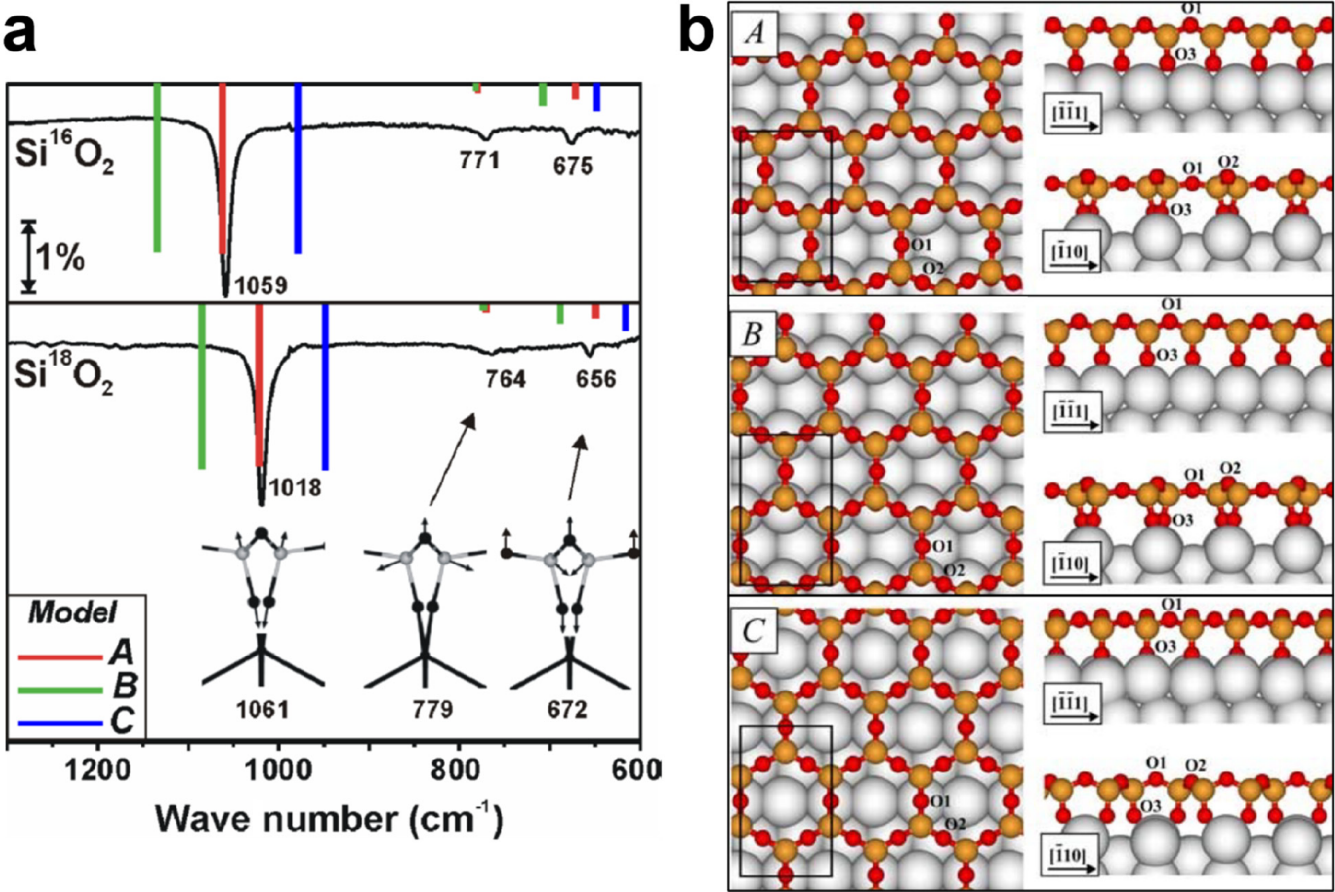
(a) IRAS of ML silica/Mo(112) films prepared with ^18^O_2_ and ^16^O_2_. The frequencies of the infrared active vibrations from three different models (A, B, and C) are calculated as indicated by the color bars, with the height proportional to the intensity normal to the surface. The insets in panel a show the vibrational modes (view along [1̅1̅1] direction) of the most stable structure A. (b) Schematic structures of these three models (A, B, and C). The rectangles indicate the Si_4_O_10_ surface unit cells. Reproduced with permission from ref (5). Copyright 2005 American Physical Society.

**Figure 9:**
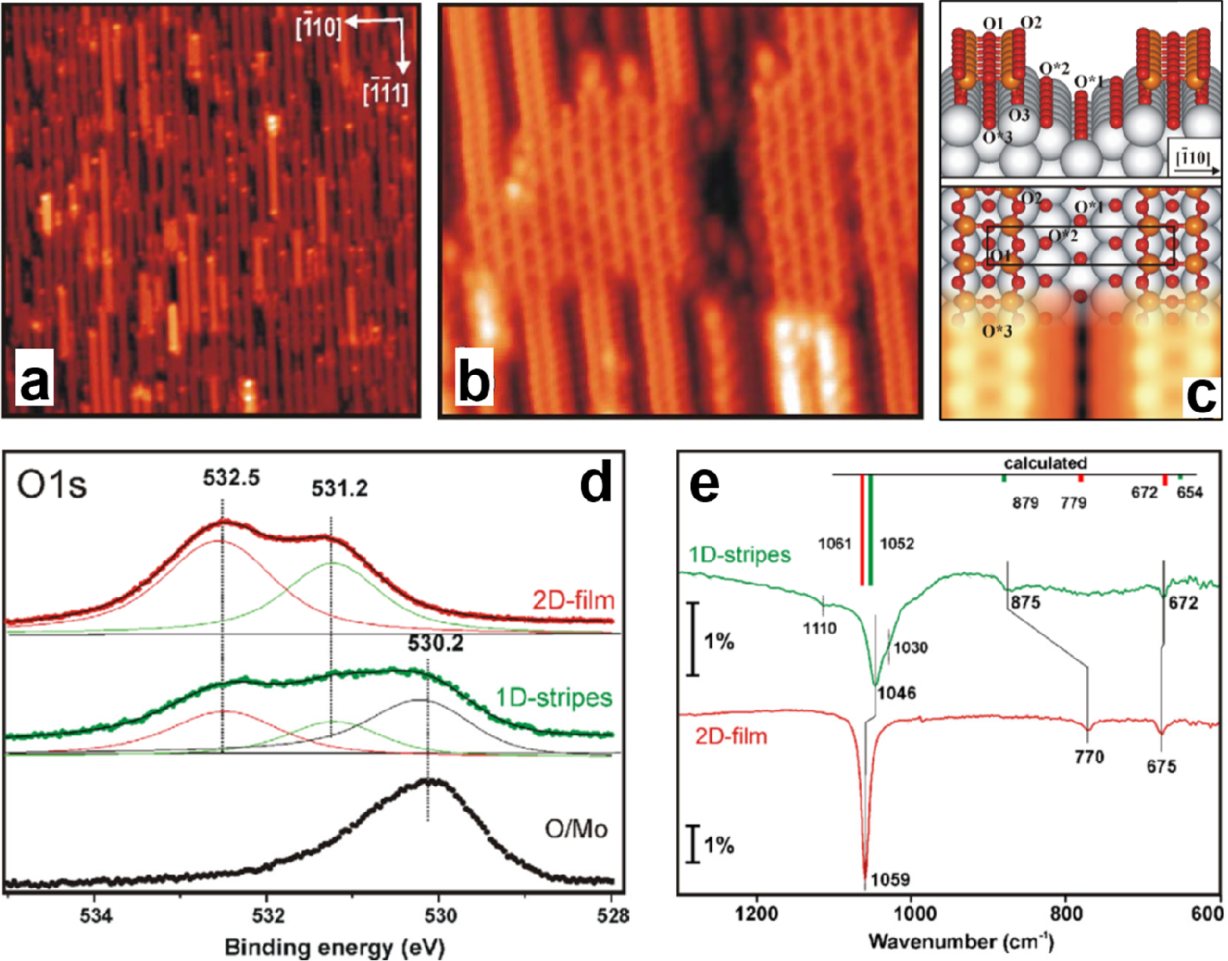
STM images of the 1D-silica stripes on Mo(112): (a) 50 × 50 nm^2^, *U*_s_ = 3.9 V, *I* = 0.2 nA; (b) 12.5 × 10.5 nm^2^, *U*_s_ = −0.4 V, *I* = 0.4 nA. (c) Schematic structure of the thermodynamically most stable 1D-silica stripes with a simulated STM image superimposed. The rectangle in panel c indicates the (1 × 3) surface unit cell. (d) XPS O 1s spectra for the 2D-silica film and 1D-silica stripe formed on Mo(112). (e) IRAS for the 2D-silica film and 1D-silica stripes formed on Mo(112). The calculated frequencies are also indicated by the color bars according to the structure model shown in panel c. Reproduced with permission from ref (86). Copyright 2006 Elsevier.

The most crucial information regarding the monolayer structure originates from the IRAS and X-ray photoelectron spectroscopy (XPS) results. Figure 8a presents the IRAS spectra for isotopically labeled silica thin films on Mo(112), showing a sharp and strong band at 1059 cm^–1^ with a full width at half-maximum (fwhm) of 12 cm^–1^, as well as two weak bands at 771 and 675 cm^–1^. It was found that the position and width of the main band sensitively depend on the quality of the film, e.g., the crystallinity and coverage of the film.^84^ These bands shifted to lower frequencies (1018, 764, and 656 cm^–1^) when ^18^O_2_ was used during film preparation. The XPS spectra of those films revealed only a single component contributing to the Si 2p peak at a the binding energy (BE) of 103.2 eV, indicating an oxidation state of Si^4+^. In contrast, the O 1s region exhibited two components centered at 532.5 and 531.2 eV (see Figure 9d).^84,110^ Both values are considerably higher than those observed for the MoO_*x*_ oxide layers.^111,112^ In these well-ordered silica films, the ratio of the peak areas for the O species at higher and lower BEs is found to be around 3:2. These results suggest a “2D-network model”^5,84,110^ instead of a “cluster model”,^62,113^ where a monolayer silica network consists of corner-sharing [SiO_4_] tetrahedra that are connected to the Mo(112) substrate via the Si–O–Mo linkages.^114^ This 2D-network structure was also evidenced by Seifert et al. based on ion beam triangulation (IBT)^115,116^ and fast atom diffraction (FAD),^117,118^ where the geometrical arrangement of the atoms in the topmost layer of the silica film can be straightforwardly derived.

Detailed atomic structures were clarified by DFT calculations.^5,119^ It has turned out that the most stable monolayer structure has a surface unit cell composition of Si_4_O_10_. In Figure 8b, three 2D-silica models are presented based on different adsorption sites of the interface O atom on Mo(112), i.e., bridge sites (model A), atop sites (model B), and pseudo-3-fold hollow sites (model C). While all models showed a *c*(2 × 2) structure with respect to Mo(112), model A was demonstrated to be thermodynamically most stable at all experimentally relevant oxygen pressures. Moreover, the calculated vibrational spectra for model A revealed the best agreement with the experimental results as shown in Figure 8a. The most intense mode at 1061 cm^–1^ originates from the Si–O–Mo asymmetric stretching, where the Si–O bond is pointing downward to the Mo substrate. The second mode at 779 cm^–1^ is caused by the Si–O–Si symmetric stretching mode coupled with Si–O–Si bending, and the third mode at 672 cm^–1^ results from a coupling of Si–O–Si bending with a small contribution of the Si–O–Si symmetric stretching mode. DFT calculations also reproduced the isotopic shifts in IRAS experiments for the silica film grown with ^18^O_2_, as well as the BE shifts (1.3 eV) in XPS experiments for the O 1s core-levels of the oxygen ion in Si–O–Si (532.5 eV) and Si–O–Mo (531.2 eV) (see Figure 9d), respectively. We note that the monolayer film has a stoichiometry of SiO_2,5_ with respect to the overall unit cell, similar to the structure of the silica film on SiC as discussed in Figure 4.

The 2D-network model described so far contains no additional surface oxygen atoms besides those involved in the Si–O–Mo linkages. Generally, the porous nature of the honeycomb-like structure of the silica film allows oxygen atoms to migrate through the rings and reside in different adsorption sites on the Mo(112) surface, which results in a so-called “O-rich” silica. The existence of these “O-rich” silica was examined by DFT and subsequently confirmed by IRAS and XPS.^87^ According to the calculations, a new phase denoted as ML silica/4O/Mo(112) is predicted to be the most stable structure. It contains four additional oxygen atoms adsorbed in a bridging position in the trenches of the Mo(112) surface along the [111] direction per surface unit cell. Adsorption of one, two, and three additional oxygen atoms per unit cell was found to be less stable. In comparison, subsurface oxidation of Mo(112) and partial decomposition of the silica film occur upon adsorption of more than four oxygen atoms.^87^ IRAS and XPS spectra revealed small but detectable changes for the silica films prepared by high-temperature annealing in either UHV (i.e., the pristine ML silica/Mo(112), “O-poor” silica) or 10^–6^ mbar O_2_ (i.e., “O-rich” silica). For example, the calculated Si–O–Mo asymmetric stretching mode shifts to 1046 cm^–1^, whereas the frequencies of the two other modes remain virtually unchanged, in agreement with the experimental IRAS results for the “O-rich” silica. The XPS results for the “O-rich” silica show an additional component in the O 1s region centered at 530.6 eV, which is attributed to the chemisorbed oxygen species on the Mo(112) surface. It is important to note that the postannealing of the “O-poor” silica in oxygen environment readily leads to the “O-rich” silica. However, it cannot be converted back to the “O-poor” silica due to the high binding energy of the chemisorbed oxygen species.

##### 2.1.1.2 Crystalline 1D-Structure

It became evident that the precise phases of the silica film may strongly depend on the film preparations. At submonolayer coverage, high-resolution STM revealed the formation of silica stripes with 0.5 nm in width along the [111] direction (Figure 9a,b).^86^ Each stripe consists of two rows of protrusions with a spacing of 2.8 and 4.5 Å in the [111] and [1̅10] directions, respectively, which are the same as the unit cell of the Mo(112) surface. Additionally, the distance between stripes (13.4 Å) is three times the Mo(112) lattice. It matches well the p(2 × 3)O–Mo(112) reconstructed surface, suggesting that the formation of stripes is associated with one oxygen-induced reconstruction of the Mo(112) surface.^109^ A model based on DFT calculations is shown in Figure 9c, illustrating that these silica stripes actually consist of paired rows of corner-sharing [SiO_4_] tetrahedra running along the [111] direction, while the Mo surface is reconstructed and possesses oxygen adsorbed in short-bridge sites (O*1), pseudo-3-fold hollow sites (O*2), and also the short-bridge sites underneath the silica stripes (O*3).

According to this model, the electronic and vibrational properties of the 1D-silica stripe are expected to be similar to the 2D-silica film. As shown in Figure 9d, the XPS O 1s spectra for 1D-silica stripes are essentially identical to those of the 2D-silica film except for the lower BE component (530.2 eV), which is related to the oxygen atoms chemisorbed on the Mo substrate (O*). The IRAS spectra for the 1D-silica stripe structure reveal a small red-shift of the main band (Si–O–Mo asymmetric stretching) from 1059 to 1046 cm^–1^, whereas the band at 770 cm^–1^ in 2D-silica film is largely blue-shifted to 875 cm^–1^. This significant shift is mainly due to the weaker couplings between the Si–O–Si symmetric stretching and the Si–O–Si bending in the 1D-silica stripes. DFT-calculated vibrational frequencies show good agreement with the experimental results as shown in Figure 9e. It should be mentioned that, similar to 2D-silica films, there are also few bands undetectable in the experiments due to the selection rules in IRAS,^120^ such as the Si–O–Si asymmetric stretching (985–1206 cm^–1^ for 1D-silica and 1008–1195 cm^–1^ for 2D-silica) and the out-of-phase Si–O–Mo asymmetric stretching (928 cm^–1^ for 1D-silica and 863–912 cm^–1^ for 2D-silica).^110^

With increasing coverage, these 1D-silica stripes with 4-membered rings coalesce and transform into 2D-silica films with 6-membered rings that fully cover the Mo(112) surface. During the film formation, line defects may occur if there is a half-lattice shift in the [111] direction between the adjacent silica stripes, which results in alternating 4- and 8-membered rings of [SiO_4_] tetrahedra as APDB (Figure 7b).

#### 2.1.2 On Ru(0001)

The Ru(0001) crystal symmetry is similar to the ML silica and is expected to further stabilize the 2D-silica phase without stress-relief defects (e.g., the misfit dislocations).^96^ The growth of silica thin films on Ru(0001) substrates was first developed in Freund’s group.^6^ After testing numerous preparation recipes, low-temperature deposition of Si in an oxygen environment was found to be the best recipe for obtaining high-quality silica thin films.^88^ Briefly, Si was deposited onto a 3O–(2 × 2)/Ru(0001) surface at ∼100 K in ∼10^–7^ mbar O_2_ and then annealed at ∼1200 K in ∼10^–6^ mbar O_2_ for a few minutes. The presence of the 3O–(2 × 2)/Ru(0001) surface may prevent the intermixing of Si and Ru as well as provide a template effect for silica growth with a honeycomb-like structure. A low substrate temperature during the Si deposition can suppress the diffusivity of Si atoms on the surface, thereby favoring the formation of 2D structures prior to the final oxidation at high temperatures.

##### 2.1.2.1 Crystalline Structures

**Figure 10:**
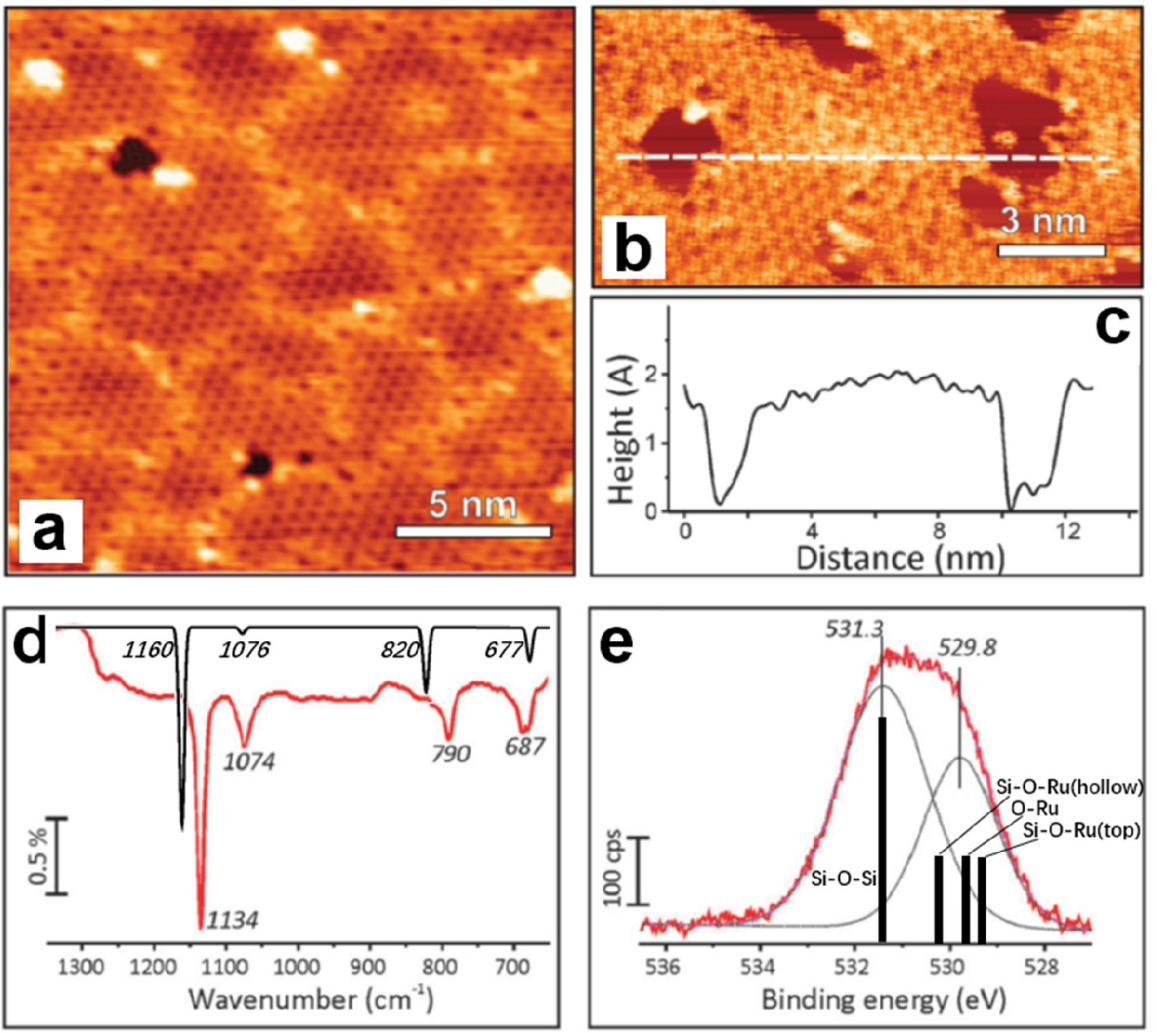
STM images of ML silica film on Ru(0001): (a) *U*_s_ = 2.0 V, *I* = 0.1 nA; (b) *U*_s_ = 1.2 V, *I* = 0.1 nA. (c) Height profile measured along the line indicated in panel b. (d) IRAS and (e) XPS O 1s of ML silica film on Ru(0001). The black spectrum in panel d and the black bars in panel e are DFT-calculated frequencies and relative BE shifts of the O 1s core-levels. Reproduced with permission from ref (88). Copyright 2012 Royal Society of Chemistry.

Deposition of 0.5 ML (with respect to the Ru(0001) surface) Si would result in an atomically flat silica film after high-temperature annealing in oxygen as shown in Figure 10a.^88^ The Ru substrate was almost entirely covered by the silica film, which comes with small pits and holes decorated by nanoparticles. The as-prepared silica film has multiple domains, all containing a honeycomb-like structure with a 5.4 Å periodicity. The domains are shifted with respect to each other by half a lattice constant, thus producing a network of domain boundaries. The silica film is ∼1.4 Å in apparent height with respect to the underlying Ru substrate, suggesting an ML structure. The corresponding IRAS spectrum in Figure 10d shows a dominant band at 1134 cm^–1^ and weaker bands at 1074, 790, and 687 cm^–1^, similar to those observed for the silica/Mo(112) film (see Figure 8a). As further clarified by DFT calculations, these bands are assigned to the asymmetric stretching of the Si–O–Ru linkages (1134 cm^–1^), the combinations of Si–O–Si symmetric stretching (1074 cm^–1^), and the combinations of Si–O–Si bending and Si–O–Ru asymmetric stretching (790 and 687 cm^–1^), respectively.^88^ XPS spectra of the film are also similar to those found for the silica/Mo(112) film, where there is only one state in the Si 2p region (102.3 eV) and two components in the O 1s region (531.3 and 529.8 eV with a peak area ratio of ∼3:2).

After combining the above experimental and theoretical results, a structure model for silica on Ru(0001) was proposed. Specifically, the film is composed of a honeycomb-shaped network of tetrahedral Si–O linkages with a lattice constant of 5.4 Å, in which every Si forms three bridging Si–O–Si bonds and one Si–O–Ru bond.

With the help of synchrotron-based high-resolution XPS spectra, detailed chemical binding configurations of the silica ML on Ru(0001) have been established.^121^ Kremer et al. experimentally demonstrated that there are two kinds of Si–O–Ru linkages involving two chemically inequivalent Ru atoms, i.e., Si–O–Ru(top) and Si–O–Ru(hollow), therefore suggesting the existence of two sublattices in the honeycomb-like structure of the ML silica/Ru(0001).^88,121^

**Figure 11:**
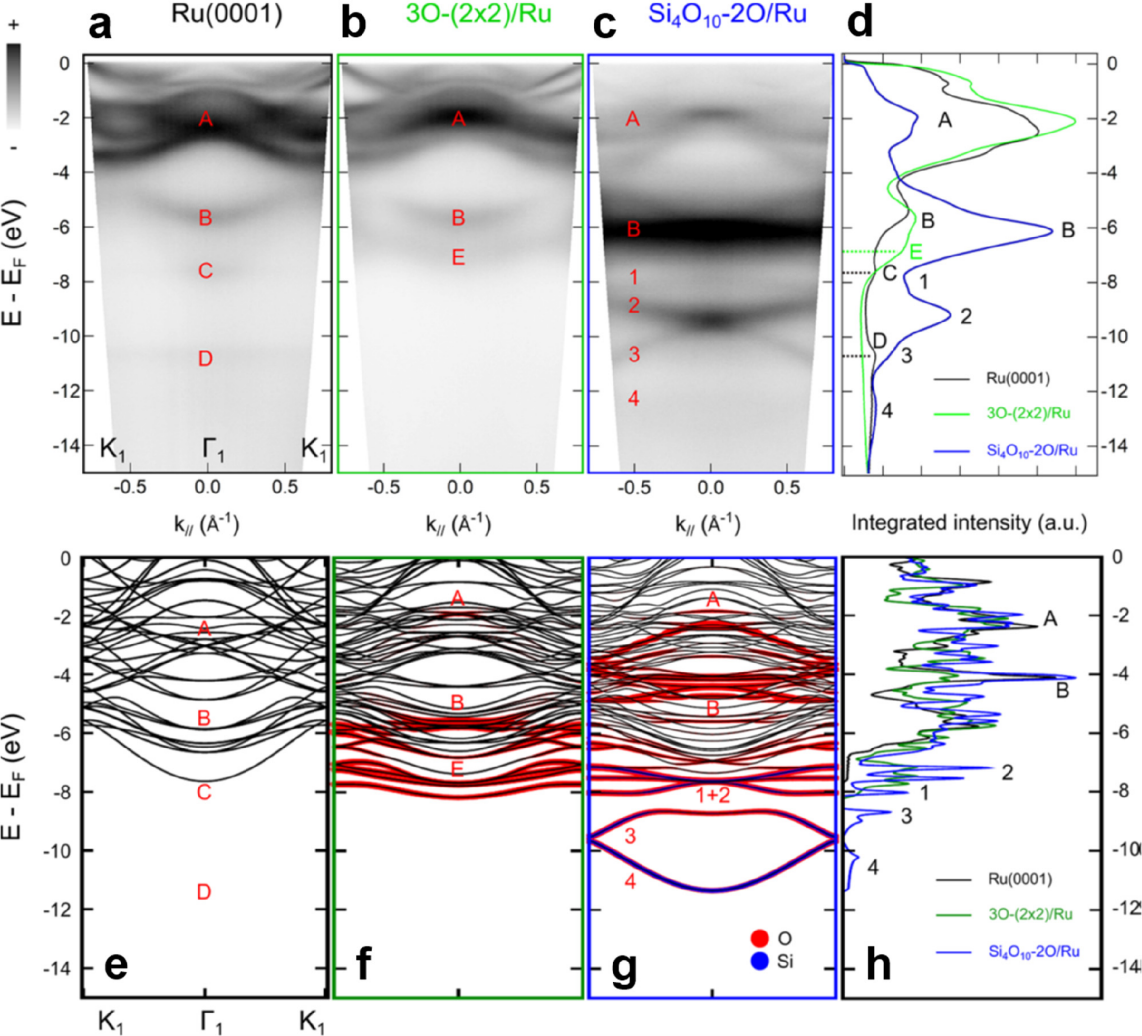
(a–c) ARPES spectra (*hυ* = 40 eV, with υ linear horizontal polarization) along the K_1_–Γ_1_–K_1_ high-symmetry line for bare Ru(0001), 3O–(2 × 2)/Ru(0001), and ML silica//Ru(0001), respectively. (d) Corresponding integrated intensities along k_∥_. (e–g) DFT-calculated band structures for bare Ru(0001), 3O–(2 × 2)/Ru(0001), and ML silica/Ru(0001), respectively. Black, red, and blue represent the Ru, O, and Si characters of the bands. (h) Calculated full density of states (DOS). Reproduced with permission from ref (121). Copyright 2019 American Chemical Society.

The electronic band structure of the ML silica/Ru(0001) system was also explored as shown in Figure 11 by angle-resolved photoemission spectroscopy (ARPES). Four bands (labeled 1–4 in Figure 11c) are observed in the energy range between −8 and −14 eV below the Fermi level, in addition to the features from the Ru(0001) substrate and surface chemisorbed oxygen atoms. Band 1 is almost flat and is located at about −8 eV. Bands 2 and 3 cross at −9.5 eV and disperse downward and upward around the Γ point, respectively. Band 4 disperses downward with a minimum energy of −13 eV at the Γ point. Due to matrix element effects,^122^ band 4 is exclusively observed in the second Brillouin zone (BZ). These dispersive electronic states are generally reproduced by DFT calculations (Figure 11g). However, it should be noted that the DFT calculations cannot reproduce the relative positions of bands 1 and 2 as well as the crossing at the Γ point between the bands 2 and 3.

The increase of the spectral weight associated with band B (Figure 11d) in silica/Ru(0001) is attributed to the O 2p states involving O atoms in silica (Si–O–Si), which has also been observed in amorphous and crystalline ML silica on Mo(112).^61,110,123^ Nevertheless, the O atoms in Si–O–Ru(top) and Si–O–Ru(hollow) might also have non-negligible contributions to the total spectral weight. Based on the polarization-dependent ARPES results (i.e., linear vertical polarization and linear horizontal polarization), the new bands 1 and 2 most likely originate from the hybridization of p_*z*_ orbitals from the O and Si atoms in Si–O–Ru linkages, specifically, the out-of-plane covalent Si–O–Ru bonds,^121^ while bands 3 and 4 were concluded to have in-plane characters and emerged from the hybridization in Si–O–Si bonds. Therefore, the ML silica/Ru(0001) is characterized by at least four inequivalent dispersive bands.

#### 2.1.3 Defect Structures

As discussed above (Figure 10a), the ML silica prepared on Ru(0001) will have multiple domains. Mathur et al. further investigated the origin of these domain boundaries.^124^ As revealed by high-resolution STM and reflection high-energy electron diffraction (RHEED), the ML silica is found to coexist with a (2 × 2) reconstruction of oxygen atoms inside the rings of the silica, which is similar to the case of the “O-rich” silica on Mo(112).^87,124^ This coexistence signals a displacive transformation from 3O–(2 × 2)/Ru(0001) to 2D-silica, which is degenerate and yields antiphase boundaries that are exclusively orientated along armchair directions and consist of pairs of 7- and 5-membered rings. Such a transformation is the leading source for the domain boundary defects in ML silica/Ru(0001). It is noteworthy that the antiphase-domain-boundaries (APDB) in ML silica/Mo(112) consist of alternating 8- and 4-membered rings (Figure 7b).

**Figure 12:**
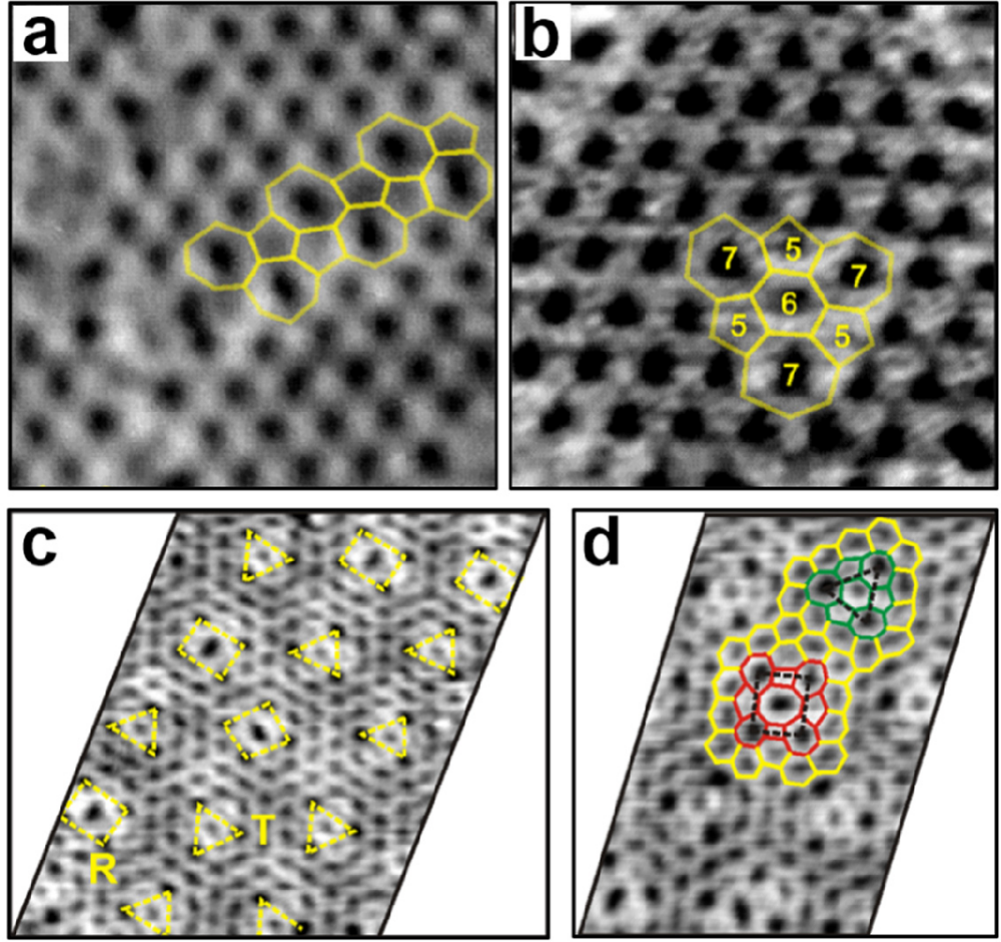
STM images show the domain boundary defects (a) and the “blister defects” (b) of ML silica film on Ru(0001). (c) STM image with arrays of blister defects, marked as R and T. (d) STM image superimposed with the polygonal representation of the defects. The R-defect (in red) and T-defect (in green) are surrounded by hexagons (in yellow). (a–d) *U*_s_ = 1.2 V, *I* = 0.15 nA. Reproduced with permission from ref (126). Copyright 2013 American Chemical Society.

**Figure 13:**
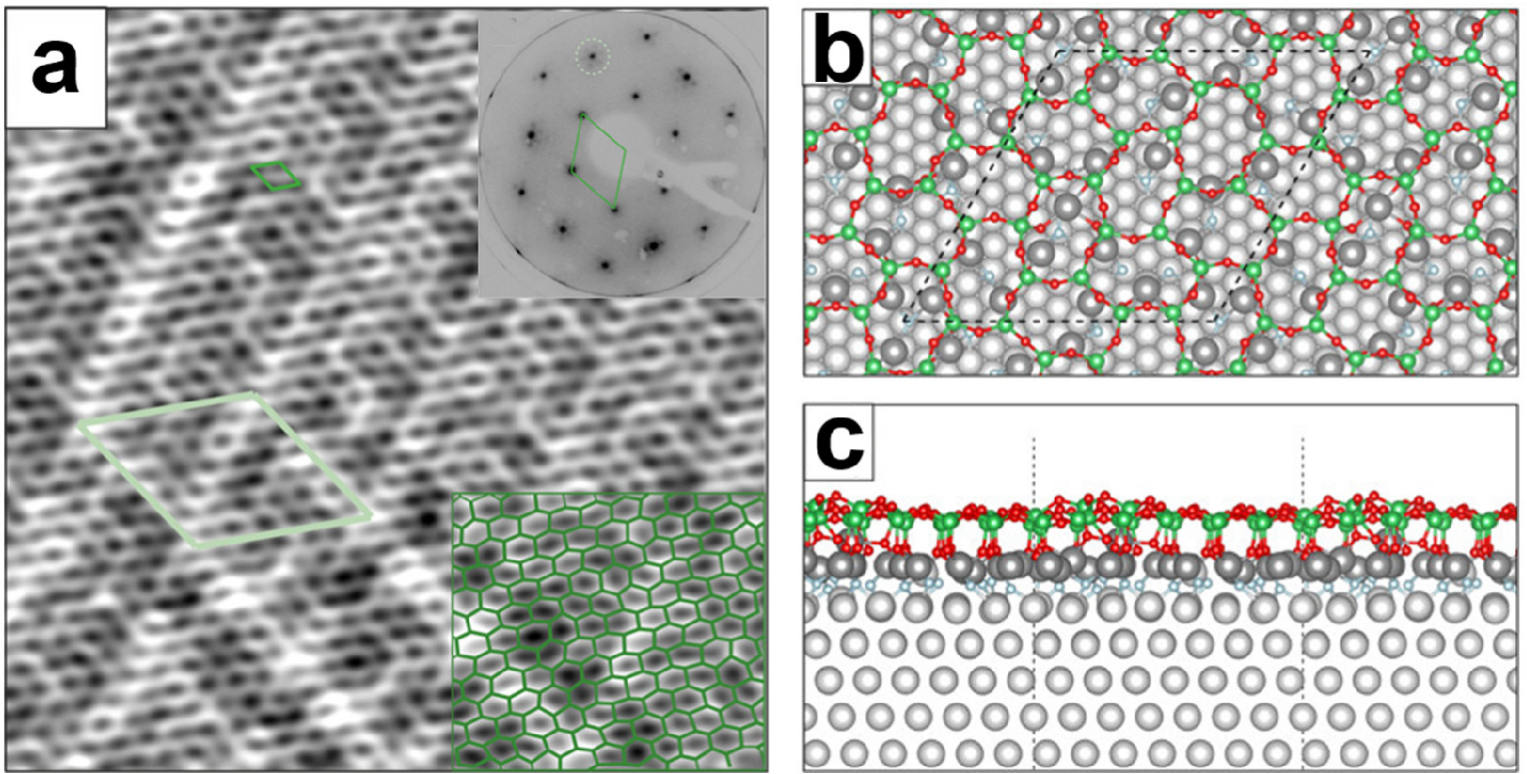
(a) STM image of the ML silica on Cu(111) (14 × 14 nm^2^, *U*_s_ = 0.8 V, *I* = 0.02 nA). Marked unit cells: silica lattice (green), moiré from the superposition of silica on the Cu_2_O-like layer (light green). Insets: (top right) LEED pattern for the silica film (at 78 eV). The green rhombus represents the silica unit cell, and the satellite spots indicated by a light green circle correspond to moiré structures. (bottom right) The superimposed green rings present solely 6-membered rings and a variety of sizes and distortions. (b, c) Top and side views of silica ML on Cu_*x*_O/Cu(111). Reproduced with permission from ref (106). Copyright 2020 American Chemical Society.

Besides the domain boundary defects (Figure 12a), the “blister defects”, consisting of a hexagon surrounded by three 7- and 5-membered rings, are also found in ML silica/Ru(0001) (Figure 12b). Such “blister defects” were previously only predicted for graphene by DFT calculations.^125^ The STM images in Figure 13c,d were obtained after annealing the ML silica/Ru(0001) in UHV at higher temperatures. It shows that the film is no longer manifested by hexagons exclusively. Instead, there are arrays of structural defects as marked with triangles (T) and rectangles (R) embedded into the hexagonal network. The T-defects are, in essence, the blister defects with 3-fold symmetry, while the R-defects constitute an octagon surrounded by two tetragons, two pentagons, and four heptagons, thus exhibiting the 2-fold symmetry. Both T- and R-defects were equally and randomly present in the entire film. However, the T-defects seem to be energetically more favorable than the R-defects since they start to dominate the film after prolonged annealing. Moreover, two isomorphs, which are rotated by 60° with respect to each other, were found for both T- and R-defects.

#### 2.1.4 On Oxidized Cu(111)

In ML silica on Mo(112) and Ru(0001), the [SiO_4_] tetrahedrons are directly bound to the metal support via Si–O–metal linkages. Recently, Navarro et al. reported a new kind of interaction for ML silica on a Cu(111) surface.^106^ By depositing appropriate amounts of silicon onto an oxidized Cu(111) surface (e.g., so-called “29” structures and “44” structures^127,128^) and subsequently annealing in ∼10^–6^ mbar O_2_ at ∼973 K, a well-ordered silica film consisting of 6-membered rings with an average periodicity of ∼5 Å can be obtained (Figure 13a). This lattice constant agrees with the expected periodicity for ML silica as discussed above for silica on Mo(112) and Ru(0001).^6,88^ Generally, the lattice shows various distortions, indicating a certain flexibility of the bonds. In contrast to the ML silica on Mo(112) and Ru(0001), no domain boundaries were observed across the entire surface as inferred from the large-scale STM images.

DFT calculations then demonstrated that the oxidized copper surface plays an essential role in preserving the hexagonal symmetry of the silica film. As compared to the ML silica on Cu(111), the distortions of the rings appear more pronounced for the ML silica on oxidized Cu(111). In Figure 13b,c, the oxygen atoms from the silica are segregated down toward the support. In contrast, the copper atoms from interface CuO_*x*_ are vertically displaced toward the silica films and horizontally displaced to saturate the dangling bonds of the silica. Such distorted 6-membered rings are similar to those predicted for reconstructed α-quartz (0001) surfaces.^129^ The relaxation of the silica structure through the distortion of the hexagonal rings without changing the lattice symmetry could be a common mechanism in crystalline silica systems. It is essential to point out that an actual crystallographic structure determination is still not achieved. Further efforts are needed to unravel the complexity of the silica film on an oxidized Cu(111) surface.

### 2.2 Bilayer Structures

It has been shown above that a crystalline silica monolayer consists of corner-sharing [SiO_4_] tetrahedra and may be grown on metal surfaces with a SiO_2.5_ stoichiometry. By increasing the amount of deposited Si, stoichiometric SiO_2_ films with a thickness of only ∼0.6–0.9 nm are obtained.^130^ However, these films turned out to be amorphous without long-range ordering. The lack of layer-by-layer growth was attributed to the strong interfacial Si–O–Mo bonds and the saturated oxygen termination of the monolayer. The first successful attempt to achieve crystalline silica film with a SiO_2_ stoichiometry was realized on a Ru(0001) substrate.^6^ Both experimental and theoretical results provide convincing evidence for the formation of crystalline SiO_2_ films with a bilayer structure that weakly binds to the metal support.

#### 2.2.1 On Ru(0001)

The preparation condition for BL silica on Ru(0001) is similar to that of ML silica on Ru(0001), except that the amount of deposited silicon has to be increased. In this section, detailed structures of BL silica/Ru(0001) systems will be discussed because of their importance as model systems for surface science studies of silica-based catalysts.^131^

##### 2.2.1.1 Crystalline Structures

**Figure 14:**
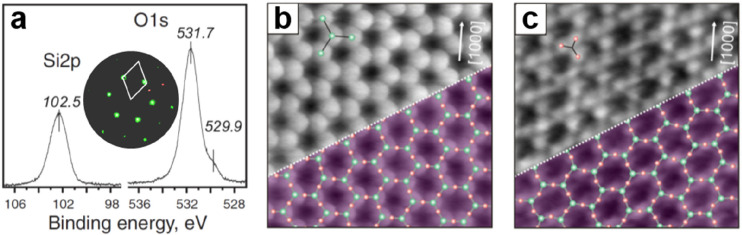
(a) XPS of Si 2p and O 1s core-levels for the BL silica on Ru(0001). The inset shows the corresponding LEED pattern (at 60 eV) with the indicated (2 × 2)–Ru(0001) unit cell. Atomically resolved STM images of the crystalline silica BL: 14 × 14 nm^2^, (b) *U*_s_ = 3.0 V, *I* = 0.1 nA; (c) *U*_s_ = 0.1 V, *I* = 0.1 nA. The crystallographic axis of the Ru(0001) substrate is indicated by an arrow. Red and green balls represent the O and Si atoms, respectively. Reproduced with permission from refs (6 and 90). Copyright 2010 American Physical Society, Copyright 2012 American Chemical Society.

After annealing in ∼10^–6^ mbar O_2_ at ∼1200 K, the XPS spectra reveal one component in Si 2p (102.5 eV) and two components in O 1s (531.7 and 529.9 eV) (Figure 14a), which are similar to that of ML silica on Ru(0001).^88^ However, the integral amount of Si was estimated to be approximately twice that of the ML silica/Ru(0001), and the intensity ratio between the O 1s main and shoulder peaks was estimated to be ∼12:1 instead of ∼3:2 in ML silica/Ru(0001). In addition, the intensity of the shoulder peak largely depends on the film preparations. It can be considerably reduced upon annealing the film in UHV at 1000 K. Therefore, the shoulder peak can be assigned to the O species chemisorbed on Ru substrate.

**Figure 15:**
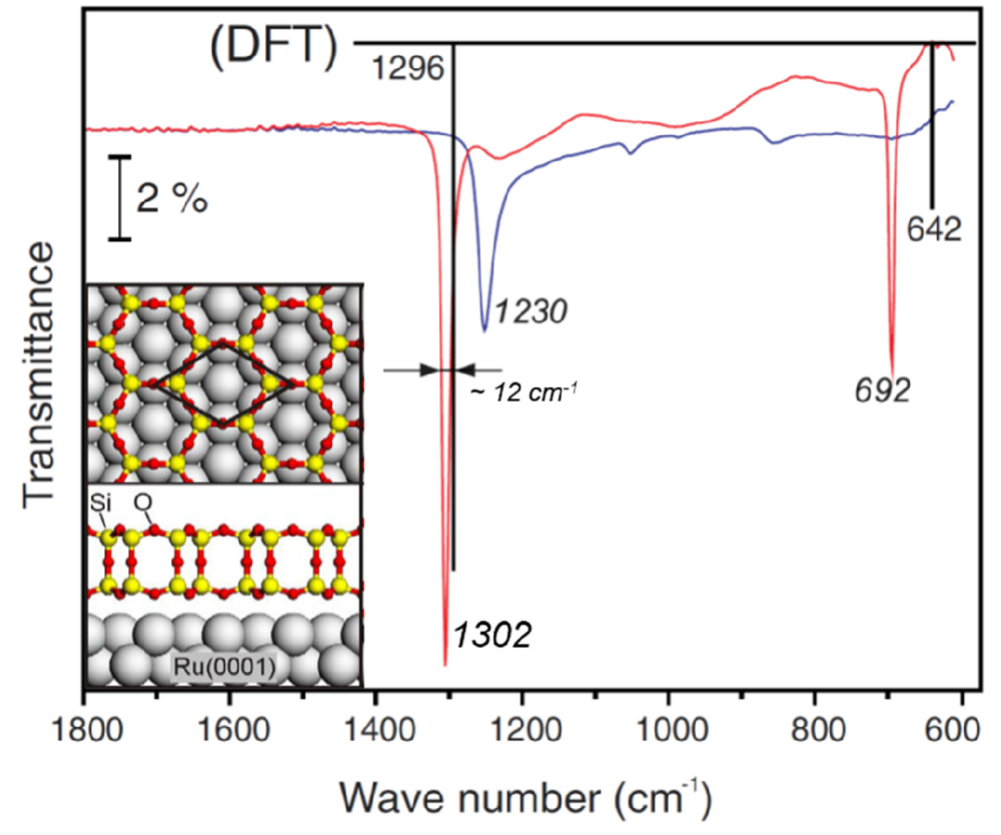
IRAS of BL silica film on Ru(0001) as deposited at 630 K (blue spectrum) and after crystallization at 1140 K (red spectrum). The black bars show the position and relative intensity of the DFT-calculated bands for the bilayer structure presented in the inset. Reproduced with permission from ref (6). Copyright 2010 American Physical Society.

Atomically resolved STM images reveal the hexagonal structure with a 5.5 Å periodicity. As shown in Figure 14b,c, the atomic structures of the topmost Si and O atoms are resolved with different imaging biases. Taking into account the (2 × 2) LEED pattern, as well as the Si 2p and O 1s XPS core-level spectra, it is deduced that the film is composed of two layers of corner-sharing [SiO_4_] tetrahedra bonded together by an oxygen linkage (Figure 15). This bilayer structure was previously considered as a possible model for the silica film on Mo(112) on the basis of calculations,^84^ but it has been discarded because of the disagreement with the experimental results.^110^

IRAS spectra provide clear evidence for this bilayer model on Ru(0001) as shown in Figure 15. The as-deposited film was dominated by a band centered at ∼1230 cm^–1^, which was previously observed on thick amorphous silica films grown on metals and assigned to the asymmetric longitudinal optical vibration mode.^52^ Subsequent high-temperature annealing results in two sharp bands at 1302 and 692 cm^–1^. The band at 1302 cm^–1^, which has never been observed previously on various silica films, is about 170 cm^–1^ higher than the Si–O–Ru asymmetric stretching on ML silica/Ru(0001).^5,47,53,132^ In films prepared with ^18^O_2_, these two bands red-shift to 1247 and 664 cm^–1^, respectively, in good agreement with the values predicted based on the reduced masses of a Si–O–Si oscillator. Combined with the DFT studies, the most intense band at ∼1302 cm^–1^ is assigned to the asymmetric Si–O–Si stretching normal to the surface, while the second band at ∼692 cm^–1^ is assigned to the symmetric Si–O–Si stretching nearly parallel to the surface. Since the silica BL structure may also be prepared via two “deposition-oxidation” steps (i.e., deposit another Si layer onto the prepared ML silica/Ru and then oxidize it in O_2_),^88^ it is necessary, in order to transform ML silica into a BL silica structure, to break the Si–O–Ru linkage and create the Si–O–Si linkage. Apparently, such a process is thermodynamically unfavorable for the Mo(112) substrate. As a result, the formation of well-ordered bilayer structures on Mo(112) has never been observed.

The BL silica film has no dangling bonds on either side and only weakly interacts with the Ru substrate. The calculated adhesion energy of the BL silica sheet to the Ru(0001) support was only about 3.1 kJ mol^–1^ Å^–2^, with the main contribution coming from the dispersion term.^6^ In analogy with the ML silica/Mo(112) system, the BL silica/Ru(0001) system also exists in the “O-poor” and “O-rich” configurations, which depends on the amount of chemisorbed O atoms on the Ru(0001) surface. For the case of an “O-rich” film with 0.25 ML chemisorbed interfacial O atoms [i.e., (2 × 2)O–Ru(0001)], the BL silica will adhere to the Ru(0001) with a position where the O atoms in the bottom layer of the BL silica are located above the hcp hollow sites of Ru(0001). The corresponding adhesion energy for this structure is reduced to 2.4 kJ mol^–1^ Å^–2^. Such interfacial properties regarding the tunable chemisorbed oxygen atoms underneath the silica films will be systematically discussed in section 3.3. In principle, the variation of oxygen concentration on the Ru surface opens the possibility of tuning the electronic properties of silica/metal systems without altering the structures of a silica overlayer itself.^133,134^

**Figure 16:**
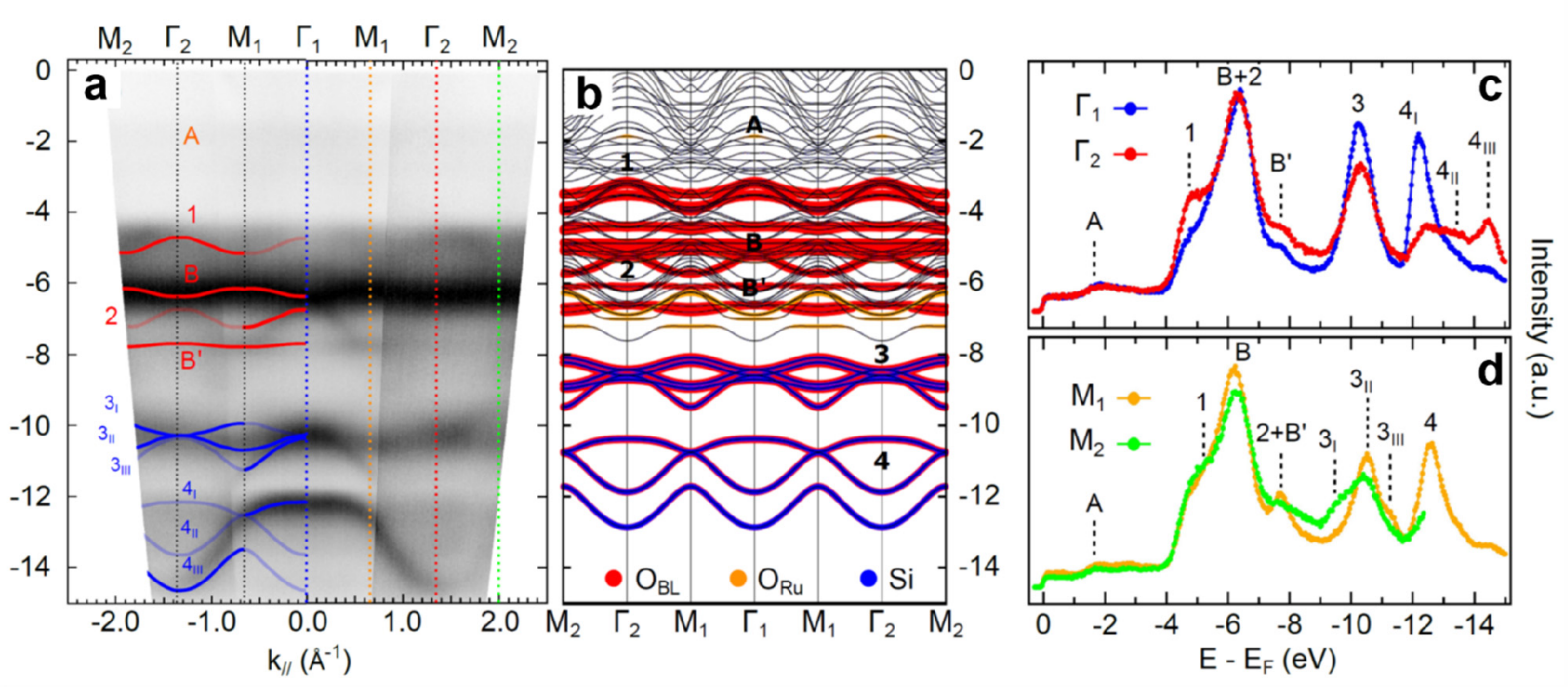
Electronic band structure of crystalline BL silica grown on Ru(0001). (a) ARPES spectra (He II, *hυ* = 40.8 eV) along the M_2_–Γ_2_–M_1_–Γ_1_–M_1_–Γ_2_–M_2_ high-symmetry direction. Red and blue curves represent the main DFT-calculated bands which are shifted by −1.7 eV. Thin and thick curves indicate the weak and strong experimental spectral weights, respectively. (b) DFT-calculated electronic band structures. Black, red, orange, and blue correspond to Ru, O in the BL silica, O chemisorbed on Ru, and Si character of the electronic bands, respectively. (c, d) The energy distribution curves were taken at the Γ points and M points of the BZ, respectively. The Γ points and M points are indicated by the colored vertical dashed lines in panel a. Reproduced with permission from ref (138). Copyright 2021 IOP Publishing Ltd.

Similar to ML silica/Ru(0001), the BL silica grown on Ru(0001) also exhibits a series of well-defined semiflat and dispersing electronic bands. As shown in Figure 16a, the band structures are mapped by ARPES along the M_1_–Γ_1_–M_1_ direction of the BZ. The band A centered around −2 eV stems from the chemisorbed O atoms on Ru(0001). The nondispersive bands B and B′ located around −6 and −7.8 eV are ascribed to the nonbonding O states. Beyond the relative intensity differences in some of the bands, four groups of dispersing bands are specifically observed in BL silica, i.e., band 1 (−4.9 to −5.2 eV), 2 (−6.2 to −7.8 eV), band 3 (−9.8 to −11.2 eV), and band 4 (−12.1 to −14.5 eV). Both bands 3 and 4 consist of three dispersive bands (3_I_, 3_II_, and 3_III_; and 4_I_, 4_II_, and 4_III_). Bands 3 are degenerate at the Γ point, resulting in a strong peak at −10.2 eV in the corresponding energy distribution curves (Figure 16c,d), while bands 4 are degenerate at the M point.

Figure 16b displays the electronic band structure according to DFT calculations, which turns out to be very close to the ARPES data except for a globally upward shift of the bands by about 1.7 eV. According to the DFT results, band 1 has both in-plane and out-of-plane character and is dominated by contributions from the s + p_*x*_ + p_*y*_ and p_*z*_ orbitals of the O atoms in Si–O–Si (bonds within the two outer planes and linkages between the two outer planes), while band 2 has only in-plane character and is dominated by the s + p_*x*_ + p_*y*_ orbitals of the O and Si atoms in Si–O–Si (bonds within the two outer planes). Similarly, the bands “3_I_ + 3_III_” and “4_II_ + 4_III_” involve in-plane O and Si orbitals (Si–O–Si bonds within the two outer planes), while bands “3_II_” and “4_I_” originate from out-of-plane O orbitals (Si–O–Si linkages between the two outer planes).

The valence band maximum (VBM) of the as-grown BL silica is located at the Γ point and around −4 eV below the Fermi level, as revealed by the ARPES data. In the DFT calculations, the VBM shifts up to −3.25 eV while the conduction band minimum (CBM) is formed at +2.47 eV above the Fermi level, resulting in a direct band gap of 5.72 eV. Moreover, the DFT shows that several linearly dispersing bands cross at the K point, which has been previously observed in the ML silica films^121^ and other 2D materials.^135,136^ It should be noted that freestanding BL silica has a similar electronic band structure, suggesting once more a weak interaction with the Ru(0001) substrate. Therefore, advanced DFT calculations were performed for the freestanding BL silica with the HSE06 exchange-correlation hybrid functions in order to accurately reproduce the experimental data. A band gap of 7.36 eV was then derived, in agreement with previous hybrid calculations (7.2 eV).^137^

##### 2.2.1.2 Vitreous Structures

**Figure 17:**
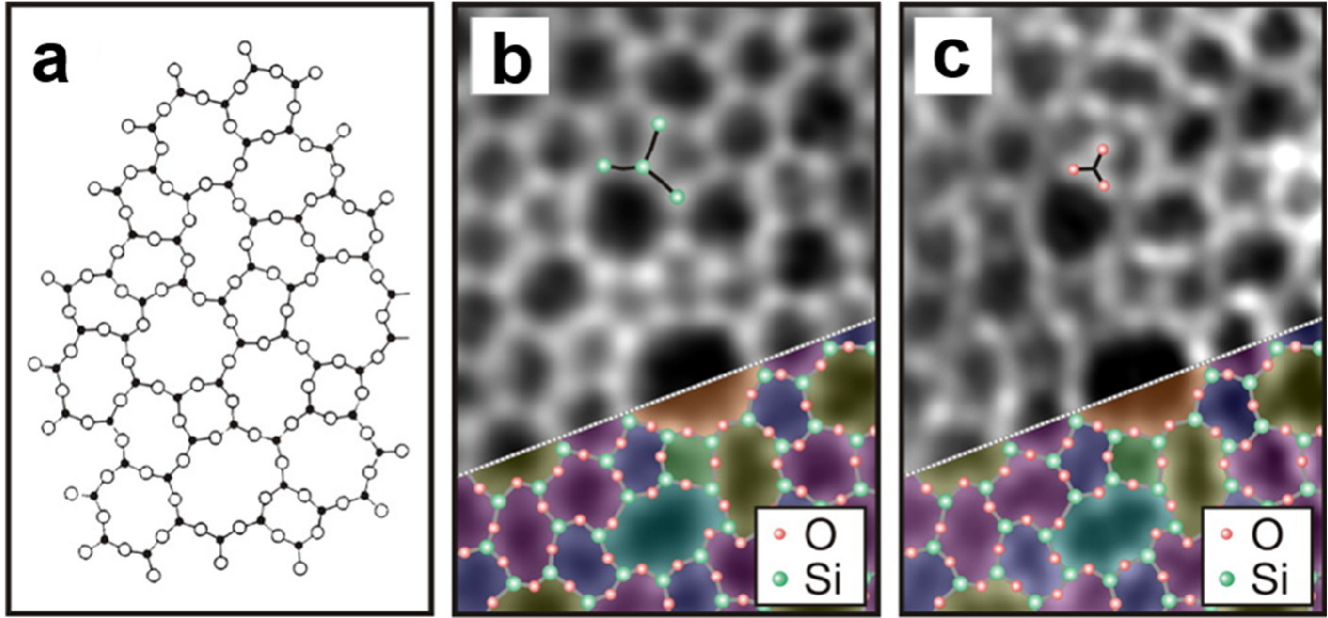
(a) Zachariasen’s structure model of silica vitreous networks (black dots, Si; white circles, O). Atomically resolved (b) nc-AFM and (c) STM images of the same vitreous silica bilayer (2.7 × 3.9 nm^2^). Imaging parameters: (b) oscillation amplitude = 0.27 nm, grayscale from −1.0 Hz (dark) to +0.6 Hz (bright); (c) *U*_s_ = 0.1 V, grayscale from 50 pA (dark) to 500 pA (bright). Panel b reveals the atomic structure of the Si atoms, whereas panel c reveals the arrangement of the O atoms. An atomic model of the topmost layer of the silica is superimposed onto the lower right part of the images (green balls, Si; red balls, O). Reproduced with permission from ref (90). Copyright 2012 American Chemical Society.

During the preparation of the BL silica, the cooling rate after high-temperature annealing is one of the critical parameters for determining the crystallinity of the films. Generally, fast cooling will result in the formation of amorphous structures.^139,140^ A diffraction ring in addition to the (2 × 2) pattern was observed in LEED for those BL silica films prepared under a relatively fast cooling (∼5 K/s),^88^ indicating a vitreous structure with randomly oriented crystallites. Although Zachariasen proposed that vitreous silica consists of a three-dimensional random network of corner-sharing [SiO_4_] tetrahedra in 1932,^36^ its atomic structure has never been verified microscopically until the STM work by Lichtenstein et al. in 2012.^91,92^Figure 17a shows on the left Zachariasen’s schematic proposal from 1932.^36^ This schematic may be compared with the STM and noncontact (nc) atomic force microscopy (AFM) images of the same area of the BL silica film. While nc-AFM provides an oxygen-dominated contrast (Figure 17b), STM (Figure 17c) shows the silicon atoms. By combining the two data sets, one obtains the full structure information including the chemical identification of the constituents of this network (see the bottom part of Figure 17b,c). The very close agreement of the measured with the suggested vitreous network structure is striking.

**Figure 18:**
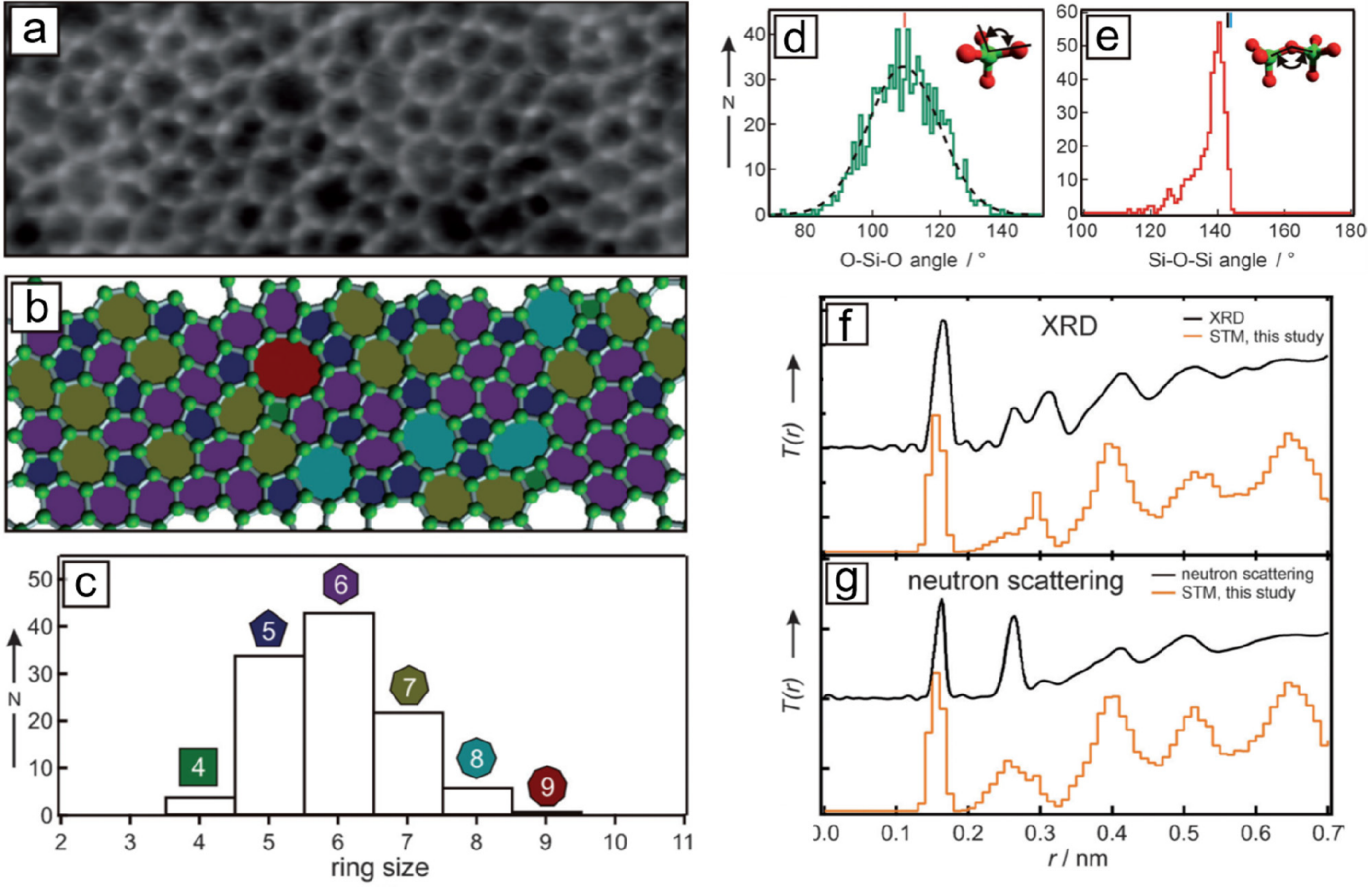
(a) STM image of a vitreous silica bilayer on Ru(0001) (8 × 3 nm^2^, *U*_s_ = 0.1 V, *I* = 0.1 nA). (b) The same image with a superimposed atomic model. Only Si atoms are identified (green balls). (c) Histogram of the differently sized silica rings. (d, e) Histograms of the O–Si–O angle and the Si–O–Si angle, respectively. The red bar in panel d indicates the regular tetrahedral angle of 119.5°. The black and blue bars in panel e indicate average values from a ^29^Si MAS NMR study^141^ and an XRD study^41^ on vitreous silica. (f, g) PCF obtained by STM compared to the PCF obtained by XRD measurements^41^ and neutron scattering measurements^148^ on vitreous silica, respectively. Reproduced with permission from ref (92). Copyright 2012 WILEY-VCH Verlag GmbH & Co. KGaA, Weinheim.

Figure 18a again shows an STM image of the areas of the amorphous silica bilayer on Ru(0001). Similarly, the polygonal networks can be clearly recognized, where the protrusions are arranged in triangles and can be assigned to O atoms in the tetrahedral [SiO_4_] building block. Figure 18b visualizes these polygons with different sizes *N* (number of members forming the ring). A histogram (based on a larger imaging area) in Figure 18c reveals that the ring size varies between *N* = 4 and 9, with a maximum at *N* = 6 corresponding to the crystalline structures. The intratetrahedral O–Si–O angle showed a symmetric distribution with an average of 110° (±10°), which matches well with the 109.5° angle in a regular tetrahedron (Figure 18d). The histogram of the Si–O–Si angle in Figure 18e reveals a peak at 141° and an edge at 145°. This peak angle of 141° is in agreement with the average angles obtained by ^29^Si magic-angle spinning NMR spectroscopy^141^ and the X-ray diffraction (XRD)^41^ for bulk vitreous silica, while the sharp edge at 145° manifests the flat and 2D character of the BL silica film.^142^

The consideration of the concept of a pair correlation function (PCF) is another practical way to characterize the atomic order in BL silica. From the experimentally derived structural model of the bilayer film, PCFs can be determined by using X-ray and neutron scattering factors of Si and O.^143,144^ To account for the 2D nature of the film, the data were additionally normalized by *r*^–1^. The peak positions and their relative intensities are shown in Figure 18f,g, which show good agreement with the PCF obtained from XRD and neutron scattering studies on 3D silica. The agreement is surprisingly good, given that 3D systems are compared with 2D systems.

In order to estimate the energy needed to form a vitreous structure by starting from a crystalline state, we consider the formation of defects. Such a defect structure can actually be obtained by rotating one (SiO_2_)_4_ unit to form two 5- and two 7-membered rings out of four 6-membered rings.^145^ This defect is called a Stone–Wales defect (5–7–5–7 rings),^81^ which will be discussed in detail in section 2.2.1.5 and which has been suggested in connection with graphene amorphization. Similar ring structures have also been observed in other oxide film systems.^146^ It should be noted that XPS, IRAS, and high-resolution electron energy-loss spectroscopy (HREELS) measurements showed no substantial differences between the crystalline BL silica and vitreous BL silica.^147^ Note also that there are no vitreous structures formed in ML silica/Ru(0001), most likely due to the strong Si–O–Ru bonds that force the ML silica to be in registry with a Ru(0001) substrate.

##### 2.2.1.3 Crystalline–Vitreous Interface

**Figure 19:**
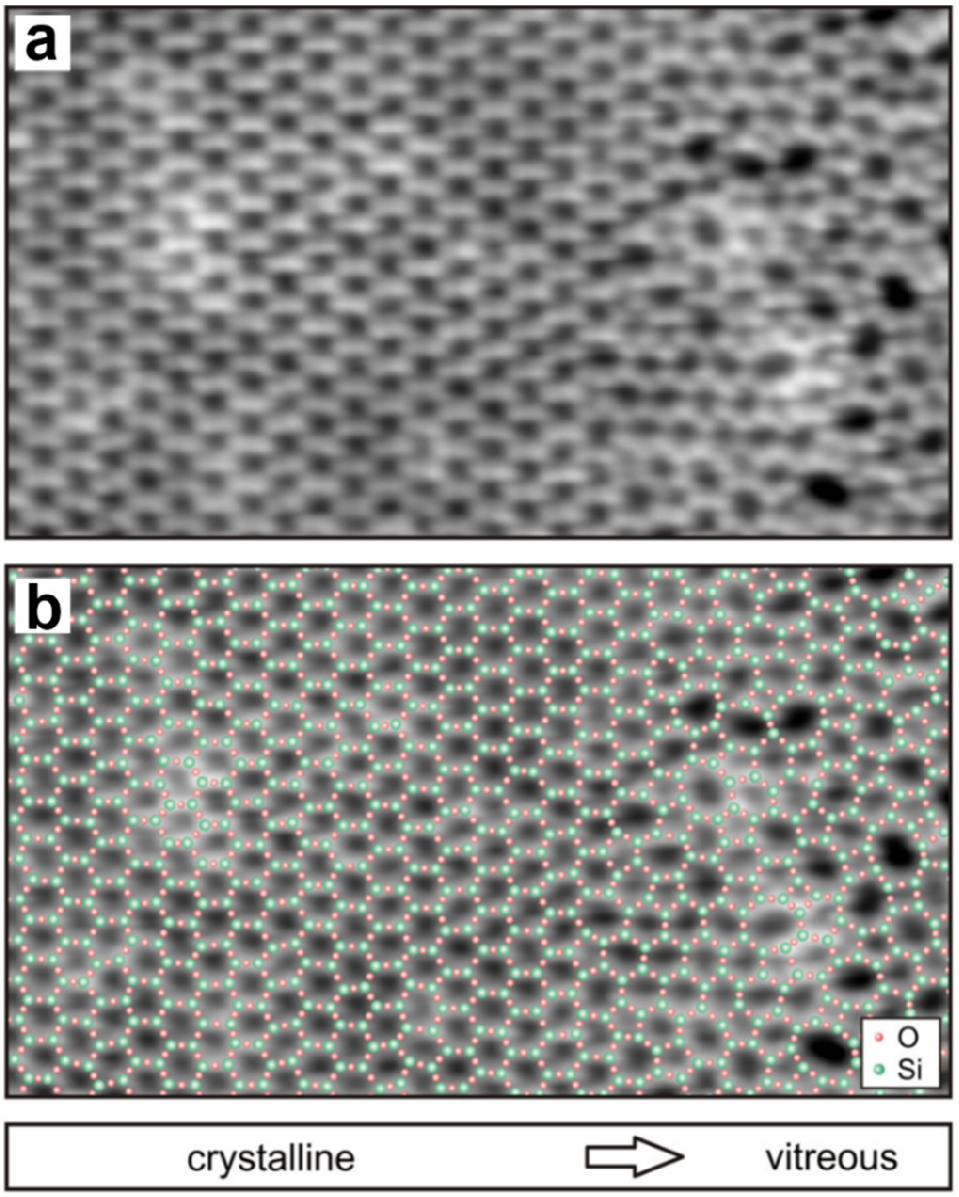
(a) STM image of the crystalline–vitreous interface in a BL silica/Ru(0001) (12.3 × 7.0 nm^2^, *U*_s_ = 2.0 V, *I* = 0.1 nA). (b) The same image with a superimposed atomic model of the topmost layer (O, small red balls; Si, large green balls). Reproduced with permission from ref (22). Copyright 2012 American Physical Society.

Direct STM imaging of the BL silica/Ru(0001) system allows us to study the structural transformation between the crystalline and vitreous phases with atomic resolution in real space. Figure 19 shows such an evaluation at the crystalline–vitreous interface of a silica bilayer film. The crystalline phase smoothly transforms into the vitreous phase without any “defects” in terms of unsaturated bonds or different atomic arrangements beyond [SiO_4_] tetrahedra. The distance of the Si–Si nearest neighbors (NN) stays constant (0.303 ± 0.025 nm) as we go from the crystalline to vitreous region, consistent with the Si–Si NN distances in bulk silica materials.^148^ However, the Si–Si NN directed distance orientation (DDO) shows a substantial change at the interface. Whereas in the crystalline region, the DDO assumes three discrete values (−60°, 0°, and 60°), reflecting the 3-fold symmetry of the crystalline structure, in the vitreous region, the orientations are randomly distributed from −90° to +90°. From the computed ring statistics, 5- and 7-membered rings appear first at the transition region, which is in line with the DFT calculations on the Stone–Wales defect.^92^ With an increasing lateral coordinate, 4- and 8-membered rings are also found. According to the crystallinity, the width of the transition region is about 1.6 nm. For comparison, the interface widths of 0.3–1.4 nm were obtained for the crystal-glass transitions in other 3D tetrahedral networks.^149−151^ This difference in the transition region widths originates most probably from the different interface systems (e.g., silica–silica vs Si–Si/SiO_2_, and 1D interface vs 2D interface).

##### 2.2.1.4 “Zigzag” Structures

**Figure 20:**
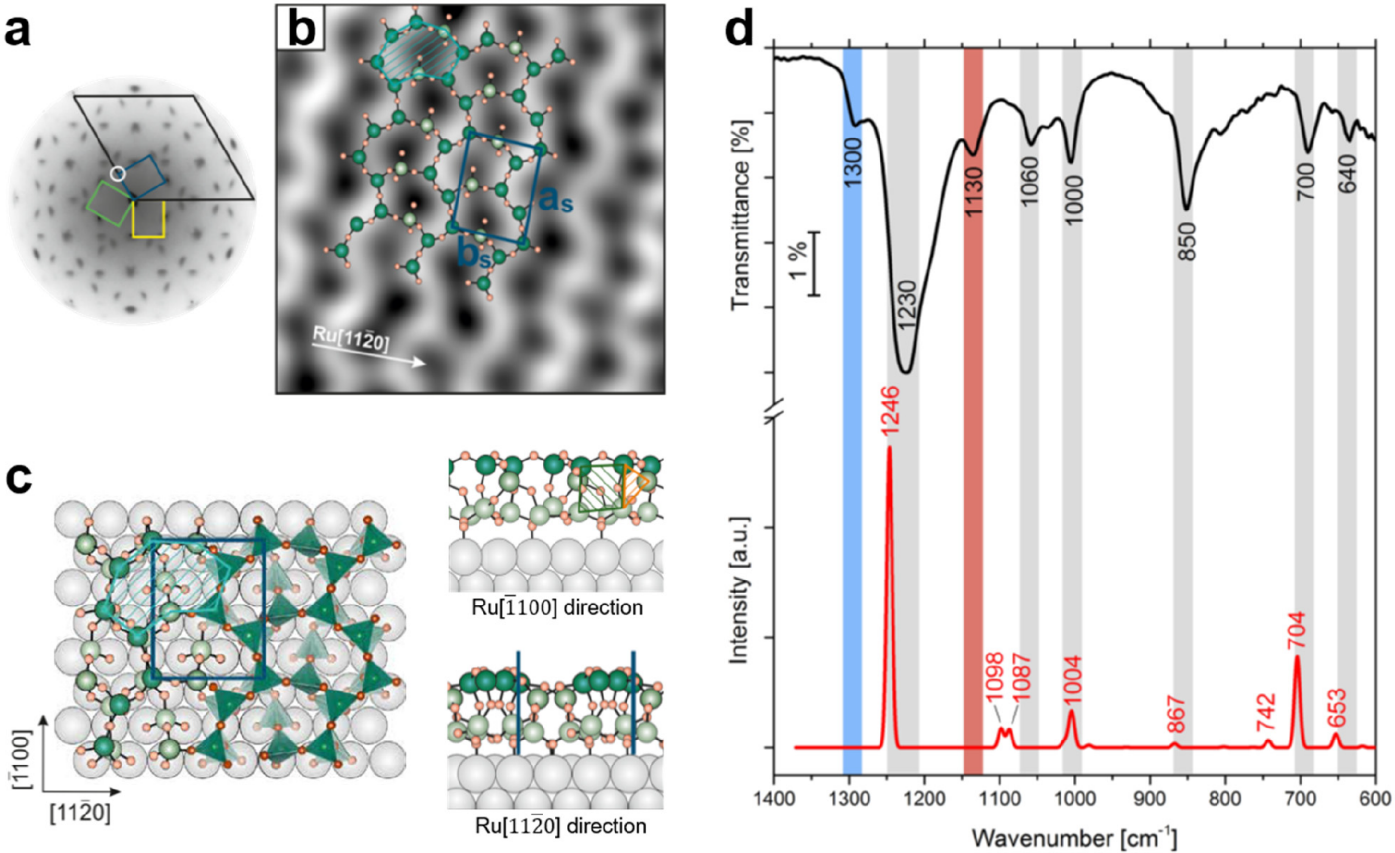
(a) LEED (at 42 eV) pattern of the “zigzag” silica on Ru(0001). The reciprocal unit cell of the Ru(0001) substrate is indicated with a black line, while the unit cells of the “zigzag” silica are indicated with blue, green, and yellow lines. (b) STM image of the “zigzag” silica together with a superimposed atomic model (3.8 × 3.8 nm^2^, *U*_s_ = 0.7 V, *I* = 0.02 nA). (c) Schematic top view (a combination of the ball and stick model and the SiO_4_-tetrahedral model representations) and side views (along the Ru[1̅100] and Ru[112̅0] directions) of the “zigzag” silica (topmost Si atoms, dark green; other Si atoms, light green; O atoms, orange; Ru atoms, gray). The overlayer unit cell is marked in blue. 8-membered, 4-membered, and 3-membered Si rings are marked with light blue, green, and orange hatched areas, respectively. (d) IRAS of the “zigzag” silica (black curve). DFT-calculated IRAS spectrum (red curve) is also shown for comparisons. The absorption peaks marked with red and blue bars are associated with the ML silica and BL silica, respectively. Reproduced with permission from ref (104). Copyright 2018 American Chemical Society.

While the silica ML films are directly bound to the Ru substrate, the crystalline and vitreous silica BL experience weak van der Waals (VDW) interactions with the Ru support. Recently, Kuhness et al. discovered a new silica structure with intermediate characteristics in terms of coupling to the substrate and stoichiometry, i.e., the “zigzag” silica/Ru(0001).^104^ The LEED pattern of the “zigzag” silica as shown in Figure 20a is clearly distinguishable from the typical LEED patterns of the ML silica (Figure 7a), crystalline BL silica (Figure 14a), and vitreous BL silica. From the LEED measurements, three reciprocal unit cells rotated by 120° with respect to each other are observed, and one real space unit cell was deduced with its unit cell vectors of **a**_**s**_ = 9.4 Å and **b**_**s**_ = 7.6 Å. In Figure 20b, a corresponding high-resolution STM image shows parallel zigzag lines which are oriented perpendicular to the Ru[112̅0] direction. These zigzag lines are actually interconnected by regularly appearing bridges.

In combination with DFT geometry optimizations, a structural model consisting of interconnected tetrahedral [SiO_4_] building blocks was proposed as shown in Figure 20c. Specifically, the zigzag lines may be traced back to vertical 4-membered Si rings, bound together via bridging [SiO_4_] units in the top layer of the system. Consequently, distorted nonplanar 8-membered Si rings are formed as viewed from the top, and vertically arranged 3-membered Si rings are formed as viewed from the side. In comparison to the ML silica, where all Si atoms bind to the metal substrate through Si–O–Ru linkages, the “zigzag” silica has only two Si atoms connected to the Ru support through two separate O atoms per unit cell. The stoichiometry for the “zigzag” silica is SiO_2.17_, different from ML silica (SiO_2.5_) and BL silica (SiO_2_).

Due to the complexity of the “zigzag” silica structure, the IRAS spectrum shown in Figure 20d exhibits more features. A theoretically calculated IRAS spectrum based on the above model generally reproduces the observed vibrational bands. The most prominent band at 1230 cm^–1^ originates from an antisymmetric stretching of the vertical Si–O–Si linkage, which goes in phase along the “zigzag” bilayer rows. The band at 1060 cm^–1^ is the corresponding antiphase stretching mode along the “zigzag” bilayer rows. The band at 1000 cm^–1^ can be assigned to an antisymmetric stretching mode of the vertical Si–O–Si in 3-membered rings, antisymmetric stretching of the horizontal Si–O–Si in 4-membered rings, and also the antisymmetric stretching of the bridging Si–O–Si that further connects to the Ru support. Other bands are attributed to the symmetric Si–O–Si stretching. It is essential to point out that small areas of coexisting ML silica and BL silica domains exist, with their intrinsic bands marked with the red (1130 cm^–1^) and blue (1300 cm^–1^) bars, respectively.

It should be noted that the “zigzag” silica was formed by oxidation at ∼1130 K in 10^–6^ mbar O_2_. Subsequent oxidation at a higher temperature of ∼1200 K will result in a single-phase BL silica structure, which cannot be transformed back to the “zigzag” silica again at varying preparation conditions. Therefore, the “zigzag” silica can be understood as a metastable silica phase.

##### 2.2.1.5 Evolution of Silica Polymorphs

So far, various silica polymorphs have been discovered on the Ru(0001) support, such as the chemisorbed ML silica, the physisorbed BL silica (crystalline and vitreous), and the chemisorbed BL silica (“zigzag”). All of them are composed of corner-sharing tetrahedral [SiO_4_] building units. Based on the STM measurements, different polymorphs have often been found to coexist with domain sizes ranging from 10 to 50 nm.^6,92,104,152^ Apart from the amount of deposited silicon, the critical parameters for steering the evolution to a particular silica polymorph are the oxygen pressure, growth/anneal temperatures, heating/cooling rate, and oxygen content on the Ru(0001) surface.

**Figure 21:**
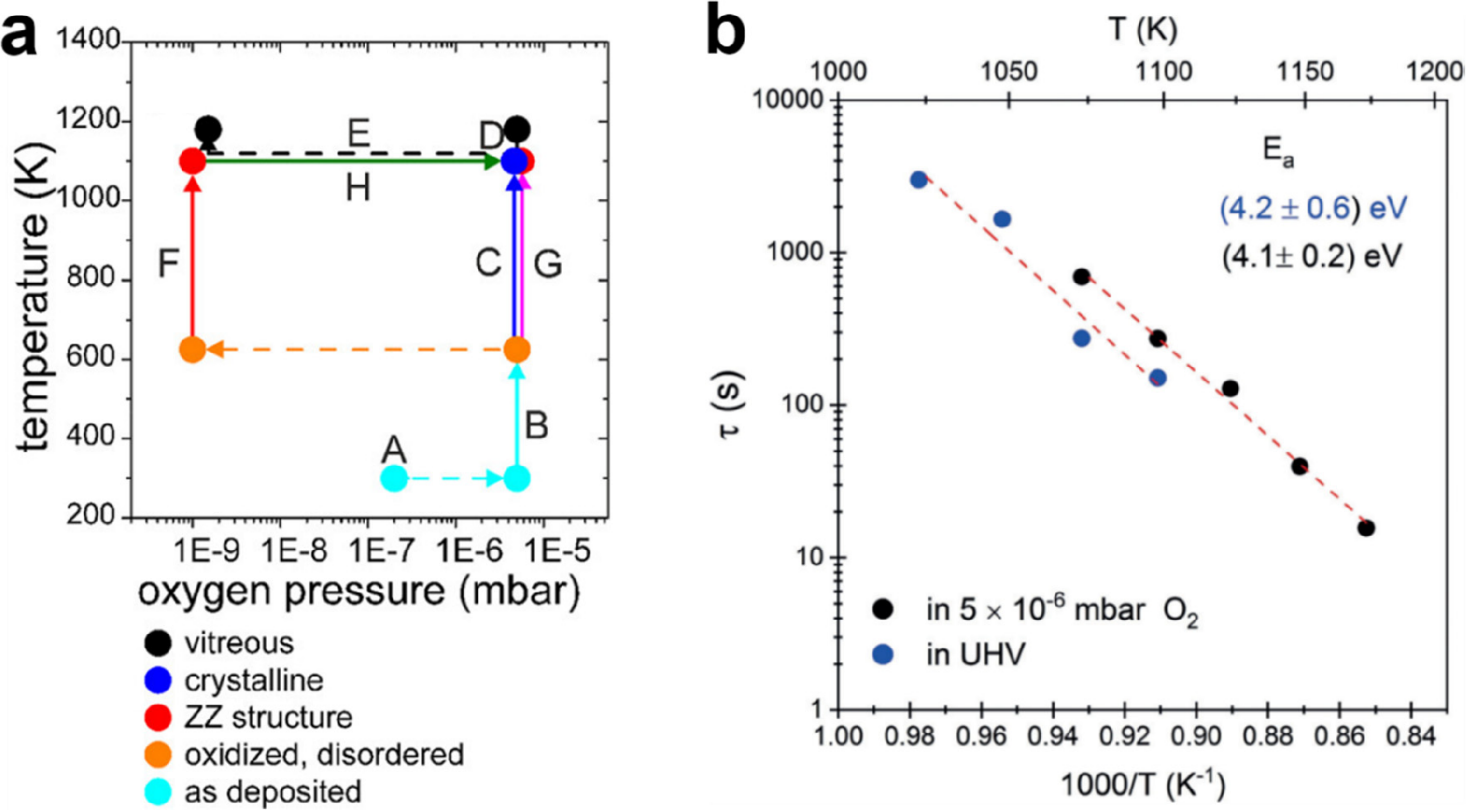
(a) Oxygen pressure and sample temperature-dependent preparation pathways for BL silica on Ru(0001). The solid points of different colors represent the obtained phases as listed in the figure. The solid lines with arrows indicate the annealing pathways and conversions, while the dashed lines indicate the changes in the oxygen pressure. (b) Transformations of the BL silica from crystalline to vitreous. Arrhenius plot of the time-constant values extracted from the fittings of the time-dependent (0, 0) LEED intensity curves. Reproduced with permission from refs (153 and 156). Copyright 2019 American Chemical Society, Copyright 2020 The Authors. Published by Wiley-VCH Verlag GmbH & Co. KGaA.

Figure 21a shows the connections among these BL silica polymorphs. It is essential to mention that all phases grow and convert homogeneously over the whole surface. Starting with the deposited Si, fast heating to the required crystallization temperature leads to the chemisorbed “zigzag” structure, while slow heating results in physisorbed BL silica. During the heating process, the concentration of surface chemisorbed O (O_Ru_) plays a critical role in determining the generation of self-contained physisorbed bilayers, where the formation of the Si–O–Ru bonds is suppressed. If the heating rate is higher than the O diffusion rate under the deposited layer, the chemisorbed “zigzag” structure will be formed; otherwise, the formation of the physisorbed BL silica dominates. The “zigzag” structure can be transformed into the physisorbed BL structure. The crystallinity (crystalline or vitreous) of the physisorbed BL silica is most likely determined by the cooling rate: the slower the cooling rate, the better the crystallinity. However, the crystalline BL silica can be converted to the vitreous one irreversibly.

Such crystalline to vitreous transformations have been studied in real-time with spectromicroscopy.^153^*In situ* low-energy electron microscopy (LEEM) and LEED have proven to be powerful techniques in phase transformation studies in different materials.^154^ Since the crystalline and vitreous silica bilayer films show characteristic LEED patterns,^88^ the intensity of the (0, 0) LEED spot was analyzed in real-time at variable temperatures and oxygen pressures. Freshly prepared crystalline BL silica has always been the starting point for all measurements performed at different temperatures. As shown in Figure 21b, the time constant (τ) values were extracted to describe how fast the film is transformed. From the analysis of the Arrhenius plot, the activation energies of (4.2 ± 0.6) and (4.1 ± 0.2) eV were obtained for the crystalline to vitreous transformations in UHV and O_2_ atmosphere, respectively. Therefore, it may be concluded that the O_Ru_ (O_2_ atmosphere) does not play a crucial role in the energetics of the phase transformation. This can be understood by the fact that the temperatures for the phase transformation are above the onset for the thermally induced desorption of O_2_ (950 K).^155^ The irreversibility of transformation could then be attributed to the existence of an even higher activation energy barrier, which is energetically unfavorable for the vitreous to crystalline phase transition.

Considering that the vitreous BL silica is characterized by a distribution of different ring sizes,^92^ it is reasonable to assume that the crystalline to vitreous transformation is caused by a number of consecutive rotations of the [SiO_4_] building units. As mentioned above in section 2.2.1.2, the formation of the first Stone–Wales defect (5–7–5–7 rings), introduced by consecutive rotation of two contiguous [SiO_4_] units in the top and bottom layers, can be rate-determining for the whole transformation process. According to DFT simulations, the transformation process is complex and involves separate subsequent changes on the different layers of the film. The DFT-calculated energy barriers for the Stone–Wales formation in freestanding BL silica and BL silica/Ru(0001) are 6.12 and 4.3 eV, respectively, in good agreement with the experimental values. Charge transfer between the BL silica and Ru(0001) substrate was found to stabilize various intermediates and to lower the activation energy barriers for breaking the Si–O bonds as compared to the freestanding BL silica.^153^

It should be noted that a defect-free BL silica was used in DFT as the starting point of the transformation process. However, as revealed by LEEM and STM studies,^152,156^ all silica polymorphs have domains with domain boundaries consisting of various defects. Take the crystalline BL silica/Ru(0001), for example, in addition to the Stone–Wales defect as discussed above; there are 5–5–8 antiphase, 5–7 rotational, and 4–8 domain boundaries. Obviously, these pre-existing defects will affect the transformation process, most likely by lowering the activation energies. It was reported that a Stone–Wales defect would cause a strain dissipation (compressive and/or tensile strain) over 2000 SiO_2_ unit cells.^157^

##### 2.2.1.6 Continuous Network Structures

**Figure 22:**
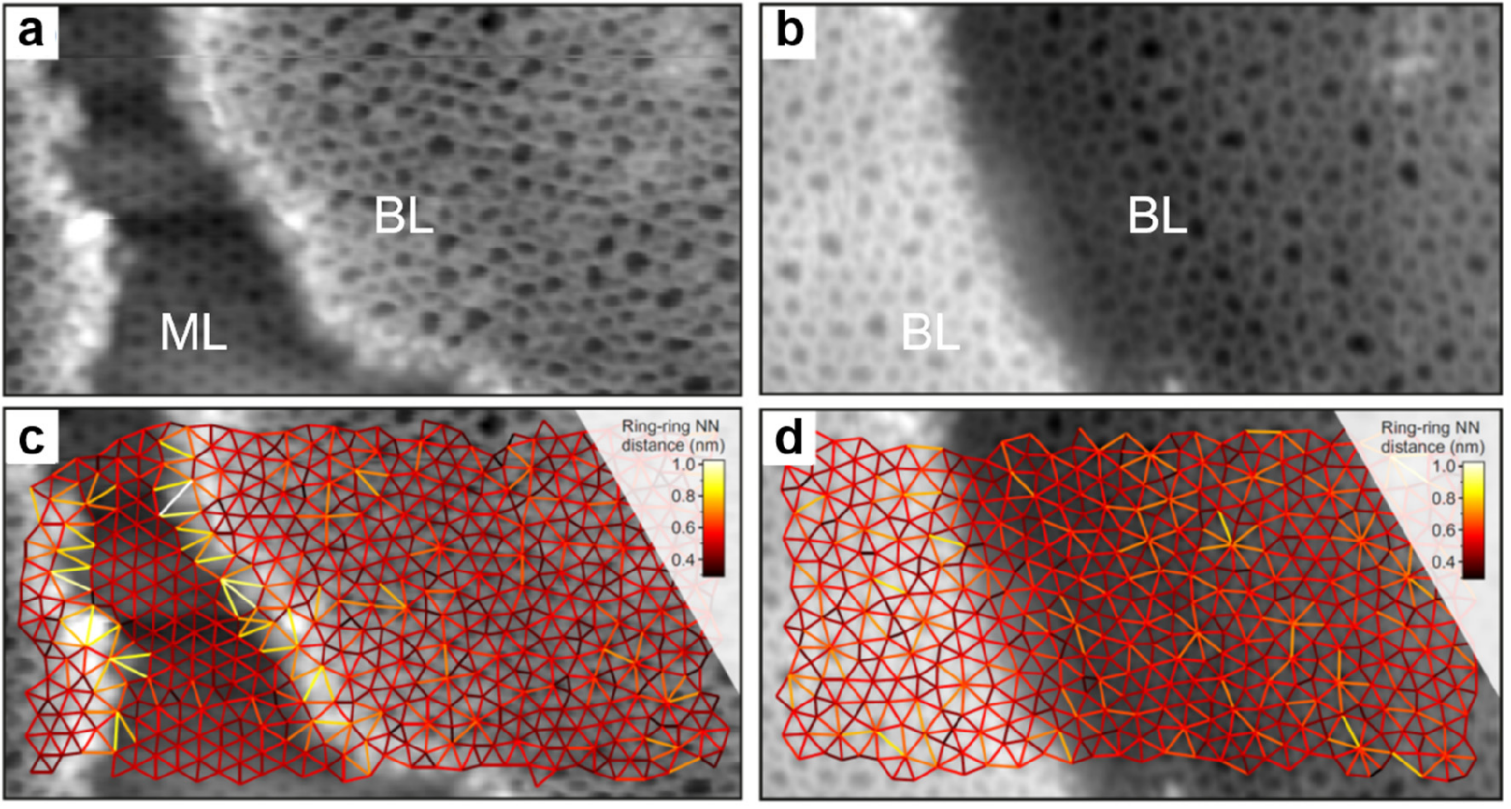
(a) STM image of a ML–BL silica transition region. (b) STM image of a BL silica across a single Ru(0001) step edge [15.3 × 8.1 nm^2^, (a) *U*_s_ = 1.0 V, *I* = 0.01 nA; (b) *U*_s_ = 2.0 V, *I* = 0.4 nA]. (c, d) The same STM images with a superimposed color-coded ring–ring network model. Reproduced with permission from ref (158). Copyright 2021 The Authors. Published by the American Physical Society.

The coexistence of different 2D-silica phases sometimes causes a stepped topography, such as at the ML–BL transition regions (Figure 22a,c).^158,159^ According to the STM investigations and the results from a semiautomated network detection program, the ring–ring distances are increased at the ML–BL transition region as compared to the uniform ring–ring distance distributions in the ML and BL regions, respectively. The STM image intensity also increases at the rim of the ML–BL transition, which is possibly caused by dangling bonds or chemically bound molecules. These results indicate that the upper layer of the vitreous bilayer is not connected to the monolayer phase.

Stepped topographies can also be observed for BL silica films across a supporting metal step edge (Figure 22b,d). In this case, the ring–ring distances are distributed uniformly across the step region, indicating a continuous random network structure across the Ru(0001) step edge. With the help of DFT calculations, two structural models (i.e., pinning mode and carpetlike mode) are proposed to explain such lateral smooth transitions of the BL silica from the upper to the lower terrace of the Ru(0001) substrate. In the pinning mode, the Si–O bonds at the bottom layer of the BL silica break at the step edge and bind to the substrate, which causes an almost steplike decrease of the line profile, while in the carpetlike mode, the topography is much smoother. In principle, the width of the Ru terraces plays a vital role in determining the detailed continuous network structures based on the DFT studies by applying both tensile and compressive strain to the structural models. Since wider Ru terraces provide better experimental conditions, the carpetlike mode is therefore preferably adopted. This observation is also in line with the higher bending rigidity of the silica bilayer.^160^ The origin of such continuous network structures of a silica bilayer across the Ru step edges may come from two aspects: the continuous coverage of the surface during the deposition process and the diffusion of the Ru atoms underneath the silica during the annealing process. This study provides an atomistic model for a freestanding BL silica that can be related to macroscopic properties.

#### 2.2.2 On Pt(111)

To elucidate the effect of metal supports on the atomic structure of 2D-silica films, we used Pt(111) as an alternative substrate and compared it with Ru(0001). The Pt(111) surface has the same crystal symmetry but with its lattice constant slightly larger than Ru(0001) (i.e., 2.77 vs 2.71 Å). As a precious metal, Pt(111) is less reactive and may exhibit a different effect during the silica film preparations as compared to Mo(112) and Ru(0001) supports. An O(2 × 2)-Pt(111) surface was first obtained prior to the Si deposition in ∼10^–6^ mbar O_2_ at ∼100 K. Final crystallization was performed in ∼10^–5^ mbar O_2_ at ∼1200 K.

**Figure 23:**
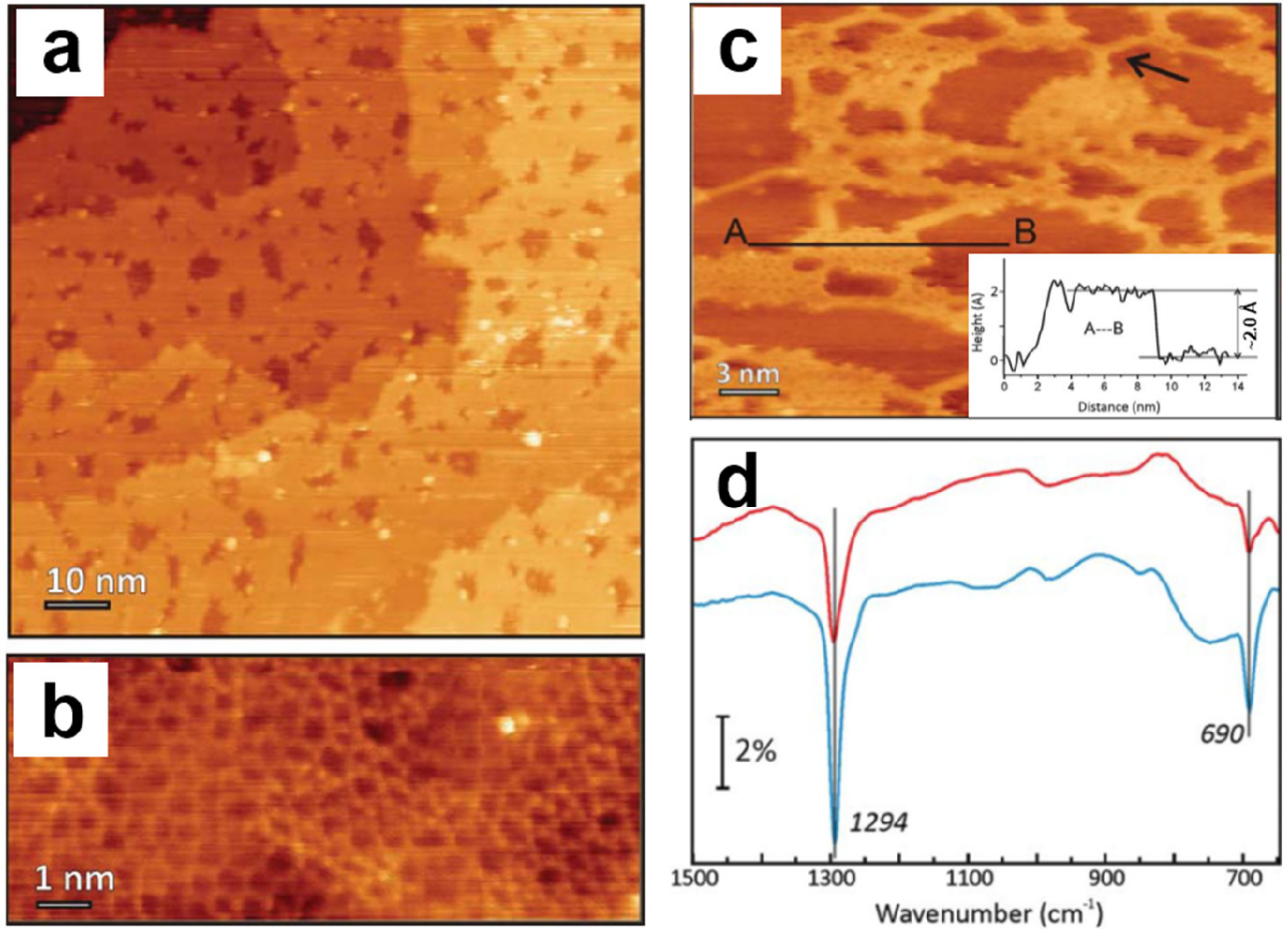
Large-scale and close-up STM images of 2 ML silica on Pt(111): (a) *U*_s_ = 4.4 V, *I* = 0.1 nA; (b) *U*_s_ = 1.3 V, *I* = 0.13 nA. (c) STM image of 1 ML silica on Pt(111) [*U*_s_ = 0.8 V, *I* = 0.06 nA]. The inset in panel c shows the height profile along the A–B line. The arrow indicates silica stripes bridging the islands. (d) IRAS of 1 ML silica (red curve) and 2 ML silica (blue curve) on Pt(111). Reproduced with permission from ref (7). Copyright 2012 AIP Publishing.

The XPS spectra of the BL silica/Pt(111) are very similar to those obtained for BL silica/Ru(0001).^6^ Only one chemical state of silicon was observed with a Si 2p BE of 102.5 eV, falling in the range of Si^4+^. The O 1s region reveals a prominent peak at 531.9 eV (O in silica), accompanied by a small shoulder at 530.1 eV (O on Pt, O_Pt_). The significantly weaker shoulder peak (contributing only ∼6% to the overall peak intensity) can be caused by the lower affinity for oxygen chemisorption on Pt(111). The STM images in Figure 23a,b reveal the vitreous nature of these 2D-silica films, similar to those obtained for vitreous BL silica/Ru(0001).^92^ The holes present in the film are ∼2 Å in depth. If the amount of deposited silicon was reduced by half, 2D-silica islands on Pt(111) with an apparent height of ∼2 Å (Figure 23c) were observed. The IRAS spectra of the 2D-silica film and islands show two strong bands at 1294 and 690 cm^–1^. In Figure 23d, no monolayer-related bands (1000–1100 cm^–1^) were observed. Moreover, the intensities of those two bands are simply proportional to Si coverages [e.g., from one monolayer equivalent (MLE) to two MLE], similar to the coverage-dependent XPS Si 2p spectra. It should be noted that the shoulder peak (O_Pt_) at ∼530 eV becomes more pronounced as the system exhibits more bare Pt(111) surfaces. It is essential to mention that the crystalline and monolayer structures observed on Ru(0001) have not yet been found at any combinations of preparation conditions studied.

Therefore, the above results demonstrate the formation of exclusively vitreous bilayer structures on a Pt(111) support.^89^ The apparent thickness (∼2 Å) obtained from STM measurements of the BL silica on Pt(111) is considerably smaller than that on Ru(0001) (i.e., ∼5 Å) but larger than the ML silica film on Ru(0001) (i.e., ∼1.4 Å).^88^ Such behavior can be attributed to the bias- and polarity-related electronic effects, which are ubiquitous in STM studies of metal oxide surfaces. This finding again demonstrates the importance of using a multitechnique approach to study the metal-supported thin oxide films, especially for the determinations of their atomic structures. Interestingly, as shown in Figure 23c, the 2D-silica islands are connected via stripes with stripe widths of 4–7 Å. These stripes mainly spread in the main crystallographic direction of Pt(111) and have the same height as that of the 2D-silica islands. However, as compared to the 1D-silica stripes that formed in the silica/Mo(112) system (see Figure 9), further studies are needed to identify the atomic structures of these stripes in silica/Pt(111).

#### 2.2.3 On Pd(100)

The preparation of ultrathin silica films on Pd(100) substrates can be dated back to 2007.^161^ Zhang et al. found that the silica films grown on Pd(100) with a thickness of 2.8 nm have smooth morphologies. Their vibrational and electronic properties are very similar to bulk silica. However, detailed structural models of these silica films were still unknown. In 2013, Altman et al. demonstrated that crystalline silica bilayers could be prepared on Pd(100). They found that the characteristic defects in the BL silica/Pd(100) are primarily determined by the lattice mismatch between the crystalline silica bilayer and the Pd(100) support.^93^

**Figure 24:**
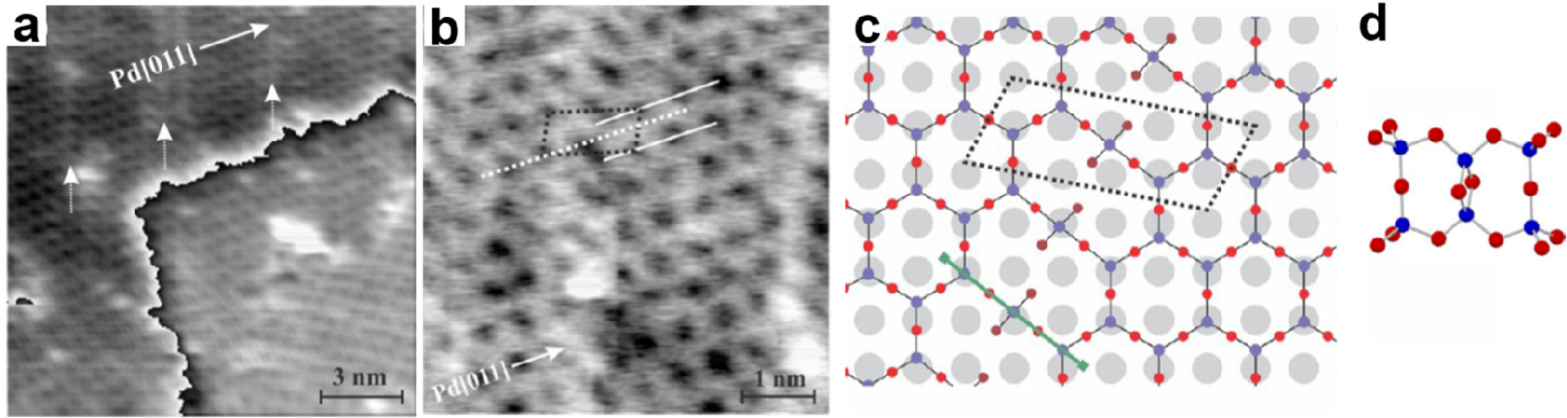
(a) STM image of a crystalline silica bilayer prepared on Pd(100) (*U*_s_ = −1.75 V). The white arrows indicate the antiphase-domain-boundaries (APDB). (b) Atomic-resolved STM image of the APDB. The dotted black parallelogram includes the closest hexagonal pores across the APDB. The dotted and solid white lines indicate the dislocation between the silica domains across the APDB. (c) Ball and stick model of the APDB (Pd, gray; Si, blue; O, red; O at a lower depth, darker red). The dotted black parallelogram is the same as that in panel b. (d) Side view of the bilayer structure along the direction as the green line indicated in panel c. Reproduced with permission from ref (93). Copyright 2013 American Chemical Society.

The preparation of a silica film at modest temperatures (<975 K) leads to a smooth but atomically disordered film. Auger electron spectroscopy (AES) confirms that the stoichiometry of these silica films is indeed SiO_2_. In contrast, annealing a film with less than 3 ML Si at higher temperatures of ∼1075 K in oxygen changes the structures dramatically. Both STM and LEED disclose a well-ordered hexagonal or nearly hexagonal silica film formed on Pd(100). Large-scale STM shows a different step-terrace morphology. The step edges are straightly aligned and directed to the square symmetry of the Pd(100) substrate instead of the hexagonal silica layer. Closed-up STM images show an expected 30° rotational domain boundary (lower right of Figure 24a) as well as several slightly brighter lines running over the terraces. These are antiphase-domain-boundaries (APDB), and their structures can be inspected more clearly in Figure 24b. The hexagonal silica lattices that extend along the Pd[011] direction are shifted in the Pd[01̅1] direction by a distance of roughly 0.3 nm at the APDB, which is in agreement with the dislocation of the 2D-silica domains, i.e., by a shift of one lattice constant of the Pd(100) substrate in the Pd[01̅1] direction. The dotted black parallelogram in Figure 24b includes the closest hexagonal pores across the APDB.

Based on the experimental observations, a model of the APDB structure is proposed in Figure 24c. There is an elongated 8-membered ring created by embedding a rotated [SiO_4_] tetrahedra into a 6-membered ring. This elongated 8-membered ring occurs in both the top and the bottom layer of the BL silica/Pd(100). Therefore, two remaining oxygen atoms from the inserted [SiO_4_] tetrahedra of the top layer can be shared with the inserted [SiO_4_] tetrahedra in the bottom layer. As illustrated in the side view of the APDB structural model, there are no dangling bonds (Figure 24d). Similar rotated tetrahedra have been suggested for Mo(112)-supported silica layers.^72,85^

The crystalline bilayer silica on Pd(100) has a lattice constant of 0.55 nm as deduced from STM and LEED data, which is larger than the computed values for a freestanding silica bilayer (0.53 nm). Therefore, there is considerable tensile stress in the BL silica/Pd(100). The frequently observed APDB along Pd[011] thus can be attributed to the intrinsic features of the silica bilayer, which can help relieve structural stress. Similar stress relief behavior has also been observed for an alumina film on a NiAl support.^162^ Since the crystalline structure of the silica bilayer is incommensurate with the square symmetry of the Pd(100) support, the silica domains are then large along the bilayer [1 1] direction, where they can be relaxed by contraction of the hexagonal structures.

As discussed in the last section, only amorphous BL silica films could be produced on Pt(111).^7^ As the interactions of oxygen with Pd(100) and Pt(111) are very similar, it has been proposed that the square symmetry of the Pd(100) substrate indeed favors the formation of the crystalline silica bilayer on Pd(100). The stress relief is realized by contractions, which originate from the films’ registry with the substrate, suggesting the possibility of growing crystalline BL silica on Pt(100) or Pt(110).

**Figure 25:**
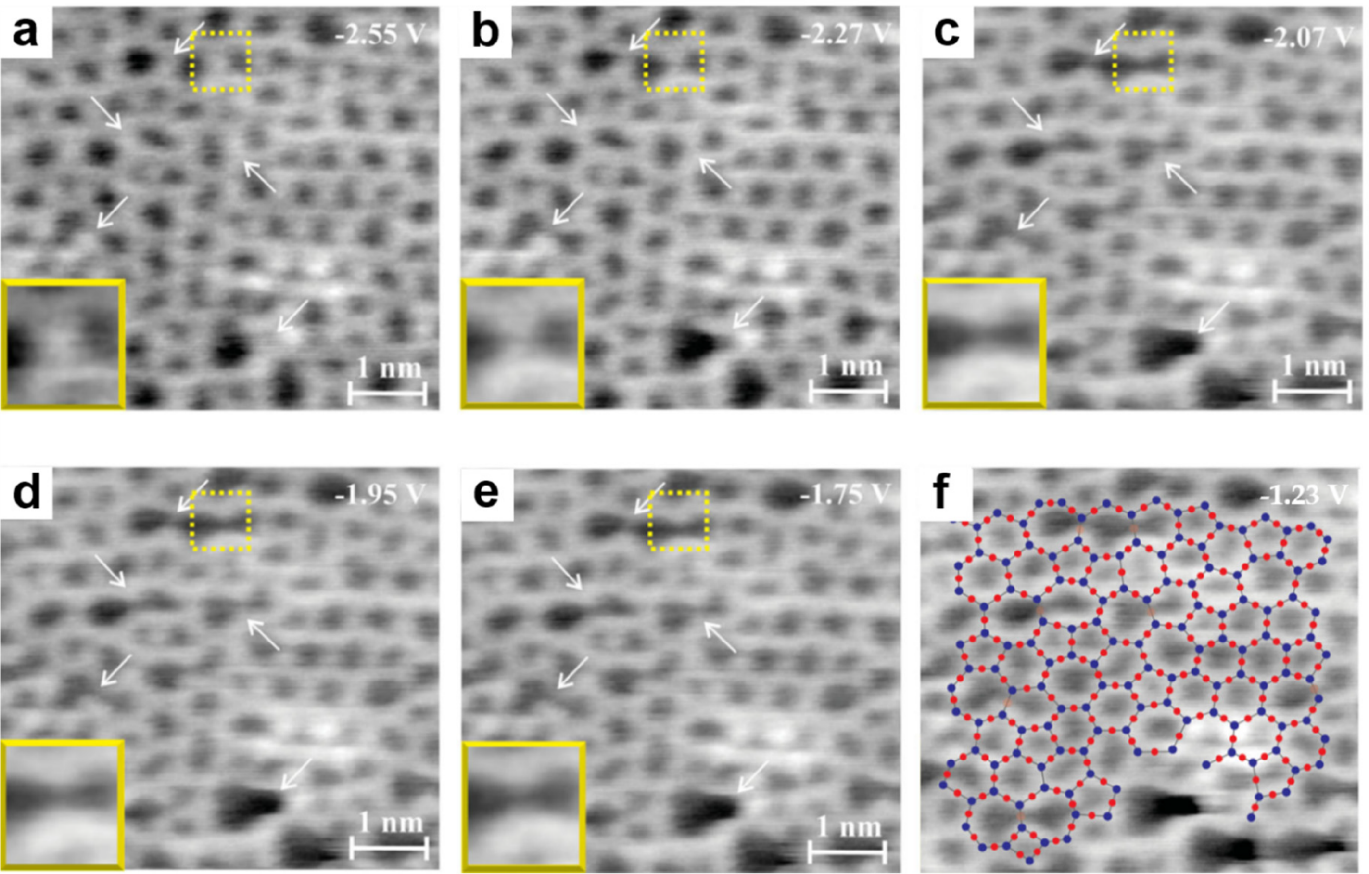
Bias-dependent STM images of an amorphous BL silica on Pd(100): (a) −2.55 V, (b) −2.27 V, (c) −2.07 V, (d) −1.95 V, (e) −1.75 V, (f) −1.23 V. The imaging rate was 0.15 frames/s at 256 × 256 pixel density. Arrows highlight locations where the image contrast changes. The insets in the yellow frames show expanded views of the dashed yellow box areas. Panel f is superimposed with an atomic structure (O, red balls; Si, blue balls). Reproduced with permission from ref (94). Copyright 2014 WILEY-VCH Verlag GmbH & Co. KGaA, Weinheim.

In addition to the crystalline phase, amorphous BL silica can also be grown on Pd(100).^94^ Their morphologies strongly resemble those obtained for amorphous BL silica on Ru(0001) and Pt(111).^7,92^ Interestingly, the structural disorder (i.e., a random network of 4- to 9-membered rings of corner-sharing [SiO_4_] tetrahedra) in amorphous BL silica/Pd(100) can induce variations in the local electronic properties. In Figure 25, a series of bias-dependent STM images were recorded at precisely the same area. Significant changes can be observed at these sites highlighted by the white arrows (and yellow boxes). The ridges or walls begin to vanish with progressively decreased sample bias (a positive bias refers to tunneling into unoccupied sample states). The STM images also show an elongation of the holes next to the disappearing walls. Moreover, the surface corrugation increased from 0.020 to 0.053 nm as the bias decreased from −2.71 to −0.75 V. It should also be noted that the vanishing walls bridge a 6-membered ring (or larger ring) and a 7-membered ring (or larger ring), which is associated with oxygen sites (Figure 25f).

These results demonstrate that a structural characterization alone cannot provide a complete description of the amorphous BL silica system. There are several possible origins for this local electronic heterogeneity. First, surface hydroxyls may be responsible for the contrast variations, although the preference for hydroxyl formation on larger rings is still unclear.^163^ Second, the distortions of the [SiO_4_] tetrahedra may result in a rehybridization of the oxygen atom from sp^2^ toward sp as the Si–O–Si angle increases.^164^ Therefore, the electron density around the oxygen sites becomes more diffuse as the hybridization decreases.^165^ Third, the contributions from the underlying metal support and its interactions with the BL silica also need careful consideration. Last but not least, a possible tilt of the [SiO_4_] tetrahedra at larger rings may also lead to variations in the coupling between the oxygens and the metal surface.^142^

#### 2.2.4 On Pd(111)

It has been shown that substrate interactions, substrate geometry, and lattice mismatch all play important roles in determining the structures of the 2D silica films.^99^ Formation of only amorphous BL silica on Pt(111) presumably is due to the considerable bilayer–substrate lattice mismatch (4.6% tensile)^93^ or the weak oxygen–metal bond strength.^7^ In contrast, both amorphous and crystalline BL silica were observed on Ru(0001) with a smaller lattice mismatch (2.2% tensile)^23^ or on Pd(100) with an intermediate lattice mismatch (3.8% tensile).^93,94^ On the square substrate, Pd(100), the crystalline BL silica contains nearly periodic domain boundaries that can be related to uniaxial strain relief in the film. However, the amount of tensile strain that a substrate can impart is still unknown. The Pd(111) surface, with a lattice constant of 2.75 Å [larger than Ru(0001), ∼2.71 Å, but smaller than Pt(111), ∼2.77 Å], has therefore been used by Jhang et al. to address these unresolved issues. This study provides a direct comparison with Pd(100) for the impact of surface geometry and with Ru(0001) and Pt(111) to study the effect of substrate interactions and strains.^101^

It was found that BL silica tends to form an incommensurate crystalline phase on Pd(111) as inferred from AES, LEED, STM, and DFT studies. The film with the best crystallinity can be obtained by annealing in 10^–6^ Torr O_2_ at 1000 K. Two domains rotated with respect to each other by 30° are observed in LEED/STM as well as the distinguishable satellite and moiré patterns, suggesting an incommensurate crystalline phase caused by a large biaxial lattice strain of 3.8%. Further experimental and theoretical work reveals that the lattice strain energy can be significantly reduced from 0.492 to 0.126 eV by replacing 25% of the Si with Al in the bilayer.

However, Tissot et al. later reported that only amorphous BL silica was obtained on Pd(111) at similar preparation conditions.^102^ They claimed that the silica on Pd(111) grows as a bilayer from the onset, the same behavior as on Pt(111).^7^ These two studies on Pd(111) further exemplify the complex nature of silica crystallization and the related phase transitions.

#### 2.2.5 On Ni_*x*_Pd_1–*x*_(111)

At least until now, it has been demonstrated that the affinity of a metal substrate to oxygen plays a decisive role in the growth of the principal structures (monolayer vs bilayer) of the silica films on metal substrates. At the same time, the lattice mismatch determines the crystallinity in the bilayer structures.^96^ Therefore, the careful selection of substrates with specific lattice constants is critical for low-strain growth of the BL silica films, as it is also for the growth of other van der Waals (VDW) materials.^166,167^ Epitaxial growth on transition metal alloy systems (e.g., Ni_*x*_Pd_1–*x*_) provides such opportunities for strain engineering with continuously tunable lattice constants. Hutchings et al. have successfully prepared highly crystalline BL silica on the Ni_*x*_Pd_1–*x*_(111) alloy surfaces tailored to match the lattice constant of BL silica.^103^

High-quality Ni_*x*_Pd_1–*x*_(111) alloy surfaces were prepared by employing a Cr_2_O_3_(0001) adhesion layer on α-Al_2_O_3_(0001) via molecular beam epitaxy (MBE). Cr_2_O_3_, with a thickness of 15–20 nm, was used to improve the high-temperature stability as well as the crystallinity of the Ni_*x*_Pd_1–*x*_(111) films. Ni_*x*_Pd_1–*x*_(111) films (∼50 nm in thickness) with side lengths (face-centered cubic unit cells) between 3.52 and 3.89 Å, corresponding to nearest-neighbor distances between 2.49 and 2.75 Å, were obtained depending on Pd concentrations.^168^ As the repeat length of the unstrained bilayer is ∼5.30 Å,^80^ a Ni_*x*_Pd_1–*x*_(111) substrate with a lattice constant of 2.65 Å was desired, corresponding to 52% Pd based on fits to Vegard’s rule.^168^ BL silica prepared on such Ni_*x*_Pd_1–*x*_(111) (*x* = 0.48) substrates showed a commensurate crystalline phase as assessed by AES, LEED, and STM.^103^ While point defects are still visible, the lack of strain helps eliminate the amorphous phase in BL silica, showing the potential of using alloy surfaces to manipulate 2D VDW material growth.

#### 2.2.6 On graphene/Cu

Interestingly, BL silica was also synthesized by accident during the chemical vapor deposition (CVD) growth of graphene on quartz-supported copper foils.^80^ In contrast to the metal substrates, the formation mechanism of BL silica on inert graphene had been speculated to most likely result from contaminants in the graphene growth furnace (e.g., oxidations of the copper foil and its reactions with the quartz substrate). Nevertheless, TEM and STEM clearly showed atomically resolved crystalline and amorphous regions of the BL silica supported on graphene, similar to the results of Lichtenstein et al.^92^

**Figure 26:**
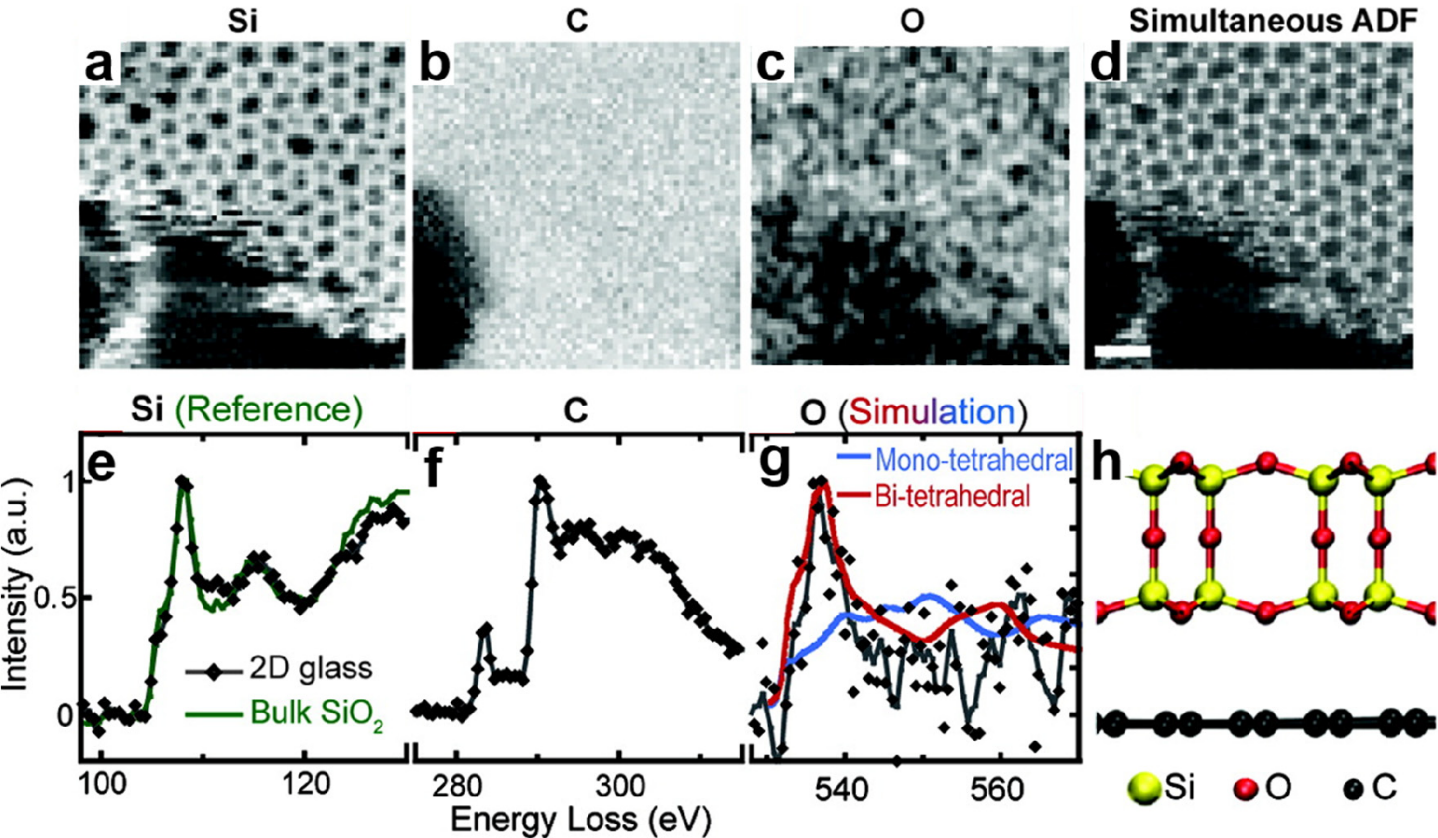
(a–c) EELS maps of the Si, C, and O distributions in a region of bilayer graphene partly covered by BL silica (top half). (d) Corresponding annular dark-field STEM image. Scale bar 2 nm. (e–g) Experimental EELS of BL silica (black lines) plotted with reference data (bulk α-SiO_2_, green line) and *ab initio* FEFF9^171^ simulations (blue and red lines). (h) Side view of the BL silica structure on graphene. Reproduced with permission from ref (80). Copyright 2012 American Chemical Society.

Scanning transmission electron microscopy (STEM) and electron energy-loss spectroscopy (EELS) were used to map the composition and bonding of the 2D-silica. Figure 26a–c displays the atomically resolved maps of the Si, C, and O distributions in silica/graphene, while Figure 26c–g shows the corresponding EELS spectra. Clearly, the Si L_2,3_-edges in 2D-silica are similar to bulk α-SiO_2_ with tetrahedrally bonded [SiO_4_] units. The C K-edge exhibits graphene-related fine structures, indicating the absence of the C–O bonding.^169^ The O K-edge spectrum is plotted together with the simulated spectra of monotetrahedral and bitetrahedral silica,^170^ and good agreement (a peak at 536 eV) is found for the BL silica structure. All of these experimental observations identify the formation of BL silica without detectable bonding to the graphene (Figure 26h). However, it should be noted that, in the bottom portion of the image (Figure 26a), where the silica bilayer structure is damaged, the Si atoms have SiC-like fine structures, indicating that they are bonded to the graphene edge.

By combining the BL silica/graphene and TEM techniques, the possibility arises to study the glass transition in real space at atomic resolution and eventually in real-time.^81^ Low-voltage aberration-corrected transmission electron microscopy (TEM) was used to image and restructure a 2D-silica, where the emitted electrons (with low probabilities) can transfer sufficient local energy to break bonds through elastic or inelastic scattering.^172−174^ Ring rearrangements from 5 to 7–5–7(vitreous) to 6–6–6–6(crystalline) have been observed in real-time (∼28 s). Based on the trajectory analysis and molecular dynamics simulations, it was found that elastic displacements (i.e., small motions of nearby atoms) are directly correlated to the plastic deformation (i.e., breaking and making of new bonds) of the ring rearrangements and likely represent the relaxation of the structure around the new ring configuration. It was also proposed that the deformation in amorphous 2D-silica is mediated by shear transformation zones.^175,176^ As discussed in section 2.2.1.5, such vitreous to crystalline transformation is energetically unfavorable based on classical thermal activations.^92,153^ However, the TEM studies here strongly suggest that external stimuli (e.g., the electron bombardment) can significantly affect the phase transformations in silica.

#### 2.2.7 On Ru, Co, and Fe Nanoplatelets

As compared to the accidentally observed silica growth on graphene/Cu, the solid-state growth of silica films on Ru, Co, and Fe nanoplatelets provides some insights into the growth mechanism.^96,177^ Crystalline metal (Ru, Co, and Fe) platelets with a thickness of ∼10 nm were prepared on amorphous SiO_2_ (10 nm) TEM grids at 973 K in separate experiments. The nucleation and growth of silica films on these metal platelets take place during the cooling process down to 723 K. On Ru and Co nanoplatelets, BL silica was observed, while on Fe nanoplatelets, only ML silica was observed. Such solid-state growth can be explained by bulk/surface diffusion of silicon and oxygen atoms through/on the metal platelets. As soon as the Si and O atoms (originating from the SiO_2_ grid substrate) migrate to the metal surface, silica films start to grow at the grain boundaries by reducing the surface metal oxide. Both crystalline and vitreous silica structures may be grown, which is determined by the kinetics of the growth process. It is important to mention that the ML is energetically favorable with respect to the BL under certain conditions according to the experimentally observed and theoretically calculated growth dynamics.

### 2.3 Thick Layer Structures

In principle, silica can be grown on many substrates that are stable to high temperatures and are less reactive toward oxygen than silicon. With increased amounts of deposited Si, poorly defined silica films will be formed, e.g., on Mo(100),^51^ Mo(110),^52,53,178^ Mo(112),^57,60^ Ni(111),^179^ Pd(100),^161^ Pt(111),^89^ and Ru(0001).^88^ This shows that decomposition or material loss will occur during the high-temperature annealing for these thick silica films, indicating a self-limiting growth mechanism for monolayer or bilayer films.

**Figure 27:**
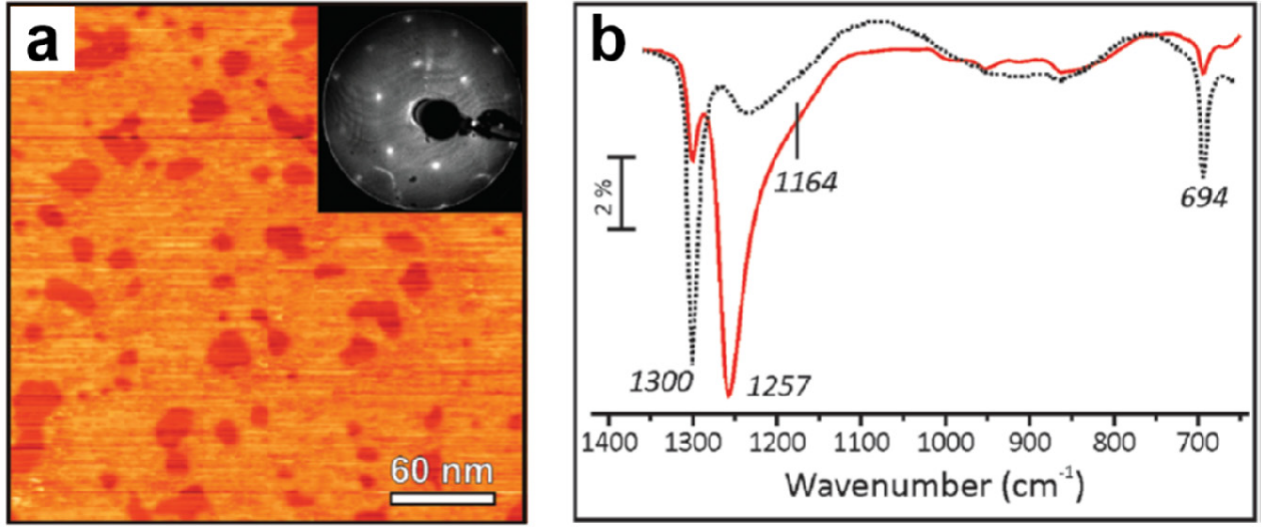
(a) STM image of 4 ML silica film on Ru(0001) [*U*_s_ = 9.0 V, *I* = 0.1 nA]. The inset in panel a shows the LEED pattern. (b) IRAS of 4 ML silica film on Ru(0001). The dashed line in panel b shows the IRAS for 2.2 ML silica film for comparison. Reproduced with permission from ref (88). Copyright 2012 Royal Society of Chemistry.

For example, thicker silica films were grown by deposition of 4 ML Si on Ru(0001).^88^ The results were almost independent of whether the films were prepared in sequential deposition or a one-step deposition. A large-scale STM image in Figure 27a reveals a smooth surface, albeit not atomically flat. Due to the insulating nature of these thick silica films, attempts to achieve atomic resolution were not successful as the STM imaging becomes unstable at low biases. However, there are no additional features in LEED besides the (2 × 2) diffraction spots and the ring. As in the cases of ML and BL structures, substantial changes are observed in IRAS spectra (Figure 27b): the bands at 1300 and 694 cm^–1^ attenuate significantly, and a new band develops at 1257 cm^–1^ with a prominent shoulder at 1164 cm^–1^. The shape and position of this band (1257 cm^–1^) are characteristic of the longitudinal optical vibration modes in bulk silica.^47^ Therefore, it appears that thicker films exhibit a 3D network of [SiO_4_] tetrahedra, rather than the layered structure observed for mono- and bilayer films, indicating the absence of the layer-by-layer growth mode. In this case, the termination of the film may be complex and ill-defined, which results in relatively high surface corrugation as measured by STM. Further efforts remain to be devoted to elucidating the surface structures of these thick vitreous silica films.

Very recently, atomic layer deposition (ALD) has been demonstrated to be a viable method for the scalable production of 2D-silica.^107,108^ BL silica (and thick silica) can be grown via ALD on Au and Pd polycrystalline foils by depositing a few cycles of bis(diethylamino) silane, followed by an oxygen plasma treatment and high-temperature annealing. In summary, a number of experimental parameters during the preparations can affect the atomic structure of 2D-silica, for example, substrate choice, coverages, thermal treatments, deposition method, etc. A complete understanding of these influencing factors will require more systematic investigations.

## 3 Chemical Modifications of the 2D-Silica

In the previous section, the preparation and characterization of pure 2D-silica films have been considered. These 2D systems provide unique opportunities to visualize the atomic structures of both crystalline and vitreous silica directly and open new playgrounds for studying model catalysts involving silica films.^82^ For example, aluminosilicates can be synthesized by doping with Al atoms or as supports for anchoring specific metal species, which will be discussed in this section and section 4, respectively. This section will introduce several methods developed for chemical modifications of the 2D-silica during/after initial preparations, including the incorporation of additional chemical elements, hydroxylation of the hydrophobic surfaces, and the interface engineering of the energy levels, thereby increasing the complexities of the 2D-silica systems significantly.

### 3.1 Metal Doping in 2D-Silica

In 2D-silica, the tetrahedrally coordinated Si atoms can be substituted by other atoms, which are commonly referred to as T-atoms regardless of their chemical nature. Doping 2D-silica is achieved by sequential deposition or codeposition of silicon and the material chosen as dopant, followed by high-temperature oxidation described for pure 2D-silica in section 2. From a structural point of view, three types of doping can be differentiated: isomorphic substitution, formation of nontetrahedral building units, and formation of coordinative disorder.^180^ So far, Al-,^9,10^ Fe-,^95^ Ti-,^11^ and Ni-doped^105^ 2D-silica films have been successfully prepared to model the internal surfaces of zeolites,^181^ with particular emphasis on the structures and properties of the Al-doped 2D-silica (i.e., aluminosilicate) due to their wide usage in heterogeneous catalysis.^182,183^

#### 3.1.1 Aluminosilicate

In inorganic chemistry, aluminosilicates are silicates in which some of the Si^4+^ ions are replaced by Al^3+^ ions. The resulting excess negative charges are balanced by positive ions, such as H^+^ or alkali metal cations.^183^ Zeolites are microporous members of the aluminosilicates family and play an important role in industrially relevant catalytic processes, as well as in the preparation of adsorbents. Millions of hypothetical zeolite structures have been proposed based on topological considerations, and 255 structures had been realized as of March 2022.^184,185^

The first attempts to prepare model zeolites to be studied by surface science techniques are documented by the work of Somorjai and co-workers.^186^ They prepared silica–alumina films (<10 nm) by an argon-ion-beam-sputter deposition on a gold foil using HY-zeolites as targets. In contrast, Goodman and co-workers used direct deposition of metallic Al onto a silica film supported on Mo(100).^187^ These approaches resulted in homogeneous but amorphous silica–alumina films with electronic structures similar to bulk aluminosilicates. However, the precise determination of the structures and studies toward structure–property relationships were hampered by the lack of structural definition.^188^

##### 3.1.1.1 ML Aluminosilicate/Mo(112)

**Figure 28:**
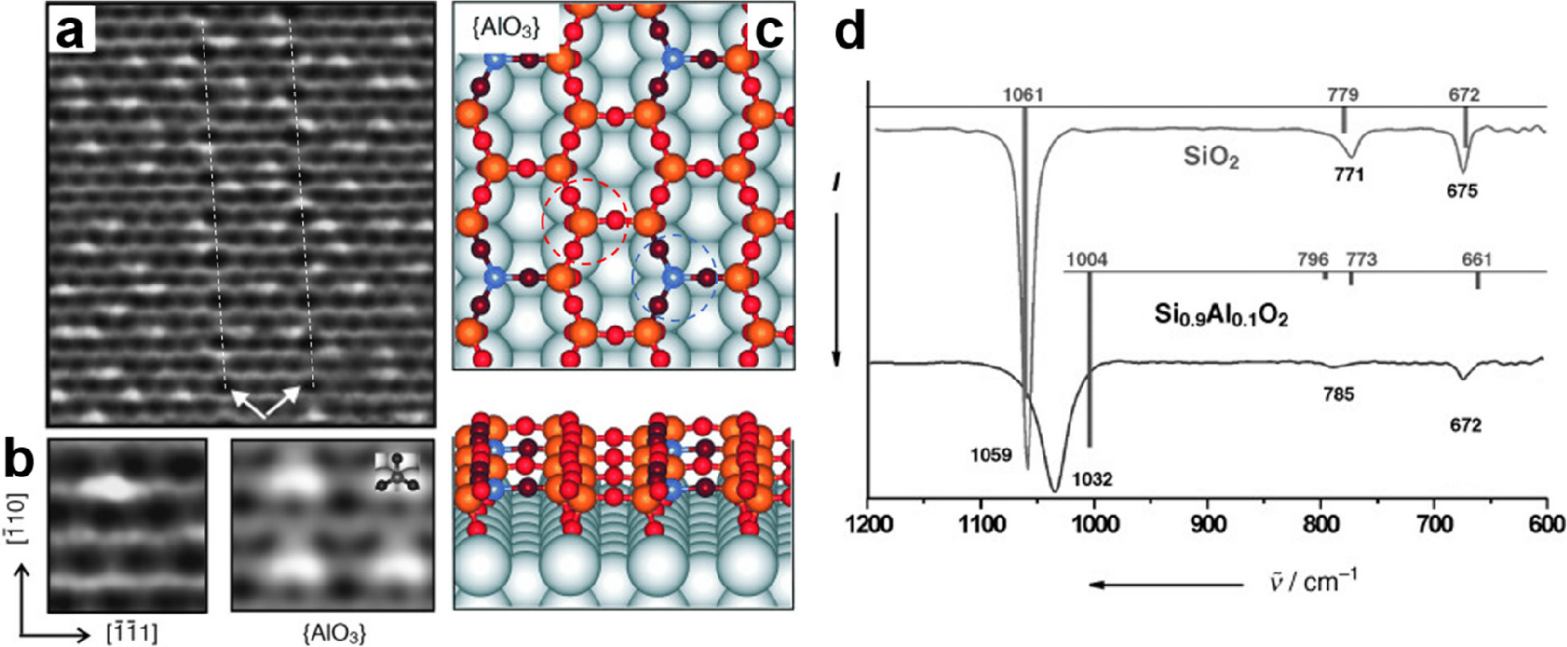
(a) High-resolution STM image of ML aluminosilicate (Al/Si ≈ 1:5) on Mo(112) substrate (8 × 6 nm^2^, *U*_s_ = 1.2 V, *I* = 0.3 nA). The dashed lines indicate APDB consisting of a row of 8- and 4-membered rings along the Mo[1̅10]. (b) Close-up STM image (left) with bright asymmetric protrusions and simulated STM image (right) with superimposed [AlO_3_] units. (c) Top and side views of the [AlO_3_] model of aluminosilicate ML on Mo(112). The [AlO_3_] and [SiO_4_] building units are highlighted by blue and red circles, respectively (Mo, gray; Al, blue; Si, orange; O, red). (d) IRAS of ML aluminosilicate (Al/Si ≈ 1:9) on Mo(112) substrate. Bars indicate the calculated frequencies, and the height of the bars is proportional to the IRAS intensity. The spectrum for silica/Mo(112) is also presented for comparison. Reproduced with permission from ref (9). Copyright 2006 WILEY-VCH Verlag GmbH & Co. KGaA, Weinheim.

Stacchiola et al. first reported a well-ordered ultrathin aluminosilicate film on a Mo(112) substrate following the discoveries of crystalline silica/Mo(112).^9^ Codeposition of Al and Si onto the Mo(112) surface in ambient oxygen was used for preparation to facilitate the intermixing of components in the films (with Al/Si ≈ 1:5). High-temperature annealing at ∼1100 K results in an ordered c(2 × 2) structure, revealing STM images similar to pure silica/Mo(112).^5^ However, many bright spots (slightly elongated along the Mo[111]) were observed on aluminosilicate in addition to the same honeycomb-like structure and antiphase-domain-boundaries (APDB) (Figure 28a,b) as in pure silica. The density of these spots is proportional to the Al content as determined by XPS (in the range of low Al/Si ratios). The random distribution of these Al-related features suggests a random distribution of the Al ions in aluminosilicate/Mo(112).

Further, DFT calculations were performed to determine the atomic structure of Al-substituted silica. As shown in Figure 28c, an [AlO_3_] model was proposed, in which some Si^4+^ ions in [SiO_4_] tetrahedra are replaced by Al^3+^ ions. The Al^3+^ ions are only coordinated by three O^2–^ ions from the top layer of the film (i.e., [AlO_3_]). Since no H^+^ was detected in IRAS, the extra charge may be accommodated by electron transfer from the Mo substrate. As compared to the [AlO_4_] model, where the Al^3+^ ions are also bonded to interface oxygen atoms, the [AlO_3_] model shows better agreement with the experimental observations (STM, XPS).^9^

The IRAS spectrum taken on an aluminosilicate/Mo(112) film shows a principal peak at 1032 cm^–1^ (asymmetric Si–O–Mo stretching), which is red-shifted and significantly broadened as compared to the IRAS spectrum of the pure silica/Mo(112) film (Figure 28d), indicating a strong influence by the Al dopants. Also, the weak peaks around 800–600 cm^–1^ (symmetric Si–O–Si stretching and bending) were affected by the presence of [AlO_3_]. It should be noted that there are some discrepancies between the experimental and DFT-calculated frequencies, most likely originating from the randomly dispersed Al ions in real aluminosilicate films that cannot be represented perfectly by using a small unit cell in performing the calculations. Nevertheless, the calculations correctly predict the red-shift of the principal peak and the splitting of the weak peak (around 780 cm^–1^) as compared to pure silica films. Therefore, it is concluded that, at low Al/Si ratios, the aluminosilicate film on Mo(112) exposes a 2D network of corner-sharing [AlO_3_] and [SiO_4_] units.

##### 3.1.1.2 BL Aluminosilicate/Ru(0001)

**Figure 29:**
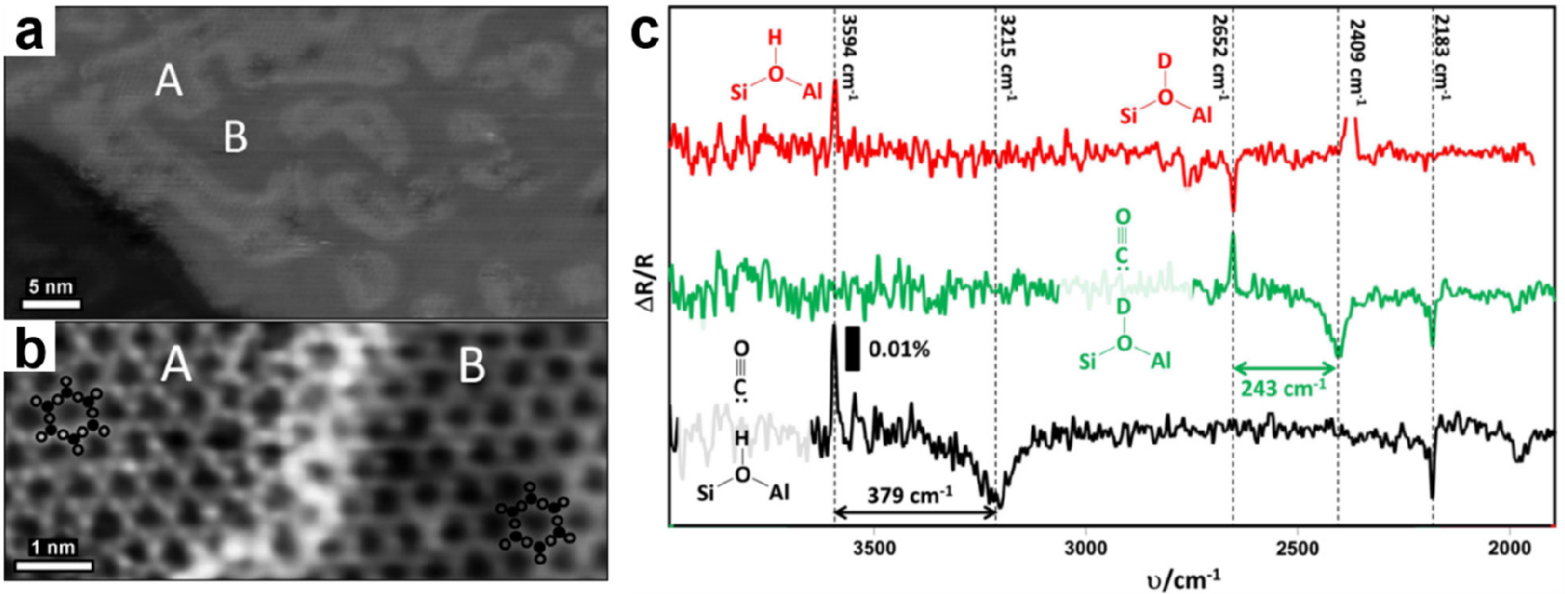
(a) Large-scale and (b) high-resolution STM images of an Al_0.12_Si_0.88_O_2_ film (*U*_s_ = 0.15 V, *I* = 0.07 nA). Two distinct domains labeled as A and B are indicated in STM images. The positions of the Si and O atoms in the top layer are shown by black dots and open circles, respectively. (c) IRAS of the Al_0.4_Si_0.6_O_2_ films on Ru(0001). Black and green spectra: hydroxylation with H_2_O and D_2_O, respectively, recorded in 2 × 10^–5^ mbar CO. The red spectrum is that of the OH-terminated surface upon subsequent hydroxylation with D_2_O. Reproduced with permission from ref (10). Copyright 2012 WILEY-VCH Verlag GmbH & Co. KGaA, Weinheim.

Similar isomorphic substitution can also take place in BL silica. In contrast to a random distribution of Al ions in ML aluminosilicate/Mo(112), the BL aluminosilicate/Ru(0001) exhibits segregations of Al-rich domains and all-Si domains (labeled as A and B, respectively) at low Al/Si ratios. It has a composition of Al_*x*_Si_1–*x*_O_2_, where *x* is the Al molar ratio. The Al_0.12_Si_0.88_O_2_ film, as shown in Figure 29a, exhibits both domains with predominantly honeycomb-like structures, however, with disordered morphologies at the A–B boundaries. The protrusions in domain A are assigned to the oxygen atoms in the top layer of the BL aluminosilicate (Figure 29b), whose proportion of the total surface area increases with the amount of deposited Al. To some extent, these findings contradict Dempsey’s statement^189^ on the Al arrangement in zeolites (i.e., the Al ions prefer to be arranged as far as possible from each other due to the electrostatic interactions). This effect may be explained by considering the Al^3+^ ions as defects, which induce strain. The total strain may be minimized via gathering these defects (e.g., the Al–O–Si–O–Al linkages) in one area.^190^

At low Al/Si ratios (*x* < 0.25), the isomorphic substitution occurs preferentially in the bottom layer. The metal substrate provides an electron reservoir to compensate for charge imbalances caused by the substituted Al^3+^ ions. When the Al/Si ratio approached 1 (*x* = 0.5), it was impossible to prepare well-defined films, which is in line with Loewenstein’s rule, saying that an Al/Si ratio equal to 1 is the largest possible ratio in zeolitic frameworks.^191^ Therefore, it is important to note that Al will populate the bottom layer first until *x* = 0.25; subsequent Al atoms start to occupy sites in the top layer.

For these BL aluminosilicate films, no new features are observed in IRAS as compared to the BL silica (see Figure 15, with characteristic bands at 1300 and 690 cm^–1^). However, with increasing Al content, the high-frequency band in aluminosilicates gradually red-shifts (about −30 cm^–1^ at *x* = 0.4), while the low-frequency band blue-shifts (about +10 cm^–1^ at *x* = 0.4) and broadens. The observed changes suggest that while the vibrations are affected by the Al substitutions, the tetrahedral building blocks are preserved.

The charge imbalances due to the incorporated Al^3+^ ions in the top layer are compensated by surface hydroxyls (*x* > 0.25) with characteristic stretching OH (OD) vibrations at 3594 (2652) cm^–1^ (see the black and green spectra obtained from the Al_0.4_Si_0.6_O_2_ film in Figure 29), which falls into the frequency range of bridging hydroxyl groups (Si–OH_br_–Al) known for zeolites.^192^ These bridging hydroxyls (OH_br_ or OD_br_) are thermally stable up to 650 K. Moreover, the H–D exchange reaction, a well-known phenomenon in zeolite chemistry,^193^ was also observed in the films when D_2_O was adsorbed on the OH-terminated surface. These findings indicate that the BL aluminosilicates can be suitable model systems for zeolites.

##### 3.1.1.3 BL Aluminosilicate/Pd(111)

A similar BL aluminosilicate may also be prepared on Pd(111).^99,101^ Jhang et al. found that the BL aluminosilicate/Pd(111) (Al_0.25_Si_0.75_O_2_, *x* = 0.25) has commensurate crystalline and amorphous structures. This result contrasts the pure BL silica/Pd(111) discussed in section 2.2.4, which only forms an incommensurate crystalline structure due to a large biaxial lattice strain. Doping of Al^3+^ ions into the framework eliminates one of the two preferred orientations and facilitates the phase transformations from an incommensurate to a commensurate structure. LEED and STM measurements show an inhomogeneous distribution of Al^3+^ ions in the BL films, allowing the amorphous phase to compete with the crystalline phase.

DFT calculations demonstrate that the replacement of Si atoms with Al (*x* = 0.25, the Al atoms only at one side of the bilayer) causes the expansion of the equilibrium lattice constant and the decrement of the 2D bulk moduli for the freestanding systems. The larger lattice constant (5.395 vs 5.309 Å) and softer nature (23.47 vs 28.07 eV Å^–2^) of the BL aluminosilicate help to reduce the energy penalty for lattice matchings. For example (at *x* = 0.25), the lattice strain energy can be significantly reduced from 0.492 to 0.126 eV per unit cell on Pd(111), and from 0.166 to 0.006 eV per unit cell on Ru(0001).^101^ Moreover, the interface distance between the bilayer and Pd(111) substrate (i.e., the O_bottom_–Pd distance) is reduced from 2.89 Å (SiO_2_) to 2.22 Å (AlSi_3_O_8_), indicating a much stronger interaction for aluminosilicate on Pd(111). The calculated PDOS shows a chemical bond involving charge transfer from the Pd support to the aluminosilicate, thus creating a driving force to form a commensurate layer.

As reported by Altman et al., the lattice mismatch is vital to controlling bilayer silica structures.^8,99^ The incorporation of the Al^3+^ ion then provides an additional possibility for structure control via strain. However, the appearance of the amorphous phase and the spatial variations in the Al concentration remain challenges that must be surmounted in the preparation of specifically desired aluminosilicates.

#### 3.1.2 Fe-Silicate

Following similar approaches, we can prepare other zeolitic films containing transition metal cations. For example, the Fe-silicates and Fe-zeolites, which are widely used in several industrially important oxidation reactions, can be synthesized by substituting a small fraction of Si^4+^ with Fe^3+^ in the framework.^194^ They can be very complex due to a large number of different Fe coordinations inside and outside of the crystalline framework.

##### 3.1.2.1 Monolayer of Clay: Fe_4_Si_4_O_16_·2O/Ru(0001)

**Figure 30:**
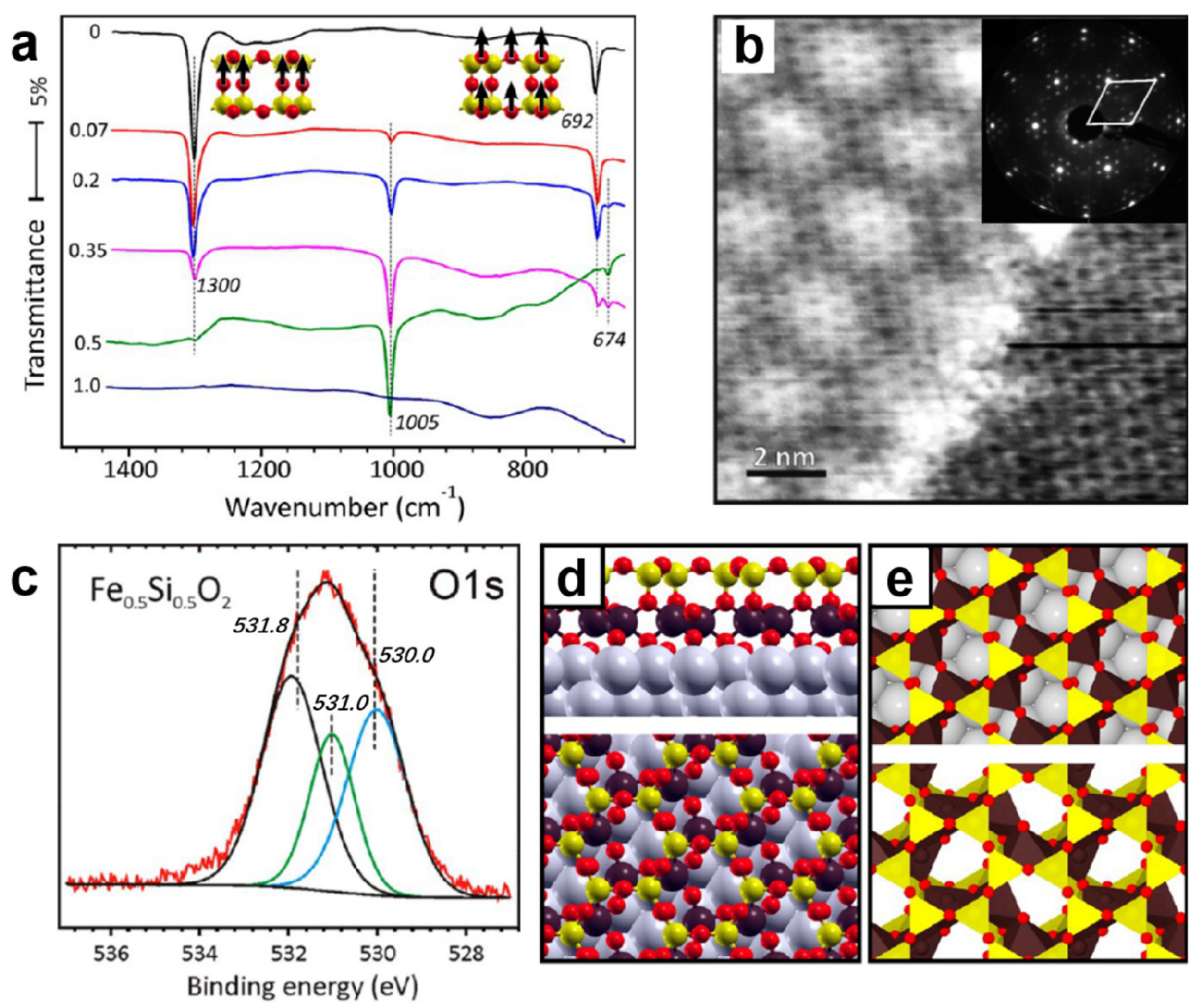
(a) IRAS of Fe_*x*_Si_1–*x*_O_2_ films on Ru(0001) with increasing Fe content (*x*). (b) High-resolution STM images of the Fe_0.2_Si_0.8_O_2_ film on Ru(0001) (*U*_s_ = 0.47 V, *I* = 0. 08 nA). The inset in panel b shows the LEED pattern. (c) XPS of the O 1s core-level in Fe_0.5_Si_0.5_O_2_ film on Ru(0001). (d) Top and side views of the most stable structure of Fe_4_Si_4_O_16_·2O/Ru(0001) found by DFT. (e) Top views of Fe_4_Si_4_O_16_·2O/Ru(0001) (top panel) and dehydroxylated nontronite^198^ (bottom panel) in polyhedral representation. Si, yellow; Fe, dark violet; O, red; Ru, gray. Reproduced with permission from ref (95). Copyright 2013 American Chemical Society.

Fe-doped 2D-silica films were prepared on Ru(0001) in the same way as aluminosilicates.^10,95^Figure 30a collects a series of IRAS spectra for the Fe-silicate films with increasing Fe content (*x*). For all films (Fe_*x*_Si_1–*x*_O_2_), the sum of the molar amounts of Fe and Si was equal to the Si necessary to prepare the BL silica film. Clearly, the bands at 1300 and 674 cm^–1^ gradually attenuate and ultimately disappear at *x* ≈ 0.5, whereas a sharp and strong band at 1005 cm^–1^ together with a weak band at 674 cm^–1^ appear and gain intensity with increasing Fe content. Such spectral evolution suggests a two-component system, where the films spatially segregate into Fe-containing and pure silica phases. For comparison, Al doping in silica (i.e., aluminosilicate) only causes red-shifts and broadens the principal phonon bands (e.g., from 1300 to 1270 cm^–1^).^10,195−197^ As revealed by STM and LEED (Figure 30b), the unit cell of the Fe-silicate films is rotated by 30° with respect to Ru(0001) with a shortened lattice constant of 5.25 Å. The appearance of the moiré structure also indicates a lattice mismatch to the metal support. It is essential to mention that the surface area of the moiré structure linearly increases with increasing Fe content, ultimately expands over the entire surface at *x* ≈ 0.5, and, therefore, can be assigned to the Fe-silicate phase. The XPS O 1s core-level of the Fe_0.5_Si_0.5_O_2_ film shows a relatively broad peak. It can be deconvoluted into three oxygen species according to the XPS analysis of different Fe_*x*_Si_1–*x*_O_2_ films, i.e., the O atoms in Si–O–Si (531.8 eV), Si–O–Fe (531.0 eV), and Fe–O–Fe (530.0 eV) coordination, respectively (Figure 30c).^95^ The signal of Fe–O–Fe coincides with the peak of surface chemisorbed O atoms (Ru–O) at ∼530.0 eV.

Based on the experimental results, the Fe-silicate is structurally very different from the 2D aluminosilicate films on Ru(0001). In fact, it can be described as a silica monolayer on top of an iron oxide monolayer on Ru(0001) with an Fe_4_Si_4_O_16_ composition, which is firmly supported by DFT calculations. Bilayer structures of Fe_*n*_Si_8–*n*_O_16_·2O/Ru(0001) (*n* = 0–4) are modeled by sequential Fe substitutions of the bottom layer Si atoms. It turned out that the Fe atoms prefer to segregate into the Fe-rich structure, and the phase separation is a thermodynamically driven process (at *n* = 1–3). Surprisingly, at *n* = 4 [i.e., Fe_4_Si_4_O_16_·2O/Ru(0001)], the bilayer structure significantly rearranges as shown in Figure 30d, where the bottom layer becomes a 2D network of edge-sharing and corner-sharing [FeO_5_] square pyramids ordered in 6-membered rings. The formation of bridging Fe–O–Ru bonds significantly increases the adhesion energy and the charge transfer from the Ru support to the adlayer. Moreover, the bottom and top layers are slightly shifted with respect to each other. Therefore, perpendicular Si–O–Si linkages between layers are no longer present in Fe_4_Si_4_O_16_·2O/Ru(0001). The simulated IRAS spectrum is in excellent agreement with the experimental one. The band at 1005 cm^–1^ is assigned to the Si–O asymmetric stretching vibrations oriented perpendicular to the surface. The lower-frequency band at 674 cm^–1^ originates from the Si–O–Si bending and is only slightly affected by the presence of the Fe in films.

The structure of prepared Fe-silicate films has close similarities to the mineral nontronite, representative of Fe-rich smectites. In an ideal nontronite, an octahedral Fe-hydroxide sheet is sandwiched in two tetrahedral silicate sheets. From this perspective, the Fe-silicate film on Ru(0001) can be viewed as a single sheet of dehydroxylated nontronite, where the tetrahedral silicate sheet is replaced by the Ru support (Figure 30e). According to the composition of dehydroxylated nontronite [i.e., Si_4_Fe_2_O_11_, or (Fe_2_O_3_)(SiO_2_)_4_ with an oxidation state of iron +III], the unit cell composition of the Fe-silicate film can be written as (FeO_2_^–^)_4_(SiO_2_)_4_·2O/Ru(0001), where the adlayer is formally reduced, and the Ru substrate serves as an electron reservoir. The structural difference between Al- and Fe-silicate films mimics the different behavior of naturally occurring Al- and Fe-silicate materials. In aluminosilicate films, the Al^3+^ is present in 4-fold coordination as found in natural zeolite materials, whereas in Fe-silicate films, the iron oxide is present in layered structures in combinations with silica, a characteristic for clay minerals.

##### 3.1.2.2 Fe-Containing Aluminosilicate/Ru(0001)

Fe-containing aluminosilicates (e.g., Fe-ZSM-5) are active catalysts in the selective catalytic reduction of nitrogen oxides^199^ and the oxidation of benzene to phenol.^200^ However, the nature of active species in Fe-ZSM-5 remains controversial due to the variations in Fe coordination.^194^ 2D model systems of Fe-containing aluminosilicate were then studied by incorporating iron atoms in aluminosilicate bilayer films grown on Ru(0001).^100^

Two different approaches have been attempted to prepare Fe-containing aluminosilicates. The first one includes Fe deposition on top of the prepared BL aluminosilicate/Ru(0001). The Fe atoms can readily migrate through the film and adsorb on the Ru(0001) substrate as revealed by IRAS and XPS results. Upon subsequent oxidation at ∼800 K, the bilayer structure becomes poorly defined, and 3D nanoparticles (silica/alumina or iron oxides or both) were formed on top of the film (or directly bonded to the Ru support).

**Figure 31:**
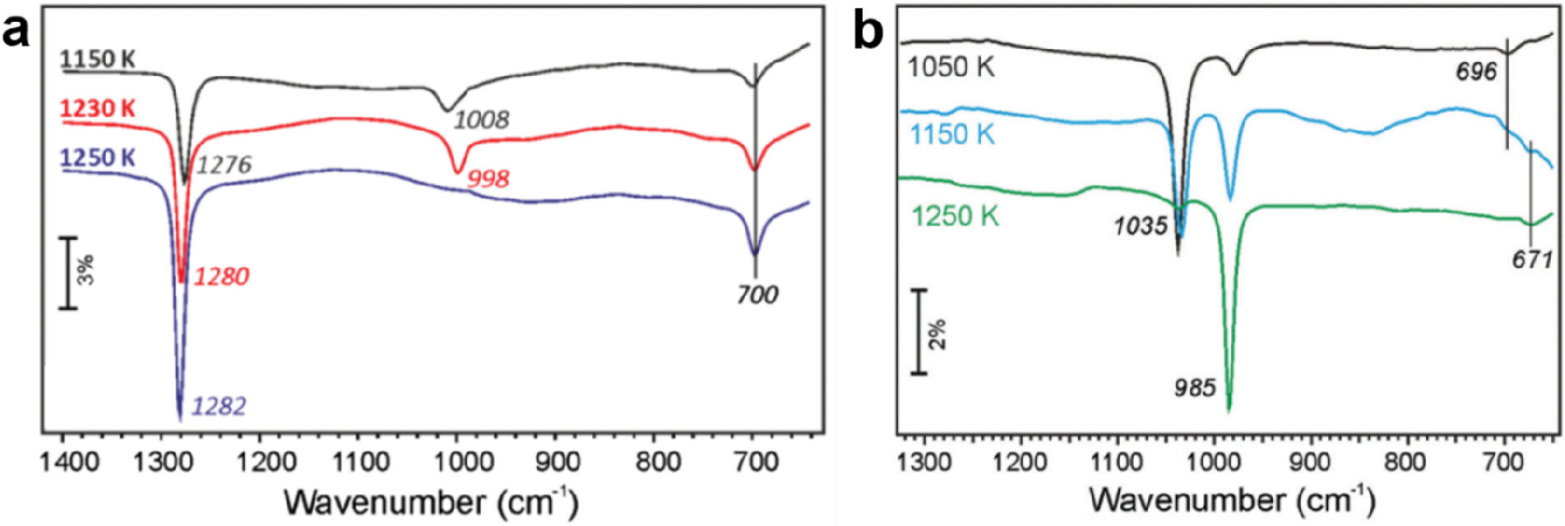
IRAS of the (a) Fe_0.25_Al_0.2_Si_0.55_O_2_ and (b) Fe_0.5_Al_0.13_Si_0.37_O_2_ films prepared on Ru(0001) by annealing in 10^–6^ mbar O_2_ at the temperatures as indicated. Reproduced with permission from ref (100). Copyright 2016 The Authors. Published by Royal Society of Chemistry.

The second preparation employs codeposition of Fe and Si(+Al), which is followed by crystallization in an oxygen environment at high temperatures. At a relatively low Fe/(Si + Al) molar ratio (e.g., the Fe_0.25_Al_0.2_Si_0.55_O_2_ in Figure 31a), the Fe-containing aluminosilicate film segregates into pure aluminosilicate and Fe-silicate phases, similar to that observed on Fe-doped silica films.^95^ The film becomes better ordered upon further oxidation at a higher temperature of 1230 K. The IRAS band associated with the aluminosilicate phase gains intensity and shifts to the higher frequency at 1280 cm^–1^, while the band corresponds to the Fe-silicate phase becomes narrower and shifts to the lower frequency at 998 cm^–1^. By increasing the oxidation temperature to 1250 K, Fe-silicate-related features vanish, and the film becomes virtually identical to a pure aluminosilicate film, as judged by IRAS.

At a considerably high Fe/(Si + Al) molar ratio (e.g., the Fe_0.5_Al_0.13_Si_0.37_O_2_ in Figure 31b), no pure aluminosilicate phase was formed. In contrast, two principal bands at ∼1035 and ∼985 cm^–1^ fall in the range of the Fe-silicate phase, although both frequencies somewhat deviate from the Fe-silicate phase in Fe_0.25_Al_0.2_Si_0.55_O_2_ (1008–998 cm^–1^). With increasing the oxidation temperatures, the IRAS band at 1035 cm^–1^ is attenuated while the band at 985 cm^–1^ is enhanced. According to the STM and LEED results, these two bands possibly correspond to a less-ordered Fe-silicate structure and a highly ordered Fe-silicate structure, in which both structures are terminated by an (alumino)silicate top layer. At low oxidation temperatures, Al ions can still reside in the bottom layer together with iron oxide to constitute a poorly defined bottom layer. However, they were pushed out by iron oxide from the bottom layer and segregated as alumina clusters at the surface at higher oxidation temperatures. These results suggest that it is thermodynamically unfavorable to form in-frame Fe species in zeolites.

##### 3.1.2.3 Fe-Induced Crystallization of the 2D Silicates

As discussed in sections 2.1.1.1 and 3.1.1.1, ML silica and aluminosilicate are often obtained in the crystalline form due to their strong interaction with the metal support (e.g., via the Si–O–Mo linkages),^5,9^ whereas their BL versions exhibit more structural flexibility with a broad distribution of *n*-membered rings because of the relatively weak interactions with the metal substrates.^10,92^ Interestingly, Fe-doped BL silica and aluminosilicate films show almost 100% crystallinity even though they are prepared at considerably lower annealing temperatures than those used for pure BL films. For example, the Fe-silicate phase starts to form at ∼1000 K and decomposes at ∼1200 K, while pure silica starts to form at ∼1200 K and remains stable up to ∼1275 K.

**Figure 32:**
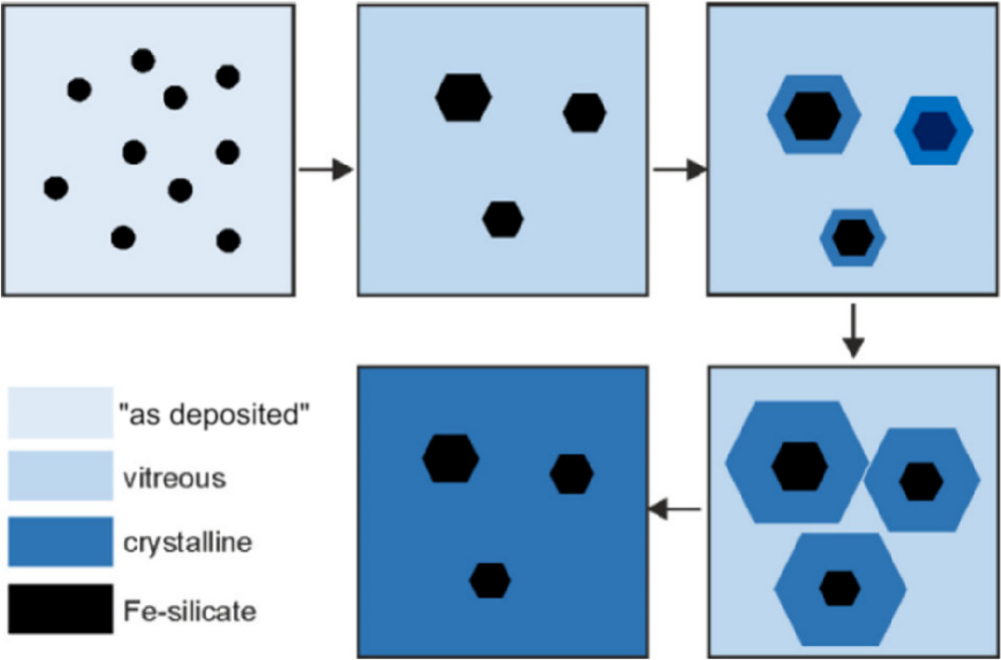
Growth scenario of the crystalline Fe-modified silica films. Reproduced with permission from ref (201). Copyright 2017 American Chemical Society.

It was proposed that the Fe-silicate phase can trigger the formation of a crystalline BL silicate structure, which propagates as a “crystallization wave” outward from the Fe-silicate until covering the entire substrate as shown in Figure 32.^201^ Therefore, the early formed Fe-silicate domains at relatively low temperatures behave as “seeds” to improve the film crystallinity and lower the preparation temperatures. Moreover, as described in Figure 30b, the entire Fe-silicate films are rotated by 30° with respect to Ru(0001). Fe-induced crystallization can now be verified via the “30°-rotated Fe-silicate domain”, which has strong interactions with the Ru(0001) substrate via the Fe–O–Ru bonds and drives the entire BL silicate films to grow in the same orientation through good epitaxial relationships at the interface to the Fe-silicate.

#### 3.1.3 Ti-Silicate/Ru(0001)

Titanium silicates are catalysts of interest in the field of fine chemicals.^202^ For example, titanium silicate-1 (TS-1), a structural analogue to the zeolite ZSM-5, shows outstanding catalytic performance and stability in the epoxidation of 1-hexene.^203^ In contrast to 3D complex structures, Ti-deposited 2D-silica films have been previously studied by Goodman and co-workers in order to figure out the properties at the TiO_*x*_-SiO_2_ mixed oxide surfaces.^204^ Ti at varying coverages was deposited onto silica/Mo(112) surfaces, followed by oxidation at 600 K and high-temperature annealing. It was found that significant restructuring occurs upon annealing to temperatures above 1000 K, such as the formation of Si–O–Ti linkages on monolayer silica/Mo(112) or 3D TiO_2_ clusters on multilayer silica/Mo(112).

**Figure 33:**
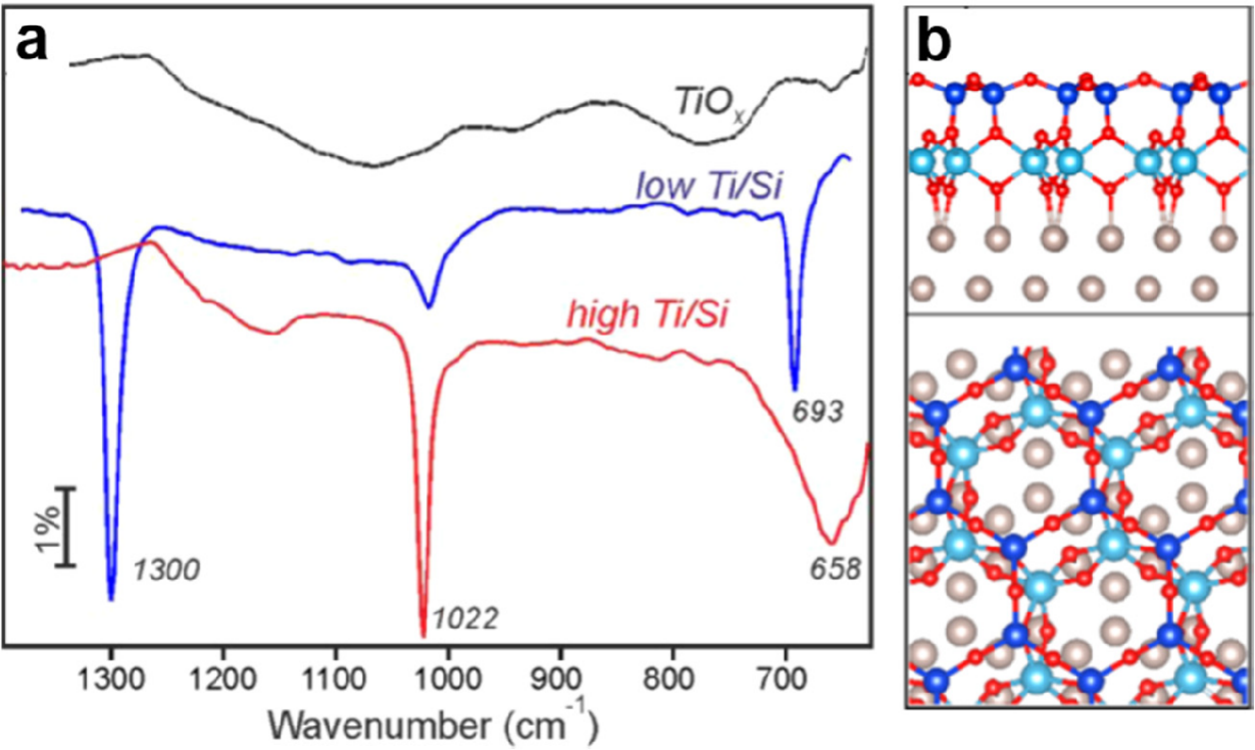
(a) IRAS of the Ti-silicate films with low- and high-Ti content. The spectrum for the TiO_*x*_ overlayer prepared under the same conditions is also shown for comparison. (b) Top and side views of the most stable structure for Ti_4_Si_4_O_16_·2O/Ru(0001). Si, blue; Ti, light blue; O, red; Ru, gray. Reproduced with permission from ref (11). Copyright 2015 American Chemical Society.

The detailed structural model for the atomic structure of ultrathin Ti-silicate was revealed by Fischer et al.^11^ Both experimental and theoretical results show that the Ti-silicate has a very similar structure to the Fe-silicate/Ru(0001). Specifically, a uniform distribution of Ti in the silica bilayer framework is energetically unfavorable, and the BL film will segregate into pure silica and a Ti-silicate phase (Figure 33a, the IRAS band at 1022 cm^–1^ originates from the stretching of the Si–O bonds, which are perpendicular to the surface and are parts of the Si–O–Ti linkages). The Ti-silicate film (with a Ti/Si molar ratio of 1) consists of a monolayer of corner-sharing [SiO_4_] tetrahedra on top of a monolayer formed by [TiO_6_] octahedra (Figure 33b). The top and bottom layers are connected by shared oxygen atoms at the corners of the [SiO_4_] tetrahedra and [TiO_6_] octahedra. In addition, the [TiO_6_] octahedra connect directly to the Ru substrate via oxygen atoms shared by two Ti atoms and one Ru atom (i.e., the Ti–O–Ru linkages).

The proposed structure for Ti-silicate has apparent similarities to phyllosilicates. A typical example is a talc [Mg_3_Si_4_O_10_(OH)_2_], which consists of two tetrahedral (T) [Si_2_O_5_^2–^] sheets with Mg^2+^ ions, together with OH^–^, sandwiched between sheets in octahedral sites (O), resulting in a “T–O–T” layered structure.^205^ In Ti-silicate/Ru(0001), the Ti^4+^ ions are coordinated octahedrally [TiO_6_], forming the O-layer. Instead of OH^–^ ions present in talc, the remaining corners of the [TiO_6_] are filled by oxygen atoms bound to the Ru substrate. Therefore, the Ti-silicate film can be viewed as a “T–O” layered structure chemically connected to a metal support.

#### 3.1.4 Ni-Silicate/Ni_*x*_Pd_1–*x*_(111)

We recall that highly crystalline BL silica films can be grown on Ni_*x*_Pd_1–*x*_(111) alloy surfaces with a continuously tunable lattice constant.^103^ Altman and co-workers further showed that the reactions of Si and O with the Ni_*x*_Pd_1–*x*_(111) alloy surface would extract Ni atoms from the substrate to form 2D Ni-silicates.^8,105^

##### 3.1.4.1 Reaction with an Alloy Substrate

A thermodynamically stable 2D Ni-silicate can be prepared by depositing one monolayer equivalent Si onto a Ni_0.26_Pd_0.74_(111) alloy substrate at ∼10^–6^ Torr O_2_ pressure followed by annealing at 950 K in the same gas environment. Using this procedure, the Ni atoms from the alloy substrate were segregated at the interface and incorporated into the framework during the annealing procedures.^105^ Similar to the Fe- and Ti-silicates on Ru(0001), the favored structure of Ni-silicate on alloy surfaces is described as a layer of 6-membered rings constructed from the corner-sharing [SiO_4_] tetrahedra, sitting on top of an octahedrally coordinated Ni–O layer. The 2D Ni-silicate is chemically bonded to the alloy substrate via this Ni–O layer, and it can transform into an incommensurate layer after relaxing to its favored lattice constant. However, it should be mentioned that the formation of Ni-silicate is energetically favored on Ni_*x*_Pd_1–*x*_(111), independent of the alloy compositions (for *x* ∼0–0.5).

Such Ni-silicates can also be classified as dioctahedral silicates that contain transition metal ions.^206^ Generally, the allocation of the metal ions (or cations) at the octahedral sites of these silicates is greatly influenced by the charge balancing. For example, with Ni in an oxidation state of +II, these Ni^2+^ ions prefer to fill the octahedral sites under the tetrahedral [Si_2_O_5_^2–^] layer. The resulting structure of the bulk Ni-silicate can then be analogous to lizardite [Mg_3_Si_2_O_5_(OH)_4_]. For comparison, the 2D Ni-silicate grown on a metal substrate can be viewed as a single layer of a dehydrated clay, where the octahedrally coordinated Ni–O layer substitutes the O–H-terminated octahedral layer in the clay. The metal substrate serves as an electron reservoir for balancing the extra positive charge on Ni-silicate. Actually, the nominal compositions of all metal-supported 2D silicates can be expressed as M_4_Si_4_O_16_, where M = Ti, Fe, or Ni. (Here, the surface chemisorbed adsorbed oxygen atoms and substrate atoms are not included in this expression.) As inferred from this expression, the donation of electrons from the metal support to the 2D silicate is quite flexible. For example, it donates two electrons to the Ni-silicate (Ni^4+^) and nominally zero electrons to the Ti-silicate (Ti^4+^). It is important to mention the possibility of isolating the metal-supported 2D silicate from the metal substrate and restoring the VDW character of clays by hydrations (e.g., by breaking the Ni–O–substrate bonds and creating new Ni–O–H bonds), which has been demonstrated for the 2D-silica.^28,80^

##### 3.1.4.2 Tuning Phase Formations

As mentioned before, it is known that lattice mismatch and substrate interactions are essential in determining the phases of 2D silica and silicate on Ni_*x*_Pd_1–*x*_(111) alloy surfaces. Recently, Altman and co-workers further studied the growth competition between silica and Ni-silicate on this substrate by changing the essential growth parameters, i.e., substrate composition, silicon coverage, partial oxygen pressure, and annealing temperature.^8^ STM, LEED, and IRAS results showed that, for Si coverages up to 2 ML equivalent, at oxygen pressures of 10^–6^ Torr, as well as at annealing temperatures of 1000 K, only a Ni-silicate phase was formed. In contrast, the BL silica phase can only be obtained by decreasing the oxygen pressure and by restricting both the annealing temperature as well as the annealing time. Thus, the high reactivity of Ni toward oxygen impedes the formation of BL silica on the Ni–Pd alloy substrate.

**Figure 34:**
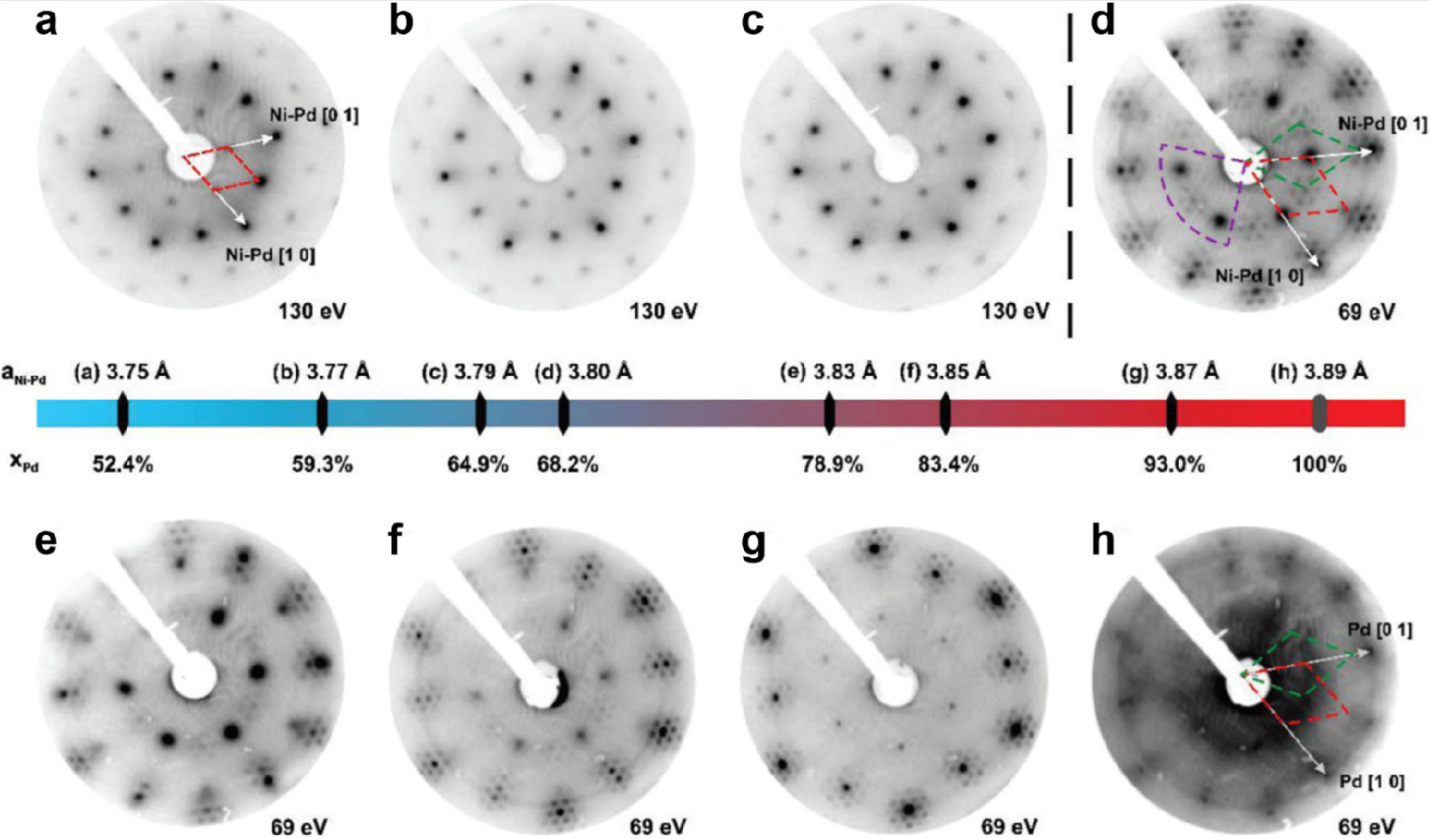
Commensurate–incommensurate transitions observed by LEED for the Ni-silicate on Ni_*x*_Pd_1–*x*_(111) alloy substrate with varied Pd concentrations between 52.4% and 100%. (a–c) Ni-silicate films with only commensurate crystalline phase. (d–g) Ni-silicate films with the coexistence of incommensurate and commensurate crystalline phases. (h) BL silica on Pd(111) with rotated and nonrotated incommensurate crystalline phases. The red and green dashed lines in panels a, d, and h indicate the unit cells of a nonrotated and rotated Ni-silicate overlayer, and the arrows show the primary directions of the Ni_*x*_Pd_1–*x*_(111) substrates. The lattice constants of the Ni_*x*_Pd_1–*x*_(111) substrates are also labeled on the color bar together with the Pd concentrations. Reproduced with permission from ref (8). Copyright 2019 Royal Society of Chemistry.

In addition, Altman and co-workers elucidated the influence of epitaxial strain on the Ni-silicate structure by varying the substrate alloy composition. Figure 34 shows a series of LEED patterns recorded for Ni-silicate/Ni_*x*_Pd_1–*x*_(111) with the Pd concentration varying between 52.4% and 100%. These results indicate that the lattice constant of the Ni-silicate overlayer can only be expanded between 1.12% and 1.40% before relaxing to its “natural” lattice constant despite the existence of chemical bonds with the alloy substrate. For lattice mismatches above 1.40%, incommensurate crystalline domains appear in the LEED pattern (Figure 34d). For comparison, the Ru(0001) substrate can impart a tensile strain of 2.1% to BL silica because of the much weaker VDW silica/Ru interactions. This result suggests that the 2D modulus,^99,207^ the accessibility of other phases,^99^ and the energy penalty for incommensuration^105^ are all critical factors in determining the epitaxial strain in Ni-silicate besides the film–substrate interaction. Although the energy cost of the incommensuration in Ni-silicate is modest,^105^ even longer annealing times and higher annealing temperatures do not transform Ni-silicate into its commensurate phase or into 2D-silica.

The experimental results were compared with DFT calculations, including first-principles atomistic thermodynamics. The obtained *ab initio* phase diagram clearly points toward the formation of 2D-silica for Si-rich and O-lean growth conditions. However, in contrast to the prediction of a thermodynamically stable 2D-silica phase, the experiments revealed disproportionation reactions of the 2D-silica into Ni-silicate and 3D-silica at high temperatures. Here, the limitations of the thermodynamic model, such as the assumption of the presence of a uniform surface freely exchanging atoms between reservoirs, may be responsible for the discrepancy. The present study demonstrates interesting possibilities of tuning the resulting phases and structures of 2D materials by varying the growth conditions as well as the composition of the solid solution substrate.

### 3.2 Hydroxylation of 2D-Silica

Hydroxylation is another way to modify 2D-silica chemically.^208^ It is well-known that the participation of silica in catalysis, where it may be used as either a support or as the active surface, is often determined by surface hydroxyl species. Generally, the surfaces of all naturally occurring and synthetically produced (from molecular precursors) silica are hydroxylated. Even UHV-cleaved silica surfaces can be immediately hydroxylated by dissociating residual water due to the presence of undervalent Si or highly strained siloxane.^209^ Numerous experimental and theoretical studies have been performed to understand the interactions between water and silica.^210−213^ Both isolated silanols (i.e., single silanols Si–OH, geminal silanols Si–(OH)_2_, and vicinal silanols HO–Si–O–Si–OH) and hydrogen-bonded silanols have been identified by various analytical tools.^210,214^

However, due to the structural complexity and diversity of 3D silica, the chemical properties of hydroxylated silica surfaces remain the subject of intensive investigations. Well-defined 2D-silica films prepared on metal surfaces represent new playgrounds for the mechanism studies of detailed hydroxylation processes, as well as for reactivity studies of different hydroxyls on silica.^215^

#### 3.2.1 Surface Hydroxyls on ML Silica/Ru(0001)

**Figure 35:**
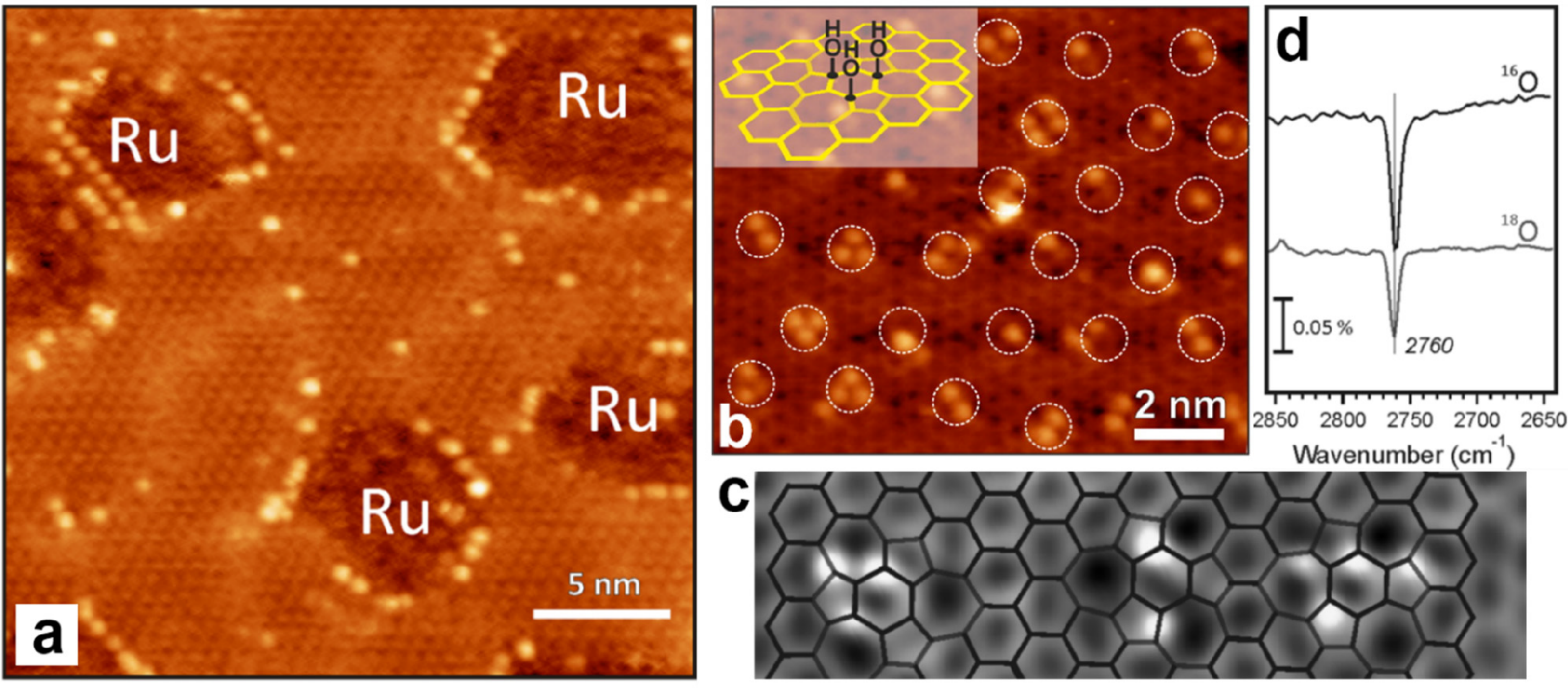
Surface hydroxyls on ML silica/Ru(0001). (a) STM image of the hydroxylated silica monolayer on Ru(0001) with Ru-exposing holes (*U*_s_ = 1.5 V, *I* = 0.1 nA). (b) STM image of the hydroxylated haeckelite-like silica monolayer (*U*_s_ = 1.3 V, *I* = 0.1 nA). (c) High-resolution STM image overlapped with polygonal representations showing the preferential locations of surface hydroxyls. (d) IRAS [*υ*(OD) region] of the haeckelite-like silica prepared with ^16^O_2_ (top spectrum) and ^18^O_2_ (bottom spectrum), which are both hydroxylated with D_2_^16^O. Reproduced with permission from refs (163 and 218). Copyright 2013–2014 American Chemical Society.

The surfaces terminated with siloxane groups (Si–O–Si) are usually hydrophobic. It was reported that water molecules exclusively bind via weak and nondissociative interaction on defect-free ML silica/Mo(112).^216,217^ The presence of defects in silica films considerably changes the water adsorption behavior, which leads to dissociative binding of hydroxyls. Step edges and domain boundaries are two types of commonly observed defects in epitaxially grown silica films. Yang et al. visualized the silanols on defective ML silica at the molecular level by STM as shown in Figure 35.^163,218^ The hydroxylation of ML silica/Ru(0001) was processed with water (D_2_O) vapor exposure at ∼100 K and by subsequently heating this ice-covered film to 300 K in UHV. Atomically sized protrusions with a height of ∼1–2 Å appear at film edges (Ru-exposing holes) and domain boundaries only upon hydroxylation (Figure 35a). Those protrusions are assigned to surface hydroxyls, as also inferred from the appearance of a band at 2760 cm^–1^ in IRAS. The shortest distance between these protrusions is close to the length of the silica unit cell (i.e., 5 Å), indicative of the isolated nature of these hydroxyls.

Based on defect-mediated hydroxylation, the spatial distribution of surface hydroxyls can then be tuned by modifying the defect structure of the silica films. As discussed in connection with Figure 12, arrays of structural defects (i.e., T-defect and R-defect) can be formed after annealing the as-prepared ML silica/Ru(0001) to 1100 K in UHV.^126^ For example, the T-defect with 3-fold symmetry resembles the haeckelite-like structures in graphene that is formed by three pentagons and three heptagons surrounding one hexagon.^125^ Hydroxylation of the haeckelite-like silica monolayer results in atomically sized protrusions and aggregates (mostly dimers and trimers) as shown in Figure 35b,c, which are arranged on the surface following the same long-range periodicity (∼24 Å) of the T-defects in haeckelite-like silica. High-resolution STM images reveal that hydroxyls are preferentially located above the Si atoms at the nodes formed by a pentagon, hexagon, and heptagon (5,6,7-sites). This result suggests that the hydronation may involve the breaking of the Si–O bond (i.e., the Si–O–Ru linkages) and subsequent flipping of the Si atom on top to bind the OH from the water.

Moreover, the isotopic experiments demonstrate that the hydroxyls exclusively stem from the adsorbed water molecules (Figure 35d). After hydroxylation with D_2_^16^O, only one ^16^OD stretching band (2760 cm^–1^) was observed for both silicas prepared with ^16^O_2_ and ^18^O_2_, indicating no scrambling with the lattice oxygen atoms. However, the fate of the second H from water remains puzzling and needs further investigation, in particular, by theoretical calculations.

It should also be noted that the silanols on both as-prepared and haeckelite-like silica monolayers are virtually identical except that, with respect to their thermal stabilities, silanols are stable up to ∼1050 K in the as-prepared silica as compared to ∼800 K in the haeckelite-like silica.

#### 3.2.2 Surface Hydroxyls on BL Silica/Ru(0001)

The surface of BL silica is also hydrophobic. By following the same “hydroxylation” procedure (i.e., D_2_O exposure at 100 K and heating to 300 K), as applied to the ML silica films, a sharp band centered at 2765 cm^–1^ was observed in IRAS, which is attributed to the formation of hydroxyls on the defect sites of BL silica/Ru(0001).^163^ It is noteworthy that the *υ*(OD) in ML silica/Ru(0001) is 2760 cm^–1^, indicating some effect from the chemical bonds between the silica and Ru substrate. In addition, a prominent shoulder that extends to 2700 cm^–1^ appears on the low-frequency side of the main OD band in BL silica, which originates from hydrogen-bonded OD species. Upon heating to elevated temperatures, the D-bonded OD species desorb first at ∼800 K, while the silanols are stable up to ∼1100 K, which is below the dihydroxylation temperature of powdered silica samples.^219^ The surface density of silanols was roughly estimated with an upper limit of ∼0.1 nm^–2^ as inferred from the integral intensity of the temperature-programmed desorption (TPD) signal for recombinative water desorption, corresponding to one silanol per every 40 6-membered rings by assuming a single crystalline structure of the silica BL.

##### 3.2.2.1 Electron Stimulated Hydroxylation

**Figure 36:**
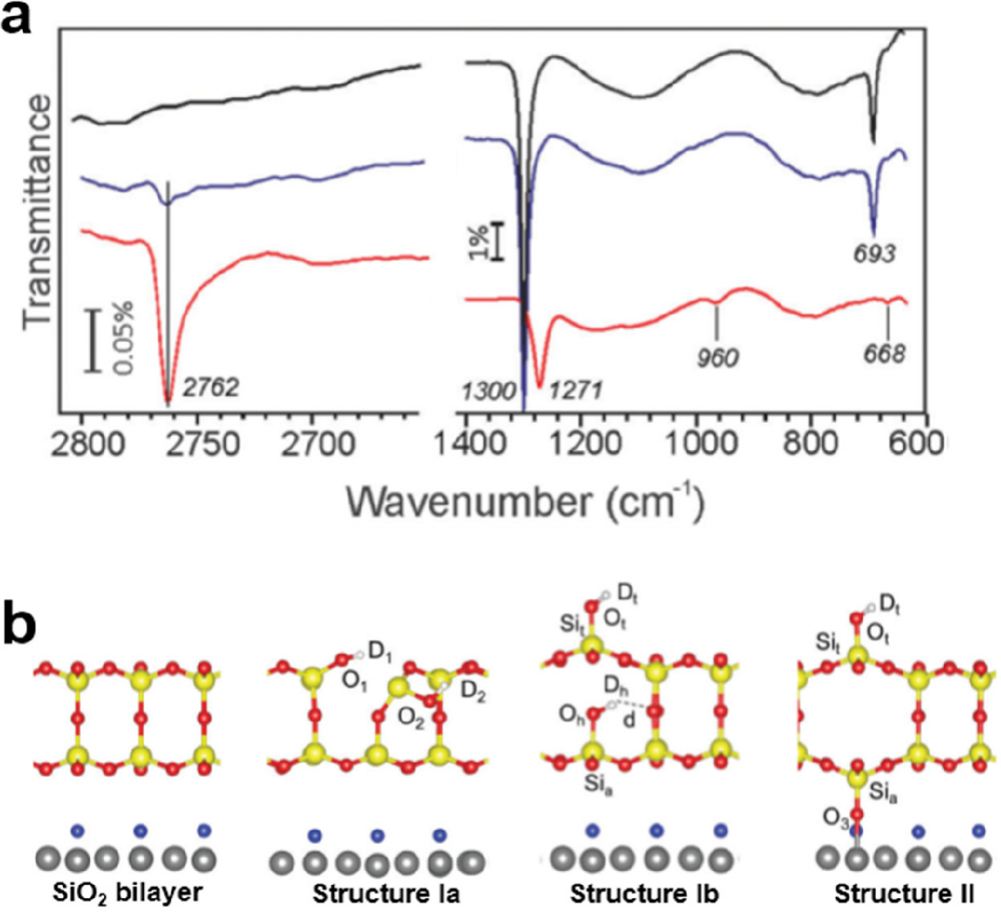
(a) IRAS of the pristine and electron stimulated hydroxylation of BL silica/Ru(0001). Black curve, as-grown silica; blue curve, silica exposed to D_2_O at 100 K and then flashed to 300 K; red curve, silica exposed to D_2_O at 100 K and irradiated with 150 eV electrons before the flash to 300 K. (b) Schematic side views of the pristine bilayer structure, hydroxylation **structure Ia**, hydronation **structure Ib**, and **structure II** that form a Si–O–Ru bridge to the Ru surface, respectively. Si, yellow; O(in silica), red; O(on Ru surface), blue; Ru, gray. Reproduced with permission from ref (220). Copyright 2016 Royal Society of Chemistry.

Yu et al. found that the silanol coverage can be significantly increased by low-energy electron irradiation of the ice-covered silica films.^220^ The irradiation parameters (beam energy, exposure time) and the ice thickness play essential roles in determining the degree of hydroxylation. For example, the silanol coverage has been increased to ∼15% with 150 eV electron irradiation treatments before flashing the ice-covered silica to 300 K in UHV. The considerable surface hydroxylation causes a strong attenuation and red-shift of the vertical Si–O–Si bands (1300 and 693 cm^–1^), as well as the appearance of new Si–O bands (960 cm^–1^) in IRAS (Figure 36a). The principal bilayer structure is maintained after electron stimulated hydroxylation, according to IRAS and STM results. The silanol species are very stable and only start to desorb above 1100 K. It should be noted that the intensity of the principal silica phonons cannot be fully recovered after dehydroxylation, indicating some destruction of the silica bilayer.

DFT calculations show that the hydroxylation can take place on two types of the Si–O–Si bonds, which are oriented either parallel (i.e., the topmost layer) or vertical (i.e., connecting two layers) to the surface (Figure 36b). In the case of breaking “in-plane” Si–O–Si bonds, two silanol species emerge on the surface (**structure Ia**), while in the case of breaking “vertical” Si–O–Si linkages, there is a vertical distortion of the upper Si out of the surface plane upon forming an OD (**structure Ib**). In addition to **structure Ia** and **structure Ib**, which are assumed for bulk silica surfaces, another structure, **structure II**, should be considered for metal-supported silica films. As shown in Figure 36b, **structure II** involves an inverted [SiO_4_] tetrahedron in the bottom layer, bonded to the Ru substrate via an O atom. Consequently, one hydrogen is produced, and the Ru surface is partially oxidized. According to the calculated hydroxylation energies, the relative stabilities of these three structures depend on the amount of chemisorbed O(H) atoms on the Ru surface.

However, the precise mechanism of electron stimulated hydroxylation remains to be established.^221,222^ According to the TPD results,^223^ it is most likely related to the radiolysis of water molecules in the ice layer. Nonetheless, the obtained hydroxylated silica surface can be further used for chemical reaction studies, such as for anchoring catalytically active species and their subsequent reactions.

##### 3.2.2.2 Mechanism of the Hydroxylation and Dissolution

**Figure 37:**
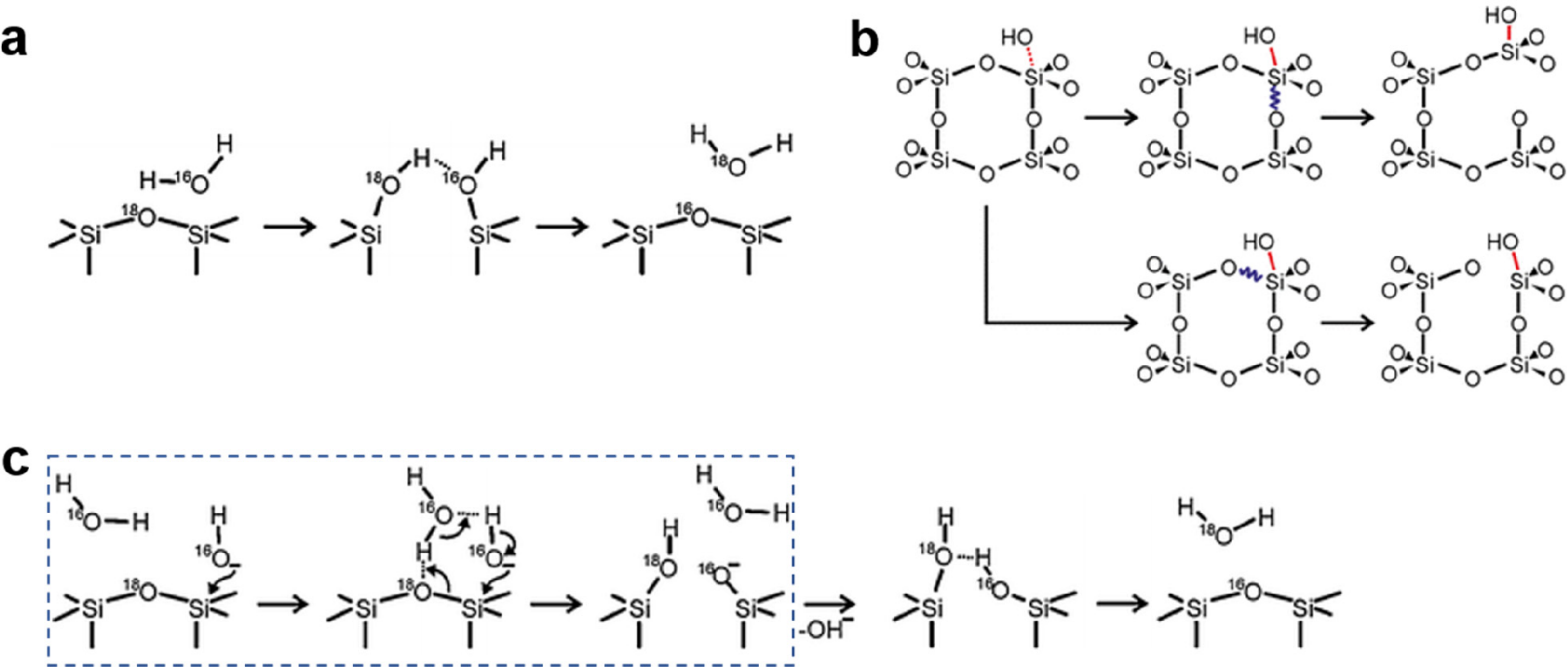
(a) Possible reaction pathway regarding the oxygen exchange between adsorbed water and silica. (b) Proposed mechanism for silica dissolution in high-pH aqueous conditions: either vertical (upper) or lateral (lower) siloxane bonds were broken following the hydroxylation by OH^–^. (c) Possible reaction pathway regarding the hydroxylation of the silica that involves a cyclic transition state (dashed rectangular) and subsequent steps that lead to oxygen exchange between adsorbed water and silica. Reproduced with permission from ref (223). Copyright 2017 The Authors. Published by Springer Nature.

It is generally accepted that hydroxylation of silica proceeds via the cleavage of siloxane bonds.^215,224^ Unlike the defect-caused hydroxylation, significant isotopic mixing occurs in the electron irradiation stimulated hydroxylation as shown in Figure 37a, which may be caused by opening and reforming siloxane bonds within the film.

The general mechanism of silica hydroxylation may also be based on a dissolution mechanism under aqueous conditions. The dissolution of silicates usually depends on one charged and one neutral species, involving the creation of hydroxyls at the expense of siloxanes. In an ice-covered BL silica/Ru(0001), aggressive agents such as hydroxide ions can be produced in the ice layer during electron bombardment, which will readily attack Si atoms to form silanol groups. As depicted in Figure 37b, either vertically or laterally oriented Si–O–Si bonds can be broken, followed by hydroxylation.^225^

In the presence of additional water molecules, the hydroxide ions (OH^–^) may activate the water molecule at the Si—O bonds in the siloxane bridge, resulting in water dissociation and formation via a cyclic transition state as shown in Figure 37c.^226^ Subsequent protonation of the ≡Si—O^–^ site and siloxane bridge reformation may also account for the isotope exchange (^16^O–^18^O) between water and silica.

##### 3.2.2.3 Acidity of the Hydroxylated Silica

The acidity of the hydroxyls on BL silica films can be estimated by the adsorption of weak and strong bases (e.g., CO and NH_3_). Taking CO adsorption as an example, the magnitude of the spectral shifts in both OH and CO stretching bands is a measure of the proton acidity once there forms an OH(OD)···CO adduct. Strong bases may even abstract a proton.^227^ It was found that continuous exposure of the hydroxylated silica films to ∼10^–5^ mbar CO at 300 K causes no changes in both position and intensity of the OD band at ∼2765 cm^–1^, suggesting that CO does not interact with the silanol on silica.^163^ For comparison, in the case of stronger base adsorption (NH_3_), it appeared to interact more strongly with surface hydroxyls, forming an OD···NH_3_ complex. In addition, the exchange reactions between hydroxyls and ammonia likely proceed via the same mechanism as that between the hydroxyls and water. According to the TPD and IRAS results, the desorption of ammonia from the OD···NH_3_ complex was found to be exclusively in the form of NDH_2_. Therefore, an upper limit approximation of the H–D exchange activation barrier can be assessed from the ammonia desorption energy, i.e., ∼37 kJ/mol.^163^ It is noteworthy that the hydroxylated ML silica/Ru(0001) showed the same behavior with respect to the H–D reaction with ammonia.

#### 3.2.3 Bridging Hydroxyls on Aluminosilicate/Ru(0001)

In contrast to pure silica films, where only a small amount of surface hydroxyls associated with defect sites were observed by IRAS, unambiguous bridging hydroxyls [with the OH_br_ (OD_br_) band at 3594 (2652) cm^–1^] were straightforwardly produced on aluminosilicate films (Al_0.4_Si_0.6_O_2_) after a similar hydroxylation process due to the charge imbalances caused by Al^3+^ ion incorporation (see Figure 29c).

##### 3.2.3.1 Acidity of the 2D Zeolite

The acidic properties of bridging hydroxyls can also be examined by the adsorption of different probe molecules. As already mentioned in Figure 29c, CO molecules can bind to the bridging hydroxyls on aluminosilicate/Ru(0001), which induces considerable red-shifts of the OH_br_ (OD_br_) stretching bands by 379 (243) cm^–1^. In parallel, the CO stretching band blue-shifts by 40 cm^–1^ with respect to the gas-phase CO molecule. These results largely differ from the hydroxylated silica films (section 3.2.2.3), indicating much higher reactivities of these bridging hydroxyls.

**Figure 38:**
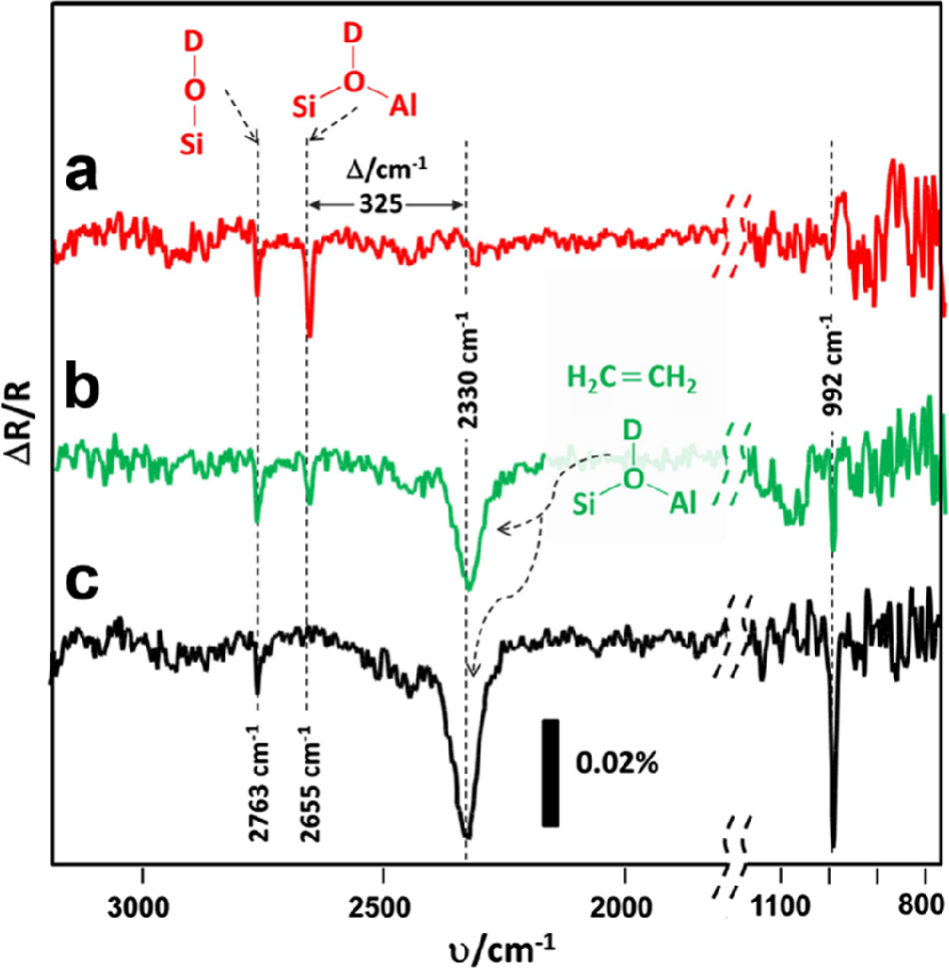
IRAS of an aluminosilicate film with bridging hydroxyls (OD_br_, 2655 cm^–1^) and surface silanol (Si-OD, 2763 cm^–1^) (a) before dosing C_2_H_4_ and (b, c) after increasing doses of C_2_H_4_. The CH wagging mode (992 cm^–1^) from C_2_H_4_ is also evident in the spectra. Reproduced with permission from ref (229). Copyright 2013 American Chemical Society.

Another weak base usually used as a probe molecule is ethene (C_2_H_4_). As shown in Figure 38, the adsorption of C_2_H_4_ on bridging hydroxyls induced a similar red-shift in the OD_br_ stretching band, i.e., from 2655 to 2330 cm^–1^. The broadening and increase in intensity upon formation of the OD_br_···C_2_H_4_ adduct rae common to H-bonded complexes and have also been observed in zeolites.^228^ It is noteworthy that this aluminosilicate film also contains surface silanol (Si-OD, 2763 cm^–1^) originating from surface defects, which allows us to directly compare the acidities of two different types of OD groups. As evidenced by Figure 38b,c, the Si-OD group stays intact with increasing doses of C_2_H_4_ due to its low acidic character.

**Figure 39:**
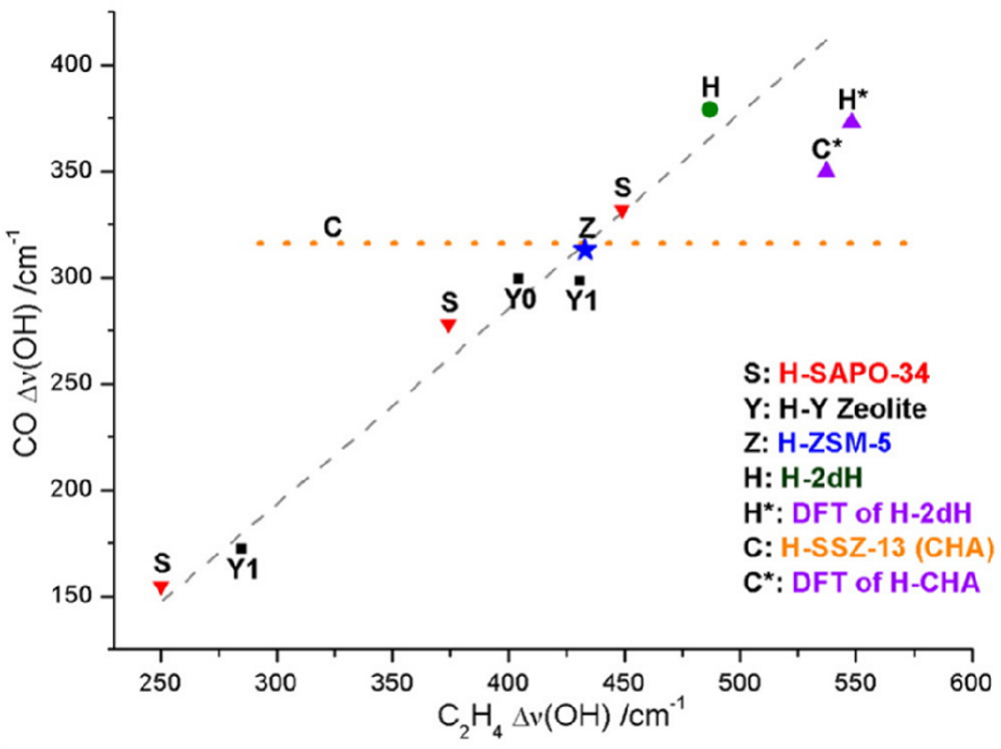
Plot of shifts in the OH band induced by C_2_H_4_ (*x*-axis) and CO (*y*-axis) adsorptions for a variety of zeolites and zeotypes, including the 2D-aluminosilicate film (green circle). Reproduced with permission from ref (229). Copyright 2013 American Chemical Society.

Weak bases such as CO and C_2_H_4_ form complexes with the proton of the bridging hydroxyl group without breaking the O–H bond. Generally, there is a strong dependence of the catalytic activity of zeolite with the acidity of the bridging hydroxy.^230,231^ Specifically, larger frequency shifts in bridging hydroxyl have been correlated to the higher acidity and catalytic activity. Figure 39 shows a plot of *Δν*(OH) shifts induced by CO and C_2_H_4_ for a variety of well-defined zeolites and zeotypes taken from the literature as well as the 2D-aluminosilicate films (referred to as H-2dH).^229^ The results clearly demonstrate that the acidity of the OH species formed on aluminosilicate/Ru(0001) is among the highest reported for zeolite. Therefore, it can be used as a model system for mechanistic studies of the large number of chemical reactions that are performed on zeolites.

In contrast, adsorption of strong bases such as ammonia and pyridine will abstract the proton from the bridging hydroxyl to form ammonium and pyridinium ions, respectively.

##### 3.2.3.2 Effect of the Al/Si Ratio

DFT calculations were performed to further study the acidity of the 2D zeolite by constructing different H_*n*_Al_*n*_Si_64–*n*_O_128_ cells with increasing Al/Si ratio, namely, 1/63 (*n* = 1), 1/7 (*n* = 8), and 1/3 (*n* = 16).^232^ The deprotonation energy of a zeolite is calculated as the energy difference between the deprotonated and the protonated zeolite. It was found that the deprotonation energy of the 2D zeolite increases with increasing Al/Si ratio, suggesting that acidity of the bridging hydroxyl (Brønsted site) is governed by its local environment, i.e., the number of [AlO_4_] tetrahedra in the second coordination sphere of the acidic site,^233^ which is in agreement with several experimental and theoretical studies.^233,234^

An increase in the Al/Si ratio will also decrease the Al–O bond length and the Al–O–Si angle in 2D zeolites. However, the changes in these geometric structures do not affect the relaxation energy of the anion, suggesting that the decrease of the acidity with a concomitant increase of the Al content is caused mainly by the changes in the electronic structure of the 2D zeolite. It is important to note that the 2D zeolite model (aluminosilicate/Ru(0001)) is very different from the real zeolites, where typical Al/Si ratios are significantly lower. This difference is because Al at low contents preferentially occupies the sites in the bottom layer for effective charge compensation from the metal support.

##### 3.2.3.3 Effect of the Surface Curvature and Film Thickness

DFT calculations were also used to estimate the influence of surface curvature on the acidity of the bridging hydroxyls by computing adsorption energies. It was found that the adsorption energies of weak bases are larger in cavities (e.g., OH_br_ in H-chabazite) than the ones in the planar case (e.g., OH_br_ in H-2dH) because of the larger dispersion contributions for curved surfaces.^229^ In the planar system, the Si–O–Al angles are closer to 180° than in H-chabazite, and the corresponding strain probably induces a weaker O–H bond and hence increases the acidity of H-2dH.

The calculated dielectric constant of the H-2dH is relatively small, and it depends on the film thickness and the distance of the charge from the surface. Based on the study of thickness-dependent deprotonation energies for thin H-MFI films, it was predicted that the acidity of the surface Brønsted sites increases with decreasing film thickness.^232,235^ As compared to bulk systems, such as H-chabazite, the very low deprotonation energy of the H-2dH can be attributed to the small dielectric constant of ultrathin dielectrics immersed in a vacuum, which leads to better stabilization of the charge created upon deprotonation.

However, it should be mentioned that the deprotonation energy is not a suitable reactivity parameter for solid acids.^236^ For example, whereas the adsorption energies of strong bases (e.g., NH_3_) are about the same in 2D and 3D systems, the deprotonation energies are much lower for 2D systems than for 3D systems. The difference between deprotonation energy and adsorption energy is due to the interaction of the NH_4_^+^ cation (formed by the protonation of NH_3_) with the negatively charged surface site.

### 3.3 Engineering the Interfacial Energetics at 2D-Silica/Metal Heterojunctions

Besides the direct metal doping and surface hydroxylation of the 2D-silica framework, the interface engineering at the 2D-silica/metal heterojunctions offers a different approach to tuning the properties of 2D-silica films. As discussed in section 2, the silica bilayer interacts weakly with the metal support via van der Waals forces, and oxygen molecules can intercalate and chemisorb at the interface. The amount of these surface chemisorbed oxygen molecules can be reversibly controlled by vacuum annealing and oxidation. The electronic properties of the silica/metal systems, therefore, can be regulated without altering the atomic structure of the 2D-silica.^133,134,237,238^

#### 3.3.1 Energy Level Shifts at the Silica/Ru(0001) Heterojunction

Considerable attention is paid to the effect of interfacial electronic structures on catalytic performance. For example, the dynamic surface potential barrier was demonstrated to be a rational descriptor for catalytic selectivity under oxidation reactions.^239^ As a model system, BL silica/Ru(0001) offers a new playground to study energetics at a weakly interacting oxide/metal interface. Wlodarczyk et al. have discovered the electronic state tuning at the silica/Ru(0001) interface through the addition or reduction of chemisorbed oxygen on Ru(0001) substrate.^133^ Wang et al. later reported that the surface and interfacial charge transfer-induced dipoles dominate the energy level alignment at the silica/Ru(0001) interfaces and the core-level binding energies in the silica films.^134^

Three mechanisms may contribute to the formation of surface or interface dipole moments at the silica/O–Ru(0001) heterojunction. The first one is the “push-back” effect,^240^ where the Pauli repulsion due to the silica film suppresses the tail of the Ru surface electron density that spills out into a vacuum. The second one is the charge redistributions caused by chemisorbed O atoms via Ru–O hybridization, referred to as surface charge transfer. The third one is electron tunneling from the silica (p orbitals of the bottom O layer in silica) to the Ru support (Ru *d*-bands), referred to as interface charge transfer. The dominating contributions arise from the last two mechanisms, which induce negative surface dipole moments (*p*_sur_) and positive interface dipole moments (*p*_inter_) along *z*. The competition between these two dipole moments depends on the amount of chemisorbed oxygen. Experimentally, considerable core-level (0.75 eV) and work function (WF) shifts (1.10 eV) were observed in the silica films upon decreasing the coverage of chemisorbed oxygen from 0.42 to 0.06 ML, supporting the importance of both the surface and interface dipoles at the silica/Ru(0001) heterojunction.^134^

**Figure 40:**
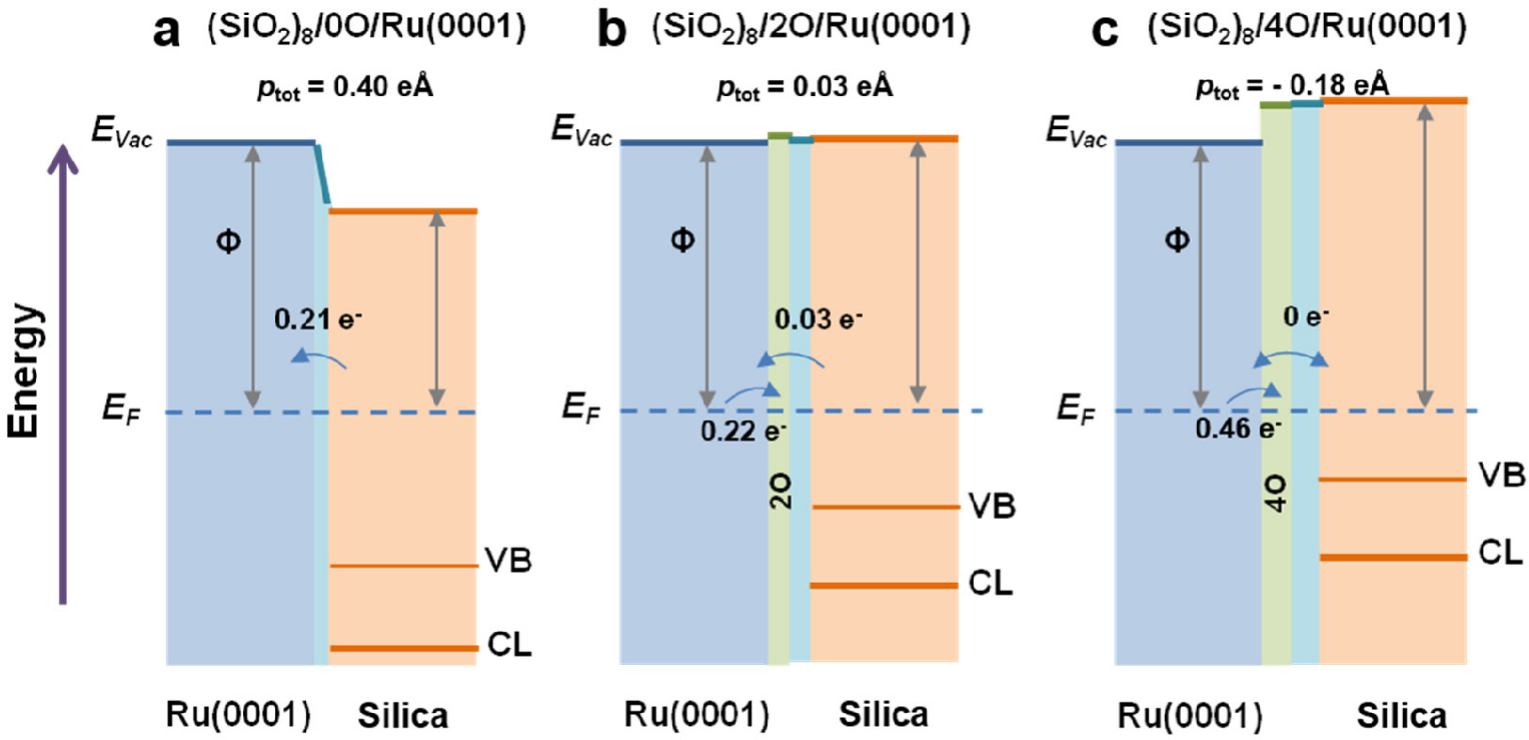
Interfacial chemisorbed oxygen-dependent energy level shifts at the silica/Ru(0001) heterojunction. Reproduced with permission from ref (134). Copyright 2016 Springer Nature.

DFT has been applied to study (SiO_2_)_8_/*n*O/Ru(0001) models, where *n* = 0, 2, or 4 and corresponds to 0, 0.25, or 0.50 ML chemisorbed oxygen, respectively.^133,134^ It was found that the interface distance [*d*(Ru–O_si_)] increases from 2.84 Å (*n* = 0) to 3.84 Å (*n* = 4). The magnitude of the surface charge transfer and interface charge transfer per unit cell (Δ*q*) was calculated by integrating the plane-averaged charge density differences (Δρ) along *z*, respectively. As the silica film is pushed away from the substrate, one expects an exponential decay of tunneling electrons. At *n* = 0, the dominating factor is the interfacial charge transfer with Δ*q*_inter_ = 0.21*e* and *p*_inter_ = 0.40 e Å. The interface dipole moment (*p*_inter_) causes the WF to decrease by 1.24 eV compared to Ru(0001) as shown in Figure 40a. With the increasing oxygen coverage (*n* = 2), Δ*q*_inter_ and *p*_inter_ decrease to 0.03*e* and 0.06 e Å. On the other hand, surface charge transfer starts to show an impact with Δ*q*_sur_ = 0.22*e* and *p*_sur_ = −0.03 e Å, thus resulting in a net dipole moment (*p*_tot_) of 0.03 e Å at the (SiO_2_)_8_/2O/Ru(0001) heterojunction (Figure 40b). At *n* = 4, surface charge transfer turns out to be a dominating factor, i.e., Δ*q*_inter_ and *p*_inter_ being negligible, while Δ*q*_inter_ and *p*_inter_ further increase to 0.46*e* and −0.18 e Å, respectively. The net dipole moment (*p*_tot_) is −0.18 e Å at the (SiO_2_)_8_/4O/Ru(0001) heterojunction, leading to a WF increase by 0.81 eV (Figure 40c).

#### 3.3.2 Charge Rearrangement at the Aluminosilicate/Ru(0001) Heterojunction

The studies of interfacial electronic properties were also extended to the case of bilayer aluminosilicate/Ru(0001), which is particularly important for catalysis as a zeolite model. Similar to the silica/Ru(0001), the energy level alignment at the aluminosilicate/Ru(0001) heterojunction is also determined by surface and interface dipole moments. The magnitude of these dipole moments may be modified by the aluminum concentration and the surface oxygen coverages on Ru(0001). However, the substitution of the Si^4+^ by Al^3+^ in the bottom layer of the BL structure will cause a charge transfer from the substrate to the film. The shorter film–substrate distance due to the electrostatic attraction makes the chemisorption of surface oxygen not as easy as the silica/Ru.^241,242^

**Figure 41:**
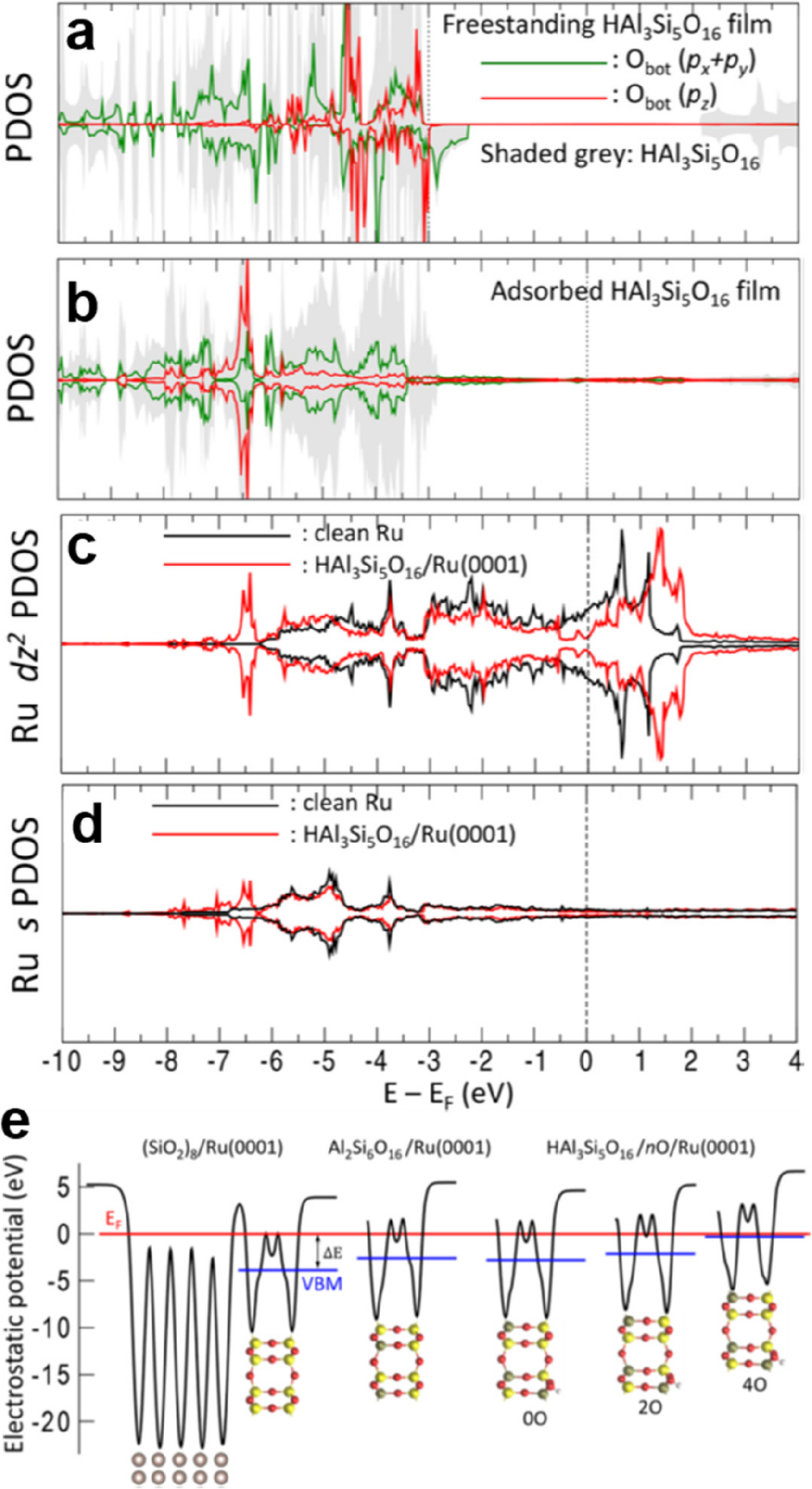
(a, b) Projected density of states (PDOS) of p_*x*_, p_*y*_, and p_*z*_ orbitals of O_bot_ from the freestanding HAl_3_Si_5_O_16_ and adsorbed HAl_3_Si_5_O_16_/Ru(0001), respectively. A shaded gray area represents the PDOS of the HAl_3_Si_5_O_16_. (c, d) PDOS of d_*z*_^2^ and s orbitals of Ru atoms under O_bot_ (Ru_O_) from a clean Ru(0001) surface and HAl_3_Si_5_O_16_/Ru(0001), respectively. (e) Electrostatic potential of (SiO_2_)_8_/Ru(0001), Al_2_Si_6_O_16_/Ru(0001), HAl_3_Si_5_O_16_/Ru(0001), HAl_3_Si_5_O_16_/2O/Ru(0001), and HAl_3_Si_5_O_16_/4O/Ru(0001). Fermi level (*E*_F_) and valence band maximum (VBM) are indicated by red and blue lines, respectively. Reproduced with permission from ref (238). Copyright 2019 American Chemical Society.

The aluminosilicate/Ru(0001) heterojunction is modeled by HAl_3_Si_5_O_16_/Ru(0001), where 50% of the bottom layer Si atoms and 25% of the top layer Si atoms are substituted by Al atoms, which corresponds to H_0.125_Al_0.375_Si_0.625_O_2_/Ru(0001) in experiments.^10,238^ The interface distance [*d*(Ru–O_bot_)] of this heterojunction is 2.23 Å. The major charge transfer arises from the d_*z*_^2^ and s orbitals of Ru to the p_*x*_ and p_*y*_ orbitals of O_bot_ (Figure 41a–d), leading to a net charge transfer of 1.27*e* per unit cell. In comparison, there is 0.21*e* transferred from the silica to the substrate at the SiO_2_/Ru(0001) interface (Table 2). Such a difference results in lower O 1s core-level binding energies (∼0.7 eV) in aluminosilicate films as compared to silica films at similar oxygen coverage (O_Ru_).^134^

**Table 2:** Interface Distance [*d*(Ru–O_bot_) in Å], Amount of the Interfacial and Surface Charge Transferred Electrons (Δ*q* in *e*^*–*^), Dipole Moment (*p* in e Å), and Work Function (Φ in eV) of (SiO_2_)_8_/*n*O/Ru(0001), Al_2_Si_6_O_16_/Ru(0001), and HAl_3_Si_5_O_16_/*n*O/Ru(0001), Where *n* = 0, 2, and 4^134,238^

	interface distance [*d*(Ru–O_bot_)]	interfacial charge transfer (Δ*q*_inter_)	surface charge transfer (Δ*q*_sur_)	interface dipole moment (*p*_inter_)	surface dipole moment (*p*_sur_)	bilayer dipole moment (*p*_bialyer_)	net dipole moment (*p*_tot_)	work function (Φ)
(SiO_2_)_8_Ru(0001)	2.84	0.21		0.40			0.40	3.88
(SiO_2_)_8_2O/Ru(0001)	3.65	0.03	0.22	0.06	–0.03		0.03	5.16
(SiO_2_)_8_/4O/Ru(0001)	3.84	0	0.46	0	–0.18		–0.18	5.93
Al_2_Si_6_O_16_/Ru(0001)	2.22	1.25		–0.28	0.02	0.21	–0.05	5.50
HAl_3_Si_5_O_16_/Ru(0001)	2.23	1.27		–0.25	0.03	0.42	0.20	4.61
HAl_3_Si_5_O_16_/2O/Ru(0001)	2.29	1.04	1.74	–0.38	–0.01	0.41	0.02	5.24
HAl_3_Si_5_O_16_/4O/Ru(0001)	2.61	0.85	3.27	–0.74	–0.10	0.46	–0.38	6.69

The interface distance [*d*(Ru–O_bot_)] as well as the charge redistribution at the aluminosilicate/Ru(0001) strongly depend on both the aluminum concentration and the O_Ru_ coverages as displayed in Table 2. For HAl_3_Si_5_O_16_/*n*O/Ru(0001), as *n* increases from 0 to 2 to 4, there is an increase in *d*(Ru–O_bot_) that results in a decrease in the interfacial charge transfer from Ru to aluminosilicate. At the same time, O_Ru_ draws more electrons from the Ru substrate. Considering that the *p*_bilayer_ (HAl_3_Si_5_O_16_) is ∼0.4 e Å, the *p*_tot_ changes from positive (0.20 e Å) to negative (−0.38 e Å), which leads to electrostatic potential shifts of HAl_3_Si_5_O_16_/*n*O/Ru(0001) as shown in Figure 41e. These studies provide physical insights into the energy level alignment of the zeolite models, which may help to understand variations in the catalytic performance of the metal–zeolite systems from the viewpoint of the electronic properties.^239^

## 4 Single Atoms and Molecules on 2D-Silica

The ability to prepare well-defined 2D-silica systems with precisely controlled atomic structures and surface/interface properties opens new perspectives for studying the deposition of atoms and molecules on nanoporous materials. The pores may accommodate single atoms/ions, molecules, or clusters for conducting chemical reactions or acting as atomic/molecular sieves. The pore sizes control the sizes of species and thus the efficiency of processes involved. Crystalline 2D-silica films consist of 6-membered −Si–O– rings (∼5 Å in diameter) that provide openings to access the nanopores. The well-defined structure of 2D-silica films, and the ability to characterize them at the atomic level, lends itself to theoretical modeling of such systems and may allow detailed interpretation of chemical interactions in silica-based systems, which is difficult to achieve with many bulk silica systems. In this section, the adsorption of single metal atoms/clusters and molecules on various 2D-silica/metal systems will be discussed with this aim and in order to set up well-defined models of supported metal catalysts or molecular sieves.

### 4.1 Adsorption of Transition Metal Atoms (Fe, Cu, Pd, Ag, Pt, and Au)

The structure and properties of single metal atoms and clusters supported on silica surfaces are a topic of interest in surface chemistry, material science, and nanotechnology.^243^ The deposition of transition metal atoms on silicon dioxide has been studied for many catalytic applications.^244−247^ In particular, single-atom catalysts featuring unique reactivity are emerging as a new frontier in heterogeneous catalysis.^248^ Intense research efforts have been devoted to the optimizations of the electronic interactions between the isolated atoms and their host materials.^249,250^ Despite the growing interest in catalytically active metal/silica composites,^251^ challenges related to the stability and activity of these materials remain.^252^ One main reason is that the silica used in such studies is often amorphous with various defects, rendering its characterization challenging. The use of well-defined 2D-silica films thus has advantages over the commonly used amorphous material, as it allows the application of the powerful toolkit of surface science.

#### 4.1.1 Transition Metal Atoms on Monolayer Silica/Mo(112)

The elucidation of the atomic structure of ML silica films on Mo(112) allows both theoretical and experimental studies of silica-supported metal atoms and clusters. As the adsorption energy of a metal atom at the silica/Mo interface is considerably larger than that on the silica surface,^253^ there is a driving force for atoms adsorbed on the outermost surface to penetrate and pass the openings in the silica layer.

##### 4.1.1.1 Effects of the Electronic Structure on the Adsorption

**Figure 42:**
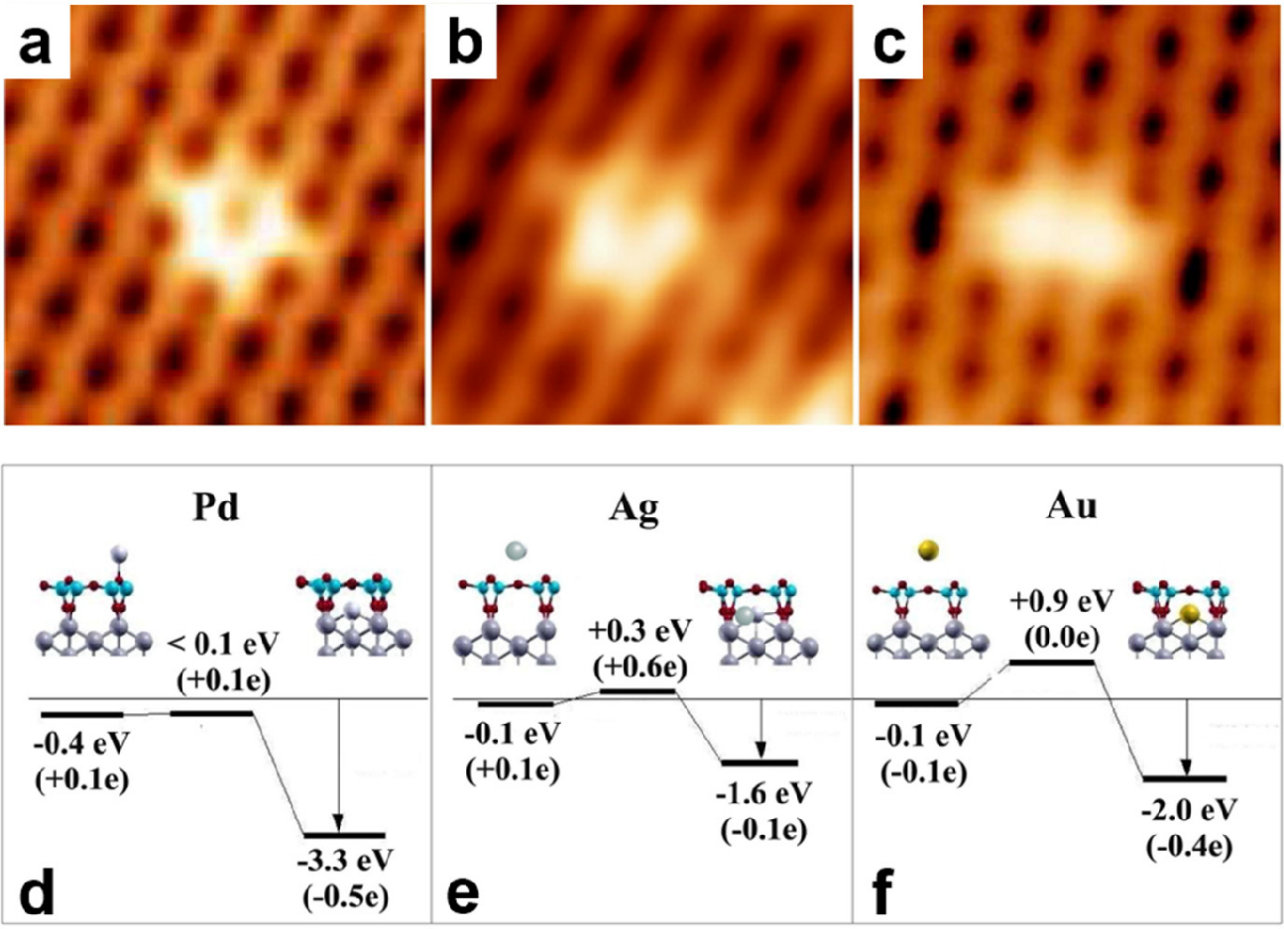
Closed-up STM images (5 × 5 nm^2^) for single atoms of (a) Pd (*U*_s_ = 0.5 V), (b) Ag (*U*_s_ = 0.3 V), and (c) Au (*U*_s_ = 0.8 V) adsorbed on silica/Mo(112). (d–f) Energy profiles for the adsorption of Pd, Ag, and Au atoms on the 6-membered ring of silica film. The values in each plot denote the atom binding energy on top of the film (left), the energy barrier for atom penetration (middle), and atom binding energy inside the film (right). All energies are given with respect to a gas-phase atom. The Bader charges during the adsorption are included in parentheses. The detailed binding configurations are shown in the insets (Mo, large gray spheres; Si, medium blue spheres; O, small red spheres). Reproduced with permission from ref (253). Copyright 2009 Elsevier B.V.

Intuitively, the relevant parameters governing the energy barrier for metal atoms to penetrate 2D-silica are connected to their size with respect to the silica pore diameter. As compared to the larger atoms, the smaller ones should exhibit a reduced barrier. Ulrich et al. showed that the barrier is determined by the electronic structure of the adsorbed metal atom, particularly by the spatial extent and electron filling of its valence orbitals.^253^ The adsorption of single Pd, Ag, and Au atoms on silica/Mo(112) was investigated by STM and compared to the results of DFT calculations. The three species are chosen for their comparable van der Waals radii (e.g., Pd ∼163 pm, Au ∼165 pm, and Ag ∼172 pm) and similar electronic properties (Ag and Au).^254^ As shown in Figure 42a, in a bias window of 0.5–1.5 V, there is a starlike appearance for the Pd on silica/Mo(112). It transforms into a bright protrusion above 2.0 V sample bias. The distinct contrast provides evidence for the binding of a Pd atom on a Mo bridge site at the interface.^255^ The embedded Pd atoms are randomly distributed on silica/Mo(112). They show no adsorption preference at the antiphase-domain-boundaries (APDB) (8-membered rings, see Figure 7b) compared to the 6-membered rings, indicating a similar penetration barrier. The same hexagonal stars appear for Ag adsorption, although in a slightly different bias regime (Figure 42b). In contrast to the Pd adatoms, Ag shows a preferred affinity to interact with the APDB with a probability 3–5 times higher than that of the 6-membered rings, suggesting a slightly lower penetration barrier at the APDB. In contrast, the Au atom shows a very different adsorption behavior, and it is entirely unable to penetrate the 6-membered rings and exclusively binds to the APDB. Figure 42c shows a single Au atom that has penetrated such an 8-membered ring, which may represent a critical nucleus for Au aggregation.

DFT calculations demonstrate that a Pd atom has a negligible energy barrier (below 0.05 eV) toward penetration and has a larger binding energy of 3.3 eV to interfacial Mo bridge sites (Figure 42d) as compared to adsorption at the top of the silica layer (0.4 eV). Once the Pd atoms are bound to the silica/Mo interface, there is a hybridization between the Pd 5s and O 2p orbitals of the silica; therefore, the Pd atom becomes partly negatively charged (−0.5*e*). The Ag atom experiences a higher penetration energy barrier at the 6-membered rings (0.3 eV), which needs to be overcome. After passing the pore, Ag binds to the silica/Mo interface with a substantially lower binding energy of 1.6 eV due to the low-lying Ag 4d states (Figure 42e). The calculated penetration barrier for Au increases to 0.9 eV on defect-free silica. The probability of reaching a high-binding site at the interface is limited exclusively to the APDB due to the presence of larger pores. A Au atom remains neutral above the silica, and it becomes partly negatively charged (−0.4*e*) at the interface due to the charge transfer from the Mo (Figure 42f). The imaging contrast of Au atoms bound to APDB primarily results from a structural distortion of the silica lattice upon Au adsorption.^255^

The penetration barrier can be related to the repulsion caused by the oxide charge density (i.e., the occupied O 2p states) on the incoming atom. Pd with an unoccupied valence s orbital produces only a small repulsion and, therefore, a low penetration barrier. Half-filled Ag 5s and Au 6s orbitals will strongly interact with the surface O 2p states during penetration. The substantially lower energy barrier for a Ag atom is caused by a transient positive charging of Ag atoms (+0.6*e* in Figure 42e) when passing the silica ring, which reduces the electron–electron interaction with the oxide states. By identifying this interaction mechanism, the penetration barriers for other atomic and molecular species can be predicted.

##### 4.1.1.2 Effect of Point Defects on Adsorption

The scenario may be different in the presence of surface defects. In silica/Mo(112) films, three major defects are expected, i.e., the extended defects (steps and kinks), line defects (antiphase-domain-boundaries), and point defects (vacancies). Regarding the deposition of metal atoms on defective silica, in most cases, metal clusters tend to nucleate on the terrace sites and along the domain boundaries.^256−258^

The adsorption structures and properties of Au atoms at point defects on the silica/Mo(112) surface have been studied by Martinez et al. based on periodic DFT calculations.^259^ Four point defects have been considered: (a) Nonbridging oxygen (NBO, ≡Si—O^●^) results from the rupture of a Si—O•••Mo bond and reversal of the ≡Si—O^●^ fragment orientation toward the vacuum. The ≡Si—O^●^ center will capture one electron from Mo to form a silanolate group, ≡Si—O^–^. (b) A Si dangling bond (E′ center, ≡Si^●^) results from the rupture of a Si•••O—Mo bond. The E′ center has a radical character and does not trap electronic charge coming from Mo. (c) An oxygen vacancy (V_O_, ≡Si—Si≡) may result from the displacement of an oxygen atom from the silica layer to the interface. (d) A peroxo group (≡Si—O—O—Si≡) can be formed by the addition of an oxygen atom to the silica layer. The nature of these defects in bulk silica has been studied intensively.^260^ However, the computational results show that only the NBO defect, as well as the V_O_ defect, is likely to form on the silica/Mo(112) surface under the applied experimental conditions. The E′ center tends to recombine with an interface oxygen atom to form an undefective structure, while the peroxo group is unstable since the additional oxygen atom prefers to bind at the interface with Mo rather than being included in the silica lattice.

**Figure 43:**
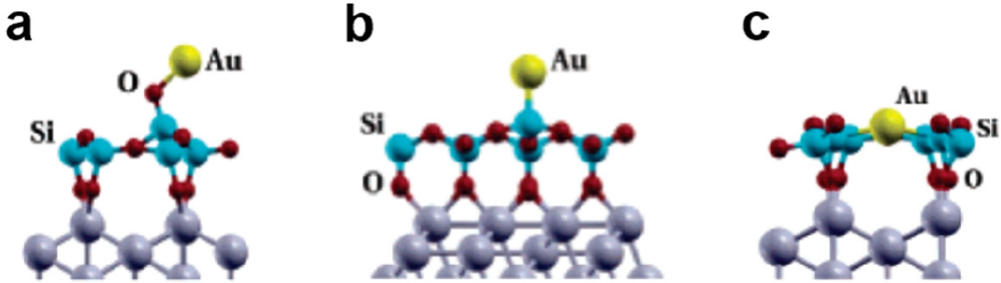
Geometric structures of an adsorbed Au atom on different defect points of silica/Mo(112). (a) On a ≡Si—O^–^ defect. (b) On a ≡Si^●^ defect. (c) On a ≡Si—Si≡ defect. Reproduced with permission from ref (259). Copyright 2006 American Chemical Society.

The adsorption interaction between a Au atom and defect-free silica is very weak. Only specific defect sites, such as the APDB, can stabilize these adsorbed Au atoms. Except for the unreactive peroxo group, the other defects bind strongly with the Au atom to form stable surface complexes (Figure 43a). For example, on an NBO defect, Au forms a neutral ≡Si—O—Au complex with a binding energy of 1.72 eV, while on an E′ center, it creates a strongly bound ≡Si—Au complex with a binding energy of 3.48 eV. The V_O_ defect acts as a gate where the Au atom can penetrate to bind efficiently at the silica/Mo surfaces. These results demonstrate that the defect-introduced new electronic states can be potentially involved in the interaction with adsorbed metal atoms.

##### 4.1.1.3 Effect of Doping on Adsorption

Besides the point defects, doping of the silica/Mo(112) films is another way to enhance the adsorption interactions between the metal adatoms and silica. Goodman and co-workers have demonstrated that the stability of silica-supported Au atoms can be significantly improved by doping the silica films with Ti or by forming TiO_2_ islands on the silica surface.^59,258^ Giordano et al. have further studied the adsorption of Au and Pd atoms on a Ti-doped silica/Mo(112) surface with periodic DFT calculations.^261^ It was found that Ti-doping is energetically favorable with an energy gain of 1.15 eV and does not lead to a significant distortion of the ML structure of silica. The Ti atoms remain bound to the Mo substrate via a Ti–O–Mo linkage.

**Figure 44:**
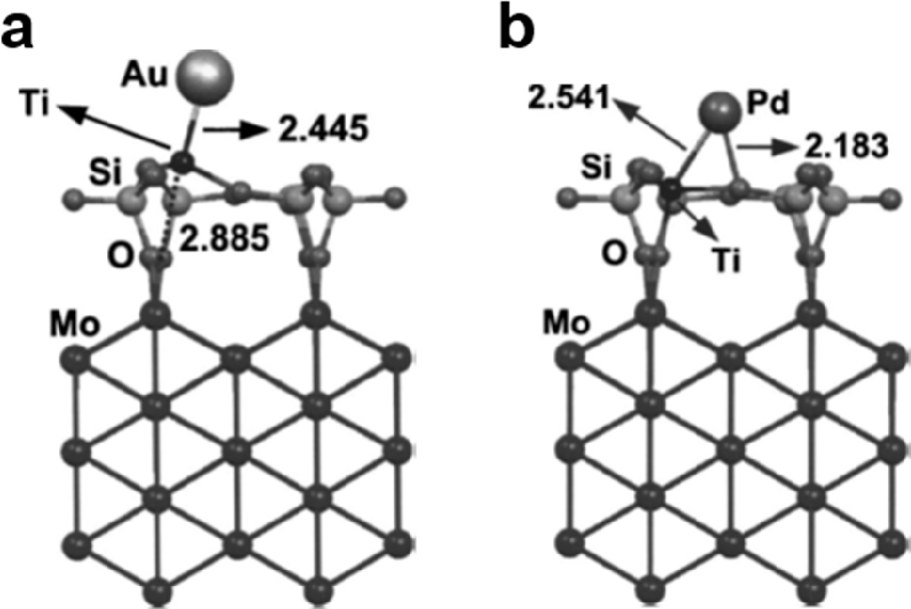
Structure of an adsorbed (a) Au atom and (b) Pd atom on Ti-doped silica/Mo(112) films. Distances are in Å. Reproduced with permission from ref (261). Copyright 2006 AIP Publishing.

The presence of Ti dopants induces low-lying empty levels with Ti 3d character, which may easily hybridize with filled orbitals of the adsorbed metal atoms. Therefore, a very different adsorption interaction occurs when Au atoms are deposited on Ti-doped silica/Mo(112). As shown in Figure 44a, the Ti•••O—Mo bond is broken. The Ti atom moves up toward the adsorbed Au atom, and the O atom moves down to bind strongly to Mo. The Ti atom remains four coordinated, by which the bond distance of Ti to Au is 2.445 Å, indicating a strong covalent bond with the Au atom. Such an anchored Au atom may act as a nucleation site for further growth of small gold clusters on silica. As a comparison, the adsorption of the Pd atom does not induce a dramatic structural rearrangement (Figure 44b). Pd interacts with both the Ti dopant and a bridging O atom.

The functionalization of the silica by the Ti dopant could be important for enhanced catalytic properties of the Au/silica system.^262^ Moreover, as discussed in section 3.1.1, Al-doped silica/Mo(112), i.e., aluminosilicate/Mo(112), is also expected to have similar anchoring properties for adatoms.

##### 4.1.1.4 Effect of Surface Oxygen on Adsorption

In section 2.1.1, it has been discussed that two kinds of silica films may be prepared on a Mo(112) substrate, i.e., “O-poor” silica/Mo(112) and “O-rich” silica/Mo(112).^87^ These two phases exhibit slightly different phonon frequencies of the Si–O–Mo asymmetric stretching band (1059 cm^–1^ vs 1050 cm^–1^).^110^ It was found that the adsorption of Pd atoms causes a red-shift of the Si–O–Mo asymmetric stretching band for both “O-poor” and “O-rich” silica/Mo(112) films. In particular, the calculation shows that the primary phonon frequency is red-shifted by 11 cm^–1^ for “O-poor” silica and by 23 cm^–1^ for “O-rich” silica. The red-shift is Pd-coverage dependent, although the Pd is not incorporated into the silica structure. These results indicate that the silica phonons are slightly perturbed by the presence of the Pd atoms at the interface.

The adsorption energy profiles for Pd adatoms on “O-poor” and “O-rich” silica/Mo(112) were obtained from DFT calculations. On “O-poor” silica/Mo(112), the estimated barrier for penetration of the Pd atom is negligible, and the adsorption energy at the interface is very large (3.3 eV as discussed in Figure 42d), where Pd interacts directly with the Mo substrate. In contrast, on “O-rich” silica/Mo(112), the Pd atom sits above the center of the ring with a small energy barrier that separates the Pd atom from being adsorbed at the interface.

**Figure 45:**
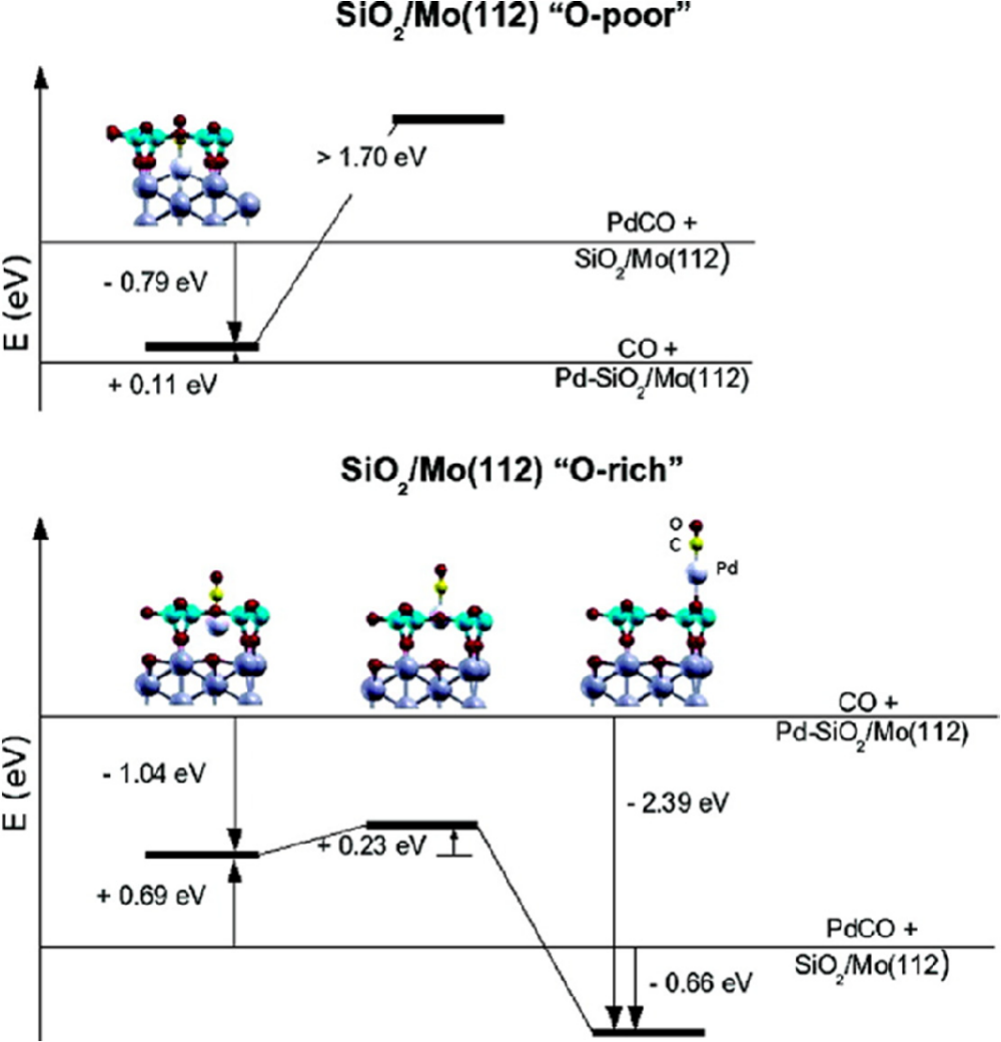
Energy profile of the interaction of CO with a Pd atom adsorbed on O-poor and O-rich ML silica/Mo(112) film. The structure of the silica and the position of the Pd–CO complex are shown in the insets (Mo, large gray spheres; Si, medium blue spheres; O, small red spheres; Pd, white spheres). Reproduced with permission from ref (264). Copyright 2008 American Chemical Society.

The interaction of Pd with the silica films was further investigated by using CO as a probe molecule (Figure 45). According to DFT calculations, CO is unbound to Pd atoms (+0.11 eV) that adsorb on “O-poor” silica. In contrast, CO binds strongly to Pd atoms (−1.04 eV), located in the cavity of the “O-rich” silica/Mo(112) interface. The formation of a strong Pd–CO bond weakens the adsorption interaction of Pd with the substrate. Therefore, the Pd–CO complex becomes unbound and can be pulled out from the hexagonal ring by overcoming a small barrier of 0.23 eV. Moreover, the Pd–CO complex is weakly bound to the silica surface and can diffuse and eventually aggregate with other Pd atoms or clusters. Such a CO-induced Pd sintering effect has been observed experimentally.^263^

##### 4.1.1.5 Effect of the Anchoring Sites on Adsorption

**Figure 46:**
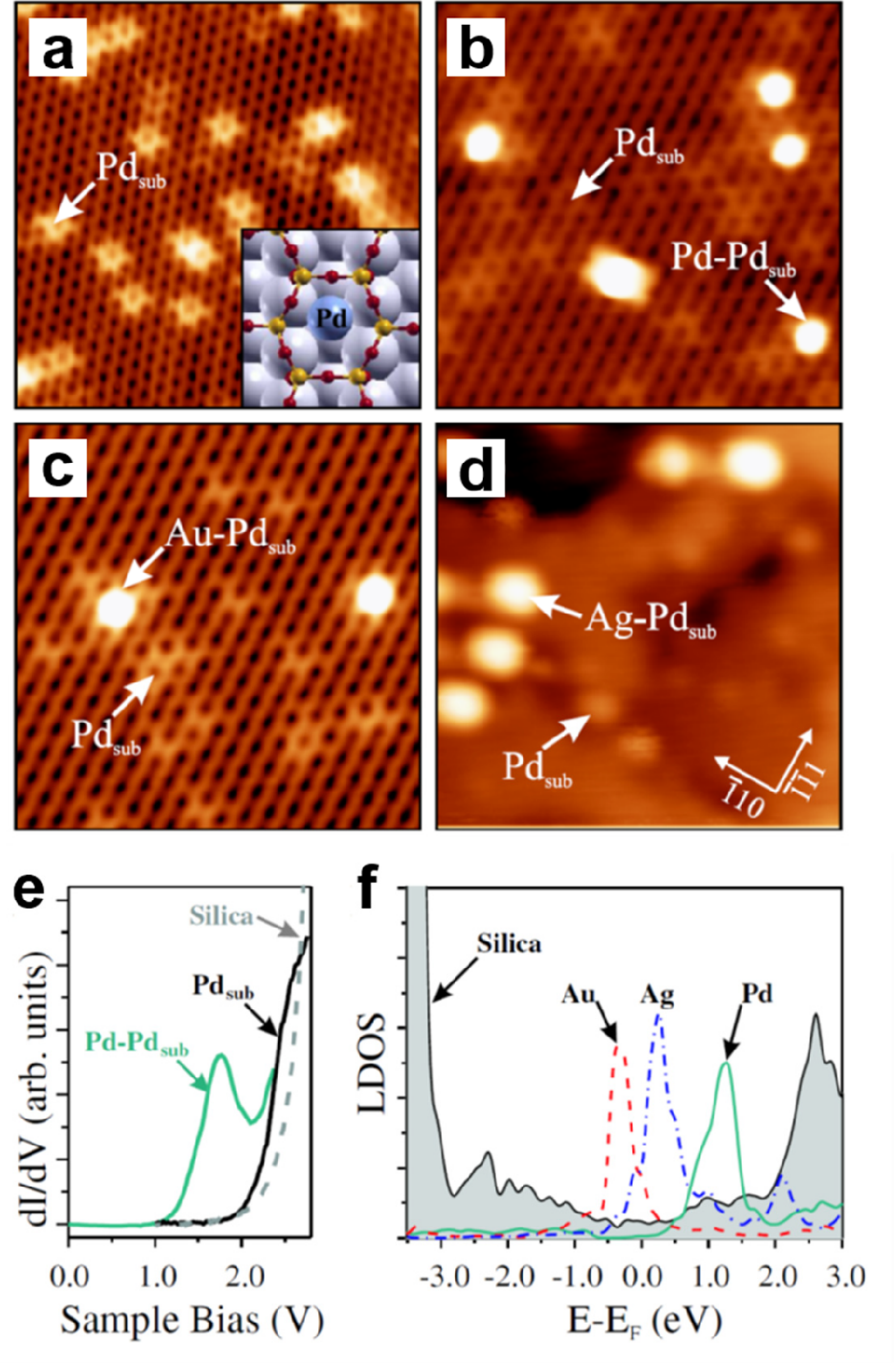
STM images showing the Pd_sub_ on silica/Mo(112) that acts as anchoring sites for Pd, Au, and Ag adatoms (14 × 14 nm^2^). (a) Pristine Pd_sub_ on silica/Mo(112) (*U*_s_ = 1.2 V). After deposition of single (b) Pd atoms (*U*_s_ = 0.5 V), (c) Au atoms (*U*_s_ = 0.5 V), and (d) Ag atoms (*U*_s_ = 1.9 V). (e) Experimental d*I*/d*V* spectra of a pristine Pd_sub_ and a Pd–Pd_sub_ complex. (f) Calculated DOS of silica/Mo(112) and respective adatom binding to a Pd_sub_ anchor. The line spectra show the contributions of Pd 5s, Ag 5s, and Au 6s to the hybrid states formed with the O 2p orbitals. Reproduced with permission from ref (265). Copyright 2009 The American Physical Society.

Like the defective silica or doped silica, the insertion of defined binding sites into the silica/Mo(112) can also be an approach toward a functionalized adsorption system. As discussed above, the adsorbed Pd atoms remain close to the surface and might be used as anchoring sites for adatoms that would not bind to the inert silica surfaces. As shown in Figure 46b–d, three sets of experiments are performed to assess the possibility of anchoring single atoms (Pd, Au, and Ag) to the inserted Pd species (referred to as Pd_sub_). In contrast to the starlike feature of Pd_sub_, the new adsorbed species are imaged as round protrusions at all sample biases, which are assigned to single atoms (Pd, Au, and Ag) bound to Pd_sub_ anchors on the silica film.

These adatoms form covalent bonds toward the Pd_sub_ with binding energies of 1.16 eV (Pd–Pd_sub_, 2.66 Å), 0.35 eV (Au–Pd_sub_, 2.54 Å), and 0.19 eV (Ag–Pd_sub_, 2.85 Å), respectively. Surprisingly, the binding energies follow an opposite trend for gas-phase dimers, where the corresponding binding energies are 0.7 eV for Pd–Pd (2.54 Å) and 1.4 eV for Au–Pd (2.68 Å), for example.^254^ The unexpected relation between short interatomic distances and low binding energies originates from two competing interaction mechanisms on the silica surface, i.e., the attractive covalent bond toward the adatoms–Pd_sub_ is counterbalanced by the Pauli repulsion exerted by the filled 2p states of the surface oxygen on the adatoms. The strength of the repulsion is controlled by the hybrid states formed between the adatoms and silica (O 2p) (Figure 46f). For example, the Pd 5s–O 2p hybrid state is located at +1.25 eV above the Fermi level and is thus empty. As a result, the binding energy of Pd–Pd_sub_ is even higher than in gas-phase Pd–Pd dimers, indicating the stabilization effect of the Mo support. In contrast, the Ag 5s–O 2p (+0.1 eV) and Au 6s–O 2p (−0.3 eV) hybrid states are singly and doubly occupied and induce substantial Pauli repulsions with the silica. This effect is more significant for Au due to its 6s^2^ configuration. However, the Au 5d states strengthen the Au–Pd_sub_ interaction more significantly than the Ag 4d state strengthens the Ag–Pd_sub_ interaction, resulting in higher binding energy in Au–Pd_sub_.

These anchored species can be easily removed from the silica surface by applying moderate STM tip pulses (3–5 V). It should also be mentioned that the approach discussed here by using anchoring sites (e.g., Pd) can be employed to produce even more complex structures, such as a functionalized adsorption system via subsequent anchoring of different atomic species.

##### 4.1.1.6 Stabilized Monomeric Iron Species

**Figure 47:**
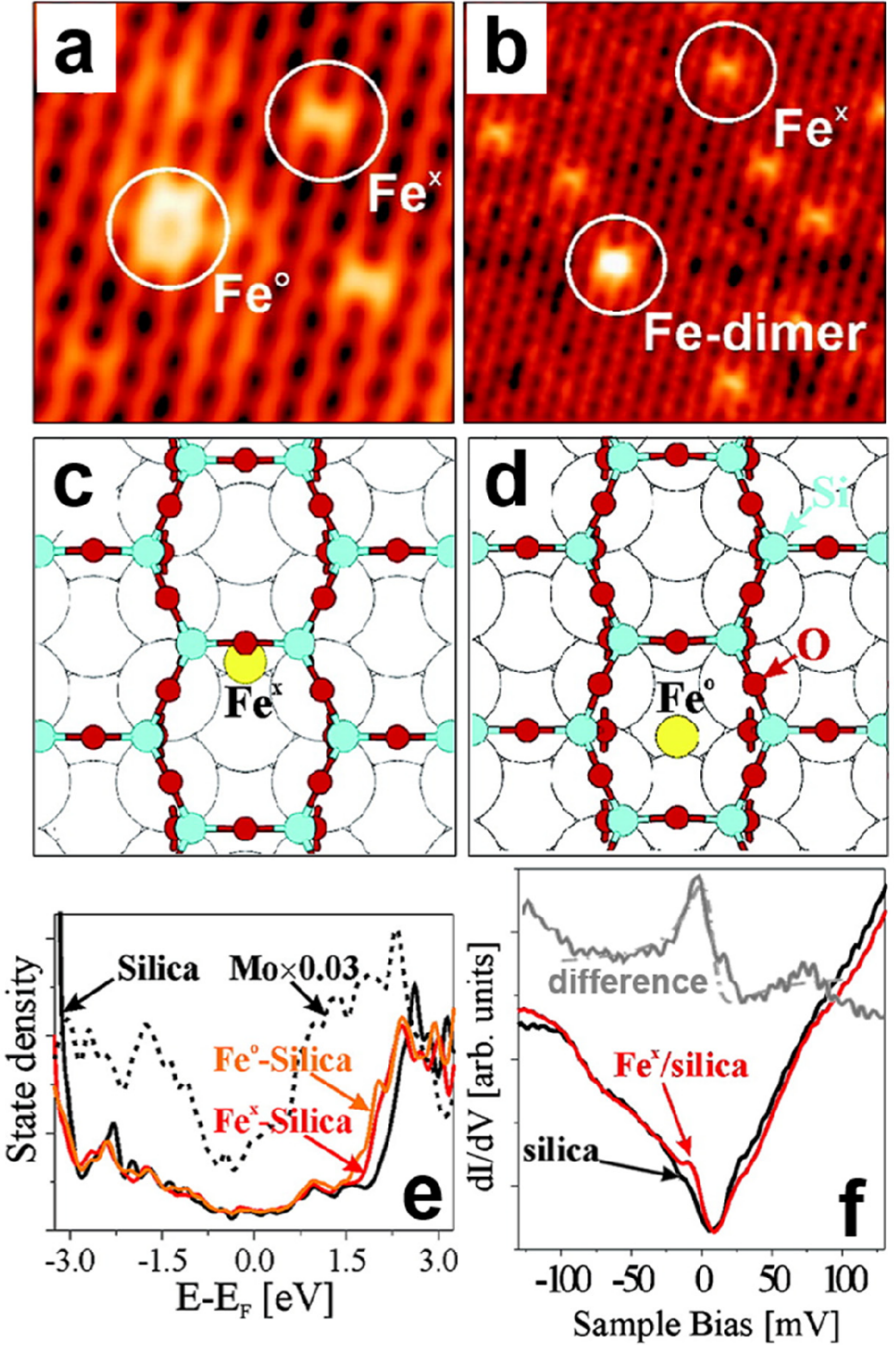
Fe atoms on silica/Mo(112). (a) Close-up STM image with subsurface Fe^o^ and Fe^x^ species (5 × 5 nm^2^, *U*_s_ = 0.5 V). (b) Close-up STM image with an Fe dimer and few Fe monomeric species (10 × 10 nm^2^, *U*_s_ = 0.5 V). (c, d) Structure model for Fe atoms in two different binding sites at the silica/Mo(112) interface, i.e., below a silica ring (Fe^o^) and below a [1̅10]-oriented Si–O–Si bridge (Fe^x^). (e) Calculated local density of states (LDOS) of silica/Mo(112) before and after Fe adsorption. (f) Differential conductance spectra of the silica/Mo (black) and a subsurface Fe^x^ species (red). The difference between the silica/Mo and the Fe^x^ conductance curve is shown in gray, and the peak at the Fermi level is fitted with the Fano model (dashed line). Reproduced with permission from ref (266). Copyright 2010 American Chemical Society.

In addition to Pd and Ag atoms, Fe atoms can also be embedded and stabilized at the silica/Mo(112) interfaces. The Fe embedment could possibly have an important future application because the nanopores in silica/Mo films may provide an interesting template to realize a storage device formed by an ensemble of magnetic atoms hosted in separate nanopores. The Fe-silica film system has been investigated by Jerratsch et al. using STM and DFT.^266^ As shown in Figure 47a,b, Fe atoms penetrate below the silica rings and occupy two different binding sites at the silica/Mo(112) interface. In the topographic images, the majority of the Fe atoms display an X-shaped protrusion, centered at the interaction of two silica rings with an orientation along the Mo[1̅10] direction (Figure 47c, denoted as Fe^x^). A minority of Fe atoms (∼10%) show ringlike protrusions, which display a higher intensity than the X-shaped ones (Figure 47d, denoted as Fe^o^). Similar to Ag adsorption, the calculated penetration barrier for both Fe species is 0.3 eV, which can be easily overcome by the thermal impact of the incoming atoms. Fe^x^ has a binding energy of 3.6 eV. In this location, the Fe 4s state hybridizes only with the Si–O–Si bridge. As a consequence, the subsurface Fe atom manifests itself with its characteristic X-shaped contrast in STM images. Fe^o^ has a smaller binding energy of 3.3 eV. Here, the Fe 4s orbital wave function overlaps with the Si and O orbitals of the silica ring, thereby locally increasing the unoccupied state density. The Fe species in both adsorption configurations are characterized by bearing a positive charge and by a strong covalent bonding to the Mo substrate. Moreover, they induce extra states around 2.1 eV above the Fermi level (Figure 47e). Larger structures were also observed on the surface, such as Fe-dimers. According to DFT calculations, both Fe^x^ and Fe^o^ species can anchor additional surface Fe atoms but with very different binding energies (e.g., 0.24 eV for Fe–Fe^x^ and 1.47 for Fe–Fe^o^). Therefore, the formation of Fe-dimers is restricted to the subsurface Fe^o^ at low Fe coverage.

STM differential conductance spectra, taken above the interfacial Fe species, display a typical Kondo feature, as illustrated in Figure 47f. Evidently, both Fe species remain magnetic in their lattice positions. A Kondo temperature (*T*_k_, 122 ± 10 K) and the maximum of the resonance (α, 6 ± 1 mV) are obtained by fitting the asymmetric differential conductance spectra with the Fano model.^267^ This *T*_k_ value is within the range of Kondo temperatures found for Co on Ag(111) and on Au(111) surfaces.^268^ It is important to mention that the Fe species are stabilized against diffusion and agglomeration even at elevated temperatures of about ∼300 K. Moreover, the chemically inert silica layer protects the embedded magnetic impurities against environmental influences, e.g., residual gas adsorption.

#### 4.1.2 Transition Metal Atoms on Bilayer Silica/Ru(0001)

Beyond the monolayer silica/Mo(112), the bilayer silica film offers new possibilities in terms of metal adsorption. Due to its cagelike structure and weak coupling to the support, the diffusion and formation of various species within the porous networks need to be carefully considered. In particular, studies of the vitreous areas within the BL silica might reveal additional insights into the adsorption mechanisms in bulk porous silica materials.

##### 4.1.2.1 Effect of the Pore Size on Adsorption

**Figure 48:**
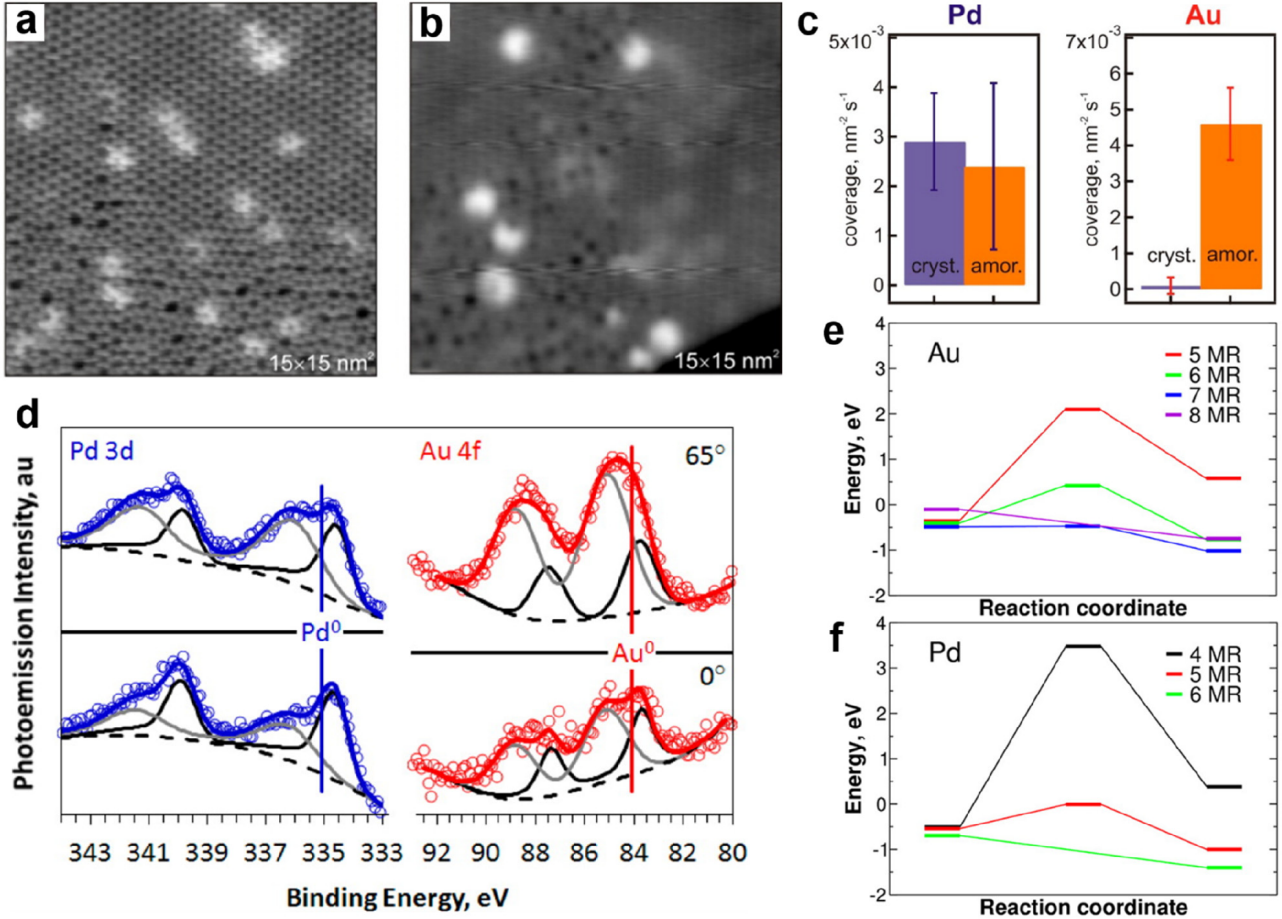
Low-temperature STM images of (a) Pd and (b) Au adatoms on bilayer silica/Ru(0001) (15 × 15 nm^2^, *U*_s_ = 2.0 V, *I* = 0.1 nA). (c) Coverage statistics of Pd and Au adatoms on bilayer silica/Ru(0001) as evaluated from STM images amounting to 3700 and 3000 nm^2^, respectively. (d) Angle-dependent XPS for 0.05 MLE Pd and 0.02 MLE Au deposited on silica/3O–(2 × 2)/Ru(0001), respectively. (e, f) Penetration profiles for Pd and Au through rings of different sizes. Energies are calculated with respect to the metal atom in the gas phase. Reproduced with permission from ref (269). Copyright 2014 American Chemical Society.

Of particular interest is the investigation of the variations in silica pore sizes on the influence of adsorption mechanisms of different metal atoms. Buchner et al. have conducted adsorption studies of Pd and Au on mixed-phase bilayer silica films with an emphasis on structures, locations, binding energies, and resultant electronic properties of the adatoms.^269^ The mixed-phase silica bilayer contains both crystalline (6-membered ring) and amorphous regions (ranges from 4- to 9-membered rings). Given the low deposition temperature of ∼5 K, the metal species adsorb predominantly as monomeric species. As shown in Figure 48a,b, the majority of the adsorbed Pd and Au appear as bright protrusions of butterfly and crescent shapes, respectively. It is noteworthy that there is no adsorption preference for Pd atoms on crystalline and amorphous regions of the silica, whereas Au is only observed within the amorphous domains (Figure 48c). Besides the binding of isolated atoms under the silica layer, Au adatoms or small clusters were also found on the domain boundaries within the crystalline areas that consist exclusively of alternating 5- and 8-membered rings. This behavior is consistent with Au on ML silica/Mo(112). Moreover, given the same terminations between the ML and BL silica films, it would be reasonable to expect Pd and Au atoms to diffuse into the pores of the silica films. Pd readily enters within both crystalline and amorphous domains of the films, while Au binds exclusively within amorphous regions (or domain boundaries), which exposes larger ring structures.

The adsorption of Pd and Au on BL silica films results in considerably higher binding energy (BE) shifts of the silica-related core-levels, which is similar to those observed after removing surface chemisorbed oxygen (O_Ru_) from the “O-rich” silica/Ru(0001) interface.^133,134^ The nature of the binding processes was revealed from the electronic structure of the adsorbed metals (Figure 48d). Starting with the lower Pd (Au) coverages, two distinct features occurred, with one at higher and the other at lower BE regions as compared to bulk Pd^0^ (Au^0^). While the peak at the higher BE region may result from several effects (e.g., reduced final-state screening,^270^ lattice contraction,^271^ and/or charge-transfer to the surface), there are relatively few effects that would be expected to induce shifts to lower BE of the supported metals particles (e.g., surface core-level shifts^272^). According to the angle- and coverage-dependent XPS results, the lower BE component can be assigned to metal species penetrating the pores of the film, and the component at higher BE can be assigned to metal binding over the silica surfaces.^273^ The interpretation of the shifts in XPS spectra is involved (Figure 48d). An Auger-parameter analysis is resorted to in order to separate initial and final state effects, and we refer to the original paper for details.^273^ The outcome of the analysis is that both species, i.e., the interfacial Pd atoms, as well as those on top of the silica film, are basically charge neutral. Due to the uncertainties in determining peak positions in XPS and Auger spectra, this is also consistent with a slight positive charge, which is in line with the prediction of the DFT calculations.

DFT calculations showed that Pd binds preferentially to the unoccupied Ru 3-fold hollow site with an *E*_ads_ of −3.60 eV. Such an off-center adsorption site, with respect to the silica ring, is the cause of the “butterfly” contrast in STM images. Au also binds strongly at the interface with an *E*_ads_ of −2.58 eV once it penetrates through the silica ring. The energy profiles and barriers for Pd and Au diffusion into the amorphous phase are explored by performing calculations with unsupported silica models (Figure 48e,f). Pd atoms can enter the cage via a nonactivated process for *n*-membered rings (*n* ≥ 6). In comparison, the penetration barrier for Au only starts to drop considerably with 7-membered rings (<0.1 eV). It should be noted that the metal atoms can hardly be stabilized inside the bilayer cage and will easily reach the silica/substrate interfaces. While the metal clusters on the silica surface appear to be effectively neutral, there are partial charge transfers of 0.32*e* (0.24*e*) from the interface-isolated Pd (Au) atoms to the Ru substrate. However, the lower component of the Pd(Au) core-levels in Figure 48d predominantly results from the lower coordination and orbital rehybridization effects rather than charge transfer effects.

Temperature-dependent XPS results show that the higher component shifts downward, and the lower component disappears gradually upon heating the Pd/silica film above 300 K. These two components are no longer distinguishable and produce a single Pd 3d_5/2_ peak, indicating the diffusion of the interface Pd atoms and the growth of silica-bound Pd clusters.^274^

##### 4.1.2.2 Effect of Surface Hydroxyls on Adsorption

**Figure 49:**
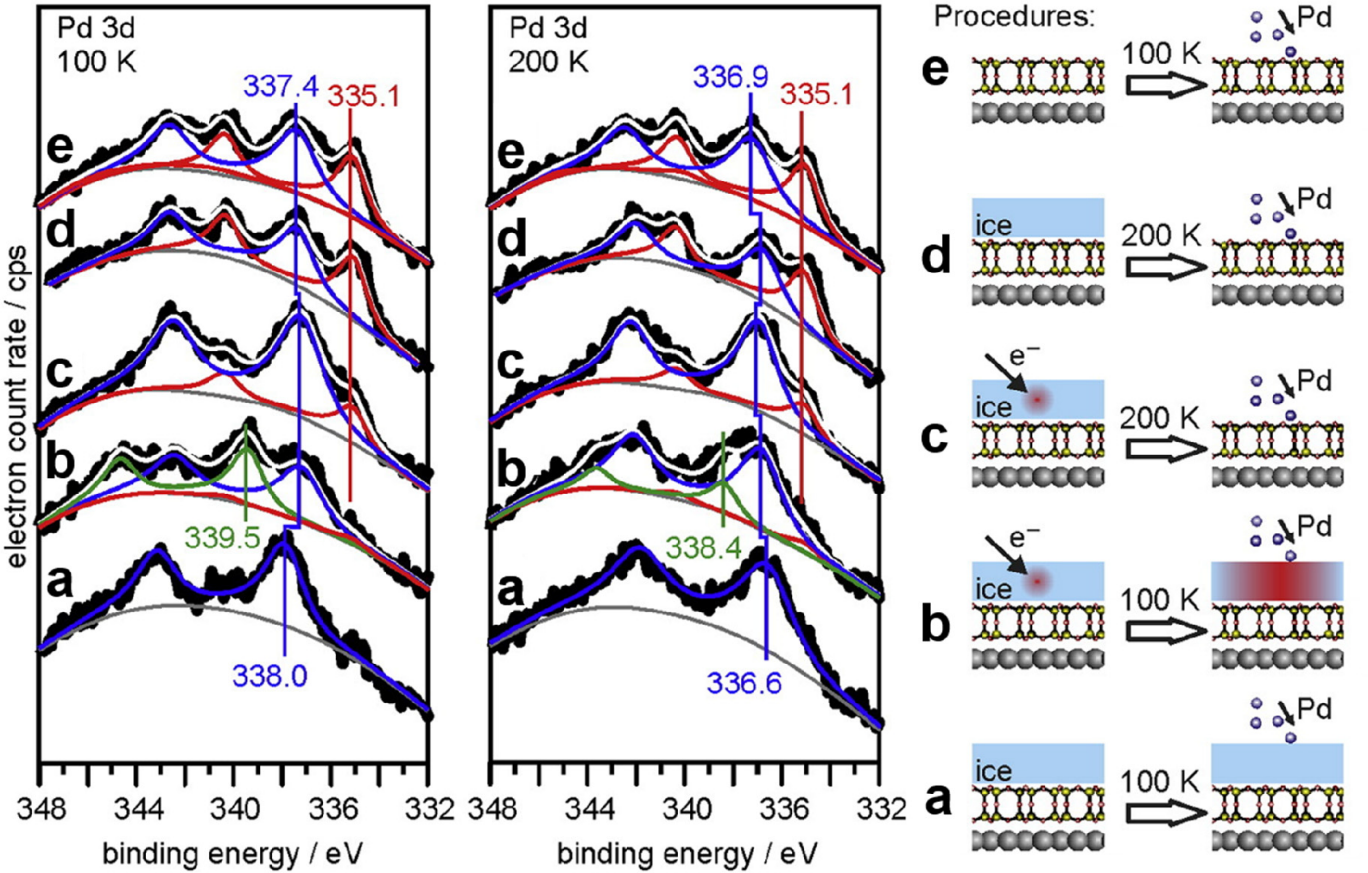
XPS of Pd 3d at 100 K (left) and 200 K (middle) for five different Pd/silica/Ru films. The Pd (0.05 ML) was deposited onto a (a) D_2_O precovered silica/Ru surface at 100 K; (b) D_2_O precovered silica/Ru surface with electron bombardment at 100 K; (c) highly hydroxylated silica/Ru surface at 100 K (hydroxylation with electron bombardment at 100 K, and the D_2_O was subsequently removed by heating it to 200 K); (d) much less hydroxylated silica/Ru surface at 100 K (hydroxylation without electron bombardment at 100 K, and the D_2_O was subsequently removed by heating it to 200 K); and (e) pristine BL silica/Ru surface at 100 K. Reproduced with permission from ref (274). Copyright 2016 Elsevier B.V.

To investigate the effect of water and surface hydroxyls on silica’s permeability, silica/Ru surfaces with five different preparation protocols were used for Pd adsorptions (Figure 49). Starting with the ice-covered silica film (Figure 49a), Pd atoms nucleate as clusters on the ice layer with a solitary peak centered at 338.0 eV. This peak shifts downward after removing the ice layer by annealing the film to 200 K, which is more consistent with the peak position associated with Pd clusters on the pristine silica/Ru surface (Figure 49e). If the ice layer is electron bombarded prior to the Pd adsorption, an additional peak then appears at the higher BE and can be attributed to oxidized Pd via the interaction with hydroxyl groups (Figure 49b).^275,276^ Therefore, the amorphous ice layer can effectively impede the diffusion of Pd atoms through the silica film. In Figure 49c, Pd was deposited on a highly hydroxylated silica/Ru surface. The presence of a small peak located at low BE suggests the penetration of the Pd atoms and their binding at the silica/Ru interface. Nonetheless, the amount of Pd atoms, having penetrated the silica film, is small compared to the one on top of the pristine silica/Ru surface (Figure 49e), indicating that the surface hydroxyls do significantly increase the probability of Pd nucleation over the silica surface. Not surprisingly, the Pd deposited on a much less hydroxylated silica/Ru surface shows a diffusion behavior similar to the one observed on pristine silica as shown in Figure 49d.

Thus, in terms of permeability, the silica surfaces can be roughly categorized into three groups, i.e., nonporous (Figure 49a,b), semiporous (Figure 49c), and porous (Figure 49d,e). It should be noted that the hydroxyl-bound molecular water is the leading cause of the semiporous property of the hydroxylated silica surface, where the limited penetrations of Pd atoms through the film are most likely realized by pore blockage with water molecules. However, surface diffusion of Pd atoms becomes significant at temperatures above 300 K in all cases, which results in the gradual growth of larger supported Pd clusters.

##### 4.1.2.3 Effect of Metal Substrate on Adsorption

It has been discussed in section 4.1.1.4 that the adsorption energy for Pd adatoms on ML silica largely depends on the coverage of surface chemisorbed oxygen (O_Mo_). A little bit similar, Pacchioni and co-workers found that the adsorption energies of Pt (Au) adatoms on BL silica also strongly depend on the substrate, ranging from −0.50 (−0.17) eV (freestanding silica) to −1.17 (−0.12) eV (Pt-supported silica) and −1.27 (−1.25) eV (Ru-supported silica).^277^ According to the DFT calculations, Pt (Au) adatoms stay on top of the silica surfaces and keep their neutral states on both freestanding silica and silica/Pt, while they turn to be negatively charged on the silica/Ru surface (−0.47*e* and −0.61*e* for Pt and Au, respectively). The most favorable adsorption sites vary from O-top (freestanding silica) to bridge (Pt-supported silica) and Si-top (Ru-supported silica).

To some extent, the support-dependent oxidation state of Pt (Au) can be attributed to the different work functions of the Pt(111) support (4.97 eV) and Ru(0001) support (3.67 eV). In principle, one could expect that Pt (Au) will accept electrons if the Fermi level of the support lies at an energy level above the adatom’s lowest unoccupied molecular orbital (LUMO). The smaller the work function of the support, the higher the ability to donate an electron to the electronegative adatom, in particular to Au.

##### 4.1.2.4 Effect of Confinement on the Adsorbed Cu Oxide Clusters

**Figure 50:**
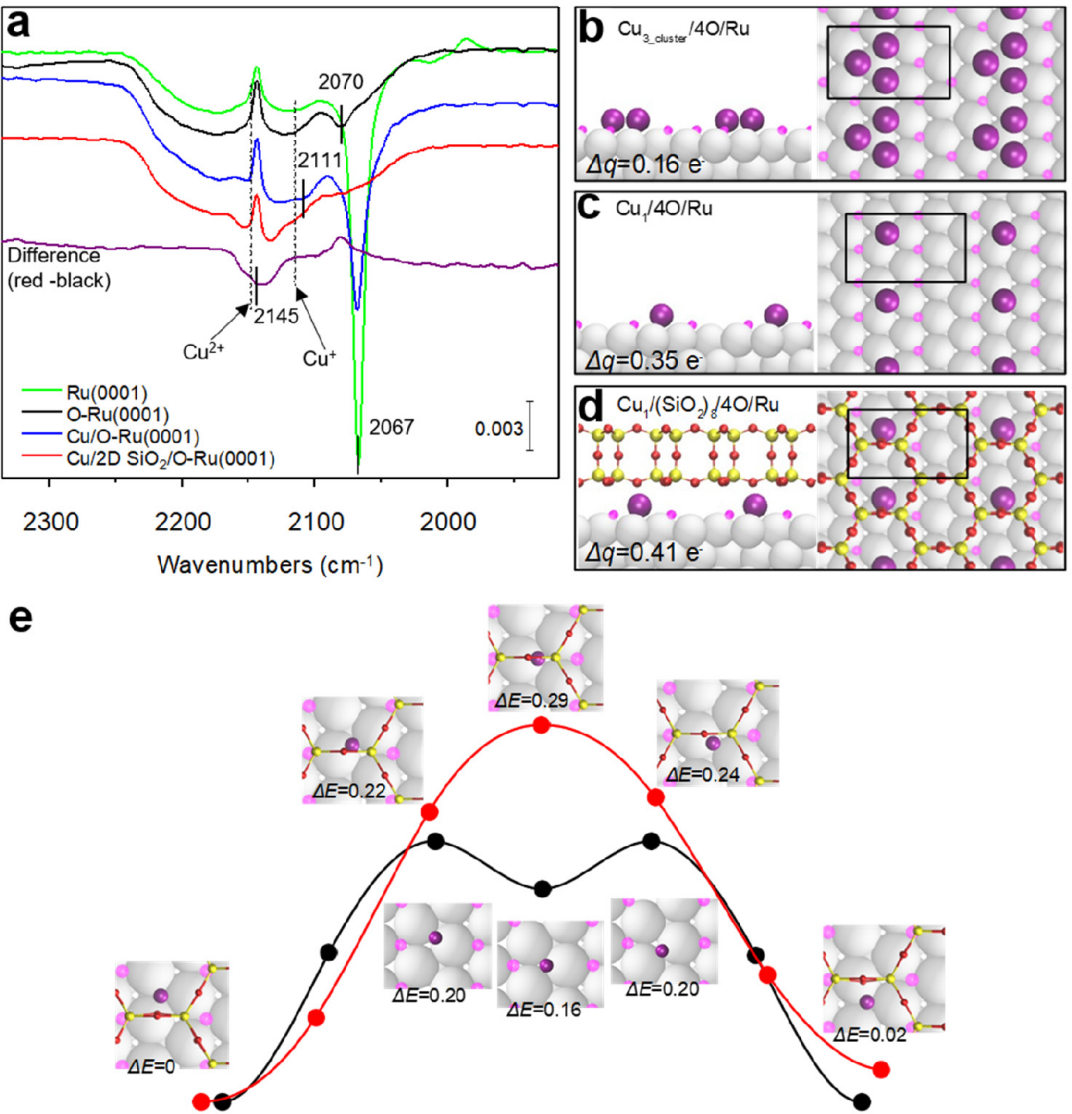
(a) *In situ* IRAS data was obtained under a 1 Torr CO environment. From top to bottom: clean Ru(0001) (green); (2 × 2)-3O/Ru(0001) (black); 0.01 ML Cu on (2 × 2)-3O/Ru(0001) (blue); 0.01 ML Cu on 2D-SiO_2_/(2 × 2)-3O/Ru(0001) (red). The purple spectrum is the difference between the red and black spectrum. The dashed lines in panel a indicate the frequencies from the literature for CO on Cu^2+^ and Cu^+^ sites. (b–d) DFT-calculated relaxed structures for Cu_3-cluster_/4O/Ru, Cu_1_/4O/Ru, and Cu_1_/(SiO_2_)_8_/4O/Ru, respectively. *Δq* is the number of electrons transferred from the Cu atom to the substrate. (e) DFT-calculated minimum energy path for the diffusion of a Cu atom on the 4O/Ru surface (black) and at the (SiO_2_)_8_/4O/Ru(0001) interface (red). *ΔE* is the diffusion energy barrier (Cu, purple; Ru, white; Si, yellow; O in silica film, red; and O chemisorbed on Ru, pink). Reproduced with permission from ref (278). Copyright 2018 Springer Nature.

Besides metal atoms/clusters, silica films can also be used to trap oxide clusters. Akter et al. have studied the silica-supported copper oxide clusters by depositing diluted copper atoms (∼1% ML) on BL silica/Ru films.^278^ It was found that dispersed Cu atoms can easily be oxidized by surface chemisorbed oxygen (O_Ru_) on the Ru substrate, resulting in stabilized Cu^2+^ cations. The oxidation state of Cu was investigated by *in situ* IRAS measurements with CO as a probe molecule (Figure 50a), whose vibrational frequency is very sensitive to the oxidation state at the adsorption site, such as the Cu^n+^ sites. In Figure 50a, the green and black spectra are obtained for CO on clean Ru(0001) and (2 × 2)-3O/Ru(0001) surfaces, respectively. Clearly, surface chemisorbed oxygen atoms can significantly block the adsorption of CO on Ru. The blue and red spectra correspond to CO on Cu/(2 × 2)-3O/Ru(0001) and Cu/2D-SiO_2_/(2 × 2)-3O/Ru(0001), respectively. Due to the overlap with the rotational band of the gas-phase CO, the small feature at ∼2111 cm^–1^ (blue) is difficult to distinguish, but its presence is evident when compared with the spectrum without Cu atoms (black). It has been well-established in the literature that CO has a vibrational frequency of 2148 (2115) cm^–1^ on Cu^2+^ (Cu^1+^) site (see dashed lines in Figure 50a).^279,280^ Therefore, the feature at 2111 cm^–1^ can be assigned to CO adsorbed on Cu clusters with exposed Cu^+^ sites, although undercoordinated Cu^0^ cannot be fully ruled out.^281^ These partially oxidized copper clusters are realized by “stealing” the chemisorbed oxygen atoms from the (2 × 2)-3O/Ru(0001) surface, resulting in bare Ru patches exposed for CO adsorption (2067 cm^–1^ peak in the blue spectrum). For comparison, a broad weak feature (2067 cm^–1^) and a new weak feature (2145 cm^–1^) are observed for CO on Cu/2D-SiO_2_/(2 × 2)-3O/Ru(0001) (red spectrum). This new peak at 2145 cm^–1^ is clearly seen in the difference spectrum (purple). It can be associated with the CO on highly oxidized Cu clusters (Cu^2+^), which remain stable under ultrahigh vacuum conditions. Note that the formation of Cu^2+^ has been reported only with the presence of oxygen.^280^

The oxidation states of deposited Cu atoms are quantified by DFT employing a Bader charge analysis. As shown in Figure 50b–d, on (2 × 2)-3O/Ru(0001) surfaces, the Cu clusters and dispersed Cu atoms lose 0.16*e* and 0.35*e*, respectively, suggesting that dispersed Cu atoms can be more easily oxidized than small Cu clusters. The presence of the silica film further increases the number of transferred electrons (0.41*e*), consistent with the binding energy shifts of the core-levels in XPS measurements. In principle, both the Cu and the O_Ru_ distribution have a substantial impact on the oxidation state of the surface adsorbed Cu. The presence of silica creates a higher diffusion barrier for Cu atoms, which prevents Cu atoms from clustering and thereby increases the Cu atom oxidation. The Cu diffusion pathway and energy barrier on the (2 × 2)-3O/Ru(0001) surface and at the silica/(2 × 2)-3O/Ru(0001) interface are shown in Figure 50e. It should be noted that the diffusion barrier would be even larger once the Cu atoms react with O_Ru_ due to the chemical and steric constraints. Thus, the silica bilayer induces a more dispersed Cu coverage which leads to oxidation.

##### 4.1.2.5 Pd Films on Aluminosilicate

It has been established that single Pd atoms can penetrate the bilayer silica film. However, when a thick Pd film (2 nm) was deposited onto the bilayer aluminosilicate, two very distinct film morphologies were observed by AFM/STM, LEEM, and X-ray photoemission electron microscopy (XPEEM).^282^ First of all, as evident by XPS depth profiling measurements, most Pd permeates through the hexagonal cages in the aluminosilicate framework (Al_0.35_Si_0.65_O_2_/Ru(0001)). The bilayer nature of the aluminosilicate framework was preserved after Pd deposition, in which the characteristic phonon mode at 1276 cm^–1^ was attenuated in intensity but remains unaltered in frequency. Large aluminosilicate terraces were partially covered by Pd particles (∼30 nm) with smaller particles in between. However, a flat wetting film was produced on narrow aluminosilicate terraces, corresponding to one or two layers of Pd. The stronger interaction with the Ru(0001) support and the presence of Brønsted acid sites in bilayer aluminosilicate can potentially affect the permeability of Pd adatoms. Further studies are still needed to gain a better understanding of the reasons why two different Pd morphologies are observed on aluminosilicate surfaces.

### 4.2 Adsorption of Alkali Atoms (Li, Na, and K)

The stabilities and properties of alkali metal atoms or ions in the cages of zeolites are vital if one wants to modify zeolites functionally.^283−285^ The small ionization potential of alkali metal atoms is technologically important. It is well-known that alkali atoms deposited on metal surfaces can induce significant changes in the work function (WF, Φ) of the systems.^286^ Furthermore, alkali atoms are widely used as promoters in catalytic applications for their ability to supply weakly bound electrons during the reduction processes.^287^ In this section, the adsorption properties of Li, Na, and K atoms on 2D-silica will be briefly reviewed.

#### 4.2.1 Alkali Atoms on Monolayer Silica/Mo(112)

**Figure 51:**
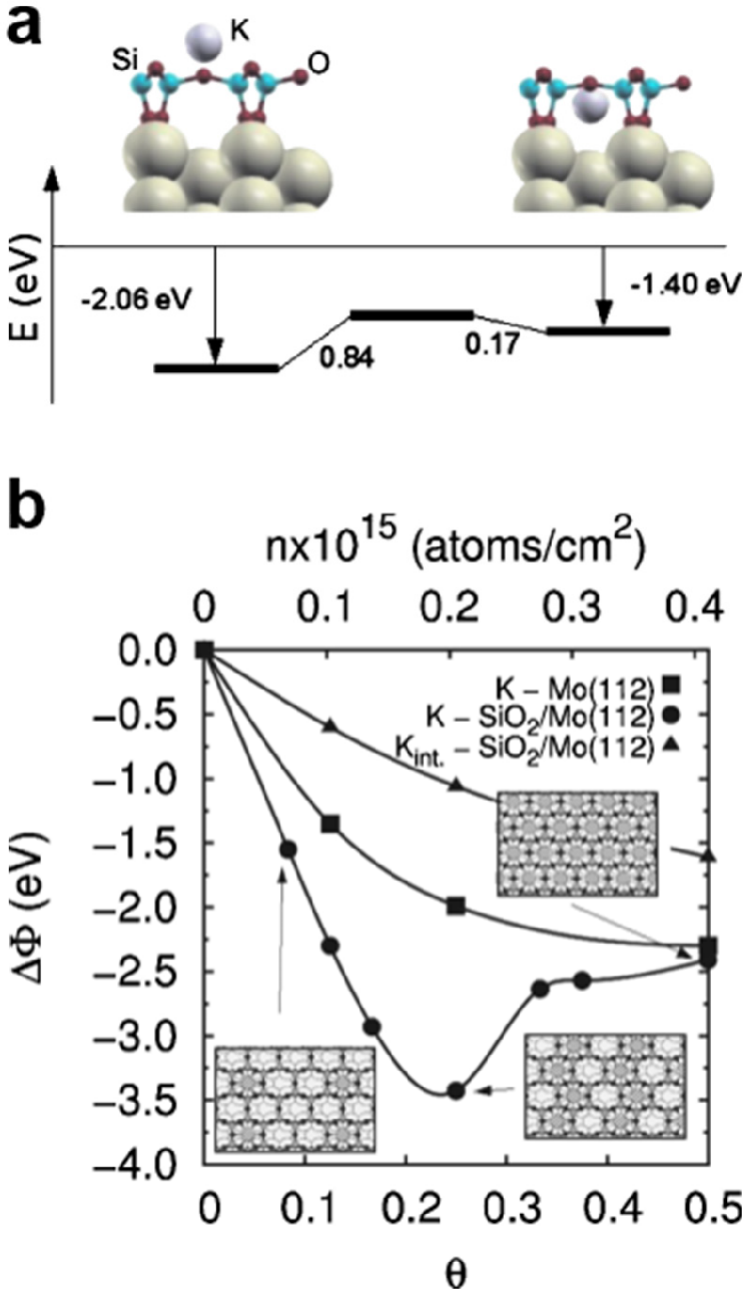
(a) Energy profile for a K atom adsorbed above and at the interface of a SiO_2_/Mo(112) film. For K at the interface, it sits 2.46 Å above the Mo surface. Na and Li bind in similar positions with alkali–Mo distances of 2.05 and 1.57 Å, respectively. (b) Work function changes (ΔΦ) in K/Mo(112), K/SiO_2_/Mo(112), and K_int_-SiO_2_/Mo(112) as a function of the K coverage. Reproduced with permission from ref (288). Copyright 2008 American Physical Society.

Similar to transition metal atoms on silica/Mo(112), two different adsorption sites can be identified. For example, Li, with a small ionic radius, prefers to adsorb at the silica/metal interface, while K, which is larger, prefers to bind above the silica layer. For Na, these two sites are almost isoenergetic due to its size with respect to the 6-membered ring of silica. As shown in Figure 51a, the adsorption energy of a K adatom on the silica surface (2.06 eV) is larger than the one at the interface (1.40 eV). This is opposite to the behavior of Pd adatom on silica/Mo(112), as discussed in section 4.1.1.1.

##### 4.2.1.1 Tuning the Work Functions of Silica/Mo(112)

The properties of K, Na, and Li atoms adsorbed on ML silica/Mo(112) were first studied by Pacchioni and co-workers using DFT calculations, with particular emphasis on the changes in the film’s work function.^288^ It was found that there is a net charge transfer from the outer ns electron of the alkali atom to the Mo conduction band, which results in surface dipoles and thus lowers the work function substantially. The changes in work function (ΔΦ) depend on the adsorption sites of the alkali atoms and their coverages (Figure 51b). First, the work function is always larger when the alkali atom sits above the silica film, which correlates with the height of the alkali atom from the metal layer. For example, at a coverage of 0.125 (i.e., one alkali atom per every eight Si atoms), the work function changes (ΔΦ) are −2.3(−0.60), −1.66(−0.50), and −1.47(−0.35) for K, Na, and Li on the silica surface (at the interface), respectively. The smaller ΔΦ for alkali atoms at the interface was attributed to the shorter alkali–Mo distance and a partial screening of the positive charge by the polarizable Mo metal electrons. Second, the ΔΦ curve for K on the silica surface shows a typical coverage-dependent behavior, with a rapid decrease at low coverages, a minimum at a critical coverage, and a characteristic value at higher coverages, which is typically observed for alkali atoms on metal substrates.^286,289^

The change in work function induced by the adsorbed alkali metal atoms can, therefore, be used to modify the properties of the silica films. For example, the alkali-modified silica may be a valuable system for studying the adsorption of electronegative species, as the reduced work function will promote charge transfer processes out of the film and stabilize anionic species with enhanced chemical activity.^290^

##### 4.2.1.2 Structural and Electronic Aspects of Li on Silica/Mo(112)

**Figure 52:**
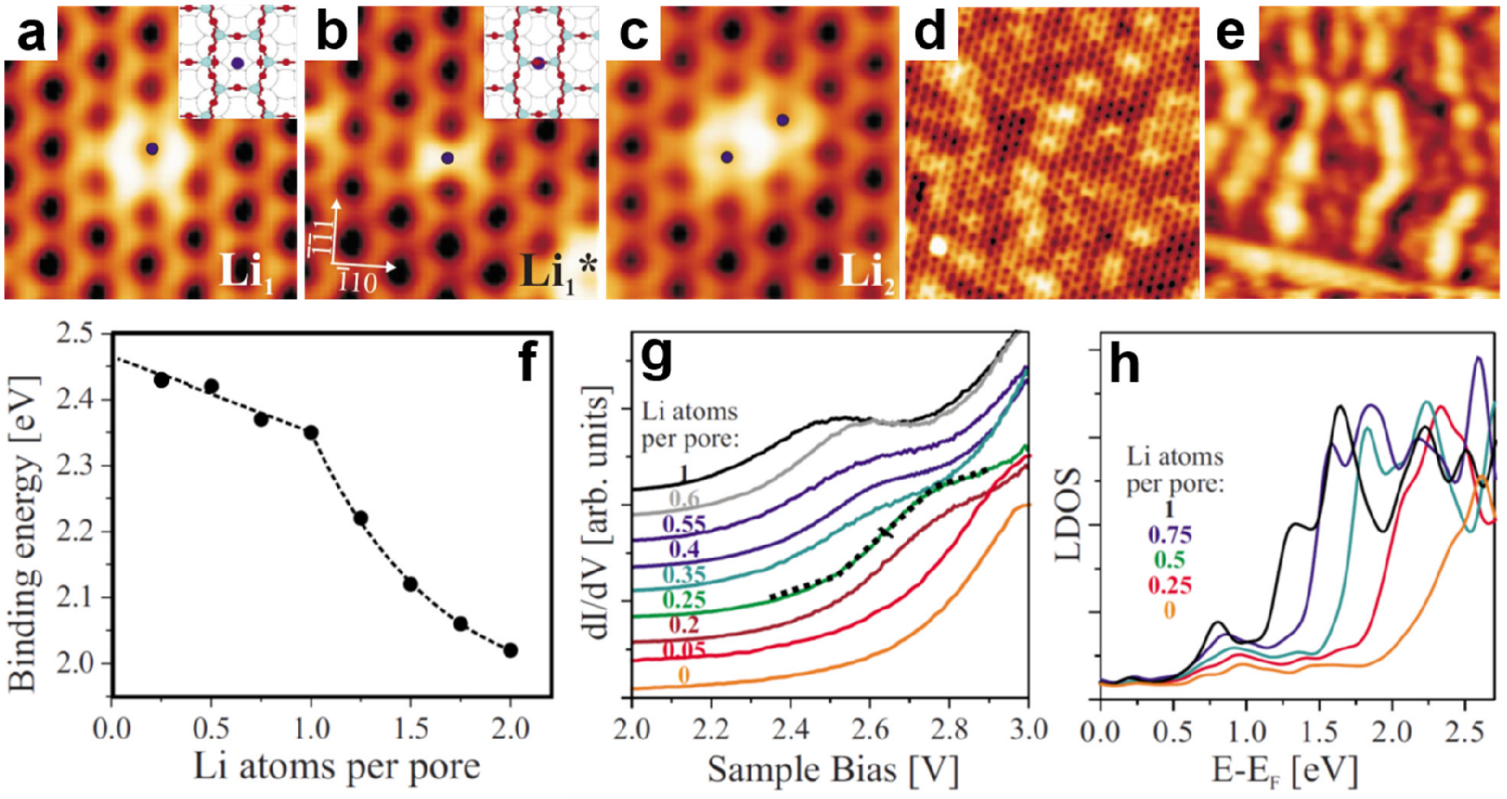
STM images of Li atoms adsorbed on silica/Mo(112): (a, b) single Li atom bound at two different interface sites (3 × 3 nm^2^, *U*_s_ = 0.1 V); (c) Li dimers (3 × 3 nm^2^, *U*_s_ = 0.1 V); (d) nominal coverage of 0.2 Li atoms per pore (15 × 15 nm^2^, *U*_s_ = 0.5 V); (e) nominal coverage of 0.4 Li atoms per pore (15 × 15 nm^2^, *U*_s_ = 2.5 V). (f) Coverage-dependent Li binding energy at the silica/Mo(112) interface. (g, h) Conductance spectra and calculated LDOS of the silica/Mo(112) with different Li concentrations. Reproduced with permission from ref (291). Copyright 2009 American Physical Society.

Li atoms are able to penetrate the silica layer with a small activation barrier of 0.3 eV and strongly bind at the silica/Mo(112) interface. Two distinct adsorption structures are observed in STM experiments (Figure 52a,b), namely, a ring-shaped structure (with Li directly below a −Si–O– ring) and an X-shaped structure (with Li at a Mo hollow site that is oriented along the Mo[1̅10] direction). The two adsorption sites are thought to be induced by an intermixing of the Li 2s and the unoccupied Si 3s–O 2p hybrid states in the silica ring, and hybridization of the Li 2s state and the states in the Si–O–Si unit, respectively.^291^ A similar behavior has also been observed for Pd, Ag, and Fe adatoms.^253,255,266^ The Li atoms become cationic in those binding configurations.

Larger adsorbate structures may be assembled from these two elementary configurations. With increasing Li coverage, the distribution of Li at the interface becomes more and more inhomogeneous. Elongated Li stripes develop at critical coverages with ordering along the [1̅10] and [111] directions of Mo(112) (Figure 52d,e). Nucleation of Li clusters occurs above a nominal Li coverage exceeding one atom per pore, which is consistent with DFT calculations (Figure 52f). The binding energy of a Li adatom only starts to decrease significantly above a maximum coverage (i.e., one atom per pore) due to the increasing Coulomb repulsion between Li ions. It is also important to mention that the penetration barrier for the Li adatom decreases when the neighboring pores are preoccupied with Li ions. The charge-density oscillations in the Mo surface are responsible for the spatial distribution of the Li ions (e.g., the Li stripes). For example, the diffusion barrier for Li along the Mo[1̅10] direction is 0.75 eV, while it drops to 0.09 eV along the Mo[111] direction.^292^

Similar to the Fe adatoms on silica/Mo(112),^266^ the surface corrugations of Li incorporated into the silica measured in STM mainly originate from electronic effects. The Li-rich stripes exhibit considerable apparent heights at elevated STM bias, suggesting the local availability of new conductance channels in those regions, which was confirmed by the STM conductance spectra taken on a silica surface with different Li content (Figure 52g,h) and DFT-calculated LDOS. The gradual downshift of silica conduction states with increasing Li coverage can be rationalized by the work function reduction in silica/Mo(112) upon Li incorporation, as discussed in section 4.2.1.1. In principle, such electronic tuning of the silica layer via Li adsorption may be applied to other oxide systems.

##### 4.2.1.3 Anchoring and Charging Au Adatoms on Li/Silica/Mo(112)

**Figure 53:**
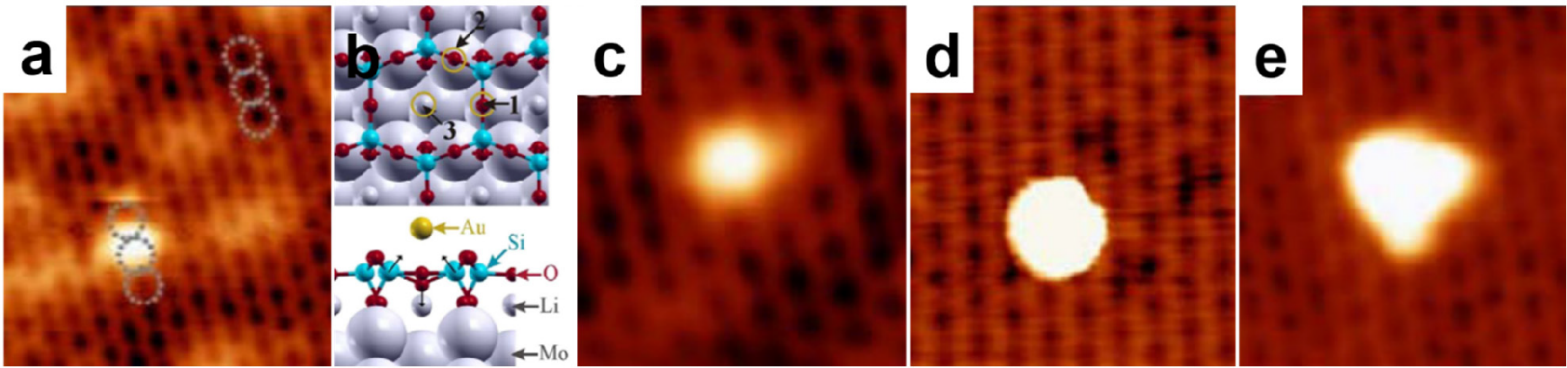
(a) STM image of a single Au adatom on Li-doped silica. The adatom is located between two hexagonal rings as marked by the dashed circles. (b) Top panel: corresponding structural model showing potential adsorption sites for Au adatoms, i.e., site 1 (on the O atom of a [1̅10] oriented Si–O–Si bridge), site 2 (on a Si–O–Si bridge on top of a Mo row), and site 3 (above a pore). Bottom panel: schematic representation of the polaronic distortion induced by the Au adatom on site 1. (c–e) STM images of Au clusters with increasing sizes on Li-doped silica. The growth of Au clusters follows the Vollmer-Weber mode, which results in the formation of 3D particles. Reproduced with permission from ref (290). Copyright 2009 American Physical Society.

As discussed in section 4.1.1.1, Au atoms cannot bind to the defect-free silica/Mo(112) film and rapidly diffuse on the surface until they become trapped at domain boundaries, where they serve as nuclei for the growth of 3D particles.^253^ In contrast, Li doping induces dramatic changes in Au adsorption.^290,293^ As shown in Figure 53, Au atoms and clusters are stabilized on the Li-doped defect-free silica terraces. At low Au coverage, the adsorbed Au has a spherical shape and sits on top of a [1̅10] oriented Si–O–Si bridge (Figure 53a). From the DFT calculations for the system with Li-doped silica/Mo (Θ(Li) = 1), there are three local minima for Au adsorption with binding energies of 1.33 eV (site 1), 0.34 eV (site 2), and 0.18 eV (site 3), respectively (see the top panel in Figure 53b). Site 1 is the most energetically favorable position for Au adatoms, which is consistent with the experimental observations. Moreover, there is a charge transfer from the support to the Au 6s orbital. The formation of anionic Au species is essentially stabilized by the strong polaronic distortion of the silica films (see the bottom panel in Figure 53b, the O atom of the top layer relaxes downward by 0.85 Å while the adjacent Si atoms relax toward the Au anion by 0.1 Å). A similar lattice distortion has also been observed for Au adsorption on other oxide films.^294^

It should be noted that the stability of the adsorbed Au anion depends on the Li coverages; i.e., the higher the Li coverages (Θ from ∼0.25 to ∼1), the larger the adsorption energy (*E*_ads_ from ∼0.48 to ∼1.33 eV).^293^ However, the degree of charge transfer is independent of Li coverage, indicating the importance of the polaronic distortions. In addition, no change occurs in the position of Li ions at the interface upon Au adsorption.

Such strongly bound Au anions act as nucleation centers for further Au cluster growth. Therefore, with increasing Au coverage, 3D particles develop with various shapes (Figure 53c–e). Interestingly, according to the DFT results, the Au dimer was found to be neutral on Li-doped silica/Mo (Θ(Li) = 1).^293^ Given the absence of charge transfer, the binding energy between the Au dimers and the Li/silica/Mo(112) is weak, ranging from 0.12 to 0.36 eV, which is slightly dependent on the geometry of the Au dimers. The closed-shell nature of the Au dimer is responsible for its neutral character, in which it does not easily bind an extra electron. However, for larger adsorbed Au clusters, such as Au_3_, Au_5_, and Au_20_, a smaller negative charge is associated with these anchoring atoms. Similar to the situation described for a single Au atom, these surfaces undergo a polaronic distortion. Thus, the Au particles can be pinned to distinct positions in the Li-doped silica films.

Generally, certain conditions must be fulfilled in order to anchor and charge the Au adatoms on silica/Mo. First, there must be direct electronic interaction between the support and the adsorbed Au atoms/clusters. Second, the substrate must undergo a polaronic distortion to stabilize the charged Au species. Lastly, the Fermi level of the silica/Mo film must be located above the empty states of the adsorbed Au species, which can be realized by Li^+^ doping.

#### 4.2.2 Alkali Atoms on Bilayer Silica/Ru(0001)

As a zeolite model, aluminosilicate can be used for ion exchange studies due to its ability to incorporate alkali metals in its cavities. In principle, the adsorption of alkali metal atoms on BL silica/Ru(0001) films will be similar to that of transition metal atoms, as discussed in section 4.1.2. Several aspects will determine the detailed adsorption behavior, such as the metal support, the doping element, and the atomic and electronic structure.

Schlexer et al. have theoretically studied the adsorption of alkali metal atoms on unsupported and supported BL silica with different ring sizes (4–8-membered rings).^295^ As expected, in the absence of point defects, silica films are inert, and the interaction of Li, Na, and K is dominated by dispersive and polarization contributions. There is no electron transfer between the adsorbed alkali metals and silica. Interestingly, the adsorption on silica surfaces is preferred for all alkali metals, but their adsorption energies do not follow a regular trend going from Li to Na, and to K. For example, on 6- and 7-membered rings, the adsorption energy is largest for K and smallest for Na. This can be attributed to different atomic polarizabilities (K > Na > Li) and atomic dimensions (K > Na > Li). However, the adsorption inside the cages is always unfavorable as compared to that on the surface. The stabilities of alkali metals increase with the ring size, and the adsorptions become exothermic (e.g., −0.33 eV for Li in 7-membered rings).

The interaction of alkali metal atoms with silica increases significantly when silica is doped with Al (i.e., aluminosilicate). For example, the adsorption energy changes from −0.37 eV (silica) to −4.05 eV (aluminosilicate) for Na on a 6-membered ring surface. According to Bader charge calculations, Na becomes cationic and transfers its valence electron to the Al. In comparison, Na^+^ adsorbed inside the cage is still less stable with respect to that on the surface, indicating that steric repulsion prevails over electrostatic interaction.

**Figure 54:**
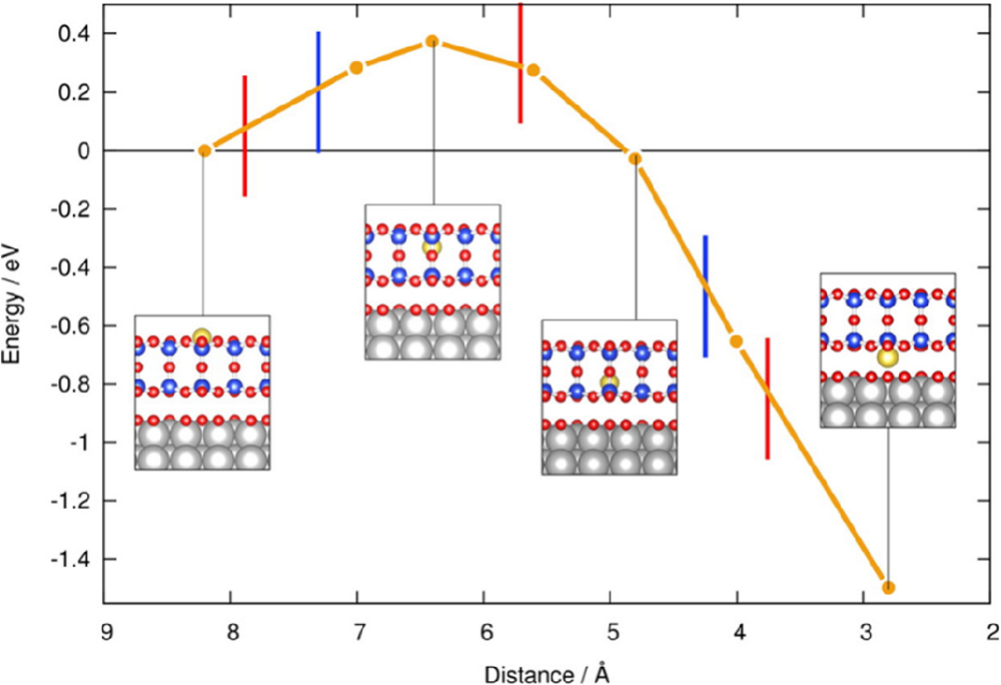
Potential energy curve for the diffusion of a Na atom into the bilayer silica/3O(2 × 2)/Ru(0001) films through the 6-membered rings. The zero of energy corresponds to a Na atom adsorbed on the surface of the silica bilayer. The positions of the O atoms and Si atoms in the silica bilayer are indicated by the red and blue vertical bars, respectively. Reproduced with permission from ref (295). Copyright 2014 American Chemical Society.

The adsorption behavior is very different on supported BL silica films (e.g., silica/3O(2 × 2)/Ru(0001)). On a supported 6-membered ring, the adsorption energy of Na (K) is much larger than that on unsupported films, i.e., −2.66 (−1.27) eV and −0.37 (−0.60) eV, respectively, indicating completely different bonding mechanisms. Even a larger adsorption energy is found at the silica/Ru interface (e.g., −4.16 eV for Na). It should be noted that no stabilization of Na inside the cage is found; Na spontaneously diffuses to the interface. Therefore, it is expected that an adsorbed Na atom can diffuse from the surface to the interface by overcoming a penetration barrier, that is, ∼0.4 eV as shown in Figure 54. This barrier disappears when Na is adsorbed on larger rings of the silica surface. A similar diffusion behavior is observed for Li on silica/Mo(112)^291^ and Pd on silica/Ru(0001).^273^ The adsorption of Na on the silica surface or at the silica/Ru interface does not change the distance between the silica and Ru support (∼3 Å). In addition, there is an electron transfer from the Na 3s states to the Fermi level of the support, indicating a full ionization of Na to Na^+^. The formation of Na^+^ substantially reduces the work function of the systems by an order of ∼1 eV or more, which is important for surface modifications and may turn the inert silica film into an active surface as discussed in previous sections.

### 4.3 Adsorption of Noble Gas Atoms (He, Ne, Ar, Kr, Xe, and Rn)

In contrast to metal atoms, noble gas atoms are the most unreactive elements in the periodic table, and they have much weaker interactions with the porous 2D-silica surfaces. However, the nanosized cages in the 2D-silica bilayer provide excellent opportunities to study the adsorption of those unreactive atoms and molecules in nanoconfinement. The study of the trapping mechanism is of great importance and could provide a guideline for designing highly efficient adsorbent materials and membranes for gas separations^107,296^ and nuclear waste remediations.^297−299^ Note that 3D porous materials have been previously used to trap noble gases, such as the 3D zeolites and metal–organic frameworks (MOFs).^300,301^ Surface trapping of noble gases is usually challenging, and it is typically achieved by condensation at cryogenic temperatures.^302^ Ion implantation and electrostatic trapping were also explored for confining noble gases on nanostructured surfaces.^303,304^

As already described in previous sections, besides the nanocages within the framework, there is a second type of confined space in metal-supported 2D-silica bilayer systems (i.e., the interfaces between the 2D framework and the metal substrate), which allows the size-selective diffusion of metal atoms and small molecules to intercalate at the interface.^241,242,305,306^ Recently, we have successfully studied the trapping and release of noble gases in 2D-silica films by using surface science methods and DFT calculations, which provides a new playground for the fundamental study of isolated noble gas atoms in nanoconfinement.^307−309^

#### 4.3.1 Single Atoms in a Nanocage

**Figure 55:**
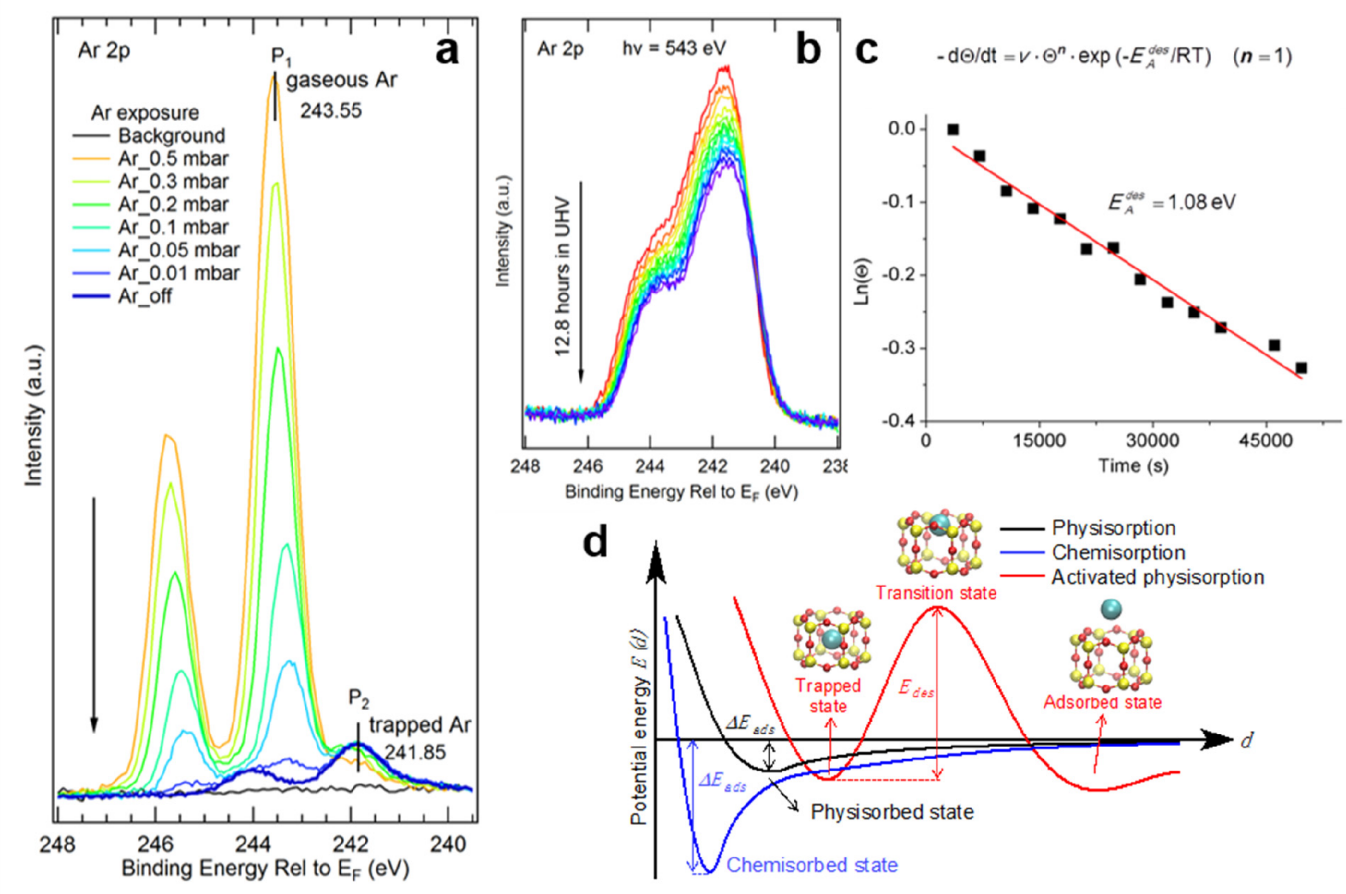
(a) Pressure-dependent XPS spectra of Ar 2p. The black spectrum in panel a is obtained in UHV before the Ar exposure, while the dark blue spectrum in panel a is obtained in UHV after the Ar exposure (photon energy *hυ* = 1000 eV). (b) Time-dependent XPS spectra of Ar 2p for the Ar-trapped bilayer aluminosilicate (Al_0.2_Si_0.8_O_2_). (c) Plot of the measured Ar peak areas using the Polanyi–Wigner equation. (d) Schematic diagram of the potential energies for physisorption, chemisorption, and activated physisorption, respectively. Δ*E*_ads_ is the adsorption energy, while *E*_des_ is the desorption energy barrier for activated physisorption. Reproduced with permission from ref (307). Copyright 2017 The Authors. Published by Springer Nature. Reproduced with permission from ref (308). Copyright 2019 WILEY-VCH Verlag GmbH & Co. KGaA, Weinheim.

**Figure 56:**
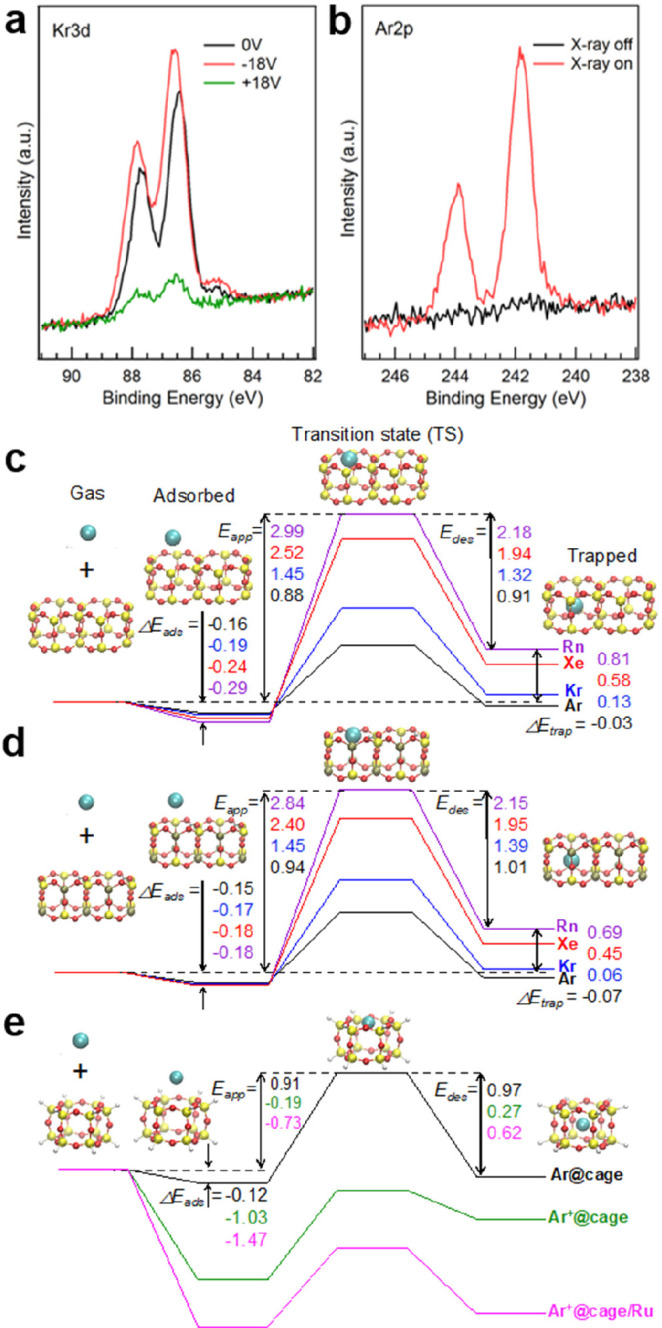
(a) Effect of sample bias on the trapping process. The silica bilayer was exposed to 2 mbar Kr for 10 min with 0 V (black), −18 V (red), and +18 V (green) sample bias, respectively, in the presence of an X-ray beam. The XPS spectra of Kr 3d were acquired under UHV conditions after evacuating the gas (photon energy *hυ* = 400 eV). (b) Effect of the X-ray beam on the trapping process. The silica bilayer was exposed to 2 mbar Ar for 10 min in the presence (red) and absence (black) of an X-ray beam. The XPS spectra of Ar 2p were acquired under UHV conditions after evacuating the gas (photon energy *hυ* = 650 eV). (c, d) Potential energy diagram from DFT calculations for Ar, Kr, Xe, and Rn atoms being trapped in silica and aluminosilicate bilayer films at Θ_cage_ = 0.25, respectively. (e) Potential energy diagram from constrained DFT calculations for a neutral Ar atom trapped inside a single freestanding silica nanocage, an Ar^+^ ion trapped inside a single freestanding silica nanocage, and an Ar^+^ ion trapped in a silica nanocage adsorbed on a Ru surface. Δ*E*_ads_ and Δ*E*_trap_ represent the adsorption energy outside the nanocage and the trapping energy inside the nanocage. *E*_app_ and E_des_ represent the apparent trapping and desorption energy barriers. Reproduced with permission from ref (308). Copyright 2019 WILEY-VCH Verlag GmbH & Co. KGaA, Weinheim.

The trapping of noble gas atoms in 2D-silica was demonstrated by *in situ* ambient pressure X-ray photoelectron spectroscopy (APXPS). As shown in Figure 55a, a well-ordered 2D-silica/Ru(0001) film was first exposed to 0.5 mbar Ar during the APXPS measurements, where the Ar 2p peaks (P_1_ and P_2_) were assigned to gas-phase Ar and surface-trapped Ar, respectively. This assignment is inferred from the fact that the P_1_ peak gradually decreases and eventually disappears, while the P_2_ peak remains even after evacuating the Ar gas. It should be noted that, except for He and Ne, other noble gas atoms (Kr and Xe) can also be trapped when the silica film is exposed to modest gas pressures during the APXPS experiments. Careful analysis (e.g., angle-dependent XPS) of the P_2_ peak reveals that most of the trapped Ar atoms are located within the hexagonal cages (Ar_cage_), and a small part of the trapped Ar is at a deeper location, i.e., the interface between the silica framework and the Ru(0001) support (Ar_inter_). The peak assignment in angle-dependent XPS is confirmed by DFT calculations, where the Ar 2p binding energy of Ar_cage_ is 1.12 eV higher than in the case of Ar_inter_. The saturation trapping coverage (Θ_sat_, defined as the number of trapped noble gas atoms per hexagonal cage) is calculated to be 0.14 ± 0.02, 0.20 ± 0.02, and 0.04 ± 0.02 for Ar, Kr, and Xe in a 2D-silica bilayer, respectively. In the case of 2D-aluminosilicate (e.g., Al_0.33_Si_0.67_O_2_), these coverages increase to 0.18 ± 0.02 (Ar), 0.26 ± 0.02 (Kr), and 0.12 ± 0.02 (Xe). A Θ_sat_ in aluminosilicates higher than in silica can be attributed to their larger trapping energies as will be explained in Figure 56c,d. It is important to mention that vitreous regions may coexist in addition to the hexagonal prism cages. According to the DFT calculations, the probability of trapping noble gas atoms (Ar, Kr, and Xe) in 5- and 7-membered rings is significantly lower than that of 6-membered rings. Therefore, the existence of vitreous regions in an as-prepared sample may lead to an underestimation of the coverage with respect to hexagonal prisms.

Compared to the 2D-silica/Ru(0001), it was found that the noble gas atoms cannot be trapped at the interface of the 2D-aluminosilicate/Ru(0001), which is attributed to the largely reduced interface distance between the aluminosilicate bilayer and the Ru support.^238^ Actually, the amount of trapped noble gas atoms at the 2D-silica/Ru(0001) interface can also be controlled by the interface distance, which in turn is governed by the coverage of chemisorbed oxygen on Ru(0001) (see section 3.3.1). For example, there is much less Ar trapped at the interface for a silica bilayer with less chemisorbed oxygen due to the smaller interface distance, leaving no space for noble gas atoms in this interfacial confined space. It is worth noting that the noble gas atoms trapped at the interface are less stable than those in the nanocages as inferred from temperature-dependent XPS. According to IRAS experiments, the inclusion of Ar atoms in a silica bilayer does not change the characteristic phonon vibration frequency of the framework associated with the perpendicular Si–O–Si linkage at ∼1300 cm^–1^, while there are an 8 cm^–1^ red-shift and a considerable broadening of the phonon peak upon Xe inclusion, most likely due to its non-negligible distortion of the bilayer upon Xe intake. DFT calculations show that the average O–O distances in the middle layer [*d*(O_m_–O_m_)] of the hexagonal prism cage were expanded by 0.05 and 0.14 Å for Ar and Xe inclusions at Θ_cage_ = 0.5, respectively.

The kinetics of desorption of Ar atoms from the nanocages were examined by time-dependent XPS for an aluminosilicate (Al_0.2_Si_0.8_O_2_) bilayer film at room temperature (Figure 55b,c). The XPS spectra (Ar 2p peak area) are displayed as a function of time, from which the rate of desorption can be determined. According to a Polanyi–Wigner analysis, the desorption rate follows an Arrhenius-type behavior, and therefore, the activation energy for Ar desorption is determined to be ∼1.08 eV.^310^ This experimentally derived activation energy is in good agreement with the DFT calculations (Figure 56c). However, this result is puzzling. How did the Ar atoms get trapped inside the nanocages at room temperature below the atmospheric pressure, if the activation energy barrier for Ar to enter the nanocages is at a similar magnitude of ∼1 eV.^296^

From the surface science point of view, the adsorption of a molecule on a surface is classified as either physisorption or chemisorption (Figure 55d). Since a noble gas atom would only form a weak bond with an inert silica surface, an activated physisorption mechanism has to be proposed for these trapped noble gas atoms with ultrahigh desorption energy barriers, where the noble gases are immobilized in the nanocages of the 2D (alumino)silicates.

Figure 55d shows schematic potential energy diagrams for an atom approaching the surface of a silica film, reducing the distance and being incorporated into a cage (either silica or aluminosilicate), where they are immobilized, in comparison to typical potential energy surfaces for physisorbed and chemisorbed species on a metal surface. The activation energy for the incorporation of a neutral, relatively extended Ar atom is large and thus remains unlikely for neutral Ar atoms. However, we may speculate that if the Ar atom was in its ionized state, the radius would be reduced, and the atom may enter the cage in the first step. We will come back to this below. Experimental evidence for the cationic nature of the noble gas atom during the trapping process comes from measurements of the bias dependence of the process, as evidenced in Figure 56. Figure 56a shows spectra of Kr, in this case, when there is no negative or positive bias, and the situation changes as expected if the noble gas atom is ionized. Positive bias decreases the trapping probability, and negative bias enhances it in comparison with an unbiased surface. Figure 56b, again shows that there is no trapping when X-rays are switched off. Of course, there are other possibilities to ionize the noble gas atoms, for example, via high electric fields,^311^ but in this case, X-ray-induced ionization is the cause.

Figure 56c,d compares, in more detail, the results of DFT calculations for energies of adsorption and trapping for the various neutral noble gases (Ar, Kr, Xe, and Rn) for the situations already schematically addressed in connection with the discussion of Figure 55d. Here, however, 2D-silica (SiO_2_) in Figure 56c and 2D-aluminosilicate (H_0.125_Al_0.375_Si_0.625_O_2_) in Figure 56d, without considering the metal substrate, are explicitly considered. The situation is rather similar for both materials. Only Ar shows a small exothermicity for trapping, while all other noble gases exhibit endothermicity. However, in all cases, the activation energies for trapping are rather large.

Figure 56e, on the other hand, compares the results of DFT calculations when the noble gas atom is ionized. The results for neutral Ar atoms are compared with Ar^+^ ions with and without the Ru surface being present. Here, the speculation, initially made, is verified: the smaller Ar^+^ ion may be trapped more easily than the neutral atom, as revealed by the lowering of the trapping energy from −0.07 to −0.19 eV, and substantially further down to −0.73 eV by the induced image potential. Concerning the elementary processes involved in the desorption of the trapped rare gas atom, we have to consider that the charged state has a very short lifetime near the metal surface. Thus, an electron is easily transferred to neutralize the noble gas atom before there has been sufficient nuclear motion of the noble gas atom to desorb.

#### 4.3.2 Separation of Noble Gases

As discussed in the previous sections, the energy barriers for the desorption of noble gases from the pure silica framework are rather large. The associated temperatures range from 373 K for Ar via 473 K for Kr up to 623 K for Xe, as judged by XPS taken as a function of temperature. In comparison, they increase even higher for the aluminosilicate framework, i.e., toward 498 K for Kr and 673 K for Xe. The latter value underlines the high stability of the trapped Xe and represents the highest stability of a trapped noble gas atom in a confined space. It is not surprising that DFT calculations performed for trapped Rn predict an even higher stability, specifically a desorption temperature of 775 K.

**Figure 57:**
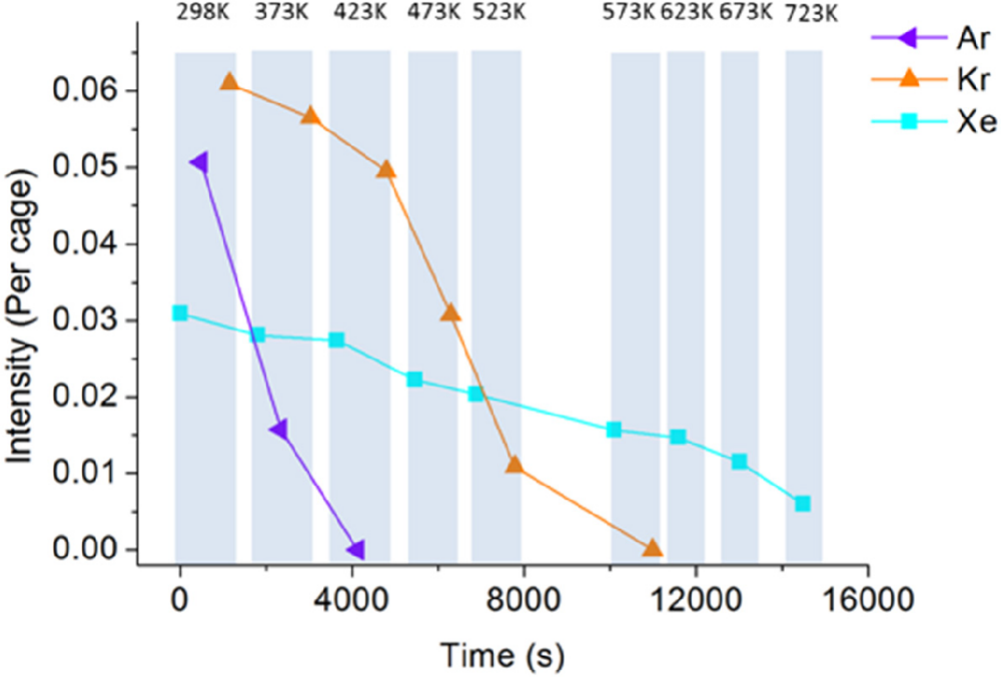
XPS analysis of mixed noble gas atoms trapped in a 2D aluminosilicate (Al_0.35_Si_0.65_O_2_) bilayer. XPS data are collected under UHV conditions after exposure to a mixture of noble gases (*n*_Ar_:*n*_Kr_:*n*_Xe_ = 1:1:1, with a total pressure of ∼2 mbar). The coverages (Θ) are calculated and displayed as a function of time and temperatures as indicated. Reproduced with permission from ref (308). Copyright 2019 WILEY-VCH Verlag GmbH & Co. KGaA, Weinheim.

The unprecedented ability of the 2D silicates to stabilize noble gas atoms heavier than Ne within their framework renders them promising candidates for potential applications with respect to gas storage and gas separation.^312^Figure 57 summarizes the data for trapping equimolar gas mixtures of Ar, Kr, and Xe in an aluminosilicate film with stoichiometry Al_0.35_Si_0.65_O_2_ at room temperature and its release as a function of temperature (top scale, which transforms into a time scale below via the used heating rate). The data points in Figure 57 have been deduced by evaluating the amount of the respective noble gas still remaining in the framework via XPS. The observation of a slightly higher coverage for Kr, initially, with respect to Ar, within the equimolar gas exposures discussed here, is in agreement with results from coverage dependences of individual noble gas adsorption experiments. As the temperature increases, Ar is released first, and at a temperature when Kr and Xe are still at a coverage of 81% and 89% of their initial coverages, respectively. When we compare Kr with Xe, Kr is fully released at 573 K, when 51% of the initial amount of Xe is still present. This clearly indicates that these silica-based materials have the potential to serve as noble gas separating systems. This is also consistent with observations deduced from simulated thermal desorption data for Ar, Kr, and Xe, which reveal peak desorption temperatures for pure silica and aluminosilicates of 351, 489, and 699 K and 387, 514, and 704 K, respectively. For Kr, data are available on the trapping capacity for a metal organic framework (MOF) material. The trapping capacity in the latter case is 13 wt % for Kr and is thus comparable to the one deduced here for Kr in aluminosilicate, i.e., 9 wt %.^300^

#### 4.3.3 Tuning the Permittivity of the Silica Films

**Figure 58:**
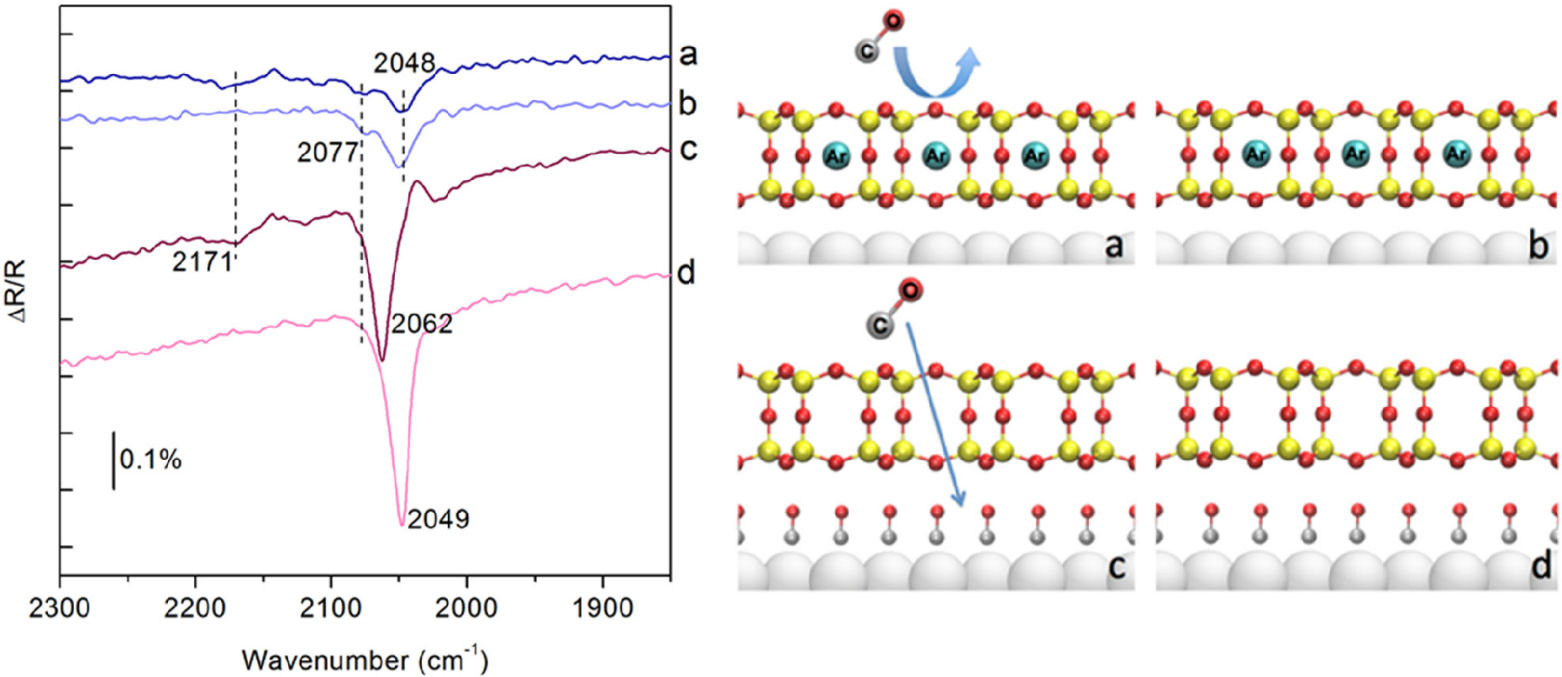
IRAS and schematic representation of O-poor Silica/Ru(0001) with trapped Ar (a) under 3 × 10^–3^ mbar CO at room temperature and (b) after pumping down the CO; and O-poor Silica/Ru(0001) without trapped Ar (c) under 3 × 10^–3^ mbar CO at room temperature and (d) after pumping down the CO. Reproduced with permission from ref (307). Copyright 2017 The Authors. Published by Springer Nature.

While previous work showed that 2D-silica bilayers are permeable to small molecules, such as H_2_, CO, and O_2_,^242,305,306^ the incorporation of noble gas atoms could allow the tuning of this permeability in a reversible manner by restricting the passage of small molecules through the nanocages. As shown in Figure 58, the tunable permeation of CO molecules has been demonstrated by IRAS. Figure 58a shows the Ar-trapped O-poor silica/Ru(0001) film under 3 × 10^–3^ mbar CO at 300 K. A very weak peak evident at 2171 cm^–1^, may be caused by the CO interactions with the silanol groups from the surface defects of the silica bilayer, which disappears when CO is evacuated. The peak at 2048 cm^–1^ is assigned to chemisorbed CO on Ru, and the small shoulder peak at 2077 cm^–1^ may be attributed to a small population of CO in the empty nanocage.

It is known that CO molecules can pass through the 6-membered ring and adsorb on the Ru(0001) surface with a small diffusion energy barrier of 0.5 eV. In comparison, the silica bilayer without trapped Ar reveals a much stronger peak at 2062 cm^–1^ under the same CO pressure condition, corresponding to the stretching vibration of CO with 2/3 monolayer coverage on the Ru(0001) surface below the silica bilayer.^306,313^ Interestingly, this mode shifts to 2049 cm^–1^ once CO is evacuated, indicating that some CO desorbs from Ru, and the CO coverage decreases to ∼0.5 monolayer.^305^ Clearly, the presence of Ar in the nanocages substantially reduces the permittivity of CO molecules and their adsorption on the Ru(0001) surface. Those experiments show that the 2D-silica bilayer is the thinnest molecular sieve so far discovered.

#### 4.3.4 Chemisorption of Xe at the Silica/Ru(0001) Interface

As discussed in section 3.3.1, the distance between the silica bilayer and the Ru(0001) support depends on the coverage of chemisorbed oxygen atoms on Ru(0001), which leads to an interfacial space of varying size and geometry. In section 4.3.2, we indicated that individual Xe atoms could be trapped at 300 K in this sub-nanometer interfacial space. Therefore, by tuning the interface distance, the strength of the Xe–Ru interaction may be successfully engineered. We discuss in the following the direct observation of room temperature *in vacuo* chemisorption of Xe atoms on Ru within the confined space at the silica/Ru(0001) interface, based on *in situ* XPS measurements and DFT calculations.^309^

**Figure 59:**
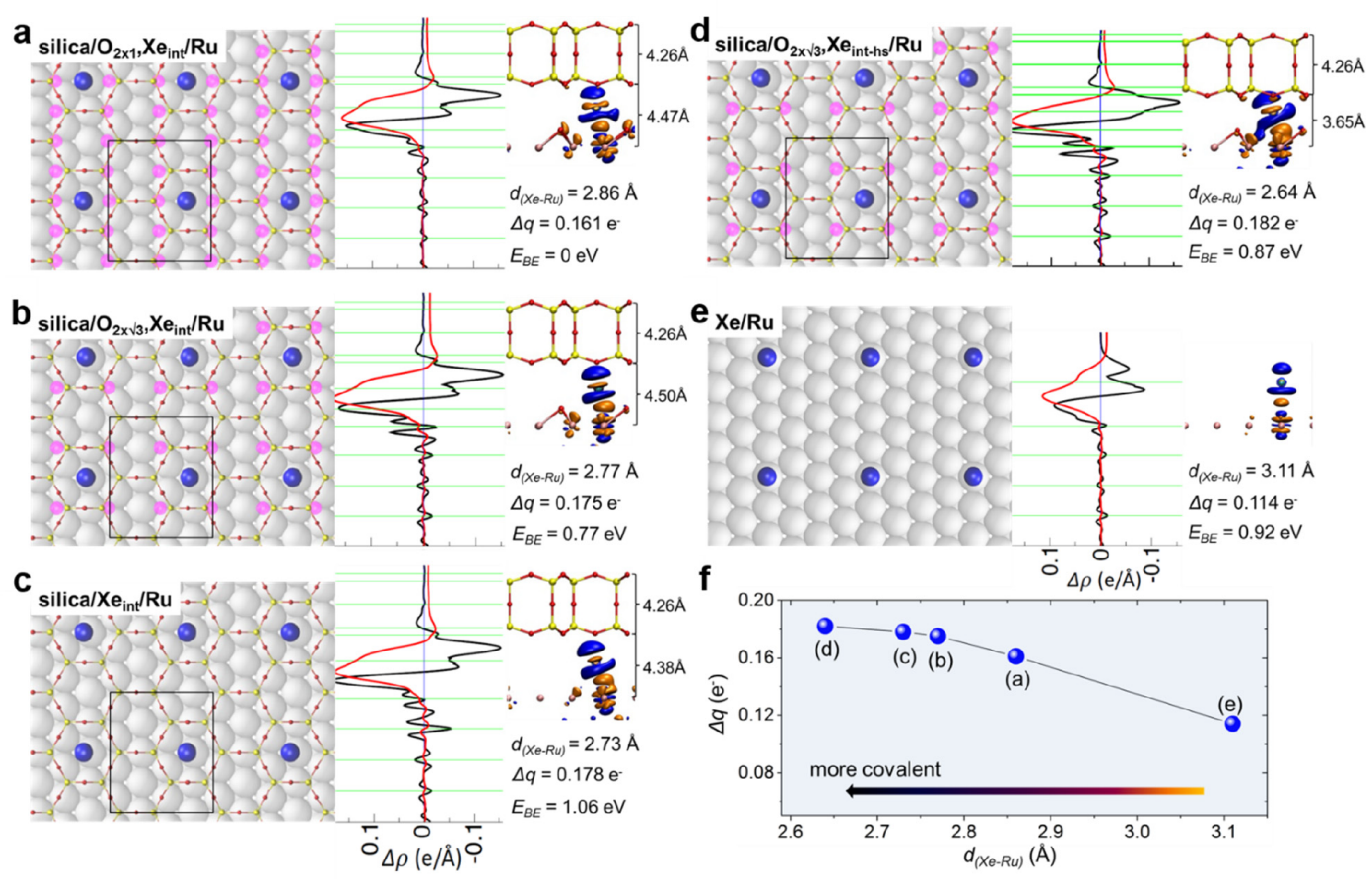
Relaxed structures of Xe_int_ [Θ(Xe_int_) = 0.25] adsorbed at the silica/Ru(0001) interface with (a) 0.5 ML O_int_ [silica/(O_2×1_, Xe_int_)/Ru(0001)], (b) 0.25 ML O_int_ [silica/(O_2×√3_, Xe_int_)/Ru(0001)], and (c) 0 ML O_int_ [silica/Xe_int_/Ru(0001)]. (d) Xe_int_ [Θ(Xe_int_) = 0.25] adsorbed at the silica/Ru(0001) interface with 0.25 ML O_int_ [silica/(O_2×√3_, Xe_int-hs_)/Ru(0001)] at a fixed interface distance of 3.65 Å. (e) Xe adsorbed on clean Ru(0001) [Xe/Ru(0001)]. The middle panels show the integrated charge density difference, and the right panels show the isosurface of the charge density difference (orange, electron accumulation; blue, electron depletion; and isovalue, 0.01 e/Å^3^). *E*_BE_ is the calculated Xe_int_ core-level binding energy relative to the Xe_int_ in silica/(O_2×1_, Xe_int_)/Ru(0001). Distances on the right correspond to the thickness of the silica film, *d*_*z*_(O_t_–O_b_), and the interface distance, *d*_*z*_(Ru–O_b_). (f) Charge transferred from each Xe atom to the Ru substrate (*Δq*) as a function of *d*_Xe–Ru_. Reproduced with permission from ref (309). Copyright 2019 American Chemical Society.

Xe, like all noble gases, is characterized by a relatively low chemical reactivity due to its stable electronic configuration with a full valence electron shell.^314^ The adsorption of Xe on metal surfaces is widely regarded as prototypical for a physisorption process via noncovalent, i.e., van der Waals interactions.^240^ Such a physisorption picture can be significantly changed once the Xe atoms are adsorbed on a Ru(0001) surface underneath a silica cover. As shown in Figure 59a–c, Xe atoms with an interfacial trapping coverage of Θ(Xe_int_) = 0.25 per cage are modeled via DFT calculations in three configurations, i.e., at the silica/Ru interfaces in the presence of varying coverages of chemisorbed oxygen atoms, i.e., 0.5 ML [silica/(O_2×1_, Xe_int_)/Ru(0001)], 0.25 ML [silica/(O_2×√3_, Xe_int_)/Ru(0001)], and 0 ML [silica/Xe_int_/Ru(0001)], respectively. After structure optimizations, it was found that there is a net charge transfer (*Δq*) of 0.161*e*, 0.175*e*, and 0.178*e*, respectively, from each Xe_int_ atom to the Ru substrate for these three theoretical models with corresponding equilibrium Xe_int_–Ru distances (*d*_Xe–Ru_) of 2.86, 2.77, and 2.73 Å, indicating stronger Xe_int_–Ru interaction due to the increased interfacial confinement.

In order to estimate the influence of the silica–Ru distance on the charge transfer between Xe and the metal substrate, an artificial model for a silica/(O_2×√3_, Xe_int_)/Ru(0001) with a reduced interface distance [*d*_*z*_(Ru–O_b_)] of 3.65 Å,^134^ as compared with 4.5 Å, has been investigated. The result is a reduced Xe–Ru separation (*d*_Xe–Ru_) of 2.64 Å and an increased charge transfer (*Δq*) of 0.182*e*. This is shown in Figure 59d. It is clear from the comparison of the various calculations, including the one on Xe interacting with pure R(0001) (Figure 59e),^315^ that the presence of the silica layer on top of the Xe/Ru system and its separation from the Ru substrate exert stress on the Xe atoms, influencing its bonding/interaction with the Ru substrate and charge transfer as revealed in Figure 59f. In Figure 59, the calculated numbers for XPS binding energy shifts for each of the situations are provided. Of course, the calculated adsorption energies also reflect the interplay between increased Xe–Ru hybridization and the penalty to be paid by pushing the Xe atoms toward the Ru, which finally leads to positive adsorption energies.^316^

## 5 Chemical Reactions on 2D-Silica

As a model system, 2D-silica allows us to study heterogeneous catalysis by making full use of its structural and chemical characteristics by applying the surface science toolkit. Taking its porosity, reactivity, and regularity, in particular, 2D-silica represents a perfect playground for fundamental studies of confinement effects, hydroxyl activity, and support effects in different chemical reactions. In this section, those three aspects will be discussed in detail.

### 5.1 Reactions in Confined Spaces

The influence of nanoscale confined spaces in catalytic reactions is well-known in the field of zeolites.^236,317^ Less known is the case of 2D confined nanospaces,^21^ which is especially true for the case of 2D zeolite models. For example, the attractive and repulsive interactions between the 2D-silica films and their transition metal supports can significantly affect the adsorption properties of the permeated gas molecules on catalytically active metal surfaces. Depending on the properties of reactants, 2D-silica may hinder its access or removal of products and thus change the rate-limiting steps. Confinement aspects in 2D catalytic systems need to be carefully considered in order to reach a rational understanding of the reaction kinetics.

#### 5.1.1 Oxidation and Reduction under Cover

**Figure 60:**
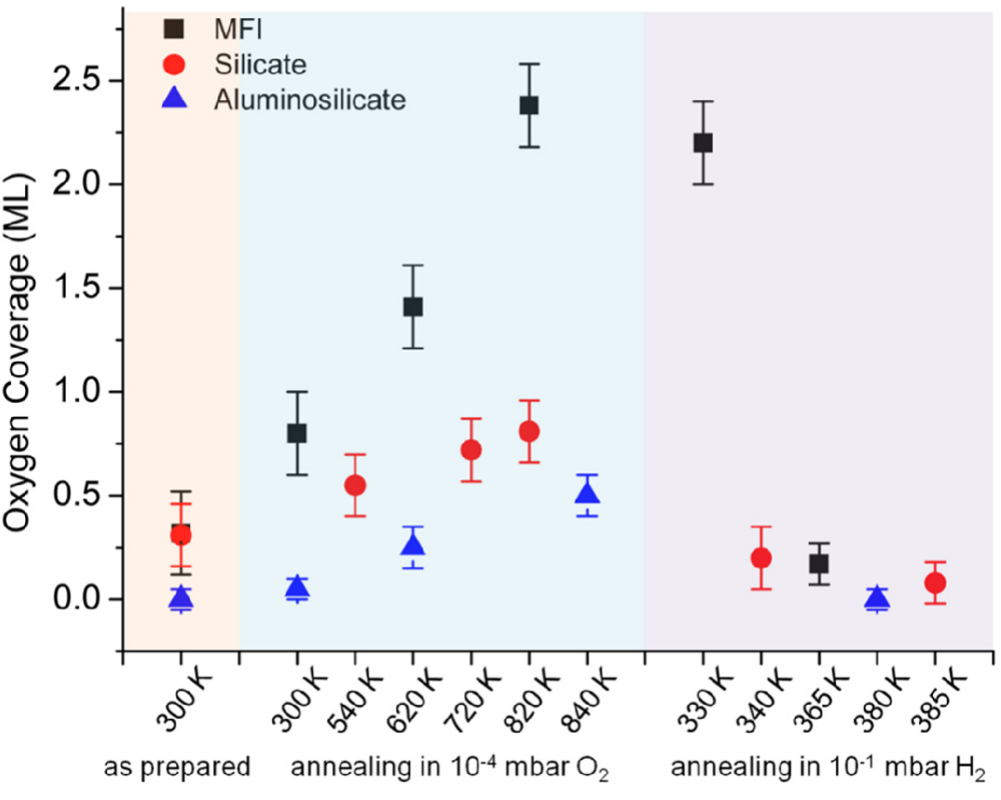
Coverages of the Ru-bound oxygen species as a function of temperature under O_2_ (green panel) and H_2_ (purple panel) for BL silica/Ru(0001), BL aluminosilicate/Ru(0001), and MFI nanosheets/Ru(0001). Reproduced with permission from ref (241). Copyright 2016 American Chemical Society.

The porous nature of silica bilayer films and their weak interactions with the metal support allow oxygen and hydrogen molecules to penetrate the nanopores and react with the metal support at the interface. Zhong et al. have studied the oxidation and reduction of Ru(0001) surfaces at the confined space between the 2D-silica framework and Ru(0001) with APXPS. Three types of 2D-silica frameworks, i.e., BL silica, BL aluminosilicate, and zeolite MFI nanosheets, have been investigated for their influence on the oxidation and reduction of the ruthenium surfaces at elevated pressures and temperatures (Figure 60).^241^ It was found that oxygen can readily penetrate through all studied frameworks and dissociate into atomic oxygen on the Ru(0001) surface. On BL silica/Ru(0001), it results in a ∼0.75 ML surface chemisorbed oxygen under ∼10^–4^ mbar O_2_ at 820 K. Note that APXPS experiments on bare Ru(0001) under the same conditions showed the formation of RuO_2_.^242^ For the BL aluminosilicate/Ru(0001), the surface oxidation of the ruthenium is further suppressed due to a smaller interface distance, which is caused by the Al substitution-induced additional electrostatic forces.^238^ In contrast, the Ru(0001) surface may be easily oxidized to RuO_2_ (equivalent to ∼2 ML surface chemisorbed oxygen) when covered by the MFI nanosheets, even though they have larger thicknesses of 3 nm as opposed to 0.5 nm for BL (alumino)silicate. The larger pore size or the different steric effects in the MFI frameworks may be responsible for the observed variations.

The removal of oxygen from the surface was followed by exposing it to 0.1 mbar H_2_. It is essential to mention that all Ru-bound oxygen is removed at low temperatures in the presence of H_2_, which is remarkable compared to the vacuum annealing of these films.^134^ This observation is connected to water formation,^318^ as will be discussed explicitly in section 5.1.2. It should be noted that 2D-silica is very stable and stays intact under these reaction conditions.

#### 5.1.2 Confinement Effects on the Water Formation Reaction

**Figure 61:**
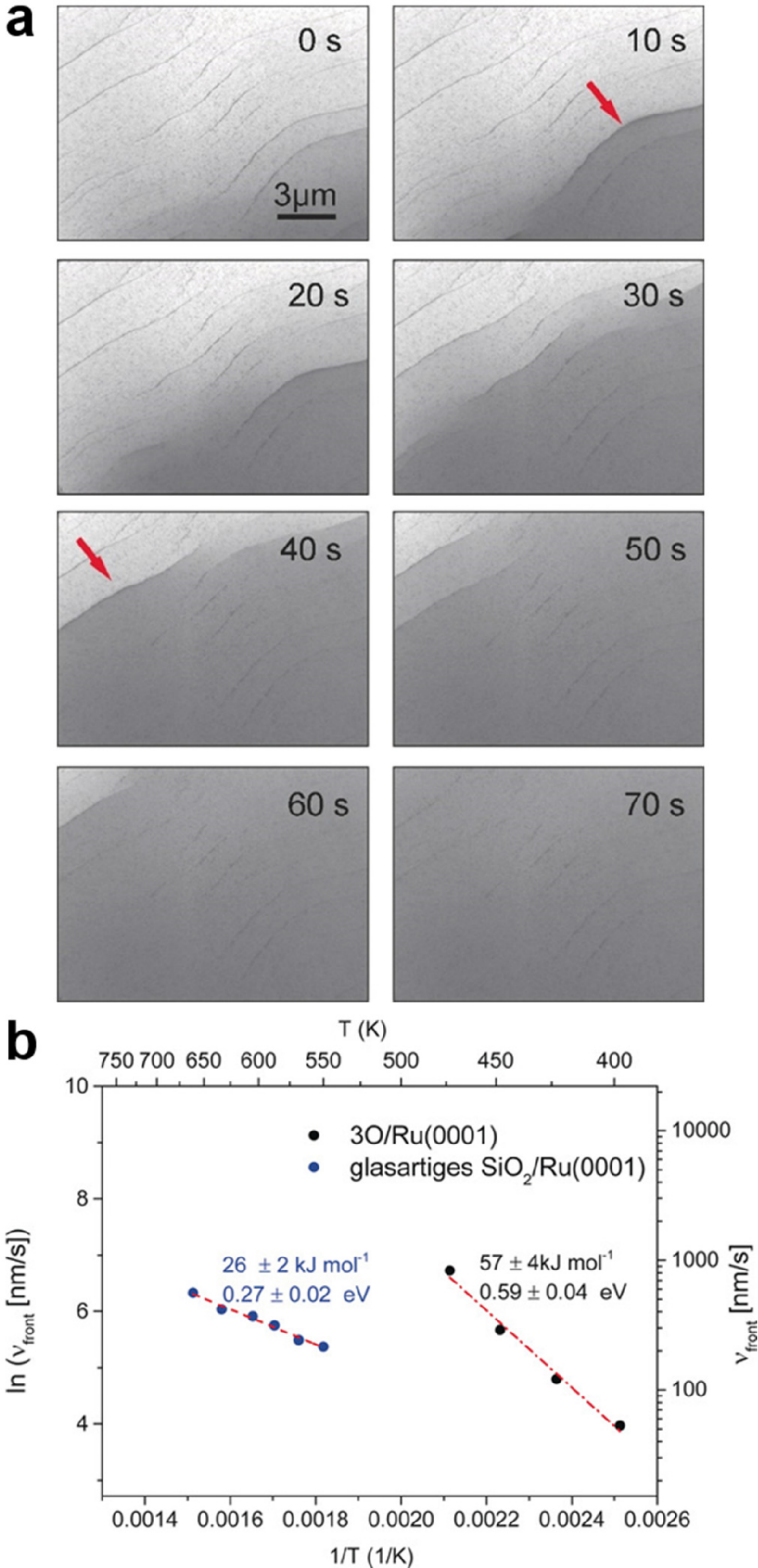
(a) Time evolution of the reaction front observed in LEEM during annealing silica/O/Ru(0001) in 1 × 10^–6^ mbar H_2_ at 550 K. *E*_kin_ = 14 eV. (b) Arrhenius plots of the temperature-dependent front velocities for silica/O/Ru(0001) (blue dots) and 3O/Ru(0001) (black dots). Reproduced with permission from ref (18). Copyright 2018 The Authors. Published by Wiley-VCH Verlag GmbH & Co. KGaA.

Prieto et al. first measured the apparent activation energy (*E*_a_^app^) in confined space by studying the H_2_ intercalation and its reaction with the Ru-bound oxygen atoms under the silica cover, i.e., the water formation reaction with respect to BL silica/O/Ru(0001).^18^ The estimated coverage of surface chemisorbed oxygen of the as-prepared 2D-silica system is 0.75 ML. Real-time LEEM is then used to monitor the kinetics of the water formation reaction by exposing it to 1 × 10^–6^ mbar H_2_ at various temperatures. As shown in Figure 61a, a reaction front appearing as a sudden change in LEEM images was seen propagating across the sample surface. According to the LEEM/PEEM-IV measurements, the bright side of the front corresponds to the unreacted area (O-rich), while the dark side of the front represents the reacted area (O-poor), which are deducted from the higher binding energy shifts of the core-levels and the reduced surface work functions that result from the removal of the Ru-bound oxygen atoms during the water formation reaction. Clearly, the reaction propagates by emptying oxygen sites. Such a reaction front is also seen on the bare Ru(0001) surface but with faster front velocity.

An Arrhenius analysis of the temperature-dependent front velocities yields the apparent activation energies (*E*_a_^app^) and reveals 0.27 ± 0.02 and 0.59 ± 0.04 eV for the silica-covered Ru surface and the bare Ru surface, respectively (Figure 61b). The *E*_a_^app^ for bare Ru agrees with the model proposed by Koch et al., where the reaction of H_ads_ + O_ads_ is the rate-limiting step.^319^ Since H_2_ cannot dissociate on a Ru surface exposing a complete oxygen coverage, e.g., a p(2 × 2)-3O/Ru(0001) structure (i.e., 0.75 ML), the reaction starts most likely at the defects sites, where local fluctuations of the oxygen coverage allow H_2_ dissociation, spreading the reaction across the entire sample surface. The obtained decreased *E*_a_^app^ for the silica-covered Ru surface suggests the necessity of a detailed evaluation of the reaction kinetics under interfacial confinement.

**Figure 62:**
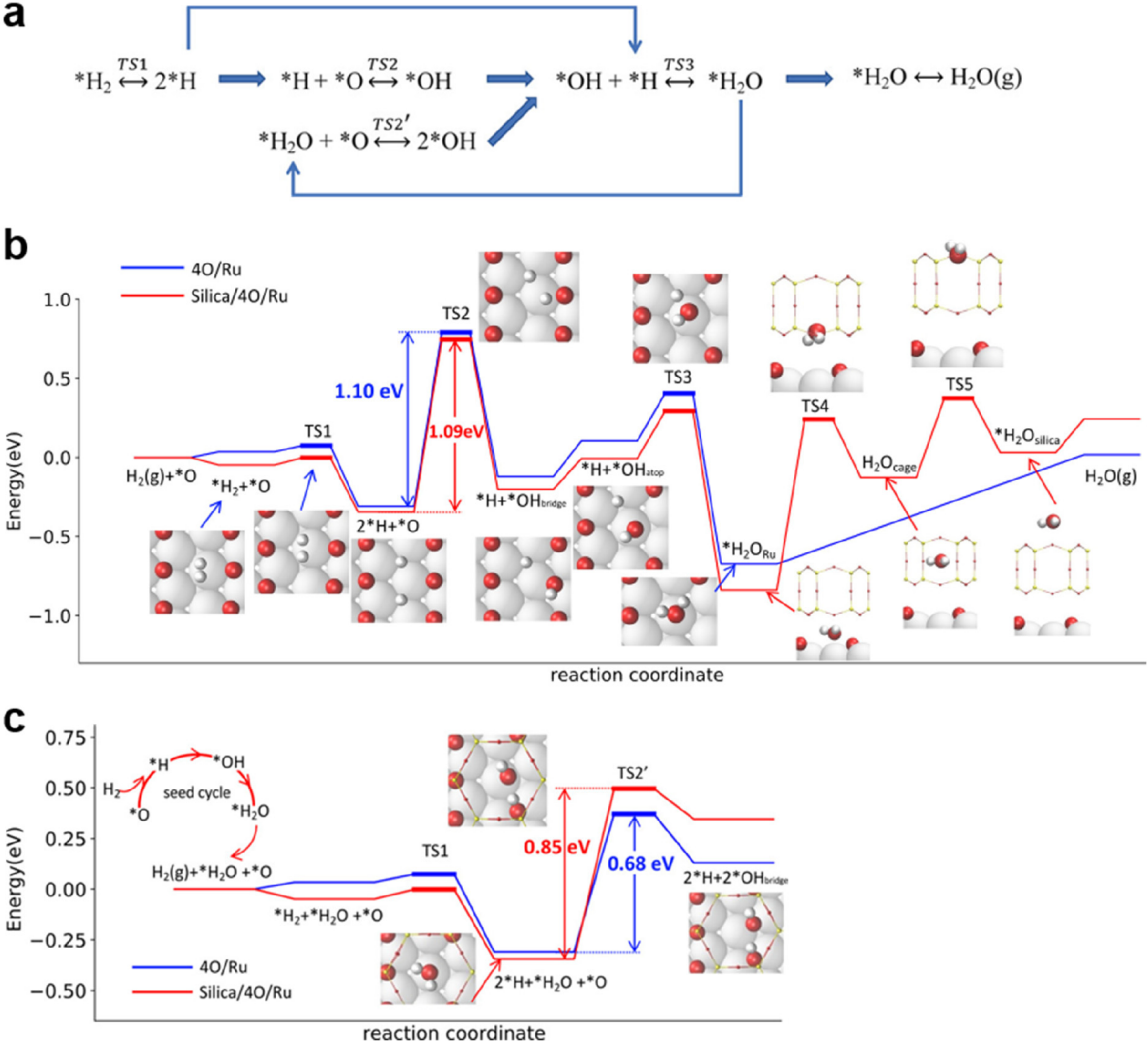
(a) Diagram of the dual-path water formation reaction on the Pt(111) surface. The adsorbed species on metal surfaces are marked by the asterisks (∗). (b) Potential energy diagram for the water formation reaction (via the first hydrogen addition) on the Ru(0001) surface (blue) and at the BL silica/Ru(0001) interface (red). (c) Potential energy diagram for the water formation reaction (via the disproportionation pathway) on the Ru(0001) surface (blue) and at the BL silica/Ru(0001) interface (red). Reproduced with permission from ref (322). Copyright 2020 American Chemical Society.

The overall water formation reaction under the silica cover is a complex process, where many parameters may play a role by either modifying existing reaction steps or adding new ones that are not present for reaction in a nonconfined space. Recently, Wang et al. investigated the energetics of the water formation reaction as well as the origin of its decreased *E*_a_^app^ in the silica/Ru system by using DFT calculations. In analogy to previous studies on Pt(111),^320,321^ the water formation reaction on bare Ru and silica/Ru may also follow a similar dual-path mechanism as shown in Figure 62a. In the rate-limiting step, the adsorbed hydroxyl groups (*OH) can be produced by a direct hydrogen addition (TS2) or a disproportionation pathway (TS2′). The potential energies for the water formation reaction on the Ru(0001) surface and at the silica/p(2 × 1)-O)/Ru(0001) interface are calculated as shown in Figure 62b. It is found that the adsorption geometry and dissociation pathway of *H_2_ (TS1), the first hydrogen addition step (TS2), and the second hydrogen addition step (TS3) are nearly the same in both cases. The interfacial confinement at the silica/Ru has little effect on *H_2_ dissociation and *H_2_O formation. However, as compared with a single desorption barrier of 0.68 eV on bare Ru(0001), the desorption of *H_2_O at the silica/Ru interface requires multiple activation steps, especially the step needed to overcome the penetration barrier (1.08 eV) of the bottom layer of the silica cage (TS4). In principle, the increased water desorption energy in silica/Ru includes contributions from the removal of *H_2_O from the Ru surface and the expansion of the silica cage.

Therefore, the silica bilayer can stabilize the *H_2_O and may increase its residence time at the interface, further resulting in a disproportionation pathway (TS2′ in Figure 62c). As shown in Figure 62c, the formed *H_2_O dissociates again, and the one H atom combines with a nearby *O atom, resulting in one *OH group sitting close to the atop site and the other *OH group located close to the bridge site. The activation energy barriers of the disproportionation pathway (TS2′) are 0.68 and 0.85 eV for bare Ru and silica/Ru, respectively. The slightly higher activation energy barrier at the silica/Ru interface mainly comes from the additional repulsive energy between the *OH group and the bilayer framework. Since the silica bilayer does not significantly change the activation energies of the individual reaction steps (TS1–TS3), Wang et al. thus concluded that the lower apparent activation energies (*E*_a_^app^) measured in the experiment for the water formation reaction at the silica/Ru interface may result from a change of the reaction pathway, i.e., in favor of the disproportionation pathway in silica/Ru. For example, at experimental conditions (e.g., above 400 K), the formation of *OH (i.e., the rate-limiting step in water reaction) on the Ru surface is dominated by the first hydrogen addition with an activation energy of 1.10 eV (TS2 in Figure 62b). At the silica/Ru interface, due to the confinement effect, the *OH is primarily formed from a disproportionation pathway with an activation energy of 0.85 eV (TS2′ in Figure 62c), i.e., 0.25 eV lower than that of the direct path on bare Ru. The decrease in activation energy barrier of the rate-limiting step is in line with the reduction in apparent activation energies (*E*_a_^app^) in the experiments.^18,322^ However, it should be noted that both the higher H_2_ pressure and the lower temperature may play roles in the enhancement of the disproportionation pathway over the direct hydrogenation pathway in the silica/Ru system. Similar spatial confinement effects were also observed in reactions within 3D materials that resulted in halving the activation energy.^323^

**Figure 63:**
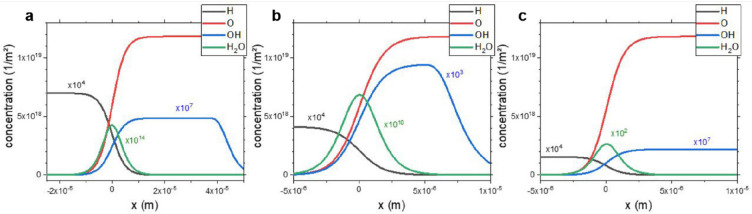
Spatiotemporal surface concentration profiles of the H_ads_, O_ads_, OH_ads_, and H_2_O_ads_ species involved in the water formation reaction were obtained from the numerical simulations (a) on bare Ru(0001), (b) in confinement under a constrained silica bilayer, and (c) in confinement under an optimized silica bilayer. Reproduced with permission from ref (324). Copyright 2021 The Authors. Published by American Chemical Society.

However, very recently, Prieto et al. came up with another conclusion that it is the H_2_ adsorption process rather than the water desorption that plays a decisive role in the observed decreased apparent activation energies (*E*_a_^app^) in the experiments.^324^ According to their DFT simulations, the rate-limiting step (i.e., the OH formation) remains unchanged upon the spatial confinement in a silica/Ru system. Thus, they rule out the widely accepted transition state effect observed in zeolites, where stabilization of the transition complex is responsible for the changes in reactivity.^325−327^ Moreover, they evaluated the kinetic aspects of the global water formation reaction by using the kinetic constants for each elementary step and the diffusion constant for H on Ru(0001), from which the spatiotemporal dependence of the surface concentration of all species (H_ads_, O_ads_, OH_ads_, and H_2_O_ads_) involved in the water formation reaction can be modeled as shown in Figure 63.

These models successfully reproduce the reaction fronts on the bare Ru and silica-covered Ru surfaces (e.g., the reaction front moves slower under confinement). The active area is found to be the region in the vicinity of the moving reaction front, where there are the highest concentrations of OH_ads_ and H_2_O_ads_. As shown in Figure 63, the presence of OH_ads_ can extend over a broad area for both nonconfined and confined cases, suggesting that the H_ads_ may be able to diffuse to the unreacted side of the front (e.g., the 3O–Ru region). Prieto et al. thus concluded that the dissociative adsorption of H_2_ and the formation of OH_ads_ are the two most important steps. For example, when the H_2_ adsorption process is fast enough, plenty of H_ads_ is available on the Ru surface to propagate the reaction front (e.g., in unconfined reactions). In contrast, when the H_2_ adsorption process is slow compared to the formation of OH_ads_, the H_2_ adsorption process will be the limiting factor (e.g., in confined reactions).

It is important to point out that the work by Wang et al. was conducted at much higher H_2_ pressures (∼0.1 mbar, i.e., 5 orders of magnitude higher than in the case of Prieto et al.), where plenty of H_2_O_ads_ may be responsible for blocking the active sites for H adsorption and diffusion. These studies by Wang et al. and Prieto et al. provide important implications for the chemical reaction pathways in the nanoporous catalytic systems. However, much more detailed investigations are still needed.

#### 5.1.3 Confinement Effects on the Selectivity of the Furfuryl Alcohol Reactions

**Figure 64:**
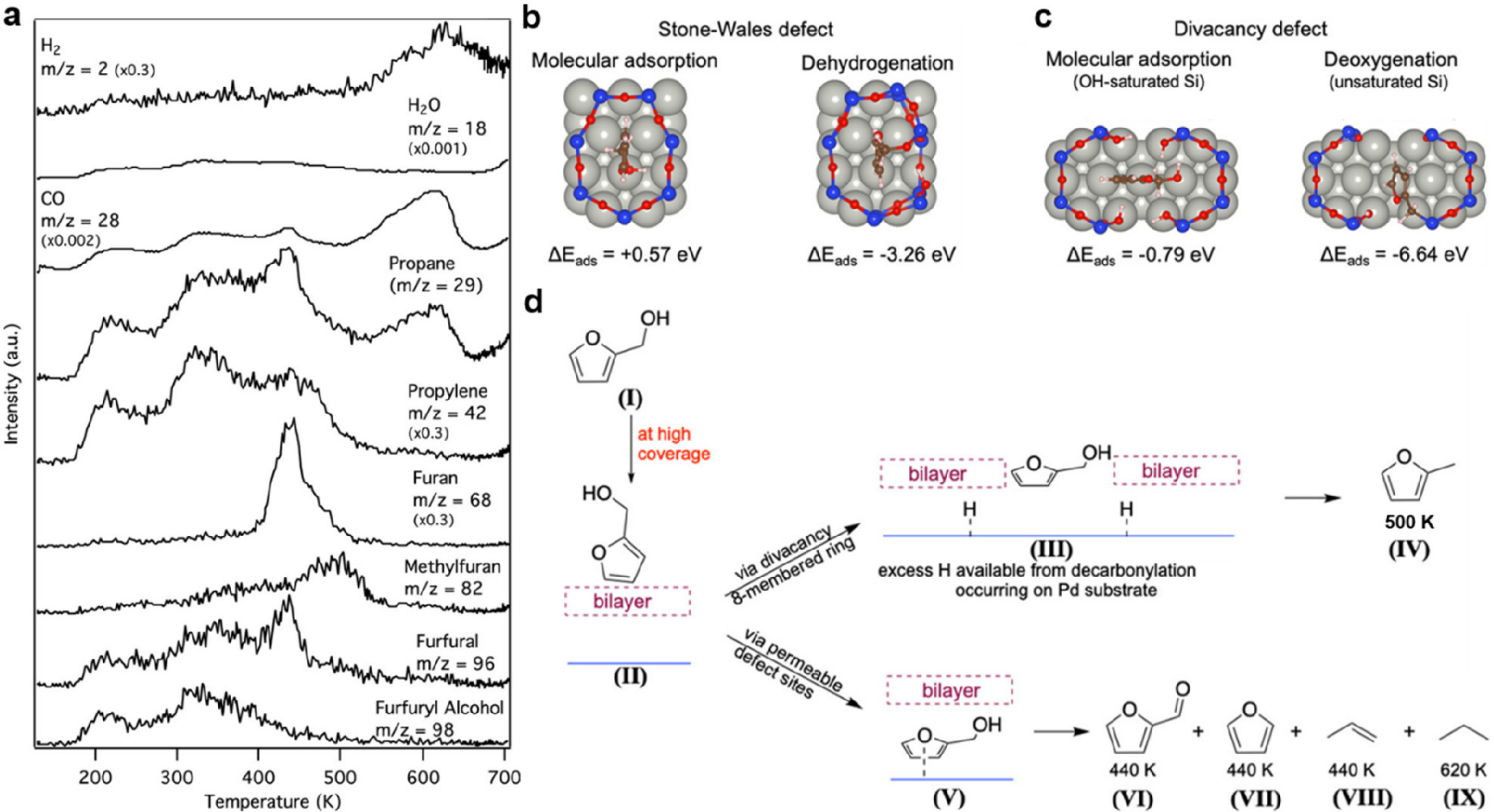
(a) Thermal desorption spectra of furfuryl alcohol on BL silica/Pd(111) films. (b) DFT-optimized structures for furfuryl alcohol in a 7-membered ring representing a Stone–Wales defect, which showed endothermic adsorption (left) and exothermic dehydrogenation (right). (c) DFT-optimized structures for furfuryl alcohol in an 8-membered ring of a divacancy defect, which showed exothermic adsorption in the OH-saturated defect (left) and deoxygenation in the unsaturated defect with dangling Si (right). (d) Proposed reaction mechanism for furfuryl alcohol on BL silica/Pd(111) films. Reproduced with permission from ref (329). Copyright 2020 American Chemical Society.

Generally, the confinement effects mainly influence the access of the reactants to the reaction sites and the release of the products from the reaction sites.^328^ The small pore size of the crystalline silica bilayer (∼5 Å) can prevent bulkier molecules (e.g., aromatic molecules) from approaching its metal support. However, the vitreous silica film usually contains bigger pores with diameters up to 1 nm, allowing chemical reactions of larger molecules at the confined silica/Ru interfaces. Mark et al. have studied the effects of interfacial confinement on furfuryl alcohol reactions in the BL silica/Pd systems.^329^ Possible dehydrogenation, decarbonylation, decomposition, and hydrogenation of furfuryl alcohol have been explored and discussed using multimodal approaches (TPD, HREELS, IRAS, and DFT). The reaction products of furfuryl alcohol on BL silica/Pd were inferred from the TPD studies. As shown in Figure 64a, the molecular desorption of furfuryl alcohol (*m*/*z* = 98) was detected at ∼215 K for multilayer and ∼325 K for monolayer. Dehydrogenation of furfuryl alcohol produced furfural (*m*/*z* = 96) at ∼440 K. Furfuryl alcohol also underwent deoxygenation and decarbonylation, which produced methylfuran (*m*/*z* = 82) and furan (*m*/*z* = 68) at ∼500 and ∼440 K, respectively. The ring decomposition of furfuryl alcohol produced propylene (*m*/*z* = 42) at ∼440 K. Similar products are also formed but at relatively lower (by 100 K) temperatures for the furfuryl alcohol reaction on bare Pd(111).^330^ The main difference on silica/Pd is the formation of propane (*m*/*z* = 29) at ∼620 K, which was not observed on bare Pd.^330^ The above-proposed reactions are summarized in Figure 64d (e.g., **IV** for deoxygenation, **VI** for dehydrogenation, **VII** for decarbonylation, and **VIII** for ring decomposition).

According to the DFT calculations, furfuryl alcohol was found to bind weakly on the surface of silica film (**II** in Figure 64d) and at the interface of silica/Pd (**V** in Figure 64d) with an adsorption energy of −0.38 and −0.78 eV, respectively, which are significantly smaller (by ∼1 eV) than those on bare Pd(111). By considering the adsorption at vitreous silica regions, two representative types of defects (i.e., Stone–Wales defect with the 7-membered ring and divacancy defect with the 8-membered ring) were computed for comparison (Figure 64b,c). It was found that the upright furfuryl alcohol had an adsorption energy of 0.57 eV at the Stone–Wales defect, which can be dehydrogenated and result in a more stable intermediate within the pore (Figure 64b). In contrast, the furfuryl alcohol at the divacancy defect can be deoxygenated (Figure 64c). It should be noted that varying adsorption configurations and adsorption energies may be expected for furfuryl alcohol at different defect sites.

Since the furfuryl alcohol would have molecularly desorbed below 400 K, it was then concluded that the furfuryl alcohol reactions were catalyzed by the Pd(111) surface at the interface (**V** in Figure 64d) or within the silica pores (**III** in Figure 64d). Larger ring sizes, film defects, and edge defects are responsible for the permeation of the furfuryl alcohol to the Pd support. Due to the presence of the silica cover, furfuryl alcohol may undergo different decomposition reactions. For example, methylfuran formation is reduced to 7% of the C3+ products, from more than 20% on bare Pd(111). Moreover, the additional product, propane, was hypothesized to be formed by intermediates that become trapped in confined sites under or within the film. The overall effect of the porous silica bilayer demonstrated in this study, therefore, can be used for selective hydrogenation of multifunctional molecules, such as converting furfural to furfuryl alcohol.

### 5.2 Reactions on Hydroxyls Groups

The aluminosilicate bilayer that exposes bridging hydroxyl groups has been demonstrated as a model system for surface science studies of chemical processes catalyzed by the Brønsted acid sites (see section 3.2.3). This allows us to do fundamental investigations on some of the most important reactions in the industry, such as the cracking of crude oil, methanol to gasoline conversion, and olefin oligomerization, from which detailed information about active sites and elementary reaction steps can be obtained for the atomistic understanding of catalysis. For example, the catalytic cracking of hydrocarbons usually involves alkane activation, C–C bond cleavage, and dehydrogenation. However, even for the simplest reaction that involves C–H bond activation, such as the isotopic exchange of alkanes with the SiO(H)Al Brønsted site, it is still under debate whether it occurs directly via carbonium-type transition structures^331^ or indirectly via hydride transfer.^332,333^ Sauer and co-workers have developed a series of hybrid quantum mechanics calculations to examine various mechanistic proposals.^232,236,334−336^

#### 5.2.1 Proton Exchange Barriers for Alkanes at Brønsted Sites

**Figure 65:**
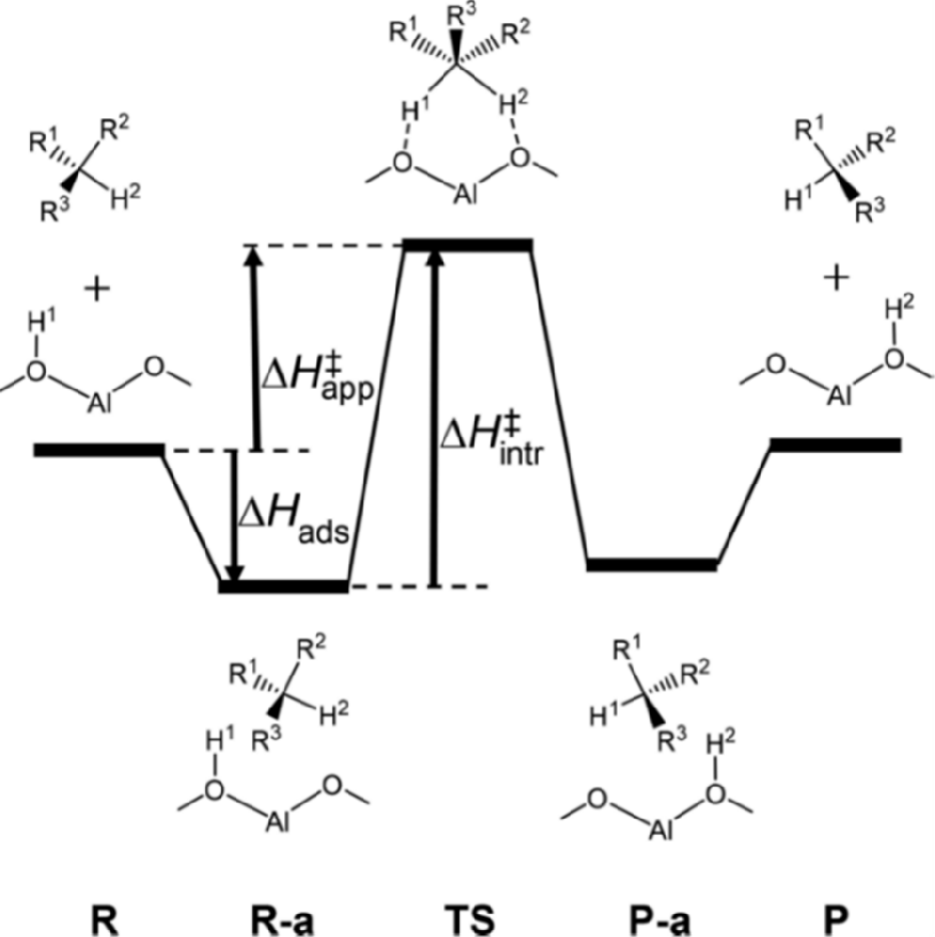
Structures involved in the proton exchange between alkanes and Brønsted sites. R1, R2, and R3 are alkyl groups or hydrogen atoms. Reproduced with permission from ref (336). Copyright 2018 American Chemical Society.

Figure 65 shows the mechanism of the direct proton exchange,^336^ in which the proton of the Brønsted site is directly transferred to the alkane molecule while one of the protons in the alkane is transferred back to the Brønsted site simultaneously. Rybicki et al. have employed a hybrid of high-level and low-level quantum mechanics methods to predict the intrinsic (Δ*H*_intr_^⧧^) and apparent (Δ*H*_app_^⧧^) energy barriers for such direct proton exchange reactions of alkanes (methane, ethane, propane, *n*-butane, and *i*-butane) at Brønsted sites of zeolite H-MFI (i.e., ZSM-5, an aluminosilicate zeolite that contains well-defined and interconnected pores and channels).^336^ It was found that while the intrinsic enthalpy barriers remain constant around 124–127 kJ/mol (1.285–1.316 eV) at 500 K, the apparent enthalpy barriers decrease with increasing carbon number from 104 to 63 kJ/mol (1.078–0.653 eV) as accompanied by the decreasing heat of adsorption (Δ*H*_ads_). The predictions are consistent with experimental results for methane,^193,337,338^ ethane,^339^ propane,^340,341^ and *n*-butane^342,343^ but not for *i*-butane,^332,333,344^ suggesting that the direct exchange mechanism is not operative for the proton exchange reaction of *i*-butane. However, the indirect hydride transfer mechanism that involves two proton transfer steps (i.e., the Brønsted proton is transferred to an alkane molecule, and then, the hydride ion is transferred from the nearby alkane to the alkyl cation) was also excluded for the *i*-butane according to the hybrid quantum mechanics calculations. Therefore, it was suspected that extra-framework aluminum species might play a role in the catalytic proton exchange for the *i*-butane.

#### 5.2.2 Methanol and Ethanol Adsorption on Brønsted Sites

**Figure 66:**
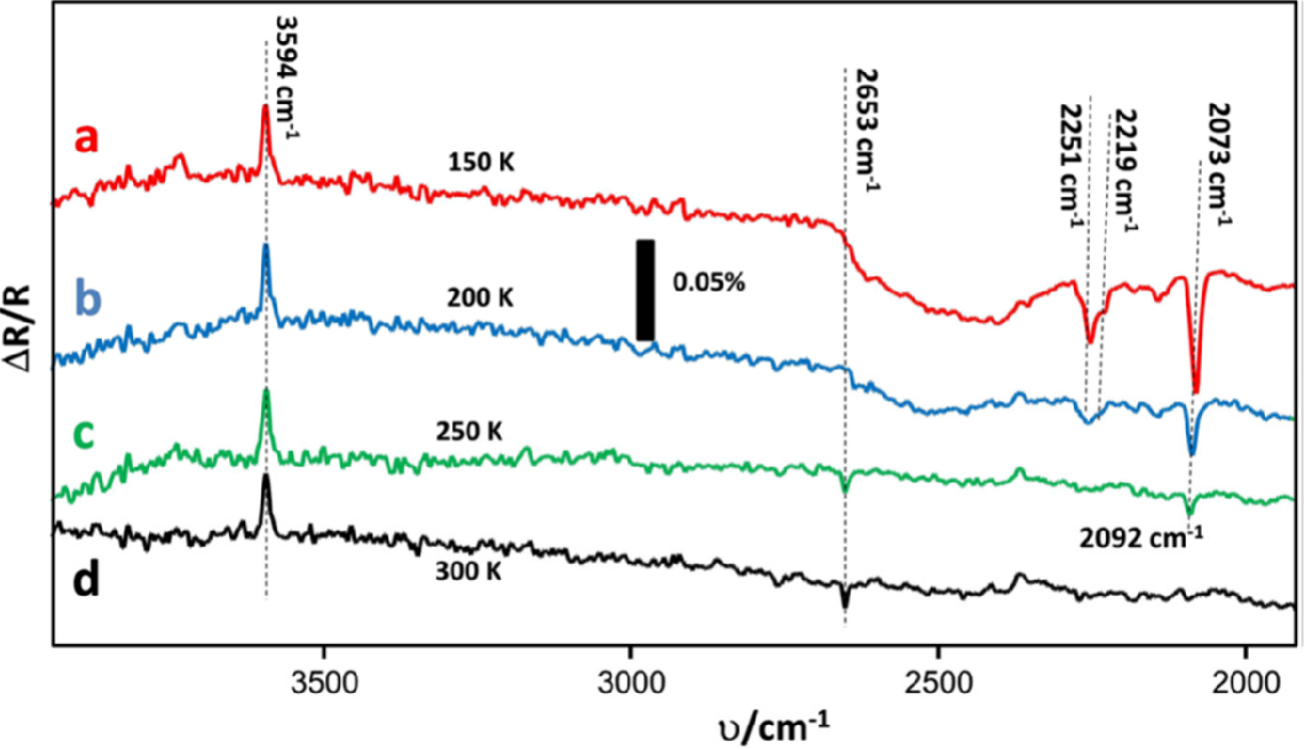
IRAS of methanol (CD_3_OD) adsorbed on bridging hydroxyls on BL aluminosilicates/Ru(0001). (a–d) CD_3_OD was adsorbed at 100 K, and then, the film was heated to the temperatures as indicated in each spectrum. The spectrum is referenced to the spectrum taken before the CD_3_OD exposure. Therefore, the peak at 3594 cm^–1^ corresponds to the consumed bridging OH groups, while the peak at 2653 cm^–1^ corresponds to the formed bridging OD groups upon the H/D exchange reaction. The broad features between 2300 and 2600 cm^–1^ in panels a and b result from hydrogen-bonded OD groups. The peaks at 2251, 2219, and 2073 cm^–1^ in panels a–c correspond to the vibrations in CD_3_OD. Reproduced with permission from ref (197). Copyright 2014 Springer Science Business Media New York.

Hydrocarbon synthesis from methanol is another important catalytic reaction in the zeolite industry. Intensive studies were focused on the mechanism of the first C–C bond formation during the methanol to gasoline methanol to olefin processes.^345^ For example, there are controversies regarding whether the methanol was protonated or not during its adsorption on the Brønsted site. Currently, the discussion tends to agree that the transfer of the proton from the Brønsted site to methanol molecule only occurs when there are two hydrogen-bonded methanol molecules at the active site,^346^ which was attributed to the larger proton affinity of the methanol dimer rather than that of the monomer in H-ZSM-5. For BL aluminosilicate/Ru(0001) model systems (Figure 66), it was found that the interaction of methanol (CD_3_OD) with bridging hydroxyl is accompanied by the H/D exchange reaction.^197^ According to the studies of CD_3_OD on H-chabazite, the hydrogens originating from the methanol hydroxyl and the bridging hydroxyl are indistinguishable in the adsorption complex.^347^

In numerous experimental studies on the interaction of methanol/ethanol with Brønsted sites in a zeolite (e.g., H-ZSM-5), the heat of adsorption varies widely, ranging from −115 to −65 kJ/mol (i.e., −1.192 to −0.674 eV) for methanol and from −130 to −90 kJ/mol (i.e., −1.347 to −933 eV) for ethanol, respectively.^348−350^ These huge differences may stem from the use of different experimental methods (e.g., calorimetry and TPD) and samples (e.g., different Si/Al ratios, crystalline structures, and sizes). From the perspective of theoretical calculations, such as the standard approach by using DFT with the inclusion of dispersion contributions (DFT + dispersion), the calculated adsorption enthalpies of methanol and ethanol on bridging hydroxyl of H-MFI at 300 K are −117 and −135 kJ/mol (i.e., −1.213 and −1.399 eV), respectively, while the usage of the hybrid quantum mechanics scheme (hybrid MP2/PBE+D2+ΔCCSD(T)] yields smaller adsorption enthalpies of −84 and −104 kJ/mol (i.e., −0.871 and −1.078 eV) for methanol and ethanol, respectively.^335^ Nevertheless, more work, both computational and experimental, is needed to further address the adsorption of single methanol/ethanol molecules on Brønsted sites with surface heterogeneities.

#### 5.2.3 Ethylene Oligomerization on Chromyl Species on Silica Hydroxyls

**Figure 67:**
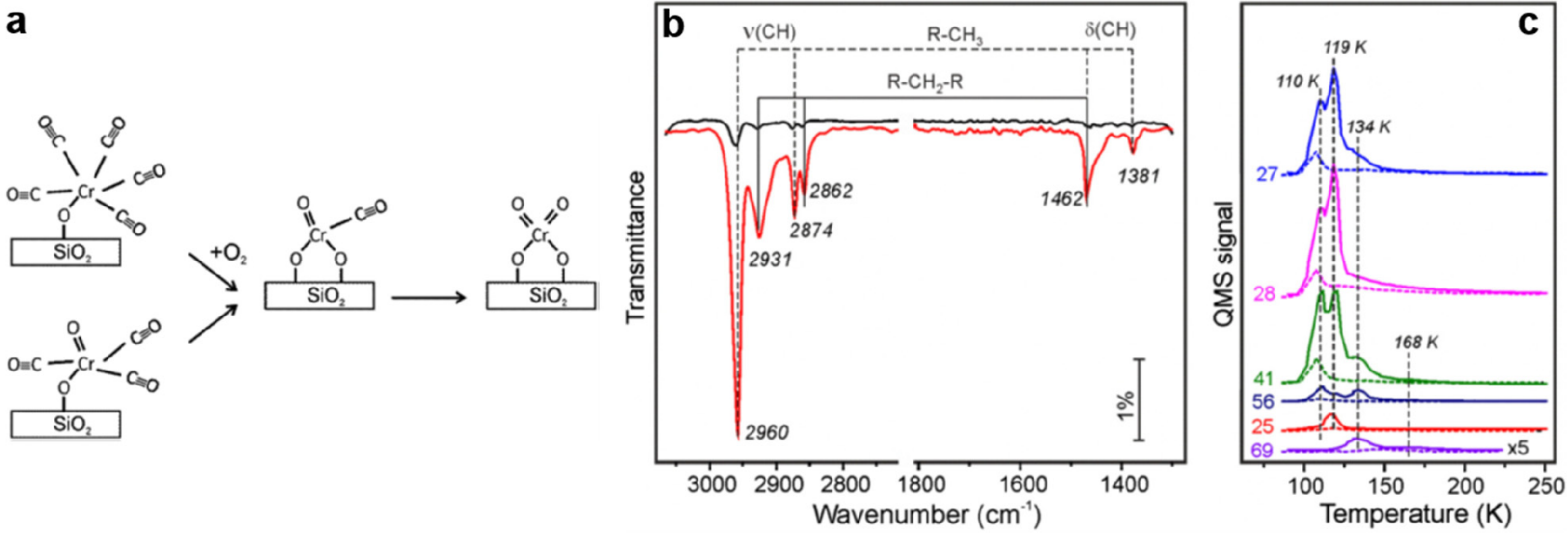
(a) Schematic diagram for monoxo and dioxo chromyl species formation on a hydroxylated silica bilayer as probed by CO adsorptions. (b) IRAS of C_2_H_4_ adsorbed on the pristine hydroxylated silica bilayer (black) and the “as-deposited” Cr/silica (red) at 85 K, respectively. (c) TPD of selected masses of C_2_H_4_ adsorbed on the pristine hydroxylated silica bilayer (dashed lines) and the “as-deposited” Cr/silica (solid lines) at 85 K, respectively. The heating rate is 3 K/s. Reproduced with permission from ref (351). Copyright 2017 Elsevier B.V.

In addition to their catalytic activity, the hydroxyl groups can also act as anchoring sites to other active metal atoms. Pan et al. have systematically studied the Phillips catalyst (Cr/SiO_2_) by depositing Cr on hydroxylated BL silica/Ru(0001) surfaces.^351,352^ The Phillips catalyst is industrially important in the large-scale production of polyethylene,^353^ which is commonly prepared by impregnating high-surface-area silica gel with chromium compounds (e.g., CrO_3_) and subsequently calcining in the air (or oxygen) to form an active catalyst (e.g., Cr(VI) species).^354^ Due to the structural complexity and surface heterogeneity of the Cr/SiO_2_, the atomic structure of the active sites and the reaction mechanism remain controversial. Previous studies employing planar model systems with Cr deposited on SiO_2_/Si(100) wafers showed that the isolated Cr sites are the most active.^355,356^ Pan et al. demonstrated that the Cr atoms were anchored by the surface hydroxyls and resulted in chromyl (Cr=O) species on the silica bilayer according to the IRAS results, which were stable up to at least 400 K. CO titration experiments were further used to study the oxidation state of these Cr species. It was found that the as-deposited Cr/silica also contains “naked” Cr in addition to Cr=O (as a minority), both of which can be oxidized and transformed into monoxo and dioxo chromyl species in ambient oxygen at elevated temperatures (up to 400 K at ∼10^–5^ mbar O_2_) (Figure 67a).

The ethylene was dosed onto both the pristine hydroxylated silica bilayer and the “as-deposited” Cr/silica at 85 K as shown in Figure 67b. The adsorption and reaction of ethylene solely occur on chromyl species. Specifically, the observed bands at 2960 and 2874 cm^–1^ and at 1462 and 1381 cm^–1^ were assigned to ν(C—H) and δ(C—H) vibrations in R—CH_3_ groups, respectively, while the bands at 2931, 2862, and 1462 cm^–1^ were characteristic for C—H vibrations in R—CH_2_—R.^357^ It should be noted that the absence of the ν(C=C) band could be explained by the surface selection rules assuming that the C=C bond is parallel to the surface. The IRAS results of ethylene on Cr/silica suggested the adsorption of butane/hexane molecules or the formation of butadiyl species.^358^ The ethylene oligomerization was further confirmed by the TPD results (Figure 67c). The masses (*m*/*z*^+^) solely for ethylene (i.e., 25) and for other alkenes/alkanes (e.g., 27, 28, 41, 56, and 69, which are the common fragments in TPD for most alkenes/alkanes) were analyzed. The desorption peaks at 110 and 119 K can be assigned to butene (formed from butadiyl species) and a mixture of ethylene and butane, respectively. However, the assignments of other species became difficult solely based on the desorption results, which needed more experimental evidence. Nevertheless, the formation of C4 molecules as the main result of ethylene oligomerization may follow the two-step initiation mechanism proposed by Brown et al.^359^ Moreover, the relatively high stability of the Cr/silica allows further investigations of ethylene polymerization under more realistic conditions.

### 5.3 2D-Silica as Catalyst Support

Catalytic metal/metal oxides nanoparticles (NPs) prepared on 2D-silica can help to link the reactivity studies between the well-defined single crystal surfaces and realistic powder catalysts, representing advanced model systems that can provide insights into the detailed reaction mechanisms, such as the role of particle size, particle morphology, alloy composition, the support effects, etc. These 2D-silica-supported NPs can be prepared via vapor deposition methods, which allow many of the traditional as well as the newly developed surface science techniques.^360^ In this section, the physical and catalytic properties of some 2D-silica supported NPs will be reviewed, aiming to demonstrate the suitability of the 2D-silica-based model catalysts for advanced studies of the structure–activity relationships under both UHV and elevated pressure conditions.^361^

#### 5.3.1 Rh and Pt NPs on Silica/Mo(112) for CO Oxidation

**Figure 68:**
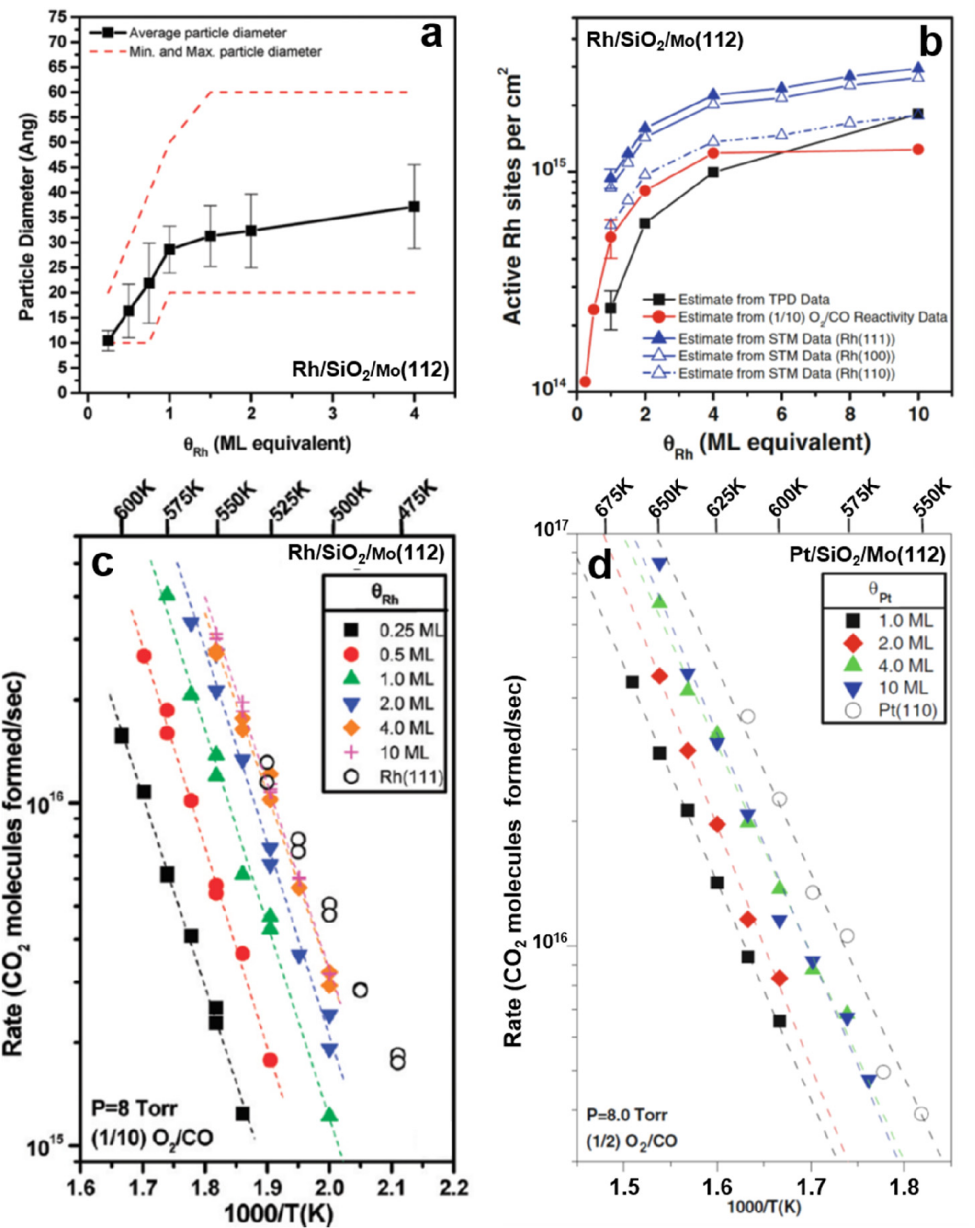
(a) Average particle size versus Rh coverage on silica/Mo(112) as determined from the STM measurements. (b) Number of active Rh sites per cm^2^ versus coverages estimated from CO TPD data, reactivity data, and STM data. (c, d) CO_2_ formation rate versus 1000/*T* for Rh and Pt on silica/Mo(112) surfaces with various coverages, respectively. CO_2_ reaction rate data obtained on Rh(111) and Pt(110) surfaces were also conducted under the same reaction conditions. Reproduced with permission from refs (362 and 364). Copyright 2009 American Chemical Society. Copyright 2009 Spring Science Business Media, LLC.

Oxide-supported Rh and Pt NPs have been widely studied due to their fundamental and practical importance. Regarding CO oxidation kinetics, much qualitative/quantitative agreement regarding reaction rates, activation energies, and the number of active sites in NPs has been achieved, which can be a valuable benchmark for future studies of more complicated reaction systems. For example, Goodman and co-workers have systematically investigated the catalytic properties of the planar model catalysts consisting of Pt-group metal NPs on silica/Mo(112) using both UHV surface analytical and near atmospheric pressure probe reactions.^360,362−365^ The STM studies enable the characterization of the NP size distributions and the estimation of active surface areas (based on simple geometrical models^366^) as a function of coverage (θ_ML_). By quantifying the total active sites on model catalytic surfaces and their reactivity data, the turnover frequency (i.e., TOF, a standard metric used for comparison of reactivity data) can be achieved and therefore can be correlated to the understanding of the structure–reactivity relationship under various reaction conditions. CO TPD and CO IRAS are also helpful methods for the characterization of different surface sites present on the NPs due to the sensitivity of the CO binding configurations on Pt group metals (e.g., bound to single or multiple metal sites, and specifically, such as the undercoordinated “steplike” site and the coordinated “terracelike” site). The results of these exercises for Rh NPs are illustrated in Figure 68a,b. (Note that the Pt NPs have similar results.) Usually, the surface fraction of undercoordinated sites increases rapidly with decreasing NP size below 5 nm, which often plays a critical role in dictating the observed activity and selectively of different catalytic reactions as will be discussed below.

It was well-known that CO oxidation exhibits structure-insensitive reaction kinetics under CO dominant reaction conditions (moderate temperatures of 450–650 K and high CO/O_2_ ratios) on both Rh^367^ and Pt^368^ surfaces with particle sizes larger than 2 nm. Figure 68c,d shows the CO_2_ reactivity measurements on a series of Rh/silica and Pt/silica model surfaces of varying coverages. The elevated pressure (8 Torr) reactivity measurements were conducted in a batch reactor mode by transferring the model surfaces *in situ* into the reactor cell. The reaction rates were measured by baratron gauge or gas chromatography. The reactivity measurements were also conducted on Rh(111) and Pt(110) single crystals for direct comparison. As expected, it shows similar activation energies of ∼110 kJ/mol (1.14 eV) for Rh/silica and Pt/silica as inferred from the analysis of the Arrhenius plots regardless of the coverages and morphologies. The activation energies are close to the CO desorption energies on Pt-group metal surfaces,^369^ indicating that the reactivity is limited by the CO desorption step as CO blocks sites for O_2_ adsorption and dissociation, with reaction kinetics reflecting traditional Langmuir–Hinshelwood behavior.^370^ Therefore, under CO-rich reaction conditions, the estimations of the active sites from the elevated-pressure CO oxidation measurements exhibit a general correlation with the low-pressure CO TPD and UHV STM measurements (Figure 68b).

However, the deactivation occurs once the reaction temperature or the O_2_ partial pressure increases to a critical point (e.g., *T* > 600 K with a CO/O_2_ ratio of 1). This behavior can be attributed to the sintering-induced reductions of the active sites or the bulk oxidation of the Rh(Pt) NPs inducing decreases in the reactivities. It should also be noted that structure-sensitivities can arise in CO oxidation kinetics when the NP sizes become very small (e.g., smaller than 2 nm).^371^ This observation is correlated to the higher binding strength of CO on the undercoordinated sites present on the smaller NPs, thus resulting in a higher activation energy and a lower CO oxidation rate. Nevertheless, the characterization and CO oxidation studies on the silica-supported Pt-group NPs have successfully demonstrated the possibilities of using the 2D-silica in complex catalytic systems regarding the structure–activity relationships.

#### 5.3.2 Rh and Pt NPs on Silica/Mo(112) for C_2_H_4_ Hydroformylation and *n*-Heptane Dehydrocyclization

C_2_H_4_ hydroformylation (C_2_H_4_ + CO + H_2_) is a well-known reaction for aldehyde synthesis, where the CO insertion reaction into the adsorbed alkyl groups is an important intermediate step. It has been proposed that the C_2_H_4_ is first hydrogenated to form C_2_H_5_ species adsorbed on a Rh surface, which was followed by CO insertion to form acyl species and then hydrogenation to form propionaldehyde.^372^ McClure et al. have utilized the C_2_H_4_ hydroformylation on Rh/silica/Mo(112) under nearly atmospheric pressure conditions as a probe system to investigate the structure–activity relationships of the CO insertion reaction, such as the effects of the Rh NP size and the reactant gas condition on the reaction mechanisms.^363^

**Figure 69:**
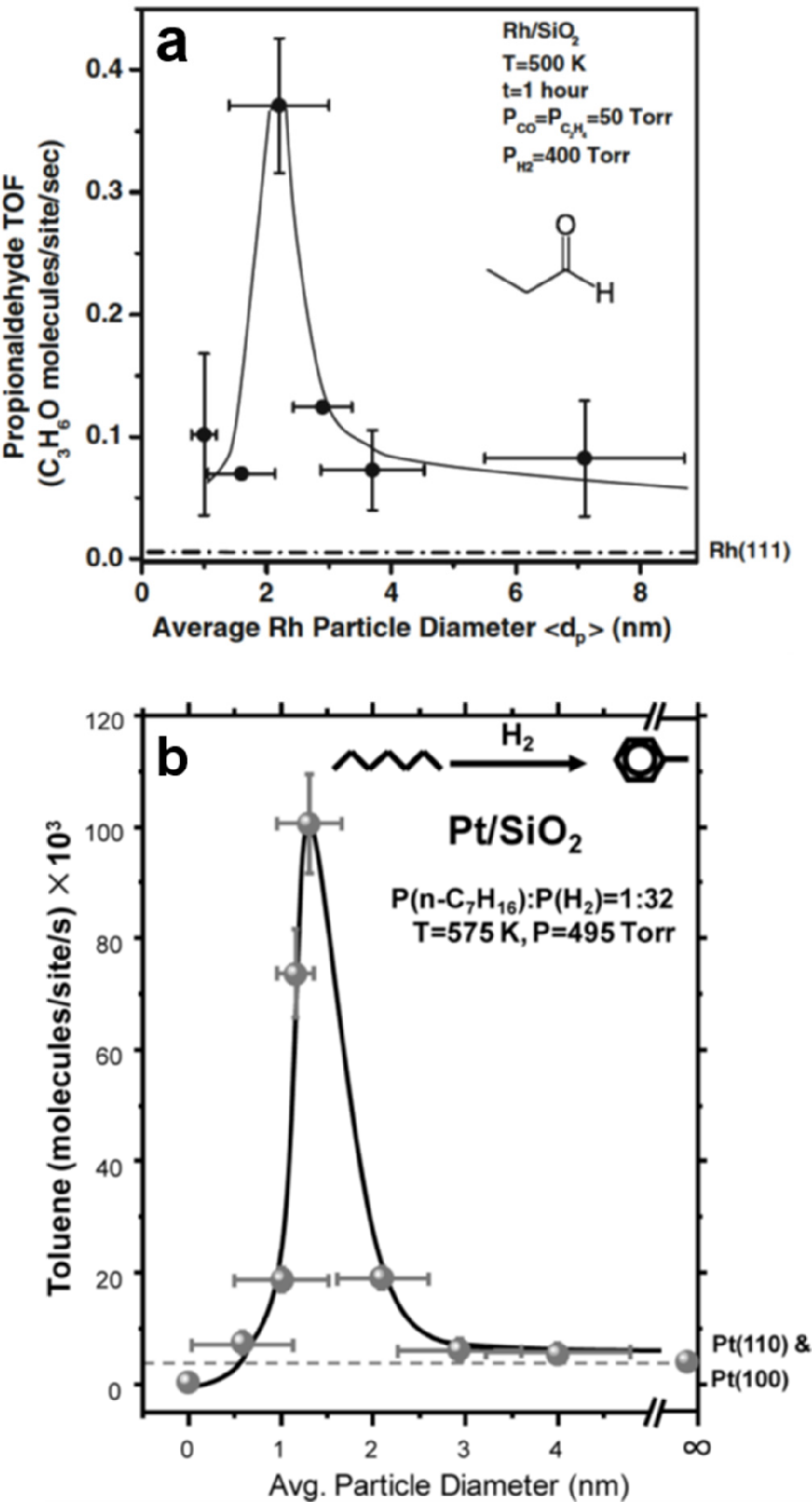
(a) Propionaldehyde formation rate [TOF in molecules/(site s)] versus average Rh particle size in C_2_H_4_ hydroformylation. Reaction conditions: CO/C_2_H_2_/H_2_ = 50:50:400 Torr at 500 K for 1 h. The dashed–dotted line shows the reactivity of the Rh(111) surface under the same reaction conditions. (b) Toluene formation rate [TOF in molecules/(site s)] versus average Pt particle size in *n*-heptane dehydrocyclization. Reaction conditions: *n*-C_7_H_16_/H_2_ = 15:480 Torr at 575 K. Reactivities of the Pt(110) and Pt(100) surfaces under the same reaction conditions are shown in the dashed line. Reproduced with permission from refs (363 and 365). Copyright 2011 National Academy of Sciences. Copyright 2012 American Chemical Society.

Figure 69a plots propionaldehyde formation rate [TOF in molecules/(site s)] versus average Rh NP size. The propionaldehyde TOF exhibits a strong dependence on Rh NP size, with a maximum (∼0.37) occurring at an Rh NP size of 2.5 nm, which is much higher as compared to that on the Rh(111) surface (∼0.0054) under identical reaction conditions (CO/C_2_H_2_/H_2_ = 50:50:400 Torr at 500 K). The numbers of total Rh sites on Rh NPs (with a 7.1 nm diameter) and Rh(111) are pretty close to each other (i.e., 1.3 × 10^15^ and 1.6 × 10^15^ Rh sites/cm^2^, respectively), while there is nearly an order of magnitude increase in propionaldehyde TOF on 7.1 nm Rh NPs. Evidently, the silica/Mo(112) support plays an important role in the CO insertion reaction to form propionaldehyde. The enhancement of the propionaldehyde TOF for the Rh NP size from 7.1 to 2.5 nm can be correlated with an increase in the number of undercoordinated “steplike” sites since the propionaldehyde formation occurs more favorably on undercoordinated sites. However, there is a significant decrease in the propionaldehyde TOF for Rh NP sizes below 2.5 nm, indicating that other factors must be involved for these very small Rh NPs on silica/Mo(112). Complementary polarization–modulation IRAS investigations under pure CO and C_2_H_4_/CO/H_2_ reaction conditions indicate the presence of Rh carbonyl species [e.g., Rh(CO)_2_ and Rh(CO)H] on small Rh NPs, which is correlated to the lower activity for propionaldehyde formation. Therefore, the observed Rh NP size effect is driven by two factors: On one hand, there is an increase in propionaldehyde formation on undercoordinated Rh sites as the Rh NP size is decreased to 2.5 nm; on the other hand, there is a decrease in propionaldehyde formation due to the presence of Rh carbonyl hydride species on Rh NPs with a size smaller than 2.5 nm.

A similar particle-size-dependent reaction was also observed for the *n*-heptane dehydrocyclization on silica/Mo(112)-supported Pt NPs (Figure 69b).^365^ The mechanism of the *n*-heptane dehydrocyclization is believed to occur through C6 ring closure and then to be followed by dehydrogenation.^373^ Generally, the dehydrogenation is considered a structure-sensitive reaction under various conditions.^374^ Lundwall et al. have found that the toluene formation rate during *n*-heptane dehydrocyclization increases as the Pt NP size is decreased from 4 to 1.5 nm. Again, this observation is related to the maximum concentration of undercoordinated sites in ∼1.5 nm Pt NPs, which is consistent with a reaction that would require 6-fold coordinated sites.^366^ However, as the Pt NP size is further decreased below 1.5 nm, the reaction rate turns out to decrease, which is most likely due to a loss of geometric and electronic effects required for dehydrocyclization.^374,375^ Interestingly, it was found that the silica/Mo(112)-supported Pt NPs sustain their activity, while the unsupported single-crystalline Pt surfaces deactivate over the same period of reaction time due to faster carbonaceous buildup. The spillover of carbon atoms from the Pt NPs onto silica/Mo(112) support may be responsible for the longer reactivity in Pt/silica/Mo(112), which needs further investigations. These studies therefore provide insights into the structure–activity relationships and offer bridges between supported and unsupported NPs under different reaction conditions.

#### 5.3.3 Pd–Cu Alloy NPs on Silica/Ru(0001) for Acetylene Hydrogenation

It should be mentioned that model catalysis systems chosen by Goodman and co-workers, as discussed above, were based on thick silica layers on a Mo(112) substrate. Sorek et al. have recently studied the support effects on Pd–Cu alloy NPs for selective acetylene hydrogenation by using a BL silica/Ru(0001) substrate and native silicon dioxide substrate.^376,377^ The Pd–Cu alloy NPs (5 ± 2 nm) with different Pd/Cu composition ratios were deposited on both substrates via the water buffer layer-assisted growth method.^378^ It was found that the Pd–Cu alloy NPs on BL silica/Ru(0001) have much higher thermal stability and sintering resistance than the ones on SiO_2_/Si(100) even under 0.2 mbar acetylene at 600 K, which presumably benefited from the charge transfer through the thin silica bilayer to the ruthenium substrate.^269,379^

**Figure 70:**
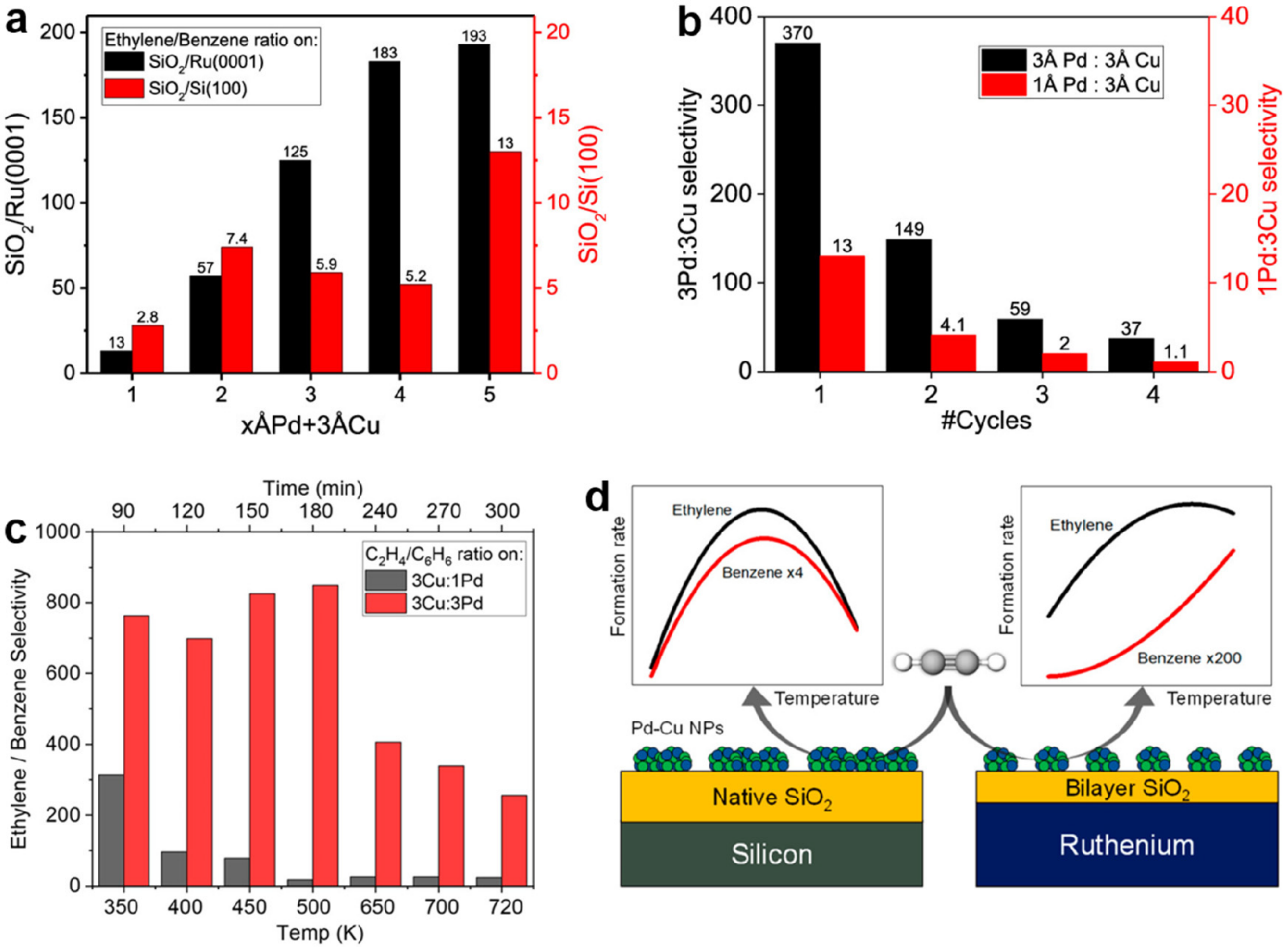
(a) Products’ selectivity (ethylene/benzene) of the acetylene hydrogenations as a function of the elemental composition of the Pd–Cu NPs on silica/Ru(0001) and SiO_2_/Si(100), respectively. Cu is fixed at 3 Å while the Pd varies from 1 to 5 Å. (b) Products’ selectivity (ethylene/benzene) of the acetylene hydrogenations as a function of the multiple reaction cycles for Pd–Cu NPs on silica/Ru(0001). (c) Products’ selectivity (ethylene/benzene) of the acetylene hydrogenations as a function of reaction temperatures. (d) Schematic diagram of the acetylene hydrogenations on Pd–Cu NPs on SiO_2_/Si(100) and silica/Ru(0001). Reproduced with permission from refs (376 and 377). Copyright 2019 and 2020 American Chemical Society.

A critical aspect in acetylene hydrogenation toward ethylene is to prevent both overhydrogenation and cyclotrimerization.^380^ Sorek et al. have further investigated the effect of elemental compositions in Pd–Cu NPs on the acetylene hydrogenation (i.e., the selectivity of ethylene/benzene) as shown in Figure 70a. The highest ethylene selectivity and formation rate were found for the Pd–Cu NPs on BL silica/Ru(0001) with the elemental composition of 5Pd/3Cu, indicating that the Pd atoms in alloy NPs together with the stabilization effect from the silica/Ru(0001) support play the most critical roles in selectivity and reactivity toward ethylene. It is important to mention that the alloy NPs are significantly more efficient than pure Pd or Cu NPs due to the synergetic effect.^381^ The long-term activity of the Pd–Cu NPs on silica/Ru(0001) was examined by performing consecutive reactivity cycles by adsorbing acetylene at 110 K with subsequent annealing up to 400 K in UHV (Figure 70b). Due to the acetylene decomposition and carbon accumulation on the alloy NPs surfaces, there is a significant decrease in the selectivity toward ethylene after the first three runs. However, the morphology and the bimetallic alloy structure of the alloy NPs were not affected as revealed by scanning electron microscopy (SEM) and TEM measurements.

In addition to the UHV studies, the acetylene hydrogenation was also conducted at near ambient pressure conditions (0.5 mbar acetylene) in order to correlate the reactivity/selectivity in both pressure regimes and therefore to better understand the pressure-gap issue in the model catalytic reactions (Figure 70c,d).^377^ Interestingly, the results are similar to those found under UHV conditions. Possible structural transformations of the Pd–Cu NPs during the reactions at elevated pressures may need to be further established.

## 6 Composite 2D-Silica Systems

Composite 2D-silica systems have the potential to become important in nanotechnology.^382,383^ As we know from the discussions in section 2, a silica monolayer is bound to a metal support through Si–O–metal linkages. In contrast, silica bilayers are weakly bound to the support via dispersive forces. There have been few attempts to combine 2D-silica with other 2D materials to form hybrid 2D structures, such as intercalate graphene underneath the silica layers,^80^ or vice versa.^384−388^ These layered silica–-graphene heterostructures may be interesting for nanotechnological applications^385^ and may also provide a basis for the development of new-generation 2D systems with unique properties.

### 6.1 Silica/Silicon-Carbide Hybrid Film

**Figure 71:**
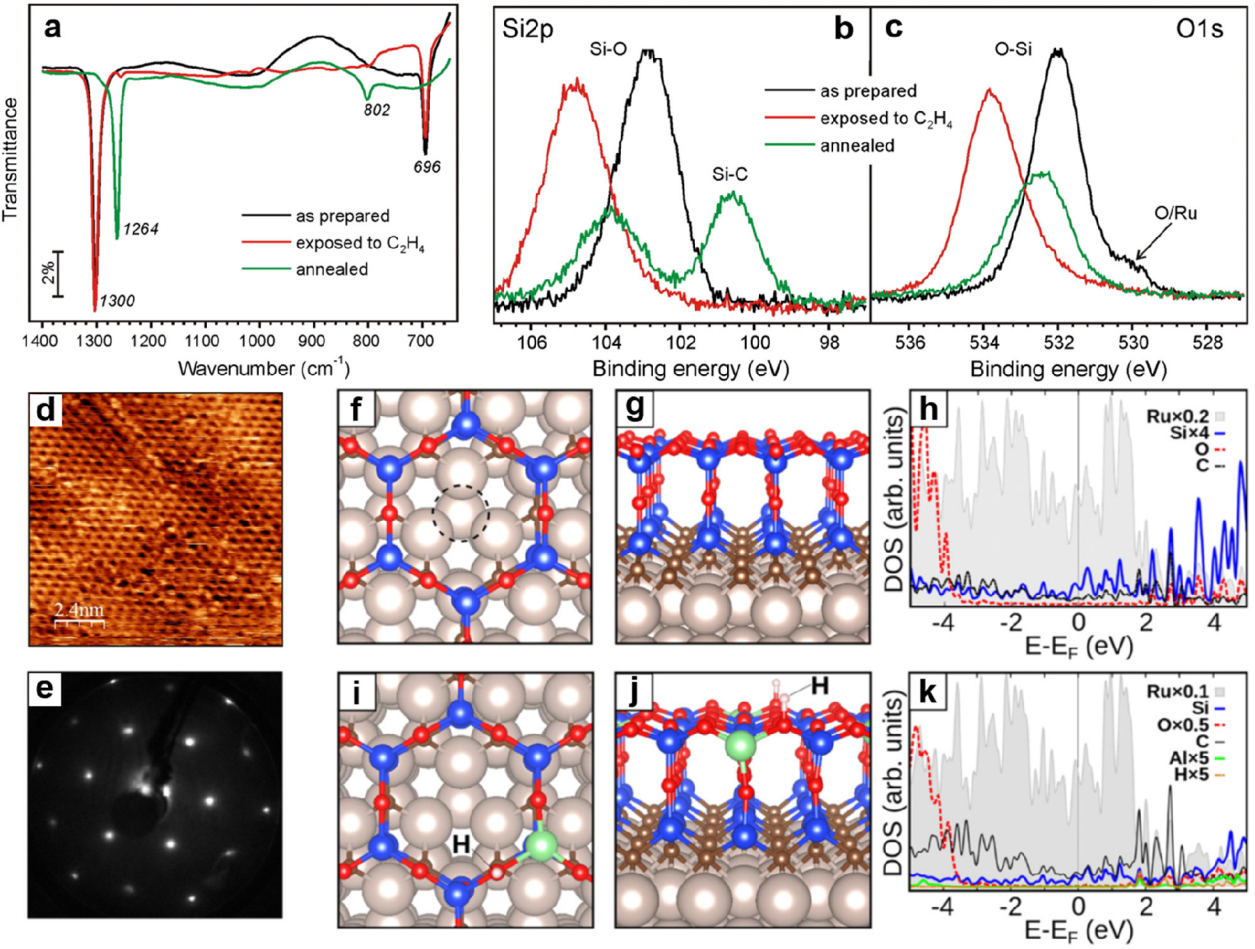
(a–c) IRAS and XPS of the silica bilayer as prepared (black), after exposure to 10 mbar ethylene at 450 K (red), and subsequent annealing in UHV at 1100 K (green, i.e., the formation of silica/silicon-carbide hybrid). (d, e) STM image (*U*_s_ = −1.0 V, *I* = 0.1 nA) and the corresponding LEED pattern (60 eV) of the silica/silicon-carbide hybrid. (f–h) Structure (top and side view) and projected DOS of the silica/silicon-carbide hybrid on Ru(0001) (i.e., SiC_*x*_O_2–*x*_, *x* = 0.75). The center of the hexagon in panel f is above a Ru-hcp position as indicated by the dashed circle. (i–k) Structure (top and side view) and projected DOS of a silica/silicon-carbide hybrid with an Al–OH unit at the top silica layer. Reproduced with permission from refs (97 and 98). Copyright 2014 Elsevier B.V. Copyright 2016 IOP Publishing.

In the attempts to intercalate graphene at the BL silica/Ru(0001) interface, Yang et al. have accidentally fabricated a well-ordered hybrid structure consisting of single-layer silica on top of a silicon-carbide monolayer on Ru(0001).^97^ The as-prepared BL silica/Ru(0001) was first exposed to 10 mbar ethylene at 450 K and then annealed in UHV at 1100 K. The ethylene or other hydrocarbon is commonly used for graphene growth on metals. As shown in Figure 71a, the bilayer structure remains intact upon ethylene exposure at 400 K, but the ethylene indeed penetrates the silica bilayer and dissociates on the Ru(0001) surface, possibly resulting in a layer of carbonaceous species that considerably shifts the core-levels to higher binding energy regions in XPS (Figure 71b,c). The transformation into a silica/silicon-carbide hybrid only occurs after UHV annealing at 1100 K as inferred from IRAS via the observation of a strong band at 1264 cm^–1^ and a weaker band at 802 cm^–1^ (Figure 71a). At the same time, the intensity of the O 1s core-level emission peak is roughly decreased by a factor of 2, and the Si 2p peak emission is split. The two resulting, equally populated species (i.e., Si–O and Si–C; Figure 71b,c) point to the formation of silicon-carbide, where half of the oxygen ions in the silica bilayer are replaced by carbon. This conclusion is plausible since the carbon atoms are adsorbed on the Ru surface prior to UHV annealing, which can replace the oxygen atoms in the bottom layer of the silica during the annealing process.

Further DFT studies by Schlexer et al. confirm the stability of this structural model [SiC_*x*_O_2–*x*_/Ru(0001), *x* = 0.75, i.e., single layer silica placed on top of a SiC-like monolayer formed on Ru(0001) as shown in Figure 71f,g^98^]. This silica/silicon-carbide hybrid model is very similar to those proposed by Heinz and co-workers for ultrathin silica films grown on SiC(0001).^49^ With the aid of DFT calculations, Schlexer et al. have explored the physical and chemical properties of the silica/silicon-carbide films compared with those of the silica bilayer. It was found that the center-hcp orientated (i.e., the center of the hexagon is above a Ru-hcp site as shown in Figure 71f) silica/silicon-carbide hybrid is the most stable one with a very large adhesion energy of −510 meV/Å^2^. The strong binding is due to the formation of covalent bonds (C–Ru) to the metal surface. Specifically, the C atoms are coordinated by two Si atoms, leaving in principle two valence electrons on C, which are available for bonding to the Ru surface (Figure 71h). The SiC_*x*_O_2–*x*_/Ru(0001) also exhibits two active IR modes, but one is red-shifted from 1296 to 1289 cm^–1^, while the other is blue-shifted from 642 to 744 cm^–1^ as compared to the silica bilayer. These computational results are consistent with the experimental observations and correspond to the asymmetric stretching of the vertical Si–O–Si bond and symmetric stretching of the parallel Si–O–Si bond in the top layer, respectively (Figure 71a).

Al-doping in SiC_*x*_O_2–*x*_/Ru(0001) was also considered by the DFT calculations (Figure 71i–k). In contrast to the aluminosilicate bilayer,^10^ the Al-doping is favored in the top silica layer, being 0.34 eV more stable than in the bottom layer. This difference is a direct consequence of replacing oxygen atoms with carbon atoms and forming a different interface bond in the silicon-carbide layer. The Al-doping can be accompanied by the appearance of surface hydroxyls, which is explained by steric effects.

Such silica/silicon-carbide hybrid systems constitute another example of the diversity of 2D materials with respect to the corresponding bulk structures, such as silicon-oxicarbide.^389^ It is important to mention that, despite the similar structure of the top layer and the identical metal support, the silica/silicon-carbide hybrid behaves quite differently from the silica bilayer, especially in terms of electronic properties.

### 6.2 Silica Intercalated under Graphene

Direct intercalation of insulating silica layers between epitaxial graphene and the metal substrate has been proposed as a transfer-free technique for fabricating graphene-based electronic devices.^385,387^ For example, Lizzit et al. have reported the formation of an amorphous thin silica film between the graphene and the Ru(0001) support by sequential exposure to silicon and oxygen, as inferred from XPS and nanoscale multipoint probe techniques.^385^ However, detailed intercalation mechanisms remain controversial on whether the Si migrates through the atomic defects of the graphene layer,^390^ directly via a Si–C exchange,^386^ or by way of cooperative interactions.^391^ Nevertheless, Guo et al. recently demonstrated the synthesis of thin crystalline silica or thicker amorphous silica at the graphene/Ru(0001) interface by stepwise intercalation of silicon and oxygen.^388^

**Figure 72:**
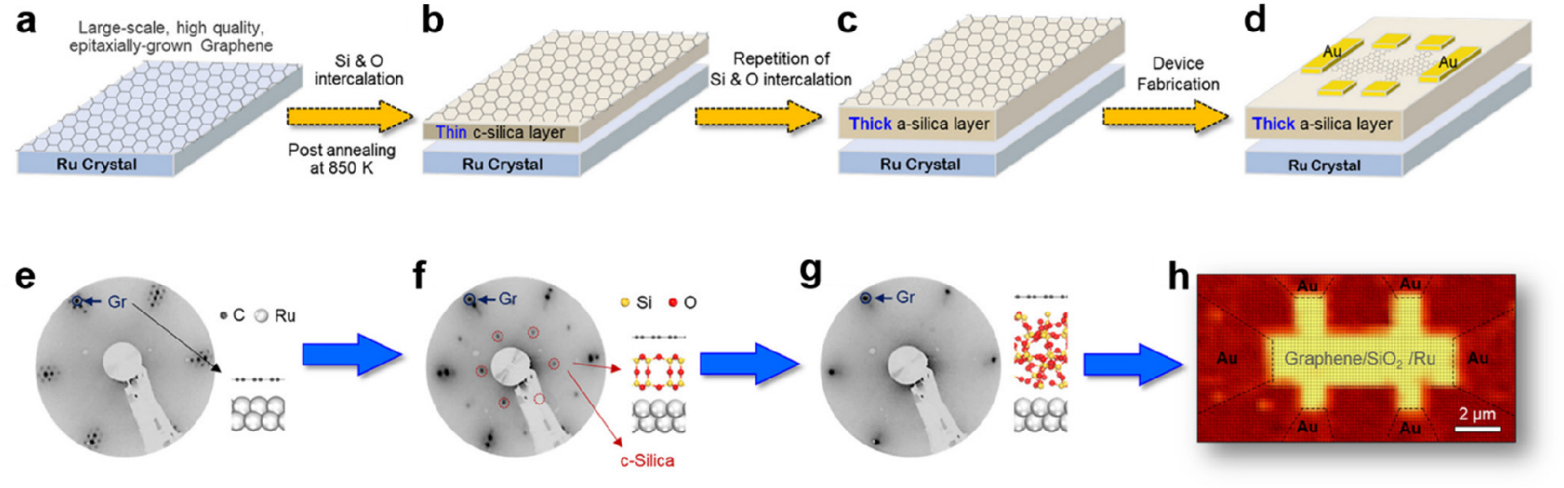
Intercalation of silica layers at the graphene/Ru(0001) interface for electronic-device fabrication. (a–d) Schematic diagrams show the sample preparation and device fabrication processes. (e–g) Corresponding LEED patterns and structure models for the sample in the preparation stages. (h) Graphene G-peak intensity mapping that shows the skeleton of the graphene Hall-bar device. Reproduced with permission from ref (388). Copyright 2020 American Chemical Society.

As shown in Figure 72, starting with the epitaxial growth of single-crystalline graphene on Ru(0001), a moderate amount of silicon was deposited on top of graphene, which was followed by UHV annealing at 900 K. Afterward, the silicon-intercalated sample is exposed to oxygen at 600 K and then annealed at 850 K to form silicon dioxide at the graphene/Ru(0001) interface. The silica becomes thicker after several cycles of intercalations. For the thin intercalated-silica layer (Figure 72b,f), Guo et al. suggested that it was a crystalline silica bilayer as judged by the LEED pattern (2 × 2 superstructure) and the cross-sectional microscopy (∼0.5 nm in thickness). This observation is surprising because the crystallization temperature needed for silica bilayer formation is around 1100 K.^6^ The confinement effect by the graphene cover plays a role in decreasing the crystallization temperature via stabilizing possible metastable silica polymorphs (see section 2.2.1.5). However, more detailed studies are still needed in order to reveal the intercalation mechanisms.

It should be noted that the graphene structure remains intact after silica intercalation according to the STM image and Raman spectra. The insulating nature of the thick amorphous silica (∼1.8 nm) was verified by transport measurements. The device quality of the corresponding graphene was further confirmed by magneto-transport measurements on the *in situ* fabricated Hall-bar device (Figure 72d,h). The hybrid graphene/silica/Ru system developed in this work provided a platform for potential graphene-based electronic devices with transfer-free techniques.

### 6.3 Transferability of 2D-Silica

Although 2D-silica is a promising candidate as an ultrathin dielectric in nanoelectronics as has already been demonstrated in Figure 72, so far, this new material has only been studied on its respective growth substrates. Moreover, the reliable preparation of freestanding silica films remains challenging. Therefore, in analogy to graphene, the ability to transfer a 2D-silica film from the growth substrate to another desired substrate is urgently required for exploring composite 2D-silica systems in nanoarchitectures. The bending rigidity (*k*), one crucial mechanical property, has recently been measured with inelastic helium atom scattering^392^ on BL silica/Ru(0001).^160^ The *k* was determined to be 8.8 ± 0.5 eV, roughly consistent with the theoretical values for a freestanding crystalline 2D-silica.^137^ For comparison, the bending rigidity of copper-supported single-layer graphene has a *k* value of 1.30 ± 0.15 eV.^393,394^ The relatively higher bending stiffness in 2D-silica is reasonably expected since it has a “three-atom-layer” structure, making the silica bilayer a more robust planar structure under thermal or mechanical perturbations. However, the mechanical behavior (e.g., ductility and tensile strength) of a vitreous 2D-silica film may significantly depend on the network heterogeneity. For example, under athermal quasistatic tensile deformations, the ductility of a vitreous 2D-silica film increases with an increase in its network heterogeneity.^395−397^

**Figure 73:**
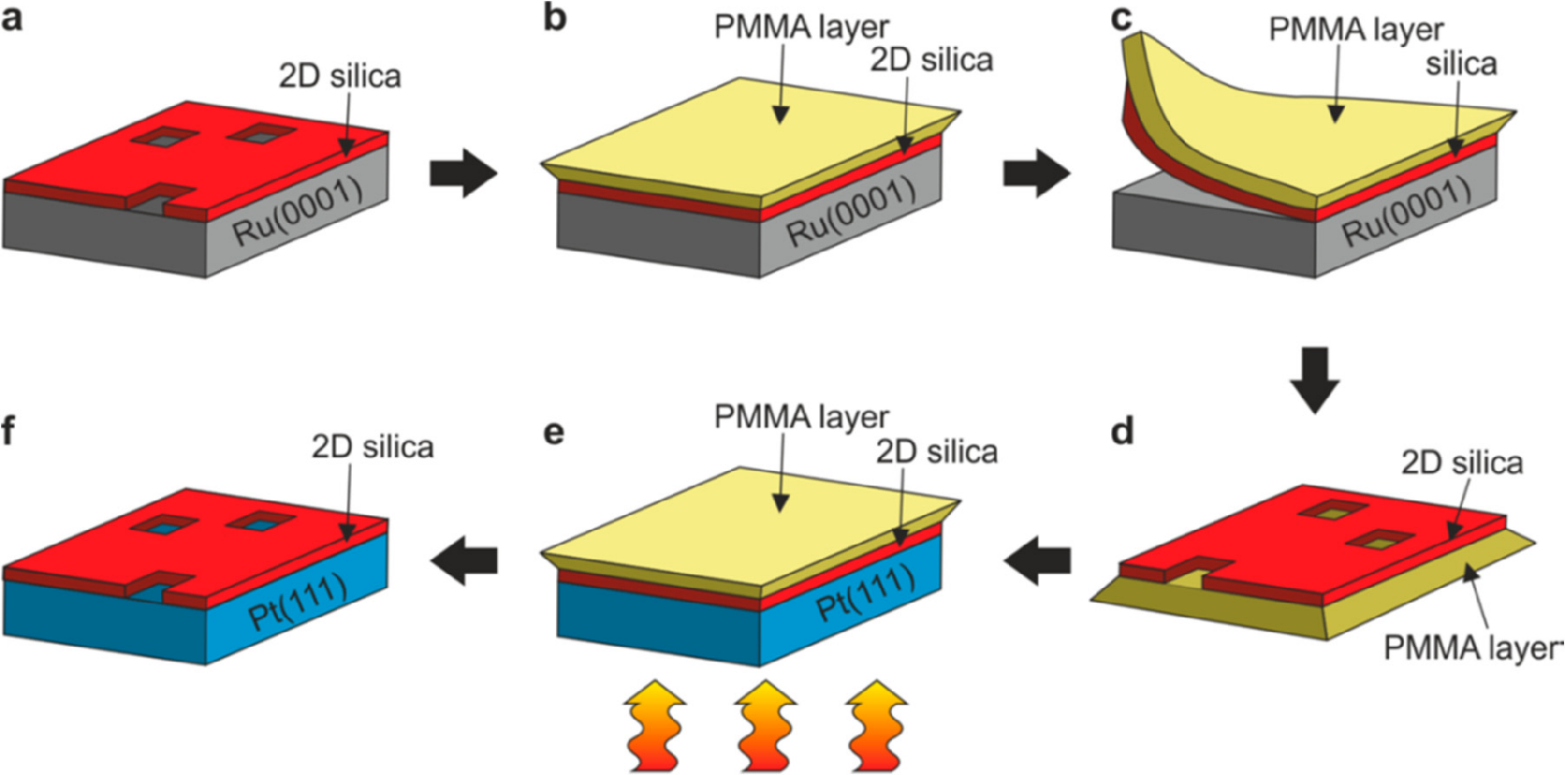
Schematic showing the transfer procedure of a 2D-silica film. (a) As-prepared silica bilayer on Ru(0001). (b) Spin coating of the system with a PMMA layer. (c) Mechanical exfoliation of the PMMA/silica layers. (d) Silica is supported on the PMMA layer. (e) Placement of the PMMA/silica layers onto a clean Pt(111) substrate, followed by heating treatment. (f) Silica is supported on a Pt(111) substrate after removing the PMMA layer. Reproduced with permission from ref (28). Copyright 2016 American Chemical Society.

The mechanical exfoliation of the silica bilayer from the Ru(0001) substrate was first realized by Büchner et al.^28^ As shown in Figure 73, the transfer procedure mainly consists of two parts: exfoliation of the film from the growth substrate (Figure 73a–c) and the subsequent transfer to the desired substrate (Figure 73d–f). Several characterizations [LEED, AES, STM, and environmental scanning electron microscopy (ESEM)] have been carried out to verify the transfer of the 2D-silica films. Briefly, the atomic structure and morphology of the silica-bilayer are maintained perfectly on the new Pt(111) support without carbon residue on the sample. This mechanically exfoliated 2D-silica may be used as a basic building block for assembling insulating layers with precise thickness control. For example, stacking two or more silica bilayers would result in a tunable dielectric layer or provide an effective tunneling barrier.^398^ Moreover, by integrating this wide-band-gap 2D-silica film into the toolbox of 2D materials, the number of van der Waals heterostructures with promising applications can be significantly increased.

## 7 Conclusions and Perspectives

In the previous six sections, we have provided a comprehensive overview of the discovery, structure, and electronic and chemical properties of well-ordered 2D-silica films as well as the ways of modifying their properties so as to use these systems as model systems for heterogeneous catalysts. Since those 2D-silica films are atomically flat, they lend themselves to detailed characterizations at the atomic level using the entire toolbox of surface science.^399,400^ In particular, scanning probe techniques provide detailed insights into the structural properties and allow for the first time, in combination with LEED, a full structural characterization of the surface structures of both a crystalline phase and a vitreous phase of a bilayer film with SiO_2_ stoichiometry on a metallic substrate.

Those studies provided initial real space images, basically proving the original ideas of W.H. Zachariasen from 1932 concerning the structure of vitreous silica.^36^ Films with coexisting crystalline and vitreous areas have also been prepared, and initial studies of the crystalline–vitreous transformation were possible using LEED and LEEM studies on the temperature dependence of diffraction peaks, revealing an apparent activation energy consistent with an initiation of the process via the induction of Stone–Wales defects (see section 2.2.1).

As a perspective at this point, it would be an appealing goal to be able to follow this transformation at the atomic level using scanning probe techniques. Attempts in this direction are ambitious but are underway by setting up an STM system that allows for high-speed scanning at elevated temperatures,^401,402^ beyond what has been achieved previously by G. Ertl and co-workers.^403^

**Figure 74:**
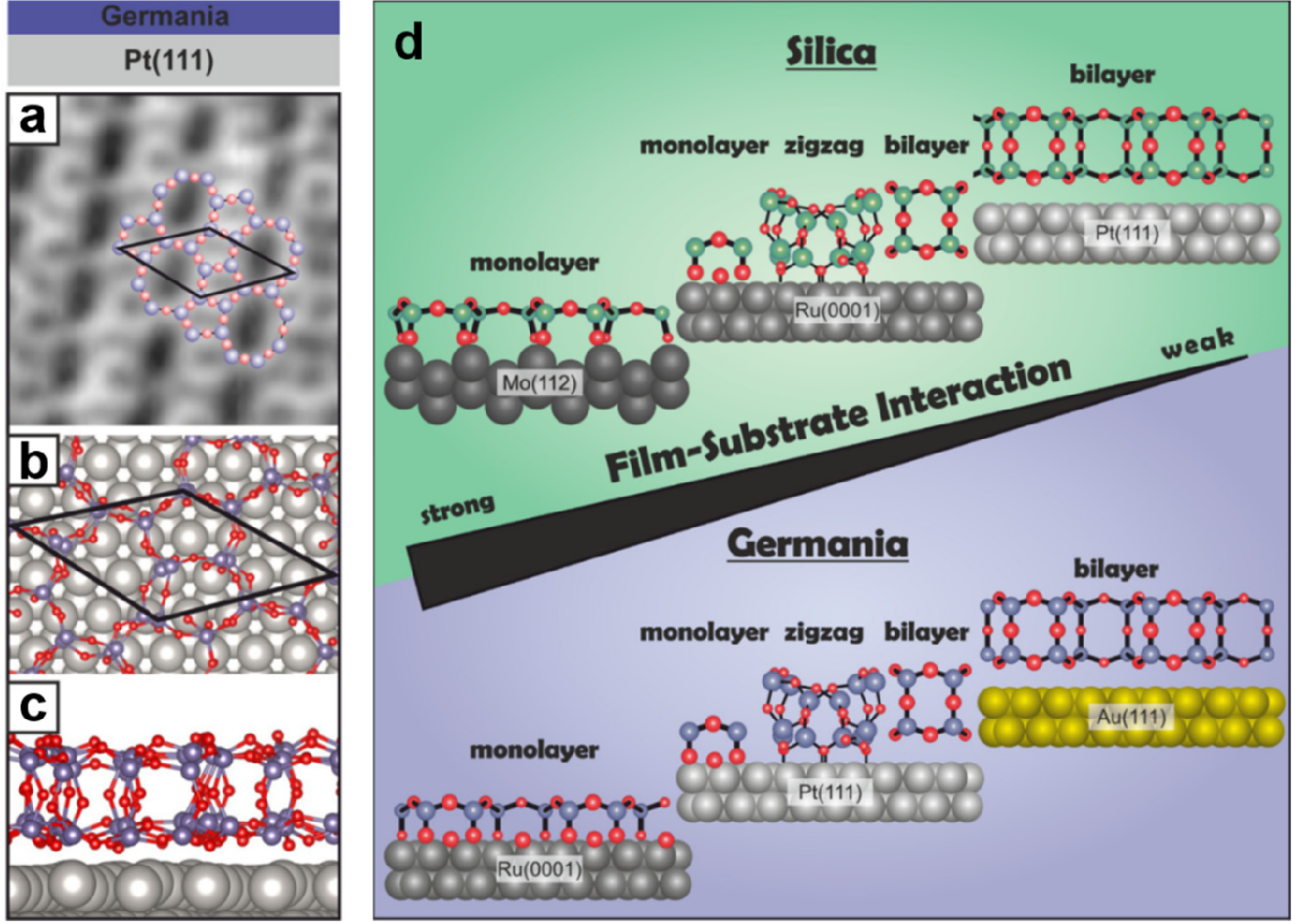
(a) STM image of a crystalline germania bilayer film supported on Pt(111) (3.8 nm × 3.8 nm, *U*_s_ = 0.3 V, *I* = 0.4 nA). The crystalline bilayer phase of germania forms an arrangement of 5- and 8-membered rings. (b, c) Top and side views of the most stable DFT cannulated model of the germania bilayer on Pt(111) (Ge with blue and O with red spheres). (d) The investigated films range from monolayer to bilayer coverage, where both the crystalline and the amorphous films contain characteristic [XO_4_] (X = Si, Ge) building blocks. The side-by-side comparison leads to a more general comprehension of the network structure of glass-former materials. Reproduced with permission from ref (405). Copyright 2020 The Authors. Published by Wiley-VCH GmbH.

Another perspective comes into play, namely, the possible preparation of another group of the IV oxide films, such as germania, which may expose similar structures and transformations, but at lower crystallization temperatures. Indeed, initial studies on germania films have been performed, and structural determinations have been started.^159,404,405^Figure 74 shows a comparison of a silica film and a corresponding germania film with structural schemes. Both can exist as vitreous films. Studies on the crystalline–vitreous transformation, corresponding to the one mentioned above for the silica film, reveal for the germania film a lowering of the transformation temperature by several hundred Kelvin, and may, in addition to interesting further studies on its properties, lower the demand on temperature stability for the STM. In looking at Figure 74, it is also obvious, and this has been addressed in section 2 of this Review in detail, that the substrate, onto which the silica film is grown, plays an important role with respect to the structural and electronic properties of the film. It was mentioned that, during the growth process, the lattice mismatch between the substrate and the film, the step density, the oxygen affinity of the metal support, as well as the deposition method play a decisive role. This has consequences for perspective studies, to be mentioned below, and is documented at this point referring again to the study of silica and germania films in comparison, which is summarized in Figure 74d. This allows us to understand in more general terms the crucial roles of the metal support for growth and possibly for the pathway from crystalline to amorphous ultrathin film growth. Analogous to 2D silica and germania, Altman et al. recently predicted the feasibility of forming similar corner-shared tetrahedra structures in 2D group III phosphate bilayers, such as AlPO_4_ and GaPO_4_.^207,406^ According to the calculations, only 7-membered rings are possible for the phosphates as compared to the silica bilayer, suggesting that the structures of AlPO_4_ and GaPO_4_ may be more easily controlled.

We have addressed in detail the use of 2D-silica films as supports for metal catalysts and have demonstrated that in several specific cases. Metal clusters might be grown on the silica film and, again, characterized with atomic precision (see sections 4.1, 4.2, and 5.3). Depending on the exposed ring sizes in crystalline and vitreous films or at phase boundaries, metal could be incorporated into the silica framework or even diffuse to the metal surface. Obviously, the metal used as support plays an important role in those processes by controlling electron transfer between the metal support and the species at the silica film surface or in the silica framework. A particularly interesting aspect, discussed in section 4.3, has been the incorporation of noble gases, such as Xe, into the silica framework, which turns out to be initiated by first ionizing the gas atoms, so they lose an outer electron, which effectively shrinks its size so the ions may enter the framework; then, the metal support effectively provides electrons to neutralize the gas atoms after incorporation. Many of those aspects are influenced by the stiffness, or rather the flexibility, of the framework. We have addressed in section 3.1 the possibility of substituting silicon atoms within the framework by a number of different species, including carbon and also metal atoms. An exciting perspective again refers to using Ge as a substituent. Corma et al. proposed to substitute Ge into zeolite frameworks in order to increase the flexibility of the framework, which would influence diffusion within it.^407^ By inspection of Figure 74, it is obvious that the variation and greater flexibility of the O–Ge–O bond angles are the cause of this increased flexibility. As a perspective, this could be explored for the silica films to influence diffusion in and out as well as through the film framework and influence electronic communication between the diffusing species and the metal support.

Those ideas are also closely connected to the studies of reactions in confined space, which have been summarized in section 5.1. One example discussed in detail referred to the formation of water at the metal surface via diffusion of hydrogen molecules through the framework to react with adsorbed oxygen atoms and the formed water molecules to diffuse through the framework to escape. The stiffness of silica, of course, limits the size of the species involved to a large extent. The above-discussed perspective studies to influence the flexibility of the framework would also possibly allow the use of more diverse species to be used in studies of reactions in confined space.

**Figure 75:**
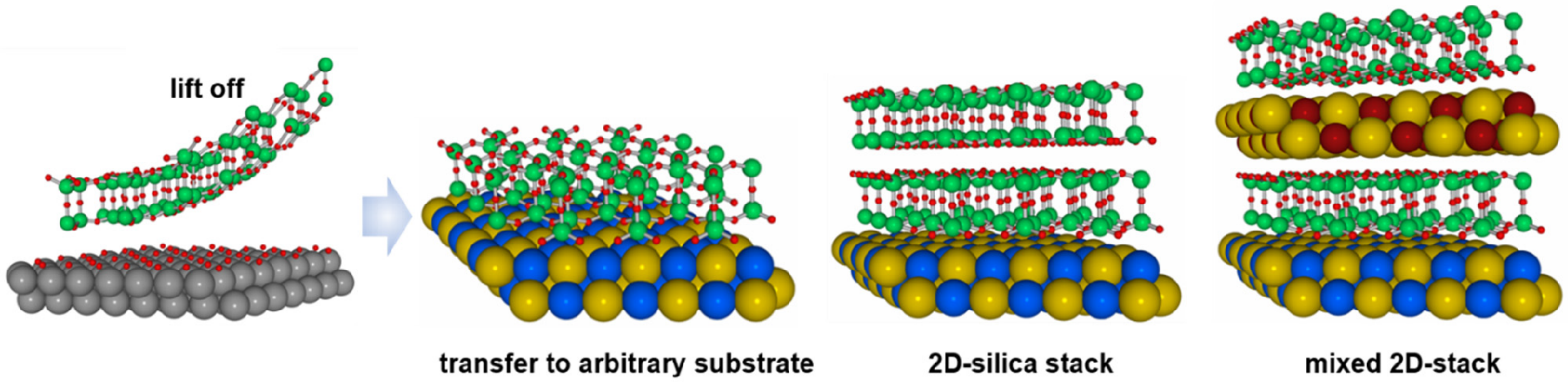
Schematic drawing of the removal of a 2D-silica film and its placement onto another arbitrary substrate, as well as a stack of pure silica films.

The present Review mainly deals with studies of 2D-silica films bound to a metallic substrate. It is mentioned toward the end of the Review in section 6.3 that the 2D film may be removed from the metal substrate by similar techniques used in connection with other 2D materials, such as graphene. Such a freestanding film may be attached to another metal substrate without changing the structure of the film, as proven via STM imaging. This transferability opens another perspective for the use of 2D-silica films: If one could demonstrate that the film may be placed on any other substrate (schematically shown in Figure 75), for example, another oxide, such as a perovskite, whose electronic properties may be controlled to a large extent through defect management, one would be in a position to build up specific electronic devices systematically. Here, the largely insulating properties of the 2D-silica films with estimated band gaps of 5.3–7.36 eV would fit well.^29,137,138^

The availability of transferable films would also open up other perspective options. For example, one might be able to stack 2D-silica films and thus create well-ordered silica film stacks of arbitrarily chosen thickness. If one would find a way to create chemical bonds between the stacked films without destroying the structure, this could lead to thicker well-ordered silica films, which is difficult, as we refer to in the Review, to achieve by simple growth. Also, if one were to stack silica films with other films or grown layers, even organic layers, in between, one would be in a position to build up and design systems with potentially very interesting properties and applications.

In summary and in conclusion, the Review has demonstrated that 2D-silica films of very well-defined and characterized structural, chemical, and electronic properties may be prepared, and thus, the 2D-silica film is another full member of the family of 2D materials and may be used in the future in applied science and engineering.

## Abbreviations

2D: Two-dimensional
3D: Three-dimensional
AES: Auger electron spectroscopy
AFM: Atomic force microscopy
ALD: Atomic layer deposition
APDB: Antiphase-domain-boundaries
APXPS: Ambient pressure X-ray photoelectron spectroscopy
ARPES: Angel-resolved photoemission spectroscopy
BE: Binding energy
BL: Bilayer
BZ: Brillouin zone
CBM: Conduction band minimum
CVD: Chemical vapor deposition
DDO: Directed distance orientation
DFT: Density functional theory
DOS: Density of states
EELS: Electron energy-loss spectroscopy
ESEM: Environmental scanning electron microscopy
FAD: Fast atom diffraction
fwhm: Full width at half-maximum
HOMO: Highest occupied molecular orbital
HREELS: High-resolution electron energy-loss spectroscopy
IBT: Ion beam triangulation
IRAS: Infrared reflection–absorption spectroscopy
LDOS: Local density of states
LEED: Low-energy electron diffraction
LEEM: Low-energy electron microscopy
LUMO: Lowest unoccupied molecular orbital
MBE: Molecular beam epitaxy
ML: Monolayer
MLE: Monolayer equivalent
MOF: Metal–organic framework
MOSFET: Metal-oxide-semiconductor field-effect transistor
NMR: Nuclear magnetic resonance
NN: Nearest neighbors
NPs: Nanoparticles
PCF: Pair correlation function
PDF: Pair distribution functions
PDOS: Projected density of states
PEEM: Photoemission electron microscopy
RHEED: Reflection high-energy electron diffraction
SEM: Scanning electron microscopy
STEM: Scanning transmission electron microscopy
STM: Scanning tunneling microscopy
STS: Scanning tunneling spectroscopy
TEM: Transmission electron microscopy
TOF: Turnover frequency
TPD: Temperature-programmed desorption
UHV: Ultrahigh vacuum
VBM: Valence band maximum
VDW: van der Waals
WF: Work function
XRD: X-ray diffraction
XPEEM: X-ray photoemission electron microscopy
XPS: X-ray photoelectron spectroscopy
